# Chromosome-level genome assembly of *Aldrichina grahami*, a forensically important blowfly

**DOI:** 10.1093/gigascience/giaa020

**Published:** 2020-03-19

**Authors:** Fanming Meng, Zhuoying Liu, Han Han, Dmitrijs Finkelbergs, Yangshuai Jiang, Mingfei Zhu, Yang Wang, Zongyi Sun, Chao Chen, Yadong Guo, Jifeng Cai

**Affiliations:** 1 School of Basic Medicine, Central South University, Changsha, Hunan Pro, China; 2 Nextomics Biosciences, Wuhan, Hubei Pro, China; 3 Institute of Apicultural Research, Chinese Academy of Agricultural Sciences

**Keywords:** *Aldrichina grahami*, blowfly, necrophagous, forensic entomology, minimum postmortem interval, genome assembly

## Abstract

**Background:**

Blowflies (Diptera: Calliphoridae) are the most commonly found entomological evidence in forensic investigations. Distinguished from other blowflies, *Aldrichina grahami* has some unique biological characteristics and is a species of forensic importance. Its development rate, pattern, and life cycle can provide valuable information for the estimation of the minimum postmortem interval.

**Findings:**

Herein we provide a chromosome-level genome assembly of *A. grahami* that was generated by Pacific BioSciences sequencing platform and chromosome conformation capture (Hi-C) technology. A total of 50.15 Gb clean reads of the *A. grahami* genome were generated. FALCON and Wtdbg were used to construct the genome of *A. grahami*, resulting in an assembly of 600 Mb and 1,604 contigs with an N50 size of 1.93 Mb. We predicted 12,823 protein-coding genes, 99.8% of which was functionally annotated on the basis of the *de novo* genome (SRA: PRJNA513084) and transcriptome (SRA: SRX5207346) of *A. grahami*. According to the co-analysis with 11 other insect species, clustering and phylogenetic reconstruction of gene families were performed. Using Hi-C sequencing, a chromosome-level assembly of 6 chromosomes was generated with scaffold N50 of 104.7 Mb. Of these scaffolds, 96.4% were anchored to the total *A. grahami* genome contig bases.

**Conclusions:**

The present study provides a robust genome reference for *A. grahami* that supplements vital genetic information for nonhuman forensic genomics and facilitates the future research of *A. grahami* and other necrophagous blowfly species used in forensic medicine.

## Background

Forensic entomology focuses on the application of insects and other arthropods in the medicolegal investigation. Studying the development rate of insect colonizers on the corpse and insect succession patterns during corpse decomposition can assist in the estimation of the minimum postmortem interval (minPMI), which represents the main task of the forensic investigation [1–3]. In addition, insect evidence is helpful in the detection and recognition of wounds, the estimation of the duration of neglect or abuse, and the investigation of the cause of death [4–7]. The most important group of insects for forensic investigation is the Diptera, especially the necrophagous fly species of Calliphoridae [8, 9]. Flies of this fauna, usually called “blowfly,” consist of many species with a parasitic or necrophagous lifestyle [10, 11]. The reliable life cycle of these necrophagous flies can provide vital information for forensic entomologists or investigators to infer a relatively accurate minPMI under certain assumptions [8, 12–14].

**Figure 1:**
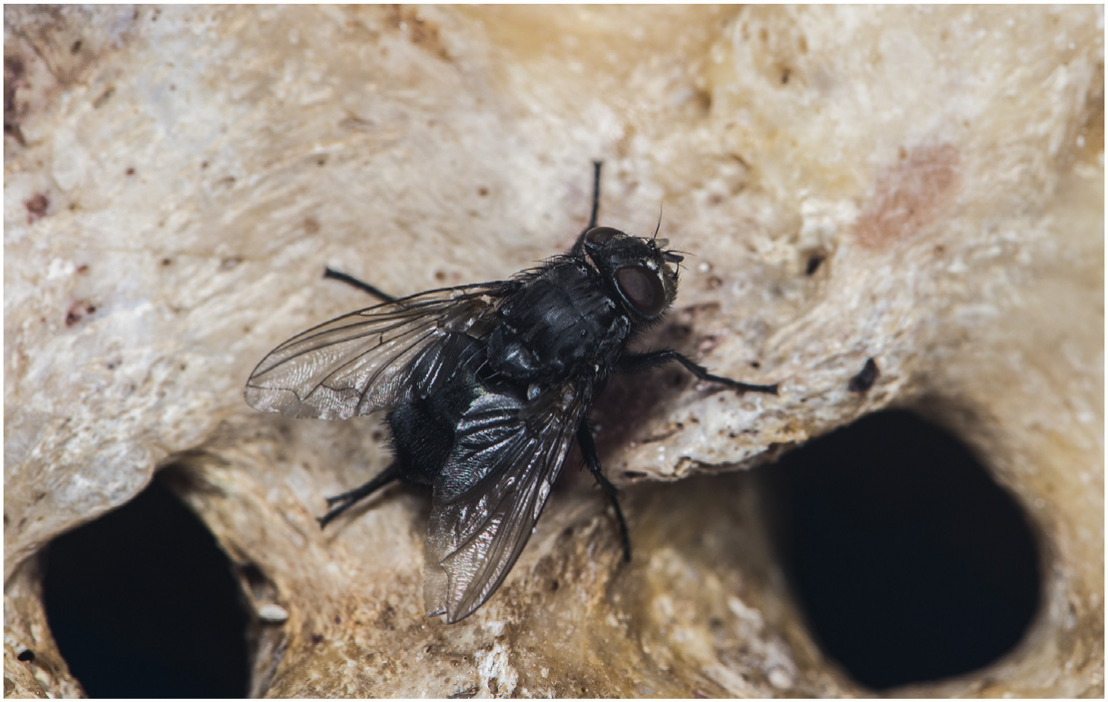
Female adult of *Aldrichina grahami* on a corpse.

*Aldrichina grahami* (Aldrich, 1930; NCBI:txid252811, homotypic synonym: *Calliphora grahami*) (Fig. 1) is a common blowfly species indigenous to East Asia [15, 16] that has expanded to the North American continent in the past several decades [17–19]. It usually breeds on carcasses or feces, posing a potential threat of contaminating human food [15]. *A. grahami* is a forensically important insect because of its necrophagous behavior, seasonal distribution, and particularly unique characteristics of low-temperature tolerance, all of which distinguish it from other necrophagous flies [20–22]. *A. grahami* is frequently the first species to colonize the corpse in early spring and late autumn, when the ambient temperature is relatively low. In some extreme cases this species can be the only colonizer [23, 24]. The information provided by the seasonal distribution pattern of *A. grahami* could be applied as a potential “season stamp” of the time of death in the PMI estimation, especially in the period when other insects are inactive [22, 25]. Moreover, the successful extraction and identification of human DNA material from gut contents of *A. grahami* and other blowfly larvae can provide important information about a missing corpse or help to interpret the evidence used for forensic investigation [26, 27]. The age-dependent altering pattern of cuticular hydrocarbons in larvae cuticle has great application potential in the forensic investigation [28, 29]. Besides the forensic importance, cases of myiasis caused by *A. grahami* have been reported routinely in China, especially when people travel back from undeveloped regions [30–33]. This blowfly species is also a potential transmitter of pathogens, such as the H5N1 influenza virus, which could cause serious public health problems in animals and humans [34].

Research on insect biochemistry and physiology prompts our deeper understanding of *A. grahami* [35–38]. Nuclear materials are primarily applied to distinguish *A. grahami* from sibling Diptera species [39–42]. Several researchers have described the development patterns of *A. grahami* under different environmental conditions [20, 21]. Nonetheless, the genome of *A. grahami* is still unavailable, which impedes its further applications in forensic research. Previous studies have indicated that variation at the genetic level has a detectable and potentially important influence on the length of development and the life cycle of the fly species among different geographic populations [43–45]. It was also recommended that such forensic investigations should be based on a high-quality genome reference of the investigated fly species [46–48]. Here we provide a chromosome-scale scaffolding of the genome assembly of this forensically important blowfly, using the Pacific BioSciences (PacBio) sequencing platform and chromosome conformation capture (Hi-C) method, which promotes the future research of forensic and medical science.

## Genome Sequencing and Assembly

### Sample preparation

The first generation of *A. grahami* was collected using beef liver as bait, in Changsha (Hunan Province, China) in March 2017. Species identification was performed through morphological and molecular methods. The fly species were distinguished following the morphological description by Fan [15]. Then cytochrome oxidase gene I (*COI*) as a molecular marker was amplified from the DNA of *A. grahami* using the previously described method (Primer F: 5´-TACAATTTATCGCCTAAACTTCAGCC-3´, R: 5´-CATTTCAAGCTGTGTAAGCATC-3´) [39]. After sequencing the amplification product (ABI 3730xl, USA), the result was searched by BLAST and deposited into the NCBI database (Accession No.: MN537823). It was recognized as belonging to *A. grahami*. The blowflies were bred for >20 generations in the laboratory of the School of Basic Medicine, Central South University, Changsha. Newly emerged and unmated female adults were used for DNA extraction.

After sample collection, the used tissues were immediately immersed into liquid nitrogen and stored at −80°C. DNA was extracted using the cetyltrimethyl ammonium bromide (CTAB) method followed by the introduction of Size-Selected 20 kb SMRTbell^TM^ Libraries for genomic DNA preparation. The quality of the extracted genomic DNA was checked using gel electrophoresis with 0.7% agarose. Then a Nanodrop spectrophotometer (Thermo Scientific, Wilmington, DE, USA) was used to calculate the DNA purity. The concentration of extracted material was examined by Qubit fluorimeter (Invitrogen, Carlsbad, CA, USA).

New males and females were sampled for transcriptome sequencing. After the extraction quality control and library construction, the (Illumina Inc., San Diego, CA, USA) was used to perform the RNA sequencing (RNA-seq). Five new female adults, with their wings dissected and gut removed, were used for library construction.

Every voucher specimen was assigned a unique code. All specimens were deposited in the forensic insect herbarium of the Department of Forensic Science, Central South University, Changsha, Hunan Province.

### Library construction and sequencing

Two libraries were constructed before sequencing. First a library of short insert length (400 bp) was constructed by Illumina TruSeq Nano DNA Library Prep Kits. The short-insert library sequencing was performed on the Illumina HiSeq X10 instrument at Genetron Health (Beijing, China) using the whole-genome shotgun sequencing (WGS) strategy. A total of 46.05 Gb of raw data were collected and subsequently filtered. Finally, 42.4 Gb of clean data for short reads were generated (Table S1).

The long-read library of 20 kb was prepared using a SMRTbell DNA Template Prep Kit 1.0 (PacBio p/n 10Tal-259-100). DNA fragments of ∼20 kb were generated by shearing genomic DNA material using a Covaris G-TUBE^TM^ (Kbiosciences p/n 520,079). The sheared genomic DNA was damage-repaired and end-repaired using polishing enzymes. The blunt-end ligation resulting from the exonuclease treatment was used to generate a SMRTbell template. After that, fragments with proper size (≥15 kb) were subsequently selected by the Blue Pippin device (Sage Science, Inc., Beverly, MA, USA). The DNA 12,000 Kit for Agilent Bioanalyzer 2100 (Agilent p/n 5067-1508) was used for figuring out the distribution of fragments with different sizes.

The prepared DNA template libraries were bound to the Sequel Polymerase 2.0 using Sequel Binding Kit 2.0 (PacBio p/n 100-862-200) in preparation for sequencing on the Sequel System. Finally, a DNA polymerase/template complex was formed according to the manufacturer's instructions. The enrichment of the larger fragments was improved by the MagBead (PacBio p/n 100-125-900) method. The long-insert size (20 kb) library was sequenced on the PacBio Sequel platform with Sequel SMRT cells 1 M v2 (PacBio p/n101-008-000), which has 1 movie of 600 minutes per Sequel SMRT cell at the Genome Center of Nextomics (Wuhan, Hubei, China). A total of 7 Sequel SMRT cells were processed. To remove low-quality bases or reads with adapters, the raw data were filtered on the basis of the sequencing platform with the default parameters. In total, 50.15 Gb of long-read clean data were obtained (Table S1). The mean length and the N50 of long subreads were 10.51 and 15.97 kb, respectively.

Hi-C libraries were constructed for *A. grahami* according to the improved Hi-C procedures [49]. After treatment with a 1% formaldehyde solution in phosphate-buffered saline at room temperature for 10 minutes to induce cross-linking, the single cell was made by trituration and filtration. The reaction was quenched by adding 2.5 M glycine to 0.2 M solution for 5 minutes. Nuclei were digested with 100 units of MboI, marked by biotin-14-dCTP (Invitrogen, Carlsbad, CA, USA), and then ligated by T4 DNA Ligase. After the reversal of cross-links, ligated DNA was purified and sheared to a length of 300–600 bp, at which point ligation junctions were pulled down by streptavidin beads and prepared for high-throughput sequencing. Sequencing was performed using the Illumina NovaSeq 6000 Sequencing System (Illumina Inc., San Diego, CA, USA) with PE150, yielding 74.24 Gb raw data (Table S1).

### Genome survey and genome assembly

The genome size was estimated on the basis of the equation *G* = *k*_num_/*k*_depth_, where the *k*_num_ is the total number of 17-mers, *k*_depth_ denotes the peak frequency of 17-mers estimated, and *G* represents the estimated genome size. Using Jellyfish v2.1.3 (Jellyfish, RRID:SCR_005491) [50], the number of 17-mers was counted as 29,131,491,603 from short clean reads, and the *k*_depth_ was 50. Therefore, the genome size of *A. grahami* was estimated as 582.63 Mb according to the above equation, and the heterozygosity rate of the *A. grahami* genome was ∼2.5% (Table S2, Fig. S1). FALCON is specifically designed to perform *de novo* assembly for PacBio long reads with ∼15% random errors [51]. After correction with FALCON (v0.4), the PacBio long reads were assembled with Wtdbg (v1.2.8) [52, 53], obtaining an initial assembly with length of ∼596.65 Mb and N50 contig of 1.93 Mb. To further improve the accuracy of the reference assembly, the following steps of polishing strategies were performed for the initial assembly. The pbalign (v.0.3.0) with default parameters was used for Quiver error correction, generating an error-corrected genome assembly of PacBio long reads. We used BWA v0.7.12 (BWA, RRID:SCR_010910) to map short reads to the error-corrected assembly. Then it was polished with Pilon v1.21 (Pilon, RRID:SCR_014731) to generate the second iteration of the assembled genome [54]. Finally, we obtained a polished assembly genome with a size of 600.09 Mb, including N50 contig of 1.93 Mb and 1,604 contigs (Table 1, Table S3). So far, the present genome has the longest N50 contig length among all the published genome assemblies of calyptratae flies of Diptera.

For the *A. grahami* genome, the assembly genome size (600 Mb) was almost the same as the genome size (582.63 Mb) estimated in 17-mer analysis. The sequencing quality was checked and the potentially contaminated contigs from other species were removed on the basis of the guanine-cytosine (GC) content and the depth of coverage of the genome assembly analyzed by the GC Depth analysis. The completeness of the assembly was evaluated by BUSCO v3.0 (BUSCO, RRID:SCR_015008). The result of BUSCO analysis indicated that our assembly covered 99.2% complete and 0.7% partial insect BUSCOs, with only 0.5% missed (Table S4).We also performed flow cytometry with propidium iodide staining to estimate the genome size of *A. grahami. Drosophila melanogaster* (strain w118) was used as the internal control with DNA content (pictogram: pg, 1 pg = 978 Mb) of 1C = 0.18 pg (175 Mb) [55]. The samples were prepared following the procedures of the previous study [56]. The flow cytometry was conducted using Accuri C6 (BD Bioscience, San Diego, CA, USA) with a 488-nm laser. Data were processed by FlowJo software (v7.6) (Fig. S2). The estimated genome sizes of male (679.2  ±  7.582 Mb, N = 6) and female (696.4  ±  6.618 Mb, N = 6) have no significant difference ( *P*-value = 0.12), showing no sexual dimorphism. However, estimated genome size is 18.1% larger than the *k*-mer–based genome size (582.63 Mb), and 14.6% larger than the assembly genome size (600.09  MB).

**Table 1:** An overview comparison of genome assembly and structure features in 5 calyptratae flies of Diptera

Parameter	*Aldrichina grahami*	*Lucilia cuprina*	*Glossina morsitans*	*Musca domestica*	*Phormia regina* (♀)
Sequencing platform	PacBio	Illumina	454/Illumina	Illumina	454/PacBio
Genome size (Mb)	600	458	366	692	550
No. of contigs/Scaffolds	1,604/7	74,043/4,436	24,071/13,807	-/20,487	192,662/-
Contig N50 (kb)	1,930	744.4	50	12	7.9
GC level (%)	31	29.3	34.1	35.1	26.2
Repetitive regions (%)	48.02	57.8	-	55	8.11
Function annotation (gene No.; %)	12,791; 99.8	12,160; 83.6	12,308; 99.5	14,180; 92.3	7,792; 94
Sequencing depth	86×	100×	160×	90×	44×
Completeness (BUSCO/CEGMA; %)	99.2	96	99	98	93.6

Genome completeness was assessed by BUSCO or CEGMA. Four genomes of calyptratae fly species were selected: *L. cuprina* [57]*, G. morsitans* [58]*, M. domestica* [59], and *P. regina* [45]. The genome version of *P. regina* female adult was chosen. NC: not reported.

## Functional Prediction and Genome Annotation

### Analysis of repeat genes

Simple sequence repeats (SSRs) are repeating sequences of 1–6 base pairs of DNAs that exist extensively in genomes. SSRs in the blowfly genome were identified by MISA (MISA, RRID:SCR_010765) [60]. MISA can distinguish and locate simple and complicated SSRs, of which the latter is always inserted by a certain number of nucleic acid bases. In total, 322,266 SSRs were found in the *A. grahami* genome.

We also analyzed the repetitive sequences in the *A. grahami* genome including in tandem repeats and transposable elements (TEs). TRF (TRF, v4.09) was used to annotate the tandem repeats [61]. A combination of *de novo* and homology-based approach was used to identify TEs at both the DNA and protein levels. First, we used RepeatModeler v1.0.8 (RepeatModeler, RRID:SCR_015027) [62] to construct a *de novo* repeat DNA library, which built a repeat consensus database with classification information. Then, the similar TEs were searched against the known Repbase library (Repbase 23.08) and *de novo*–based repeat library with RepeatMasker v4.0.6 (RepeatMasker, RRID:SCR_012954) [62]. RepeatProteinMask within the RepeatMasker package was applied to search against the TE protein database using a WU_BLASTX engine.

Overall, the *A. grahami* genome comprised 48.02% repetitive sequences, of which 43.69% were TEs. DNA with repetitive sequences accounted for 11.65% (Combined TEs) of the *A. grahami* genome, representing the most abundant repeat class (Table 2).

**Table 2:** Statistics of repeat sequence analysis

Type	RepeatMasker		LTR finder		RepeatProteinMask		RepeatModeler		Combined TEs	
	Length (Mb)	% in genome	Length (Mb)	% in genome	Length (Mb)	% in genome	Length (Mb)	% in genome	Length (Mb)	% in genome
DNA	42,174,497	7.03	0	0	41,341,346	6.89	50,464,704	8.41	69,933,653	11.65
LINE	10,505,716	1.75	0	0	19,838,372	3.31	26,169,690	4.36	34,333,817	5.72
LTR	4,789,075	0.8	15,966,332	2.66	5,778,730	0.96	1,229,900	0.2	21,249,831	3.54
SINE	51,914	0.01	0	0	0	0	453,547	0.08	446,000	0.07
Other*	12,169,475	2.02	0	0	7,698,698	1.28	50,424,873	8.4	78,096,062	13.02
Unknown*	161,876	0.03	0	0	0	0	50,424,873	16	84,103,655	14.02
Total	69,852,553	11.64	15,966,332	2.66	74,657,146	12.44	224,757,686	37.45	288,163,018	48.02

* Other represents sequences with annotation but not belonging to the above types of repetitive genes, such as satellites, simple repeats, retroposon, artifact, helitron, and low-complexity repeats; unknown represents sequences that cannot be classified. LINE: long interspersed nuclear element; LTR: long terminal repeat; SINE: short interspersed nuclear element.

### Gene prediction and functional annotation

The protein-coding genes in the *A. grahami* genome assembly were identified using *de novo*–based, homology-based, and RNA-seq–based gene prediction methods. Augustus v2.4 (Augustus, RRID:SCR_008417) [63], GlimmerHMM v3.0.4 (GlimmerHMM, RRID:SCR_002654) [64], Genemark (Genemark, RRID:SCR_011930) [65], and SNAP (SNAP, RRID:SCR_002127) [66], all trained for the *D. melanogaster* gene model before the gene prediction [67], were used in the *de novo*–based gene prediction with default parameters. GeMoMa (v1.3.1) was used to perform the annotation of protein coding based on the annotation of genes of *D. melanogaster*, *Glossina austeni*, *Lucilia cuprina*, *Stomoxys calcitrans*, and *Musca domestica* from GenBank (Table S5) [68]. The RNA-seq–based gene prediction was performed by PASA v2.0.2 (PASA, RRID:SCR_014656) [69]. Finally, the results from the 3 approaches were integrated using EVidenceModeler v1.1.1 (EVM, RRID:SCR_014659) [69]. When conducting the EVM integration, PASA-predicted transcripts from unigenes and GeMoMa-predicted homologous transcripts were given higher weights than the *de novo*–predicted transcripts. The gene set was aligned to the transposon database by TransposonPSI (v08222010) with default parameters [70]. Any gene of homology to transposons was removed from the final gene set. A total of 12,823 protein-coding genes were identified in the *A. grahami* genome, with an mean of 13,240.43 bp in length and 4.62 exons per gene (Table S6).

Gene functions of the predicted protein-coding genes were annotated using 2 strategies. First, those predicted protein sequences were aligned to Swiss-Prot and TrEMBL protein databases using Blastall with the best match parameters [71]. The pathways of the predicted gene sequences were extracted from the KEGG Automatic Annotation Server (v2.1) [72]. Then, the annotation of motifs and domains was achieved by searching the open databases including Pfam 32.0 (Pfam, RRID:SCR_004726), ProDom v2006.1 (ProDom, RRID:SCR_006969), PRINTS v42.0 (PRINTS, RRID:SCR_003412), PANTHER v12.0 (PANTHER, RRID:SCR_004869), SMRT (v7.1), and PROSITE v2018_02 (PROSITE, RRID:SCR_003457) with InterProScan v5.24 (InterProScan, RRID:SCR_005829) [73, 74]. The final dataset was obtained by combining the results of the above 2 parts. In summary, 12,791 genes were annotated with ≥1 related function, which accounted for 99.8% of predicted protein-coding genes (12,823) of *A. grahami* (Table 3). Additionally, the annotation of the non-coding RNA gene set was also performed on the basis of the RNA-seq data of *A. grahami* transcriptome data (6.6 Gb). The ribosomal RNA (rRNA), small nuclear RNA (snRNA), and microRNA were annotated using the non-coding database Rfam v14.0 (Rfam, RRID:SCR_007891). Then the transfer RNA (tRNA) sequence was annotated using tRNAscan-SE v2.0 (tRNAscan-SE, RRID:SCR_010835) [75]. The rRNA and subunits were predicted by RNAmmer (v1.2) [76]. As a result, a total of 126 microRNAs, 21 rRNAs, 192 snRNAs, and 859 tRNAs genes were annotated (Table S7).

**Table 3:** Function annotation of protein-coding genes of *A. grahami*

	Type	No. (%)
**Annotation**	Swiss-Prot	9,648 (75.2)
	TrEMBL	12,721 (99.2)
	KEGG	5,247 (40.9)
	KOG	8,252 (64.4)
	GO	7,518 (58.6)
	InterProScan	10,488 (81.8)
	Nr*	12,780 (99.7)
**Total**	Annotated	12,791 (99.8)
	Gene	12,823

* Nr: Non-Redundant Protein Sequence Database.

### Evolutionary analyses

#### Gene family and phylogenetic analyses

**Figure 2:**
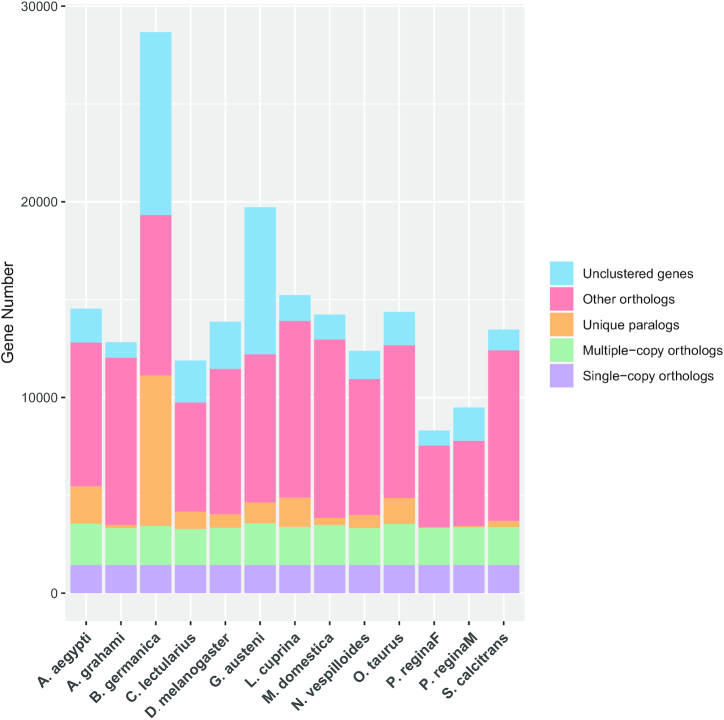
Gene family comparison between *A. grahami* and other insect species.

For the prediction of the gene family, several species were selected on the basis of genomic models, classification background, feeding habits, or lifestyles such as necrophagia, polyphagia, parasitism, or hematophagia. The genomic resources of *D. melanogaster, L. cuprina, M. domestica, S. calcitrans, G. austeni, P. regina, Onthophagus taurus, Nicrophorus vespilloides, Blattella germanica, Cimex lectularius*,and*Aedes aegypti* were used (Table S5) [67, 77–85]. OrthoMCL (OrthoMCL, RRID:SCR_007839) was used to identify the gene families [86]. First, the amino acid sequence of the longest transcript of each gene was selected from *A. grahami* and other selected insect species. Then they were aligned reciprocally with the BLASTP (BLASTP, RRID:SCR 0 01010) plug-in on NCBI with a threshold of e-value <1e−5. After that, the alignment results were clustered into family groups with default parameters. Finally, the orthologous gene families from each selected species were identified (Fig. 2). According to the results, the *A. grahami* genome contains the fewest unique genes and gene families compared to the other 11 species used in the analysis (Table 4).

**Table 4:** Genome families of *A. grahami* and other insect species

Species	Genes No.	Genes No. in families	Unclustered genes No.	Family No.	Unique families No.	Mean genes per family
*A. aegypti*	14,539	12,810	1,729	8,701	485	1.47
*A. grahami*	12,823	12,033	790	10,424	53	1.15
*B. germanica*	28,670	19,323	9,347	9,449	1,286	2.04
*C. lectularius*	11,890	9,743	2,147	8,104	250	1.2
*D. melanogaster*	13,872	11,469	2,403	9,694	235	1.18
*G. austeni*	19,722	12,205	7,517	9,867	350	1.24
*L. cuprina*	15,232	13,915	1,317	11,364	560	1.22
*M. domestica*	14,236	12,968	1,268	10,713	133	1.21
*N. vespilloides*	12,385	10,948	1,437	8,961	164	1.22
*O. taurus*	14,374	12,674	1,700	9,222	372	1.37
*P. regina* (F)	8,312	7,536	776	6,670	18	1.13
*P. regina* (M)	9,490	7,781	1,709	6,838	34	1.14
*S. calcitrans*	13,469	12,411	1,058	10,445	115	1.19

Unclustered genes and unique families represent the specific genes and families corresponding to each species.

In total, 2,989 single-copy gene families were identified among these 11 species. First, each gene family was aligned using the MAFFT program (v7) at the amino acid level [87]. All the sequence alignments were then reversely translated to nucleotide sequences. The poorly aligned positions and divergent regions were subsequently trimmed with Gblocks v0.91 (Gblocks, RRID:SCR_015945). Then, RAxML v8.2.11 (RAxML, RRID:SCR_006086) was used to construct phylogenetic trees using the GTR+GAMMA model for nucleotide sequences [88] with the branch reliability of RAxML assessed by 100 bootstrap replicates; *C. lectularius* was set as the outgroup.

**Figure 3:**
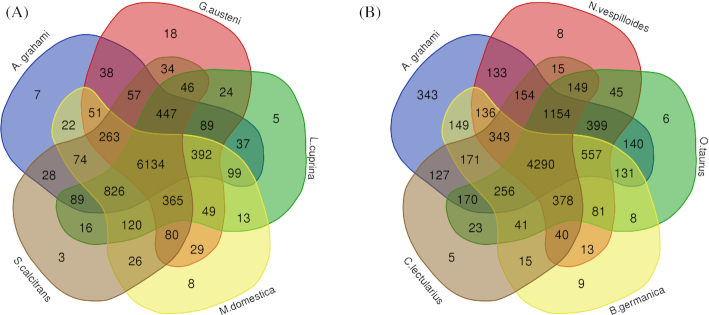
Venn diagram of orthologous gene families. (A) The intersection between *A. grahami* and other Diptera species. (B) The intersection between *A. grahami* and other non-Diptera species with different dietary habits.

In addition, 9 selected species were separated into different groups based on their dietary habits such as necrophagia, coprophagia, hematophagia, and polyphagia (Table S8). Orthologous genes of each species were also separated as a single assemblage. The shared orthologous genes of the clusters of *A. grahami* with other Diptera species and other non-Diptera species were displayed using the online Draw Venn Diagram [89]. The results may provide candidate genes for future research on the necrophagous lifestyle of *A. grahami* (Fig. 3).

#### Divergence time and gene family expansion/contraction

The estimation of divergence time was based on the results of the gene family clustering. Four-fold degenerate sites were extracted from the alignment of coding sequences of 2,989 identified single-copy gene families. The PAML MCMCTree program v4.5 (PAML, RRID:SCR_014932) was used to estimate divergence times with the calculation of the approximate likelihood test, molecular clock, and substitution model of REV [90]. The primary parameters of MCMCTree were set as clock = 2 (an independent rates model following a log-normal distribution), RootAge = <4 (400 million years ago for a calibration on the root of the phylogenetic tree), model = 7 (the substitution model, REV), BDparas = 110 (default value was used here, parameters controlling the birth-death process), kappa_gamma = 62 (transition/transversion rate ratio), alpha_gamma = 11 (γ shape parameter for variable rates among sites), rgene_gamma = 23.606 (Dirichlet γ prior for the mean substitution rate), sigma2_gamma = 11.03 (Dirichlet γ prior for the rate drift parameter). Calibrations of fossil evidence were retrieved from the TimeTree database to infer the evolutionary timescale [91].

**Figure 4:**
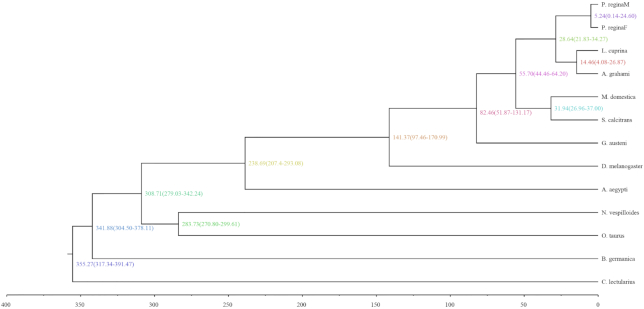
The estimation of divergence times. The numbers beside the dots of topological branches are the divergent time to the present day (million years ago). Numbers between branches represent the calibration time from fossil evidence. The right lists each family name. Numbers in parentheses are 95% confidence interval.

In the phylogenetic analysis, *A. grahami* and *L. cuprina* were clustered together at first. Then with *P. regina*, it was clustered into the branch of Calliphoridae, which is next to the family Muscidae represented by *M. domestica* and *S. calcitrans*. This result is consistent with the blowfly species taxonomy that *A. grahami* diverged with *L. cuprina* from the common ancestor ∼26 million years ago (Fig. 4).

To further explore the gene family change under natural selection, the expansion and contraction of gene families were identified using the CAFÉ program (CAFÉ, RRID:SCR_005983) [92]. The result revealed 102 expanded and 280 contracted gene families in the *A. grahami* genome. In addition, 198 gene families were lost from the genome (Table S9, Fig. S3).

#### Analysis of whole-genome duplication

We used 4-fold synonymous third-codon transversion (4DTv) [93] and Ks (a measure of synonymous substitution rate) estimation [94] to detect whole-genome duplication (WGD) events in the *A. grahami* genome. To this end, paralogous sequences of *A. grahami, Bombyx mori*, and *D. melanogaster* were identified with OrthoMCL [86]. Then, protein sequences of these insects were aligned against each other with BLASTP (using an e-value threshold of ≤1e−5) to identify conserved paralogs in each species. Finally, potential WGD events in each genome were evaluated based on their 4DTv and Ks distributions. The WGD analysis suggested that *A. grahami* may have experienced the same recent WGD events as *B. mori* (Fig. S4).

#### Chromosome assembly using Hi-C data

**Figure 5:**
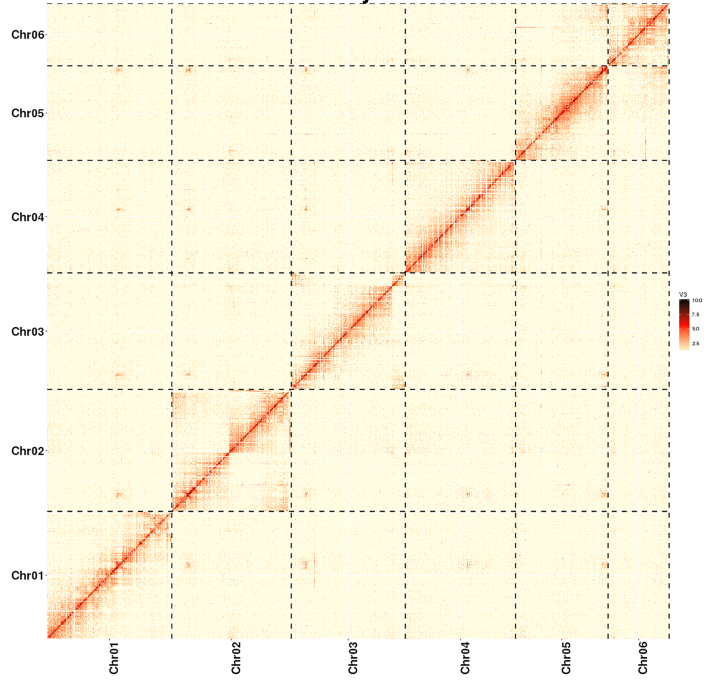
Hi-C interaction matrix maps within and among 6 chromosomes. The contact density is illustrated by the color bar from red (high density) to white (low density).

To generate a chromosome-level assembly of the genome, Hi-C fragment libraries were constructed. The Hi-C libraries were sequenced on the Illumina NovaSeq 6000 (Illumina, CA, USA), generating 495 million Hi-C paired-end reads. After low-quality sequences (quality score ≤15), adapter sequences, and sequences shorter than 30 bp were filtered out using fastp v0.12.6 (fastp, RRID:SCR_016962) [95], the clean paired-end reads were mapped to the draft assembled sequence by bowtie2 v.2.3.2 (bowtie2, RRID:SCR_005476) [96] to get the unique mapped paired-end reads. As a result, 102 million uniquely mapped paired-end reads were generated, of which 62.26% were valid interaction pairs (Table S10). Combined with the valid Hi-C data, we subsequently used the LACHESIS *de novo* assembly pipeline to produce chromosome-level scaffolds. As shown in Fig. 5, the assembled sequence was anchored onto the 6 pseudo-chromosomes with lengths ranging from 57.97 to 112.16 Mb (Table S11). The assembled pseudo-chromosomes (578,212,361 bp) accounted for 96.4% of the genome sequences (600,090,062 bp), with scaffold N50 values of 104.65 Mb (Table S3).

The similarity between the *A. grahami* genome and the published fruit fly (*D. melanogaster*) genome was analyzed [67]. The protein-coding genes from each genome were aligned using BLASTP with a threshold of e-value <1e−10. Then the results were combined with the GFF format files of the 2 genomes using MCScanX [97].

**Figure 6:**
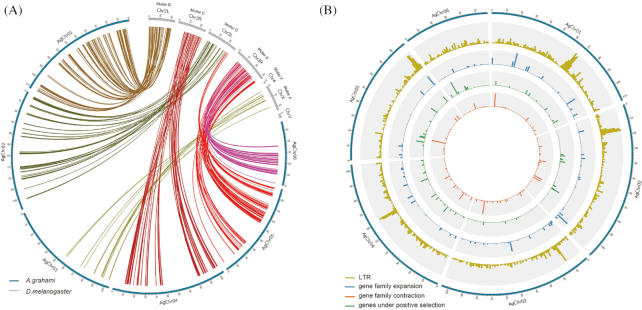
Collinearity and gene clustering of the *A. grahami* genome. (A) Collinear relationship between the *A. grahami* and *D. melanogaster* genomes. The blue bar represents the *A. grahami* genome and the grey one represents the fruit fly genome. (B) Gene density distribution on chromosomes of *A. grahami*. The outer blue circle indicates the chromosomes. The inner yellow, light blue, green, and orange circles represent the LTR, expanded gene families, contracted gene families, and positively selected genes, respectively. Window size = 1 Mb.

The collinearity between the *A. grahami* and *D. melanogaster* genomes is shown in Fig. 6A. The pseudo-chromosomes of *A. grahami* and the corresponding Muller elements of *D. melanogaster* are listed (Table S11). The Muller F was reported as the X-chromosome linked in some calyptratae species [98, 99]. In the present study, however, it is hard to tell from the results of collinearity analysis which assembled chromosome of *A. grahami* should be the Muller F. Further effort should be made to determine the sex chromosome of *A. grahami*.

In addition, we investigated the distributions of long terminal repeats (LTRs), gene family expansion or contraction, and genes under positive selection on the genome using a window size of 1 Mb across each chromosome and plotted the distributions in Fig. 6B by means of Circos (Circos, RRID:SCR_011798). There was no enrichment of genes for any particular chromosomes. All the chromosomes contain a gene density of ∼20 genes/Mb. However, the results showed that longer chromosomes tend to contain a higher number of LTRs, except for the case of Chr05. Besides, we noticed that the LTR content was enriched in the specific regions of each chromosome where it could represent the centromere locations (Table S11).

## Conclusions

In this study, we have successfully assembled the robust draft genome of *A. grahami* through long-read *de novo* technology and Hi-C sequencing technology using the PacBio Sequel sequencing platform. This reference genome is the first chromosome-level genome assembly in calyptratae, which will facilitate further genomic research of other fly species of forensic importance and promote the transition from forensic genetics to forensic genomics [48]. This draft genome resource will be beneficial to the advancement of study about the evolution of the *A. grahami* genome. It will deepen our understanding of the unique biological characteristics of *A. grahami*, such as low-temperature tolerance, seasonal distribution, necrophagous dietary habit, and its intrusion into other regions of the world. Based on qualified genome resources, studies of forensically important blowfly species will reinforce the reliability of entomological evidence and promote its application in legal criminal investigations [100].

## Availability of Supporting Data and Materials

Genome and transcriptome data of *A*. *grahami* are available in the NCBI SRA database (project accession: **PRJNA513084, SRA: SRX5207346**) and in the *GigaScience* Database, GigaDB [101]. Voucher sample information of the present work is listed in Table S12.

## Additional Files

**Additional File Figure S1**. 17-mer depth distribution curve. The x-axis represents the *k*-mer depth; the y-axis represents *k*-mer depth frequency; *Arabidopsis thaliana* (Atha) was set as reference.

**Additional File Figure S2**. Estimation of genome size of *A. grahami* by flow cytometry. Genome size (bp) was calculated from DNA content (pg) following the formula GAg = (FAg/FDm) × GDm. GAg: DNA content of *A. grahami*; GDm: DNA content of *D. melanogaster*; FAg: fluorescence value of *A. grahami*; FDm: fluorescence value of *D. melanogaster*.

**Additional File Figure S3**. Expansion and contraction at the gene family level. Branch length represents divergent time; pie chart illustrates the percentage of expansion and contraction; "+/-" means gene gain/loss.

**Additional File Figure S4**. Whole-genome duplication analysis of *A. grahami*, *B. mori*, and *D. melanogaster*.

**Additional File Table S1**. Information on sequencing platform and output data.

**Additional File Table S2**. Genome size estimation and heterozygosity based on 17 *k*-mer.

**Additional File Table S3**. Statistics results of genome assembly correction.

**Additional File Table S4**. Assessment of assembly completeness.

**Additional File Table S5**. Genome resource of 11 insect species for comparable genomics analysis.

**Additional File Table S6**. Comparison of *A. grahami* and other fly species on protein-coding gene structure and statistics.

**Additional File Table S7**. Functional annotation of non-coding RNA genes.

**Additional File Table S8**. Diet habit of 9 selected insect species.

**Additional File Table S9**. Statistics of gene family expansion and contraction

**Additional File Table S10**. Statistics of the Hi-C assembly of the *A. grahami* genome.

**Additional File Table S11**. Genome-wide characteristics of pseudochromosomes of *A. grahami*.

**Additional File Table S12**. Information on voucher samples used in the present study.

## Abbreviations

4DTv: 4-fold synonymous third-codon transversion; BLAST: Basic Local Alignment Search Tool; bp: base pairs; BUSCO: benchmarking universal single-copy orthologs; BWA: Burrows-Wheeler Aligner; CEGMA: Core Eukaryotic Genes Mapping Approach; *COI*: cytochrome oxidase gene I; CTAB: cetyltrimethyl ammonium bromide; Gb: gigabase pairs; GC: guanine-cytosine; GO: gene ontology; Hi-C: chromosome conformation capture; kb: kilobase pairs; KEGG: Kyoto Encyclopedia of Genes and Genomes; LINE: long interspersed nuclear element; LTR: long terminal repeat; MAFFT: Multiple Alignment using Fast Fourier Transform; Mb: megabase pairs; MCMC: Markov chain Monte Carlo; MISA: Microsatellite Identification Tool; NCBI: National Center for Biotechnology Information; PacBio: Pacific BioSciences; PAML: Phylogenetic Analysis by Maximum Likelihood; PASA: Program to Assemble Spliced Alignments; PMImin: minimum postmortem interval; p/n: part number; RAxML: Randomized Axelerated Maximum Likelihood; RNA-seq: RNA sequencing; rRNA: ribosomal RNA; SINE: short interspersed nuclear element; SMRT: single-molecule real time; SNAP: SNP Annotation and Proxy Search; snRNA: small nuclear RNA; SRA: Sequence Read Archive; SSR: simple sequence repeat; TE: transposable element; TRF: Tandem Repeats Finder; tRNA: transfer RNA; WGD: whole-genome duplication; WGS: whole-genome shotgun sequencing.

## Competing Interests

All authors declare that they have no competing interests.

## Funding

The present study was supported by a grant of the National Natural Science Foundation of China (81571855) and Science Foundation of Hunan Province (2017SK2015).

## Authors' Contributions

F.M. and J.C. designed the project. F.M., M.Z., Y.W., and C.C. analyzed the data. H.H., Z.L., and Y.J. prepared the samples and conducted the experiments. F.M., D.F., and Z.S. wrote and revised the manuscript. J.C. supervised the whole program and coordinated the group. Y.G. provided material and equipment for the breeding of insects.

## Supplementary Material

giaa020_GIGA-D-19-00066_Original_Submission

giaa020_GIGA-D-19-00066_Revision_1

giaa020_GIGA-D-19-00066_Revision_2

giaa020_GIGA-D-19-00066_Revision_3

giaa020_Response-to-Reviewer_Comments_Revision_1

giaa020_Response-to-Reviewer_Comments_Revision_2

giaa020_Response-to_Reviewer_Comments_Original_Submission

giaa020_Reviewer_1_Report_Original_Submission Clare Anstead -- 4/18/2019 Reviewed

giaa020_Reviewer_1_Report_Revision_1 Clare Anstead -- 8/20/2019 Reviewed

giaa020_Reviewer_2_Report_Original_Submission Aaron Tarone -- 4/19/2019 Reviewed

giaa020_Reviewer_2_Report_Revision_1 Aaron Tarone -- 8/20/2019 Reviewed

giaa020_Reviewer_2_Report_Revision_2 Aaron Tarone -- 11/11/2019 Reviewed

giaa020_Supplemental_Table
