# Supplementary material for: Chromosome-level genome assembly of Aldrichina grahami, a forensically important blowfly
Source: Gigascience. 2020 Mar 19;9(3):giaa020. doi: 10.1093/gigascience/giaa020 (PMC7081965; doi:10.1093/gigascience/giaa020)
Supplement: giaa020_GIGA-D-19-00066_Original_Submission [file giaa020_giga-d-19-00066_original_submission.pdf]

GigaScience

Chromosomal-level genome assembly of Aldrichina grahami, a forensically important blow fly

--Manuscript Draft--

**Manuscript Number:**	GIGA-D-19-00066
**Full Title:**	Chromosomal-level genome assembly of Aldrichina grahami, a forensically important blow fly
**Article Type:**	Data Note
**Funding Information:**	National Natural Science Foundation of China (81571855) — Pro. Jifeng Cai<br>Science Foundation of Hunan Province (2017SK2015) — Pro. Jifeng Cai
**Abstract:**	Background: Blow flies of Calliphoridae belong to the most frequently applied entomological evidence in forensic investigation. Aldrichina grahami is a forensically related blow fly species with some unique biological characteristics which distinguish it from the other blow fly species. Its development rate, pattern and life history can provides valuable information for estimation of the minimum postmortem interval (minPMI) and other forensic issues.<br>Findings: Here we provide a chromosomal-level genome assembly of A. grahami which was generated by Pacifc BioSciences sequencing platform and Hi-C technology. Totally 50.15 Gb clean reads of A. grahami genome was generated from Pacbio sequencing platform. Programs FALCON and WTDBG was utilized to construct the genome of A. grahami, and resulted in an assembly of 600 Mb with contig N50 of 1.93 Mb in length and 1604 in contigs number. A Hi-C sequencing and analysis generated a nearly chromosomal-level assembly within 6 chromosomes and scaffold N50 of 104.7 Mb. Nearly 96.4% of these scaffolds was anchored to A. grahami genome. We predicted 12823 protein-coding genes in A. grahami genome and 99.8% of it was functionally annotated based on data of de novo and transcriptome. Gene families clustering and phylogenetic reconstruction was performed based on co-analyses with other 10 insect species.<br>Conclusions: The present study provides a robust genome reference of A. grahami which add vital genetic information for nonhuman forensic genomics, and facilitate the development of research on A. grahami and other necrophagous blow fly species which can be useful for forensic cases.
**Corresponding Author:**	Jifeng Cai<br><br>CHINA
**Corresponding Author Secondary Information:**	
**Corresponding Author's Institution:**	
**Corresponding Author's Secondary Institution:**	
**First Author:**	Fanming Meng
**First Author Secondary Information:**	
**Order of Authors:**	Fanming Meng
	Jifeng Cai
	Zhuoying Liu
	Han Han
	Dmitrijs Finkelbergs
	Yangshuai Jiang

Powered by Editorial Manager® and ProduXion Manager® from Aries Systems Corporation

	Mingfei Zhu
	Chao Chen
	Yadong Guo
**Order of Authors Secondary Information:**	
**Additional Information:**	
**Question**	**Response**
Are you submitting this manuscript to a special series or article collection?	No
**Experimental design and statistics**<br><br>Full details of the experimental design and statistical methods used should be given in the Methods section, as detailed in our Minimum Standards Reporting Checklist. Information essential to interpreting the data presented should be made available in the figure legends.<br><br>Have you included all the information requested in your manuscript?	Yes
**Resources**<br><br>A description of all resources used, including antibodies, cell lines, animals and software tools, with enough information to allow them to be uniquely identified, should be included in the Methods section. Authors are strongly encouraged to cite Research Resource Identifiers (RRIDs) for antibodies, model organisms and tools, where possible.<br><br>Have you included the information requested as detailed in our Minimum Standards Reporting Checklist?	Yes
**Availability of data and materials**<br><br>All datasets and code on which the conclusions of the paper rely must be either included in your submission or deposited in publicly available repositories (where available and ethically	Yes

Powered by Editorial Manager® and ProduXion Manager® from Aries Systems Corporation

appropriate), referencing such data using a unique identifier in the references and in the “Availability of Data and Materials” section of your manuscript.<br><br>Have you have met the above requirement as detailed in our Minimum Standards Reporting Checklist?	

Powered by Editorial Manager® and ProduXion Manager® from Aries Systems Corporation

Manuscript

Click here to access/download;Manuscript;maintext 20180227.docx

Click here to view linked References

Chromosomal-level genome assembly of *Aldrichina grahami*, a forensically important blow fly

Fanming Meng[1], Zhuoying Liu[1], Han Han[1], Dmitrijs Finkelbergs[1], Yangshuai Jiang[1], Mingfei Zhu[2], Chao Chen[3], Yadong Guo[1], Jifeng Cai[1]*

[1] School of Basic Medicine, Central South University, Changsha, Hunan Pro, China

[2] Nextomics Biosciences, Wuhan, Hubei Pro, China

[3] Institute of Apicultural Research, Chinese Academy of Agricultural Sciences

* Corresponding authors

Emails:

F. M: mengfanming1984@163.com;

Z. L: 214872404@qq.com;

H. H: 583538543@qq.com;

D. F: dfinkelbergs@yahoo.com

Y. J: 1464804060@qq.com

M. Z: zhumingfei@grandomics.com

C. C: chenchaoiar@163.com

Y. G: gdy82@126.com

J. C: cjf_jifeng@163.com

Abstract

Background: Blow flies of Calliphoridae belong to the most frequently applied entomological evidence in forensic investigation. *Aldrichina grahami* is a forensically related blow fly species with some unique biological characteristics which distinguish it from the other blow fly species. Its development rate, pattern and life history can

provides valuable information for estimation of the minimum postmortem interval (minPMI) and other forensic issues.

Findings: Here we provide a chromosomal-level genome assembly of *A. grahami* which was generated by Pacifc BioSciences sequencing platform and Hi-C technology. Totally 50.15 Gb clean reads of *A. grahami* genome was generated from Pacbio sequencing platform. Programs FALCON and WTDBG was utilized to construct the genome of *A. grahami*, and resulted in an assembly of 600 Mb with contig N50 of 1.93 Mb in length and 1604 in contigs number. A Hi-C sequencing and analysis generated a nearly chromosomal-level assembly within 6 chromosomes and scaffold N50 of 104.7 Mb. Nearly 96.4% of these scaffolds was anchored to *A. grahami* genome. We predicted 12823 protein-coding genes in *A. grahami* genome and 99.8% of it was functionally annotated based on data of *de novo* and transcriptome. Gene families clustering and phylogenetic reconstruction was performed based on co-analyses with other 10 insect species.

Conclusions: The present study provides a robust genome reference of *A. grahami* which add vital genetic information for nonhuman forensic genomics, and facilitate the development of research on *A. grahami* and other necrophagous blow fly species which can be useful for forensic cases.

Keywords: *Aldrichina grahami*; blow fly; necrophgous; forensic entomology; minimum postmortem interval; genome assembly

Data Description

Background

Forensic entomology focuses on the application of knowledge gained from insects and other arthropods into lawsuit. Based on studies of development rate of

insect colonizers on corpse and insect succession pattern during corpse decaying process, forensic entomologist can estimate the minimum postmortem interval (minPMI) which represent the main tasks of forensic investigation [1, 2]. In addition, it can assist in the detection and recognition of wounds, estimation on the time length of neglect or abuse, as well as investigating the death reason [3-6]. The most important group of insects for forensic investigation is Diptera, especially those necrophagous fly species of Calliphoridae [7, 8]. Flies of this fauna, usually called blow fly, consist of many species living a parasite lifestyle or feeding on decaying corpses [9, 10]. The reliable life history and broadly distributing ability of necrophagous flies can provide vital information for forensic entomologist or investigators to infer a relative accurate minPMI [11, 12].

Figure 1. Female adult of *Aldrichina grahami* on corpse remain

Aldrichina grahami (Aldrich, 1930; NCBI:txid252811, homotypic synonym: *Calliphora grahami*) (Fig. 1) is a common blow fly species indigenous in east Asia [13, 14] and has expanded to American continent in the past several decades [15-17]. This blow fly usually breeds on carcasses or feces, and has the potential of contaminating human food [14]. *A. grahami* is wildly reported as a forensically important insect because of its necrophagous behavior, characteristics of seasonal distribution, and particularly unique characteristics of tolerance to low temperature than other necrophagous flies [18-20]. In early spring and late autumn, when the ambient temperature is relatively lower, *A. grahami* is frequently the first species to colonize on corpse. And it could be the only colonizer which complete the whole life history in some extreme cases [21, 22]. This information of seasonal distribution pattern of *A. grahami* can be applied as potential indicator of the season of death,

especially in the period of other insects inactive. Moreover, successful extraction of human DNA material from *A. grahami* larvae gut contents and age-dependently altering pattern of cuticular hydrocarbons indicates its great potential application value in forensic research and practice [23-25]. In addition to the forensic importance, cases of *A. grahami* caused myiasis from China have been reported routinely, especially after people traveled back from undeveloped regions [26-29]. And this blow fly species is also a potential transmitter of pathogens, like H5N1 influenza virus, which could cause serious public health problems in animal and human [30].

Knowledge about *A. grahami* has been slowly gained from basic research on insect biochemistry and physiology [11, 12, 31-35]. And the primary application of nuclear material is focused on distinguishing *A. grahami* from other sibling Diptera species [36-39]. Developmental patterns of *A. grahami* under different environment condition were also described by researchers [22, 40]. Nonetheless, the genome of *A. grahami* is still unavailable, which impedes the further applications in forensic research. Previous studies indicated the variation at genetic level among geographic populations of blow fly has ignorable influence on time length of development and life history of a fly species [38, 41]. And it is also recommended that the investigation on such forensic problems should be based on the existence of high-quality genome reference of one species [42, 43]. Here we provide a chromosome-scale scaffolding of assembly of this forensically important blow fly generated by Pacifc BioSciences (PacBio) sequencing platform and Hi-C method for future forensic research, as well as medical science.

Genome Sequencing and Assembly

Sample preparation

The samples of *A. grahami* was collected from field (Yuelu District, Changsha, Hunan, China), and has been bred for more than 20 generations at the school of basic medicine, Central South University. The new emerged female adults were used for DNA extraction.

After sample collection, tissues were immediately immerged into liquid nitrogen and stored at -80 ℃ until DNA extraction. DNA was extracted using the Cetyltrimethyl Ammonium Bromide (CTAB) method followed the introduction of Size-Selected 20 kb SMRTbell^{TM} Libraries for genomic DNA preparation. The quality of the extracted genomic DNA was checked using 0.7% agarose gel electrophoresis. Then Nanodrop spectrophotometer (Thermo Fisher Scientifc) was used to calculate the DNA purity. And the concentration of extracted material was quantified though Qubit fluorimeter (Invitrogen, Carlsbad, CA, USA).

Library construction and DNA sequencing

Two libraries were constructed before sequencing. Firstly a library of short insert length (400 bp) was constructed by Illumina TruSeq Nano DNA Library Prep Kits. Whole-genome shotgun sequencing (WGS) strategy was utilized for the short insert size library sequencing, which were performed on the Illumina HiSeq X Ten instrument at Genetron Health (Beijing, China). A total of 46.05 Gb of raw data was collected and subsequently filtered. Finally, 42.4 G clean data for short reads was generated (Table S1).

The long reads library of 20 kb was prepared using a SMRTbell DNA Template Prep Kit 1.0 (PacBio p/n 100-259-100). Genomic DNA material was mechanically sheared by using a Covaris G-TUBE^{TM} (Kbiosciences p/n 520079) to generate DNA fragments of approximately 20 kb. Polishing enzymes was used for sheared genomic

DNA damage- and end-repair. SMRTbell template was generated by blunt-end ligation reaction under exonuclease treatment and size selected by Blue Pippin device (Sage Science, Inc., Beverly, MA, USA) subsequently. A DNA 12000 Kit for Agilent Bioanalyzer 2100 (Agilent p/n 5067-1508) was applied to figure out the fragment size distribution.

A Sequel Binding Kit 2.0 (PacBio p/n 100-862-200) was used to bind prepared DNA template libraries to the Sequel Polymerase 2.0 in preparation for sequencing on the Sequel System, and finally formed a DNA polymerase/template complex according to the manufacturer's instructions. The enrichment of the larger fragments was improved by a MagBead (PacBio p/n 100-125-900) method. The long insert size (20 kb) library was sequenced on PacBio Sequel platform with Sequel SMRT cells 1M v2 (Pacifc Biosciencesp/n101–008-000) with one movie of 600 minutes per SMRT cell at the Genome Center of Nextomics (Wuhan, China). A total of 7 SMRT cells were processed and the raw data were filtered based on introduction of sequencing platform to remove low-quality bases or reads with adapters with the default parameters.In total, 50.15 Gb of long reads clean data were obtained (Table S1). The average length and the N50 of long subreads are 10.51 kb and 15.97 kb respectively.

Two Hi-C libraries were constructed for *A. grahami* and sequenced using the Illumina NovaSeq 6000 Sequencing System (San Diego, CA, USA) with PE150, which yield 74.24 Gb of raw data (Table S1).

Genome survey and Genome assembly

The genome size was estimated based on equation $G = k_{num} / k_{depth}$, where the k_{num} was the total number of 17-mers, k_{depth} denoted the peak frequency of 17-mers

estimated, and G represented the estimated genome size. Using Jellyfish (v2.1.3, RRID: SCR_005491) [44], 17-mers number was counted as 29,131,491,603 from short clean reads, and the k_{depth} was 50. Therefore, the genome size of *A. grahami* was estimated as 582.63 Mb according to the above equation and the heterozygosity rate of the *A. grahami* genome was approximately 2.5% (Table S2, Fig. S1).

PacBio long reads were corrected with Falcon (v0.4) and assembled with WTDBG (v1.2.8) [45, 46], the initial assembly was approximately 596.65 Mb in length, with contig N50 size of 1.93 Mb. For further improvement on accuracy of the reference assembly, following steps of polishing strategies were performed for the initial assembly. The pbalign (v.0.3.0) with default parameters was used for Quiver error-correction, and generated an error-corrected genome assembly of Pacbio long reads. We used BWA (v0.7.12, RRID: SCR 010910) to map short reads to this assembly. Then it was polished with Pilon (v1.21, RRID: SCR_014731) to generate the second iteration of the assembled genome [36]. Finally, we obtained a polished assembly genome in size of 600.09 Mb, with 1.93 Mb of contig N50 length and 1604 of contig number (Table 1, Table S3). So far, the present genome has the longest contig N50 length among all the published genome assembly of calyptratae flies of Diptera.

Table. 1 An overview comparison of Genome assembly and structure features in five calyptratae flies of Diptera.

Parameter	*A. grahami*	*L. cuprina*	*G. morsitans*	*M. domestica*	*Ph. regina* (♀)
Sequencing platform	PicBio	Illumina	454/Illumina	Illumina	454/PicBio
Genome Size (Mb)	600	458	366	692	550
No.of contigs/Scaffolds	1,604/7	74,043/4,436	-/13,807	-/20487	192,662/-
Contigs N50 (kb)	1930	744.4	50	12	7.9

GC level (%)	31	29.3	34.1	35.1	26.2
Repetitive regions (%)	48.02	57.8	-	55	8.11
Function annotation (gene number; %)	12,791; 99.8	12,160; 83.6	12,308; 99.5	14180;92.3	7792;94
Sequencing depth	86×	100×	160×	90×	44×
Completeness (BUSCO/CEGMA; %)	99.2	96	99	98	93.6

Genome completeness was assessed by BUSCO or CEGMA. Four calyptratae fly species genome was selected, as *L. cuprina* [47], *G. morsitans* [48], *M. domestica* [49] and *Ph. regina* [50]. The genome version of *Ph. regina* female adult was chosen.

For the *A. grahami* genome, the assembly genome size (600Mb) was almost same to the genome size (582.63 Mb) estimated in 17-mer analysis. The GC content and coverage of sequencing depth of the genome assembly was analyzed by the GC Depth analyses to check out the sequencing quality and potentially contaminated contigs from other species. The completeness of the assembly was evaluated by BUSCO (v3.0, RRID: SCR_015008). The result of BUSCO analysis proved that our assembly covered 99.2% complete and 0.7% partial insect BUSCOs, with only 0.5% missed (Table S4).

Chromosome assembly using Hi-C data

For generating a chromosomal level assembly of the genome, we took advantage of sequencing data from the Hi-C library. The Hi-C library was sequenced on the Illumina NanoSeq 6000 (Illumina, CA, USA) and generated 495 million Hi-C read pairs. After filtering out low-quality sequences (quality scores ≤ 15), adaptor sequences and sequences shorter than 30 bp by fastp (v0.12.6, RRID: SCR_016962) [51] and the clean paired-end reads were mapped to the draft assembled sequence

using bowtie2 (v.2.3.2, RRID: SCR_005476) [52] to get the unique mapped paired-end reads. As a result, 102 million uniquely mapped paired-end reads were generated, of which 62.26% were valid interaction pairs (Table S5). Combined with the valid Hi-C data, we subsequently employed the LACHESIS de novo assembly pipeline to produce chromosome-level scaffolds. As shown in Figure 2, the assembled sequence was anchored onto the 6 pseudo-chromosomes with lengths ranging from 57.97 Mb to 112.16 Mb (Table S6). The total length of pseudo-chromosomes accounted for 96.4% of the genome sequences, with scaffold N50 values of 104.65 Mb (Table S3).

Figure 2. Hi-C interaction matrix maps within and among 6 chromosomes. The contact density was illustrated by the color bar with red (high density) to white (low density).

A whole genome alignment between *A. grahami* genome and the published fruit fly (*D. melanogaster*) genome was performed to investigating a consistency between this two species using MCScanX [53] (Fig. 3, Table S7).

Figure 3. Collinear relationship between *A. grahami* and *D. melanogaster* genomes. The blue bar represent *A. grahami* genome and grey represent fruit fly genome.

Functional Prediction and Genome Annotation

Repeat genes analysis

Simple sequence repeats (SSRs) are a sort of short repeat sequences with 1-6 base pairs of DNA that wildly distribute in genomes. SSRs in the blow fly genome was identified using the MIcroSAtellite Identification Tool (MISA, RRID:SCR 010765) [54]. MISA can distinguish and locate both simple and compound microsatellites. The latter are always insert by a certain number of nucleic acid bases. In total, 322266 SSRs were found in the *A. grahami* genome.

Tandem repeats and transposable elements (TEs) of genome repetitive sequences were mined for *A. grahami*. A Tandem Repeats Finder (TRF, v4.09) approach was used to annotate the tandem repeats [55]. A combination of *de novo*-based and homology-based strategy was utilized at both the DNA and protein levels to identify TEs. Firstly we used RepeatModeler (v1.0.8, RRID: SCR 015027) [33] to construct a *de novo* repeat library of DNA level, from where a repeat consensus data set including classification information was constructed. The potential TEs assembly was searched in both the *de novo* based repeat library and a custom TE library (Repbase 23.08) using RepeatMasker (v4.0.6, RRID: SCR 012954) [56]. RepeatProteinMask which embedded in the RepeatMasker package was used to compare with the transposable element protein database with WU_BLASTX engine at protein level.

Overall, repetitive sequences accounted for 48.02% of *A. grahami* genome, and 43.69% of repetitive sequences were TEs. Repeat sequence of DNA consisted 11.65% of the *A. grahami* genome as the most abundant repeat class (Table 2).

Table 2 Statistics of repeat sequence analyses

**Type**	**RepeatMasker**		**LTR finder**		**RepeatProteinMask**		**RepeatModeler**		**Combined TEs**	
	Length (Mb)	% in genome	Length (Mb)	% in genome	Length (Mb)	% in genome	Length (Mb)	% in genome	Length (Mb)	% in genome
DNA	42174497	7.03	0	0	41341346	6.89	50464704	8.41	69933653	11.65
LINE	10505716	1.75	0	0	19838372	3.31	26169690	4.36	34333817	5.72
LTR	4789075	0.8	15966332	2.66	5778730	0.96	1229900	0.2	21249831	3.54

SINE	51914	0.01	0	0	0	0	453547	0.08	446000	0.07
Other*	12169475	2.02	0	0	7698698	1.28	50424873	8.4	78096062	13.02
Unknown*	161876	0.03	0	0	0	0	50424873	16	84103655	14.02
Total	69852553	11.64	15966332	2.66	74657146	12.44	224757686	37.45	288163018	48.02

***Other is sequences with annotation but no belong to above sorts of repetitive genes; unknown represents sequences cannot be classified.**

Gene prediction and functional annotation

A *de novo* based, homolog-based and RNAseq-based gene prediction methods were used in combination to identify protein-coding genes in the *A. grahami* genome assembly. Augustus (v2.4, RRID: SCR 008417) [57], GlimmerHMM (v3.0.4, RRID: SCR 002654) [58], Genemark (RRID: SCR_011930) and SNAP (RRID: SCR_002127) [59] were used for the *de novo*-based gene prediction with default parameters. And all of these software packages were trained using the *D. melanogaster* gene model before gene prediction. GeMoMa (v1.3.1) was used to annotate genes using homolog references of *D. melanogaster*, *Glossina austeni*, *Lucilia cuprina*, *Stomoxys calcitrans* and *Musca domestica* from GenBank [60]. PASA (v2.0.2, RRID: SCR 014656) were used for the RNAseq-based method of gene prediction [6]. Finally, the results from the three approaches were integrated using EVidenceModeler (EVM) (v1.1.1, RRID: SCR 014659) [6]. When conducting the EVM integration, PASA-predicted transcripts from unigenes and GeMoMa-predicted homologous transcripts were given higher weights than the *de novo* predicted transcripts. The gene set was aligned to the transposon database by TransposonPSI (v08222010) with default parameters to remove genes containing transposon [6]. Genes of transposon homologys were removed from the finial gene assemblage. In total, 12823 protein-coding genes were identified in *A. grahami* genome, with an average of 13240.43 bp in length, 4.62

exons per gene (Table S8).

Gene functions of predicted protein coding gene were annotated using two strategies. First, those predicted protein sequences were aligned to SwissProt and TrEMBL protein databases [4] using Blastall under the best match parameter. The gene pathway of predicted sequences was extracted from the KEGG Automatic Annotation Server (v2.1) [5]. Then the annotation of motifs and domains were performed using InterProScan (v5.24, RRID: SCR 005829) to search against opening databases of InterPro including member database Pfam (32.0, RRID:SCR 004726), ProDom (v2006.1, RRID:SCR 006969), PRINTS (v42.0, RRID:SCR 003412), PANTHER (v12.0, RRID:SCR 004869), SMRT (v7.1), and PROSITE (2018_02, RRID: SCR 003457) [6, 61]. These two parts of results were combined together to form the final data set. In summary, 12791 genes were annotated with at least 1 related function, which successfully accounted for about 99.8% of the predicated protein coding genes (12823) of *A. grahami* (Table 3). Additionally, an annotation of non-coding RNA genes set was also performed based on the RNA-seq data of *A. grahami* transcriptome data (6.6G). The data set was aligned to the non-coding database Rfam (v14.0, RRID: SCR 007891) to annotate genes of rRNA, snRNA and miRNA firstly. Then tRNA sequence was predicted using tRNAscan-SE (v2.0, RRID: SCR_010835) [62]. The rRNA and subunits was predicted by RNAmmer(v1.2) [63]. Finally, a total of 126 miRNAs, 21 rRNAs, 192 snRNAs and 859 tRNAs genes was annotated (Table S9).

Table 3 Function annotation of protein coding genes of *A. grahami*

	Type	Number	Percent (%)
**Annotation**	Swissprot	9648	75.2
	Trembl	12721	99.2
	Kegg	5247	40.9

	KOG	8252	64.4
	GO	7518	58.6
	InterProscan	10488	81.8
	nr	12780	99.7
**Total**	Annotated	12791	99.8
	Gene	12823	-

Evolutionary analyses

Gene family and Phylogenetic analyses

For gene family prediction among insect species, amino acid sequence of the longest transcript of each gene was selected from the *A. grahami*, and other insect species was used as references. The species selected for this step was based on genomic models, classification background, feeding habits or life style like necrophagous, polyphagia, parasite or blood feeding. The genomic resource of *D. melanogaster, Lucilia cuprina, Musca domestica, Stomoxys calcitrans, Glossina austeni, Onthophagus taurus, Nicrophorus vespilloides, Blattella germanica, Cimex lectularius, Aedes aegypti* were aligned reciprocally with the BLASTP (RRID: SCR 001010) plug-in on NCBI with an e-value threshold of 1e-5 [64]. The OrthoMCL (RRID: SCR 007839) was used to group orthologous protein sequences of the *A. grahami* proteome against other insect proteomes which were selected in this step. Finally, the orthologous gene families from each selected species were identified (Fig. 4). According to the statistics results of this step, *A. grahami* genome contents obviously the fewest number of unique genes and gene families comparing with other 10 species used in analysis (Table 4).

Figure 4. Gene family comparison between *A. grahami* and other insect species

In total, 2989 single-copy gene families shared within these 11 species were extracted. Each gene family was first aligned at amino-acid level using the MAFFT program (v7) [43]. All the sequence alignments were then reverse translated to nucleotide alignments, and regions with low sequencing quality were filtered out by Gblocks (v0.91, RRID: SCR 015945) subsequently. Then, RAxML (v8.2.11, RRID: SCR 006086) [52] was applied to build phylogenetic trees under a GTR+GAMMA model for nucleotide sequences. A total of 100 bootstrap replicates were employed to assess branch reliability in RAxML. *C. lectularius* was set as outgroup.

Table 4 Genome families of *A. grahami* and other insect species

Species	Genes Number	Genes number in families	Unclustered genes number	Family number	Unique Families number	Average genes number per family
A.aegypti	14,539	12,790	1,749	8,703	484	1.47
A.grahami	12,823	11,985	838	10,413	56	1.15
B.germanica	28,670	19,219	9,451	9,424	1,286	2.04
C.lectularius	11,890	9,718	2,172	8,104	250	1.2
D.melanogaster	13,872	11,442	2,430	9,707	237	1.18
G.austeni	19,722	12,012	7,710	9,794	355	1.23
L.cuprina	15,232	13,872	1,360	11,339	586	1.22
M.domestica	14,236	12,914	1,322	10,718	141	1.2
N.vespilloides	12,385	10,933	1,452	8,971	168	1.22
O.taurus	14,374	12,656	1,718	9,227	371	1.37
S. calcitrans	13,469	12,371	1,098	10,473	117	1.18

Unclustered genes and unique families represent the specific genes and families belong to each species.

In addition, we separated the 9 selected species into different group based on their diet habit like necrophagous, coprophagous, blood feeding and polyphagous (Table S10). And intersection of orthologous genes of *A. grahami* with those Diptera species and other non Diptera species were calculated respectively, which could provide candidate genes of research on necrophagous life style of *A. grahami* (Fig. 5).

Figure 5. Venn diagram of orthologous gene families. (A) Intersection among *A. grahami* and Diptera species. (B) Intersection among *A. grahami* and other non Diptera species of different diet habit.

Divergence time and gene family expansion / contraction

The estimation of divergence time was based on the results of gene family clustering. Four-fold degenerate sites were extracted from the alignment of coding sequences of 2989 identified single copy gene family. The PAML mcmctree program (v4.5, RRID: SCR 014932) was used to determine divergence times under the calculation of approximate likelihood test, molecular clock and substitution model of REV [65]. The major parameters of mcmctree was set as clock = 2， RootAge = < 4，model = 7, BDparas = 110， kappa_gamma = 62， alpha_gamma = 11，rgene_gamma = 23.606， sigma2_gamma = 11.03. Calibrations of fossil evidence were retrieved from the TimeTree database to infer the evolutionary timescale [66].

According to the phylogenetic analysis, *A. grahami* were clustered together with *L. cuprina*, and these two species clustered into branch of Calliphoridae which next to Muscidae represented by *M. domestica*, *S. calcitrans*. This result was consistent with the blow fly species taxonomy. *A. grahami* diverged from the common ancestor with *L. cuprina* around 26 million years ago (Fig. 6).

Figure 6. The estimation on divergence times. The numbers besides the notes of topological branches is the divergent time to present day (Million years ago, Mya). Red notes represents the calibration time from fossil evidences. The right lists each family name.

Based on the results of gene family prediction and phylogenetic development, the gene family expanding and contracting analyses was performed using CAFE

program (RRID:SCR_005983) [67], to further exploring the gene family change under natural selecting pressure. In summary, expansion and contraction were happened in 102 and 280 gene families respectively in *A. grahami* genome. Additionally 198 gene families were extinct from the genome (Table S11, Fig. S2).

Whole-genome duplication (WGD) and analysis

We used four-fold synonymous third-codon transversion (4DTv) estimation [68] and Ks (a measure of the substitutions per synonymous site) distribution [69] to detect WGD events in *A. grahami* genome. To this end, paralogous sequences of *A. grahami*, *Bombyx mori* and *D. melanogaster*, was identified with OrthoMCL [70]. Then, protein sequences for each of these insects were aligned against each other with Blastp (using an E-value threshold of ≤ 1e-5) to identify conserved paralogs in each species. Finally, potential WGD events in each genome were evaluated based on their 4DTv and Ks distribution. The WGD analysis suggested that *A. grahami* experienced the same recent WGD events as *B. mori.* (Fig. S3).

Conclusion

Using long reads *de novo* technology of PacBio Sequel sequencing platform and Hi-C sequencing technology, we successfully assembled the robust draft genome of *A. grahami*. This reference genome represents the first chromosome-level genome assembly in calyptratae. It will facilitate further genomic research of other fly species of forensically importance which is definitely meaningful to promote the transition from forensic genetics to forensic genomics [71]. This draft genome recourse will be an invaluable tool for advancing knowledge of biogeographics, genetics, and the evolution of *A. grahami* at genome level. It will help in understanding the genetic

mechanisms determining *A. grahami*'s unique biological characteristics like low temperature tolerance, seasonal distribution, necrophagous diet habit, and how this Asia indigenous species intrude into other regions of the world. Forensic entomological studies based on qualified genome resource will definitely consolidate the reliability of entomological evidence and promote its application in law suit [72]. Additionally, these data will also facilitate the development of repellents for flystrike.

Availability of supporting data

Genome data is available in the NCBI SRA database (project accession: **PRJNA513084**).

Additional flies

Additional File Figure S1. 17-mer Depth Distribution Curve. The x-axis represents the k-mer depth; the y-axis represent k-mer depth frequency; *Arabidopsis thaliana* (Atha for short) was set as reference.

Additional File Figure S2. Expansion and contraction at the gene family level. Brach length represents divergent time; Pie chart illustrates percentage of expansion and contraction; '+/-'means gene gain / loss.

Additional File Figure S3. Whole-genome duplication analysis of *A. grahami*, *B. mori* and *D. melanogaster*.

Additional File Table S1. Information of sequencing platform and output data.

Additional File Table S2. Genome size estimation and Heterozygosity based on 17 k-mer.

Additional File Table S3. Statistics results of genome assembly correction.

Additional File Table S4. Assessment on assembly completeness.

Additional File Table S5. Statistics of the Hi-C assembly of the *A. grahami* genome.

Additional File Table S6. Total length of contigs clustered on each chromosome.

Additional File Table S7. Genome resource of 10 insect species for comparable genomics analysis.

Additional File Table S8. Comparison of *A grahami* and other fly species on protein coding genes structure and statistics.

Additional File Table S9. Functional annotation of non-coding RNA genes.

Additional File Table S10. Diet habit of 9 selected insect species.

Additional File Table S11. Statistics of gene family expansion and contraction

Abbreviations

PMImin: minimum postmortem interval; CTAB: Cetyltrimethyl Ammonium Bromide; WGS: Whole-genome shotgun sequencing; SSR: Simple sequence repeats; TEs: Transposable elements; TRF: Tandem Repeats Finder; Mya: Million years ago; 4DTv: four-fold synonymous third-codon transversion; BUSCO: benchmarking universal single-copy orthologs; GO: gene ontology; KEGG: Kyoto Encyclopedia of Genes and Genomes; SMRT: single-molecule real time; Whole-genome duplication: WGD

Competing interests

All authors declare that no competing interests.

Funding

The present study was supported by grant of the National Natural Science

Foundation of China (81571855) and Science Foundation of Hunan Province (2017SK2015).

Author contributions

F. M., J.C. designed the project. F. M., M. Z., and C.C. analyzed the data. H.H., Z. L., Y. J prepared the samples and conducted the experiments. F. M., D.F., wrote and revised the manuscript. J.C. supervised the whole program and coordinated the group. Y. G. provided material and equipment for breeding of insects.

Reference

1. Catts EP and Goff ML. Forensic entomology in criminal investigations. Annu Rev Entomol. 1992;37:253-72. doi:10.1146/annurev.en.37.010192.001345.
2. Benecke M. A brief history of forensic entomology. Forensic Sci Int. 2001;120 1-2:2-14.
3. Tomberlin JK, Mohr R, Benbow ME, Tarone AM and VanLaerhoven S. A roadmap for bridging basic and applied research in forensic entomology. Annu Rev Entomol. 2011;56:401-21. doi:10.1146/annurev-ento-051710-103143.
4. Benecke M and Lessig R. Child neglect and forensic entomology. Forensic Sci Int. 2001;120 1-2:155-9.
5. Campobasso CP, Gherardi M, Caligara M, Sironi L and Introna F. Drug analysis in blowfly larvae and in human tissues: a comparative study. Int J Legal Med. 2004;118 4:210-4. doi:10.1007/s00414-004-0448-1.
6. JLC. JHB. Insects of Forensic Importance. In: JLC. JHB, editor. Forensic entomology : the utility of arthropods in legal investigations. Boca Raton, London: CRC Press; 2009. p. 44-6.
7. Zhu BF, Zhang YD, Shen CM, Du WA, Liu WJ, Meng HT, et al. Developmental validation of the AGCU 21+1 STR kit: a novel multiplex assay for forensic application. Electrophoresis. 2015;36 2:271-6. doi:10.1002/elps.201400333.
8. Wang HD, Ruan JG, Shen CM, Zhang YD, Liu WJ, Meng HT, et al. Polymorphic distribution and forensic effectiveness study of eight miniSTR in Chinese Uyghur ethnic group. Mol Biol Rep. 2014;41 4:2371-5. doi:10.1007/s11033-014-3091-z.
9. Du XD and Meng HQ. [Analysis of 324 cases of forensic psychiatry expert testimony in Chongqing]. Fa Yi Xue Za Zhi. 2009;25 5:362-4.
10. Rodrigues RA, de Azeredo-Espin AM and Torres TT. Microsatellite markers for population genetic studies of the blowfly Chrysomya putoria (Diptera: Calliphoridae). Mem Inst Oswaldo Cruz. 2009;104 7:1047-50.
11. Zajac BK, Amendt J, Horres R, Verhoff MA and Zehner R. De novo transcriptome analysis and highly sensitive digital gene expression profiling of Calliphora vicina (Diptera: Calliphoridae)

pupae using MACE (Massive Analysis of cDNA Ends). Forensic Sci Int Genet. 2015;15:137-46. doi:10.1016/j.fsigen.2014.11.013.

12. Zehner R AJ, Boehme P Gene expression analysis as a tool for age estimation of blowfly pupae. Forensic Science International Genetics Supplement. 2009;2 1:292-3.

13. Matuszewski S, Fratczak K, Konwerski S, Bajerlein D, Szpila K, Jarmusz M, et al. Effect of body mass and clothing on carrion entomofauna. Int J Legal Med. 2016;130 1:221-32. doi:10.1007/s00414-015-1145-y.

14. Fan DZ. Key to the common flies of China. Beijing, China: Science publishing house; 1992.

15. Adams MD, Celniker SE, Holt RA, Evans CA, Gocayne JD, Amanatides PG, et al. The genome sequence of Drosophila melanogaster. Science. 2000;287 5461:2185-95.

16. Smith BR and Unckless RL. Correction for Smith and Unckless, "Draft Genome Sequence of Lysinibacillus fusiformis Strain Juneja, a Laboratory-Derived Pathogen of Drosophila melanogaster". Genome Announc. 2018;6 21 doi:10.1128/genomeA.00475-18.

17. Smith BR and Unckless RL. Draft Genome Sequence of Lysinibacillus fusiformis Strain Juneja, a Laboratory-Derived Pathogen of Drosophila melanogaster. Genome Announc. 2018;6 5 doi:10.1128/genomeA.01571-17.

18. Norris KR. The Bionomics of Blow Flies. Annreventomol. 1965;10 1:47-68.

19. Luo B, Ji MM, Meng HH, Chen XP and Tao LY. [Forensic Application of Objective Assessment on Visual Acuity by ERP]. Fa Yi Xue Za Zhi. 2017;33 3:232-5. doi:10.3969/j.issn.1004-5619.2017.03.003.

20. Jin X, Wei Y, Chen J, Kong T, Mu Y, Guo Y, et al. Phylogenic analysis and forensic genetic characterization of Chinese Uyghur group via autosomal multi STR markers. Oncotarget. 2017;8 43:73837-45. doi:10.18632/oncotarget.17992.

21. Guo YD, Cai JF, Tang ZC, Feng XO, Lin Z, Yong F, et al. Application of Aldrichina grahami (Diptera, Calliphoridae) for forensic investigation in central-south China. Romanian Journal Of Legal Medicine. 2011;19 1:55-8. doi:10.4323/rjlm.2011.55.

22. Guntner AT, Pineau NJ, Mochalski P, Wiesenhofer H, Agapiou A, Mayhew CA, et al. Sniffing Entrapped Humans with Sensor Arrays. Anal Chem. 2018;90 8:4940-5. doi:10.1021/acs.analchem.8b00237.

23. Ramos-Onsins S and Aguade M. Molecular evolution of the Cecropin multigene family in Drosophila. functional genes vs. pseudogenes. Genetics. 1998;150 1:157-71.

24. Harden N, Loh HY, Chia W and Lim L. A dominant inhibitory version of the small GTP-binding protein Rac disrupts cytoskeletal structures and inhibits developmental cell shape changes in Drosophila. Development. 1995;121 3:903-14.

25. Lee PS, Sing KW and Wilson JJ. Reading Mammal Diversity from Flies: The Persistence Period of Amplifiable Mammal mtDNA in Blowfly Guts (Chrysomya megacephala) and a New DNA Mini-Barcode Target. PLoS One. 2015;10 4:e0123871. doi:10.1371/journal.pone.0123871.

26. Wan KH, Yu C, Park S, Hammonds AS, Booth BW and Celniker SE. Complete Genome Sequence of Acetobacter pomorum Oregon-R-modENCODE Strain BDGP5, an Acetic Acid Bacterium Found in the Drosophila melanogaster Gut. Genome Announc. 2017;5 48 doi:10.1128/genomeA.01333-17.

27. Wan KH, Yu C, Park S, Hammonds AS, Booth BW and Celniker SE. Complete Genome Sequence of Acetobacter tropicalis Oregon-R-modENCODE Strain BDGP1, an Acetic Acid Bacterium Found in the Drosophila melanogaster Gut. Genome Announc. 2017;5 46

doi:10.1128/genomeA.01020-17.

28. Wan KH, Yu C, Park S, Hammonds AS, Booth BW and Celniker SE. Complete Genome Sequence of Lactobacillus plantarum Oregon-R-modENCODE Strain BDGP2 Isolated from Drosophila melanogaster Gut. Genome Announc. 2017;5 41 doi:10.1128/genomeA.01155-17.

29. Lachish T, Marhoom E, Mumcuoglu KY, Tandlich M and Schwartz E. Myiasis in Travelers. J Travel Med. 2015;22 4:232-6. doi:10.1111/jtm.12203.

30. Shen C, Wang H, Feng Z, Dong Q, Guo Y, Wang X, et al. Forensic effectiveness and population differentiations study of AGCU 21+1 fluorescence multiplex in Chinese Henan Han population. Forensic Sci Int Genet. 2017;28:e18-e21. doi:10.1016/j.fsigen.2017.01.013.

31. GilArriortua M, Salona Bordas MI, Kohnemann S, Pfeiffer H and de Pancorbo MM. Molecular differentiation of Central European blowfly species (Diptera, Calliphoridae) using mitochondrial and nuclear genetic markers. Forensic Sci Int. 2014;242:274-82. doi:10.1016/j.forsciint.2014.07.018.

32. Tarone AM, Jennings KC and Foran DR. Aging blow fly eggs using gene expression: A feasibility study. Journal Of Forensic Sciences. 2007;52 6:1350-4. doi:10.1111/j.1556-4029.2007.00587.x.

33. Sze SH, Dunham JP, Carey B, Chang PL, Li F, Edman RM, et al. A de novo transcriptome assembly of Lucilia sericata (Diptera: Calliphoridae) with predicted alternative splices, single nucleotide polymorphisms and transcript expression estimates. Insect Mol Biol. 2012;21 2:205-21. doi:10.1111/j.1365-2583.2011.01127.x.

34. Cristoni S, Dusi G, Brambilla P, Albini A, Conti M, Brambilla M, et al. SANIST: optimization of a technology for compound identification based on the European Union directive with applications in forensic, pharmaceutical and food analyses. J Mass Spectrom. 2017;52 1:16-21. doi:10.1002/jms.3895.

35. Yang Z. PAML: a program package for phylogenetic analysis by maximum likelihood. Comput Appl Biosci. 1997;13 5:555-6.

36. Walker BJ, Abeel T, Shea T, Priest M, Abouelliel A, Sakthikumar S, et al. Pilon: an integrated tool for comprehensive microbial variant detection and genome assembly improvement. PLoS One. 2014;9 11:e112963. doi:10.1371/journal.pone.0112963.

37. Hall MJ, Wall RL and Stevens JR. Traumatic Myiasis: A Neglected Disease in a Changing World. Annu Rev Entomol. 2016;61:159-76. doi:10.1146/annurev-ento-010715-023655.

38. Picard CJ and Wells JD. Survey of the genetic diversity of Phormia regina (Diptera: Calliphoridae) using amplified fragment length polymorphisms. J Med Entomol. 2009;46 3:664-70.

39. Meng F, Ren L, Wang Z, Deng J, Guo Y, Chen C, et al. Identification of Forensically Important Blow Flies (Diptera: Calliphoridae) in China Based on COI. J Med Entomol. 2017;54 5:1193-200. doi:10.1093/jme/tjx105.

40. Lambiase S, Murgia G, Sacchi R, Ghitti M and di Lucia V. Effects of Different Temperatures on the Development of Dermestes Frischii and Dermestes Undulatus (Coleoptera, Dermestidae): Comparison Between Species. J Forensic Sci. 2018;63 2:469-73. doi:10.1111/1556-4029.13580.

41. Byrne AL, Camann MA, Cyr TL, Catts EP and Espelie KE. Forensic implications of biochemical differences among geographic populations of the black blow fly, Phormia regina (Meigen). J Forensic Sci. 1995;40 3:372-7.

42. Autenrieth M, Hartmann S, Lah L, Roos A, Dennis AB and Tiedemann R. High-quality whole-genome sequence of an abundant Holarctic odontocete, the harbour porpoise (Phocoena phocoena). Mol Ecol Resour. 2018;18 6:1469-81. doi:10.1111/1755-0998.12932.
43. Arenas M, Pereira F, Oliveira M, Pinto N, Lopes AM, Gomes V, et al. Forensic genetics and genomics: Much more than just a human affair. PLoS Genet. 2017;13 9:e1006960. doi:10.1371/journal.pgen.1006960.
44. Gallagher MB, Sandhu S and Kimsey R. Variation in developmental time for geographically distinct populations of the common green bottle fly, Lucilia sericata (Meigen). J Forensic Sci. 2010;55 2:438-42. doi:10.1111/j.1556-4029.2009.01285.x.
45. WTDBG package：https://github.com/ruanjue/wtdbg. (Accessed 10 Jan 2018).
46. Hoffman EM, Curran AM, Dulgerian N, Stockham RA and Eckenrode BA. Characterization of the volatile organic compounds present in the headspace of decomposing human remains. Forensic Sci Int. 2009;186 1-3:6-13. doi:10.1016/j.forsciint.2008.12.022.
47. Anstead CA, Korhonen PK, Young ND, Hall RS, Jex AR, Murali SC, et al. Lucilia cuprina genome unlocks parasitic fly biology to underpin future interventions. Nat Commun. 2015;6:7344. doi:10.1038/ncomms8344.
48. International Glossina Genome I. Genome sequence of the tsetse fly (Glossina morsitans): vector of African trypanosomiasis. Science. 2014;344 6182:380-6. doi:10.1126/science.1249656.
49. Scott JG, Warren WC, Beukeboom LW, Bopp D, Clark AG, Giers SD, et al. Genome of the house fly, Musca domestica L., a global vector of diseases with adaptations to a septic environment. Genome Biol. 2014;15 10:466. doi:10.1186/s13059-014-0466-3.
50. Andere AA, Platt RN, 2nd, Ray DA and Picard CJ. Genome sequence of Phormia regina Meigen (Diptera: Calliphoridae): implications for medical, veterinary and forensic research. BMC Genomics. 2016;17 1:842. doi:10.1186/s12864-016-3187-z.
51. Chen S, Zhou Y, Chen Y and Gu J. fastp: an ultra-fast all-in-one FASTQ preprocessor. Bioinformatics. 2018;34 17:i884-i90. doi:10.1093/bioinformatics/bty560.
52. Langmead B and Salzberg SL. Fast gapped-read alignment with Bowtie 2. Nat Methods. 2012;9 4:357-9. doi:10.1038/nmeth.1923.
53. Wang Y, Tang H, Debarry JD, Tan X, Li J, Wang X, et al. MCScanX: a toolkit for detection and evolutionary analysis of gene synteny and collinearity. Nucleic Acids Res. 2012;40 7:e49. doi:10.1093/nar/gkr1293.
54. Wan KH, Yu C, Park S, Hammonds AS, Booth BW and Celniker SE. Complete Genome Sequence of Bacillus kochii Oregon-R-modENCODE Strain BDGP4, Isolated from Drosophila melanogaster Gut. Genome Announc. 2017;5 40 doi:10.1128/genomeA.01074-17.
55. Fresia P, Lyra ML, Coronado A and De Azeredo-Espin AM. Genetic structure and demographic history of new world screwworm across its current geographic range. J Med Entomol. 2011;48 2:280-90.
56. Wan KH, Yu C, Park S, Hammonds AS, Booth BW and Celniker SE. Complete Genome Sequence of Enterococcus durans Oregon-R-modENCODE Strain BDGP3, a Lactic Acid Bacterium Found in the Drosophila melanogaster Gut. Genome Announc. 2017;5 40 doi:10.1128/genomeA.01041-17.
57. Paredes JC, Herren JK, Schupfer F, Marin R, Claverol S, Kuo CH, et al. Genome sequence of the Drosophila melanogaster male-killing Spiroplasma strain MSRO endosymbiont. MBio. 2015;6

2 doi:10.1128/mBio.02437-14.

58. Hoskins RA, Carlson JW, Wan KH, Park S, Mendez I, Galle SE, et al. The Release 6 reference sequence of the Drosophila melanogaster genome. Genome Res. 2015;25 3:445-58. doi:10.1101/gr.185579.114.

59. Guerra JC and Licinio P. A method for extracting period~10 modulations from DNA sequence correlations applied to the Drosophila melanogaster genome. Genet Mol Res. 2012;11 3:2835-46. doi:10.4238/2012.August.24.8.

60. Kucerova L, Broz V, Arefin B, Maaroufi HO, Hurychova J, Strnad H, et al. The Drosophila Chitinase-Like Protein IDGF3 Is Involved in Protection against Nematodes and in Wound Healing. J Innate Immun. 2016;8 2:199-210. doi:10.1159/000442351.

61. Tarone AM, Picard CJ, Spiegelman C and Foran DR. Population and temperature effects on Lucilia sericata (Diptera: Calliphoridae) body size and minimum development time. J Med Entomol. 2011;48 5:1062-8.

62. Kim J and Gibson G. Insights from GWAS into the quantitative genetics of transcription in humans. Genet Res (Camb). 2010;92 5-6:361-9. doi:10.1017/S001667231000056X.

63. Gibson G and Weir B. The quantitative genetics of transcription. Trends Genet. 2005;21 11:616-23. doi:10.1016/j.tig.2005.08.010.

64. Mount DW. Using the Basic Local Alignment Search Tool (BLAST). CSH Protoc. 2007;2007:pdb top17. doi:10.1101/pdb.top17.

65. Yang ZH. PAML 4: Phylogenetic analysis by maximum likelihood. Molecular Biology And Evolution. 2007;24 8:1586-91. doi:10.1093/molbev/msm088.

66. Hedges SB, Dudley J and Kumar S. TimeTree: a public knowledge-base of divergence times among organisms. Bioinformatics. 2006;22 23:2971-2. doi:10.1093/bioinformatics/btl505.

67. De Bie T, Cristianini N, Demuth JP and Hahn MW. CAFE: a computational tool for the study of gene family evolution. Bioinformatics. 2006;22 10:1269-71. doi:10.1093/bioinformatics/btl097.

68. Kimura M. A simple method for estimating evolutionary rates of base substitutions through comparative studies of nucleotide sequences. J Mol Evol. 1980;16 2:111-20.

69. Blanc G and Wolfe KH. Widespread paleopolyploidy in model plant species inferred from age distributions of duplicate genes. Plant Cell. 2004;16 7:1667-78. doi:10.1105/tpc.021345.

70. Ayroles JF, Carbone MA, Stone EA, Jordan KW, Lyman RF, Magwire MM, et al. Systems genetics of complex traits in Drosophila melanogaster. Nat Genet. 2009;41 3:299-307. doi:10.1038/ng.332.

71. Kayser M and Parson W. Transitioning from Forensic Genetics to Forensic Genomics. Genes (Basel). 2017;9 1 doi:10.3390/genes9010003.

72. Jager AC, Alvarez ML, Davis CP, Guzman E, Han Y, Way L, et al. Developmental validation of the MiSeq FGx Forensic Genomics System for Targeted Next Generation Sequencing in Forensic DNA Casework and Database Laboratories. Forensic Sci Int Genet. 2017;28:52-70. doi:10.1016/j.fsigen.2017.01.011.

Figure 1

Click here to access/download;Figure;Figure 1.pdf

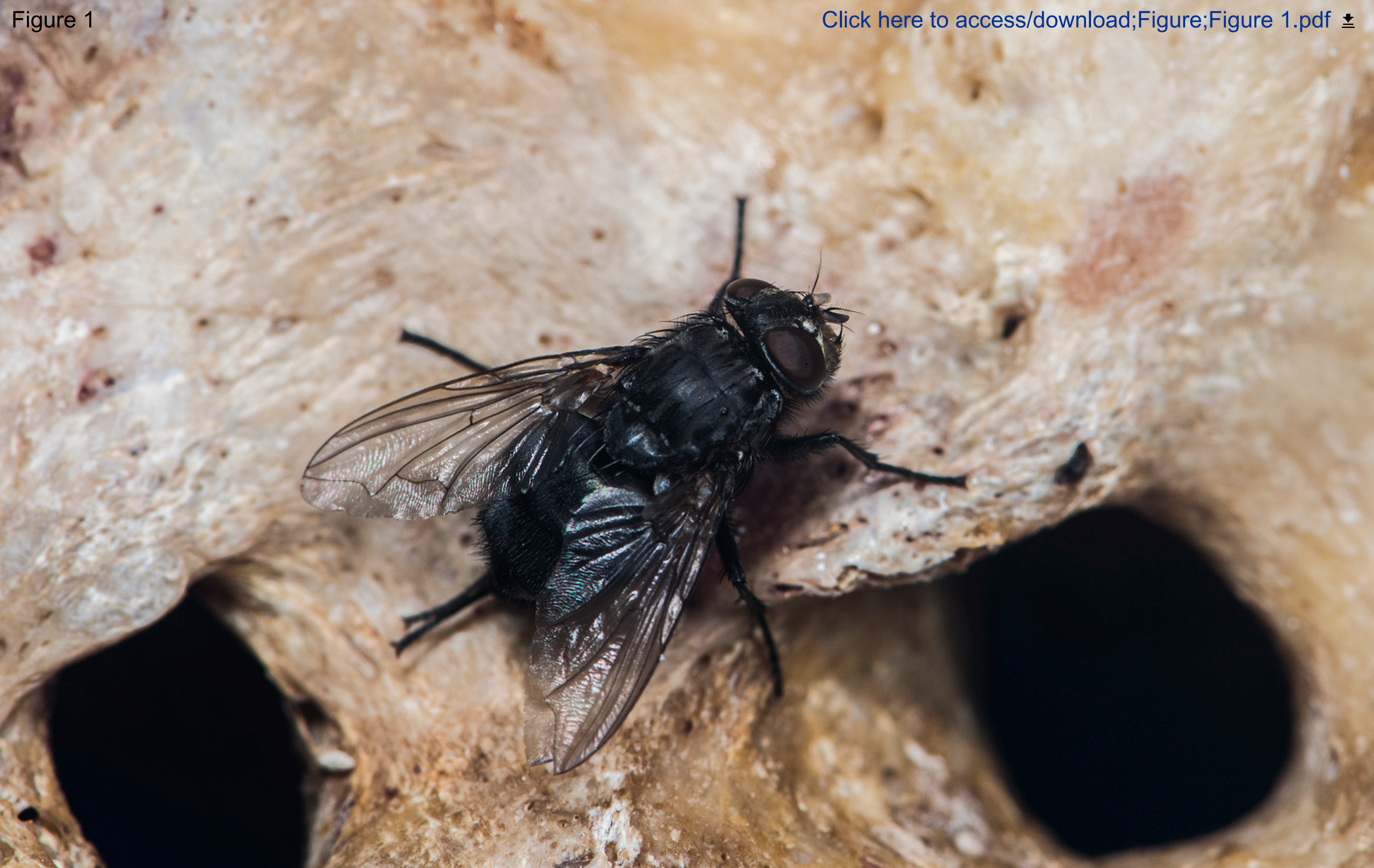

Figure 2

Click here to access/download;Figure;Figure 2.tif

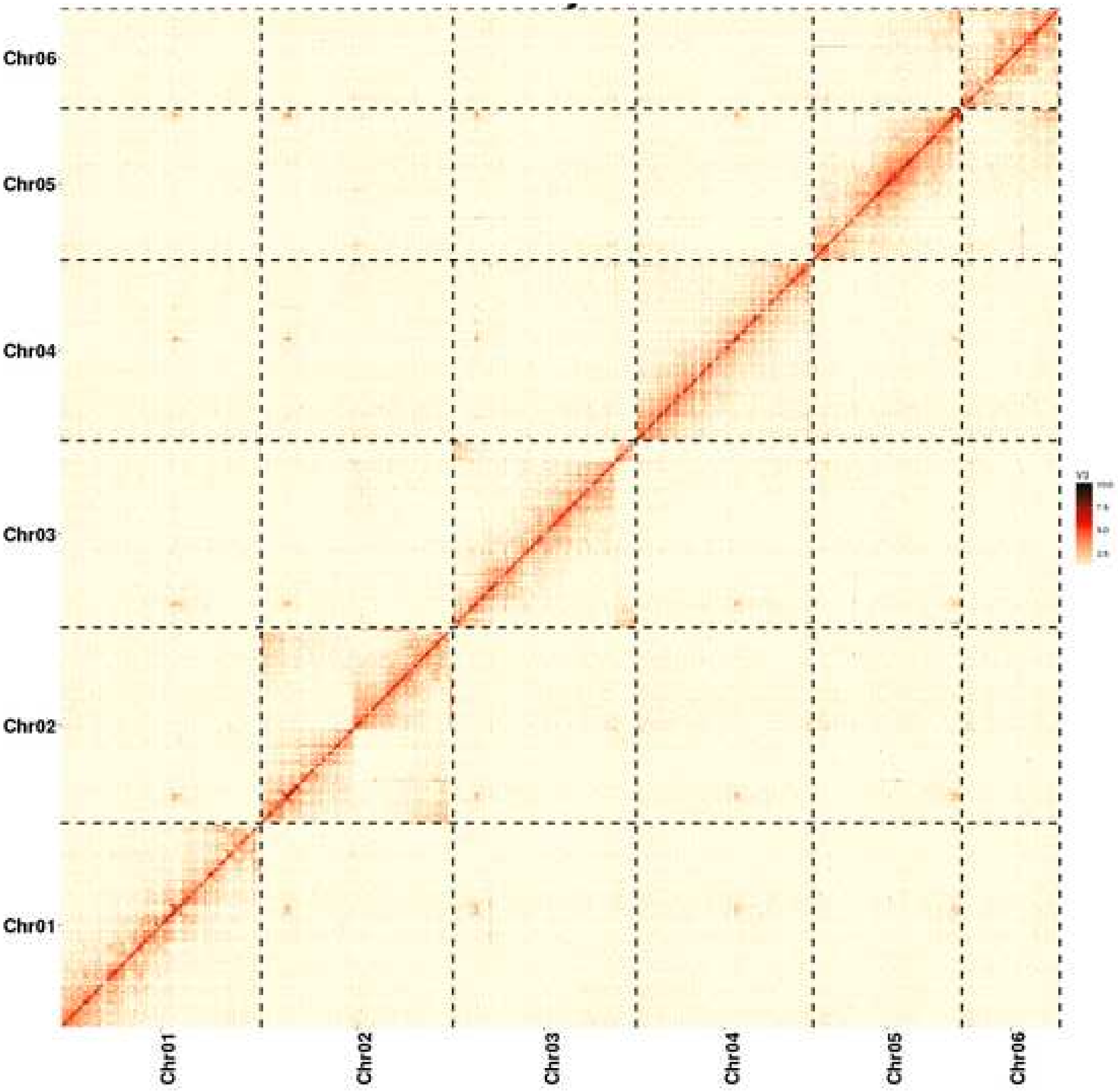

 Click here to access/download;Figure;Figure 3.tif

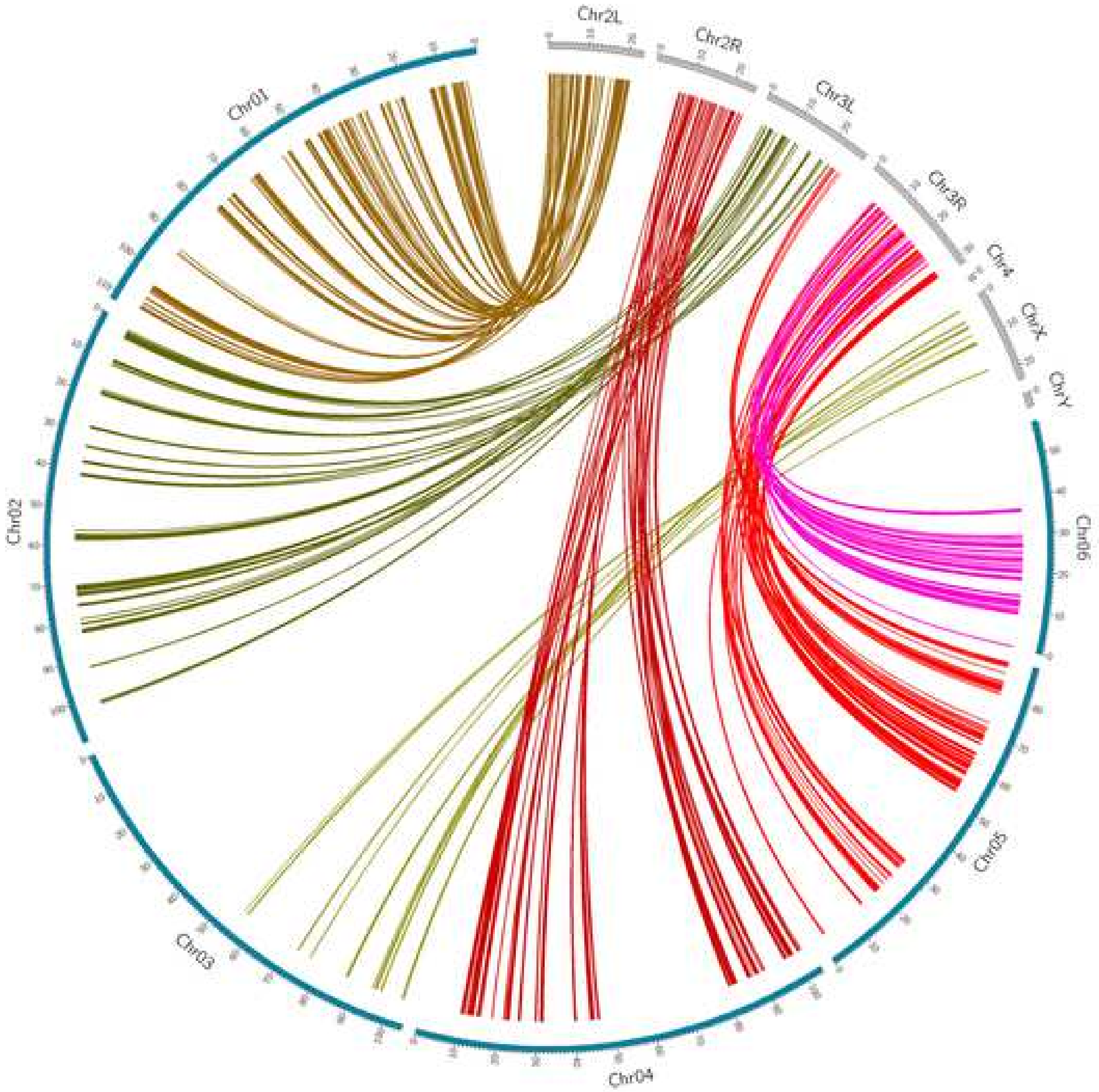

Figure 4

Click here to access/download;Figure;Figure 4.tif

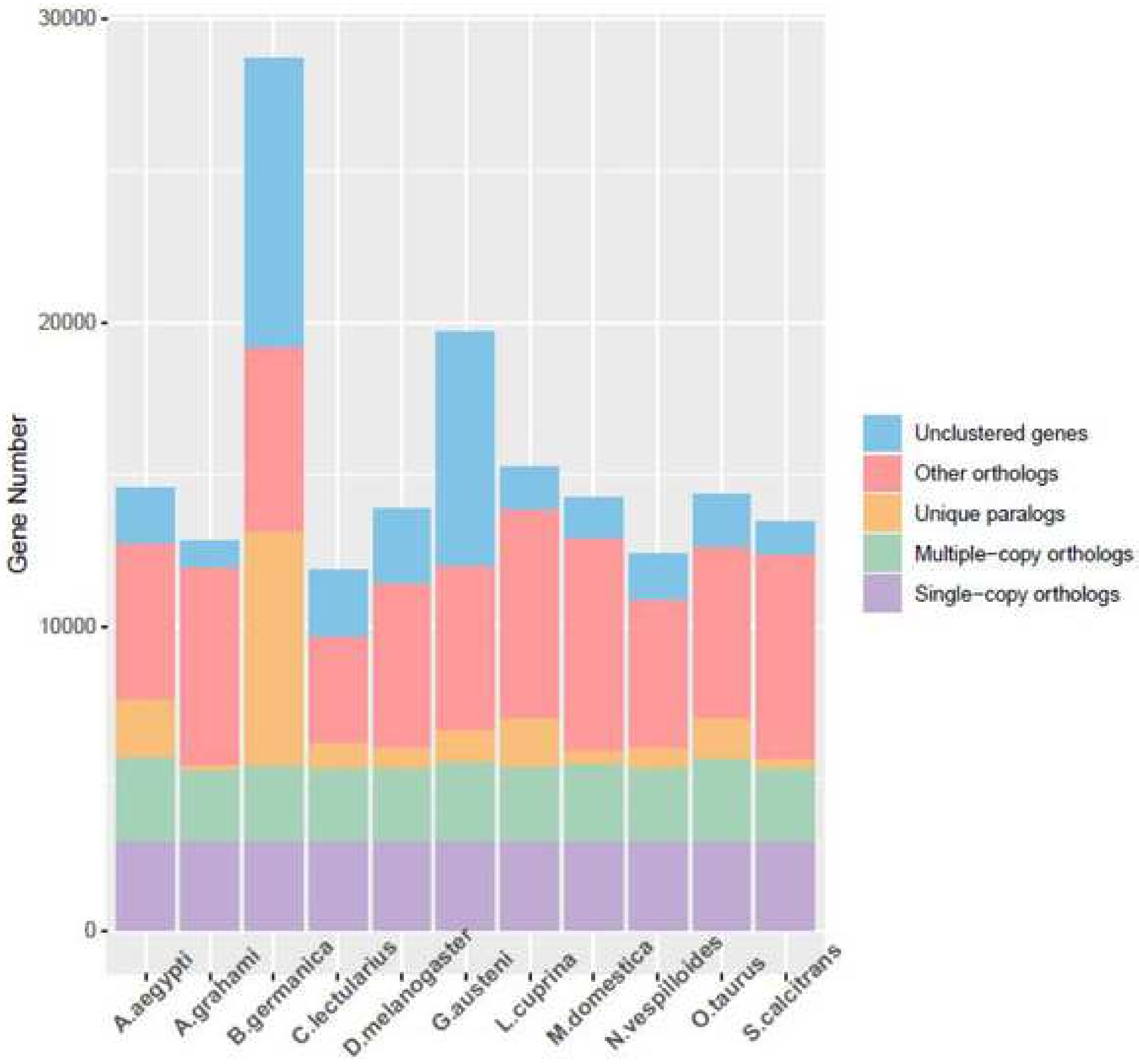

Figure 5

Click here to access/download;Figure;Figure 5.tif

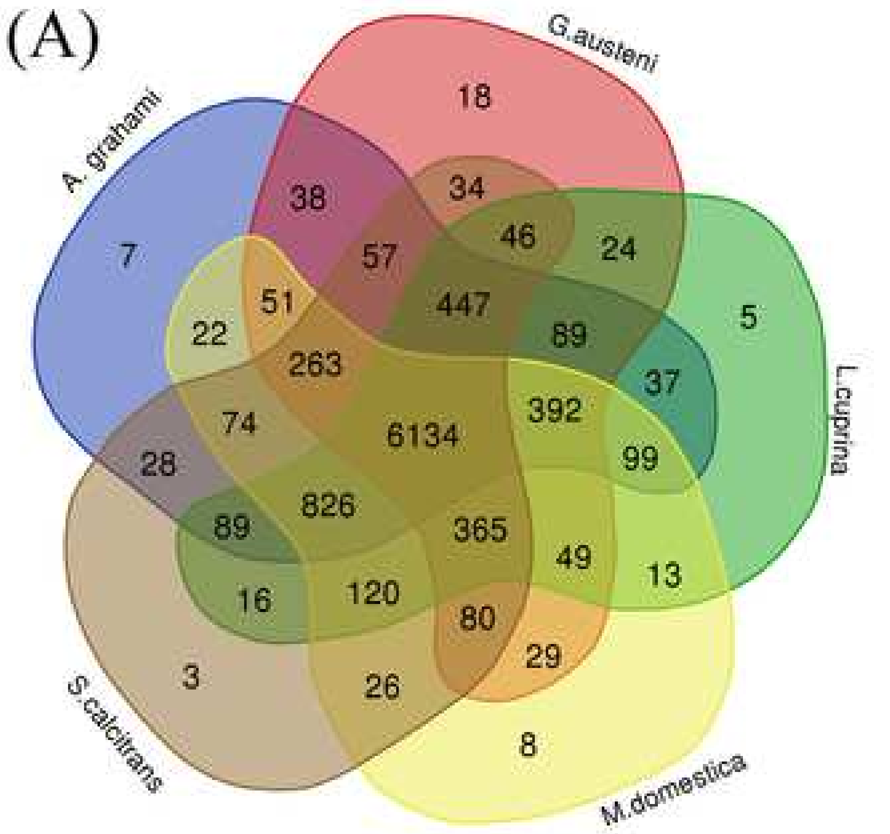

Figure 6

Click here to access/download;Figure;Figure 6.tif

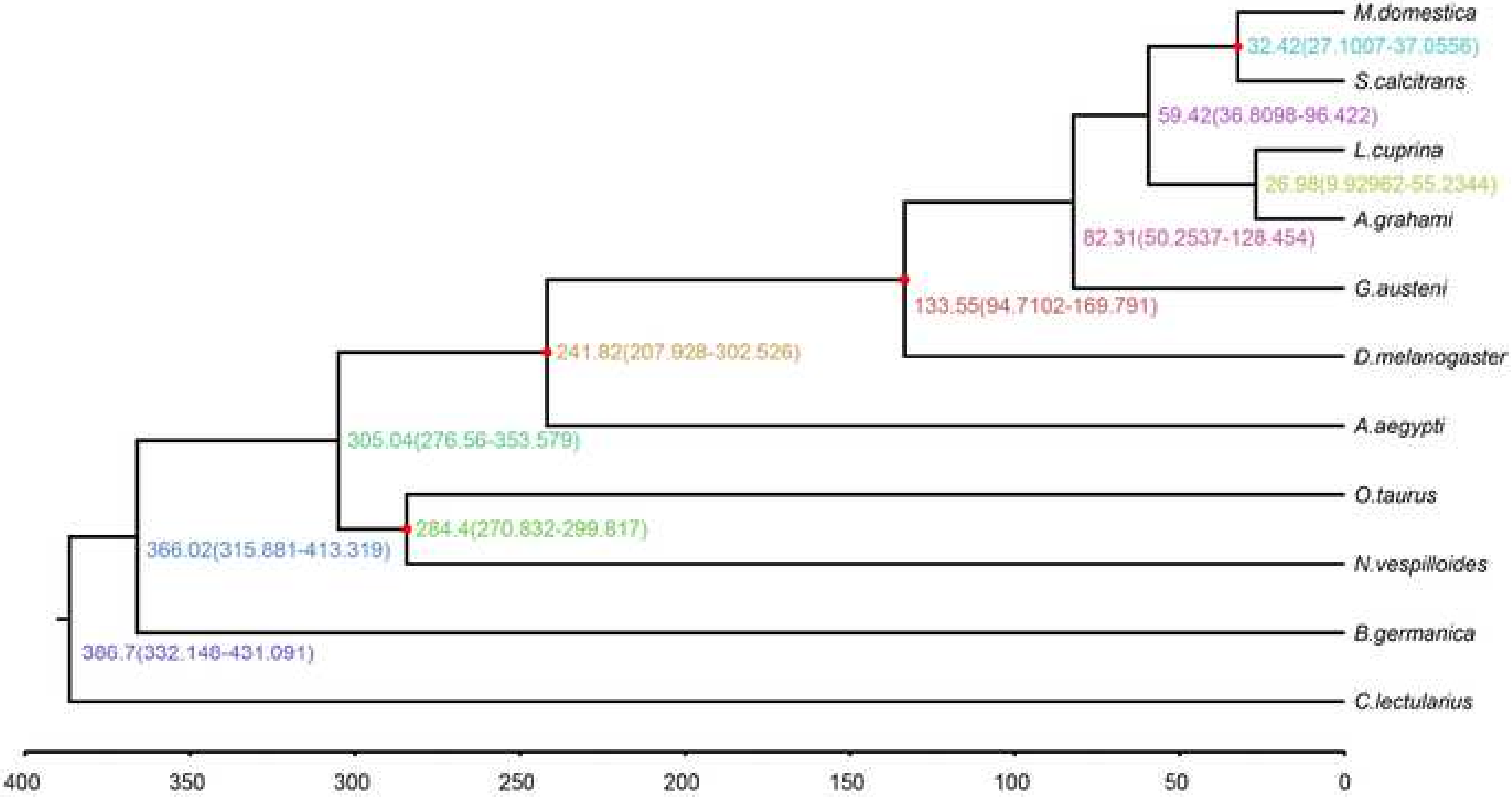

Figure S1

Click here to access/download;Figure;Figure S1.tif

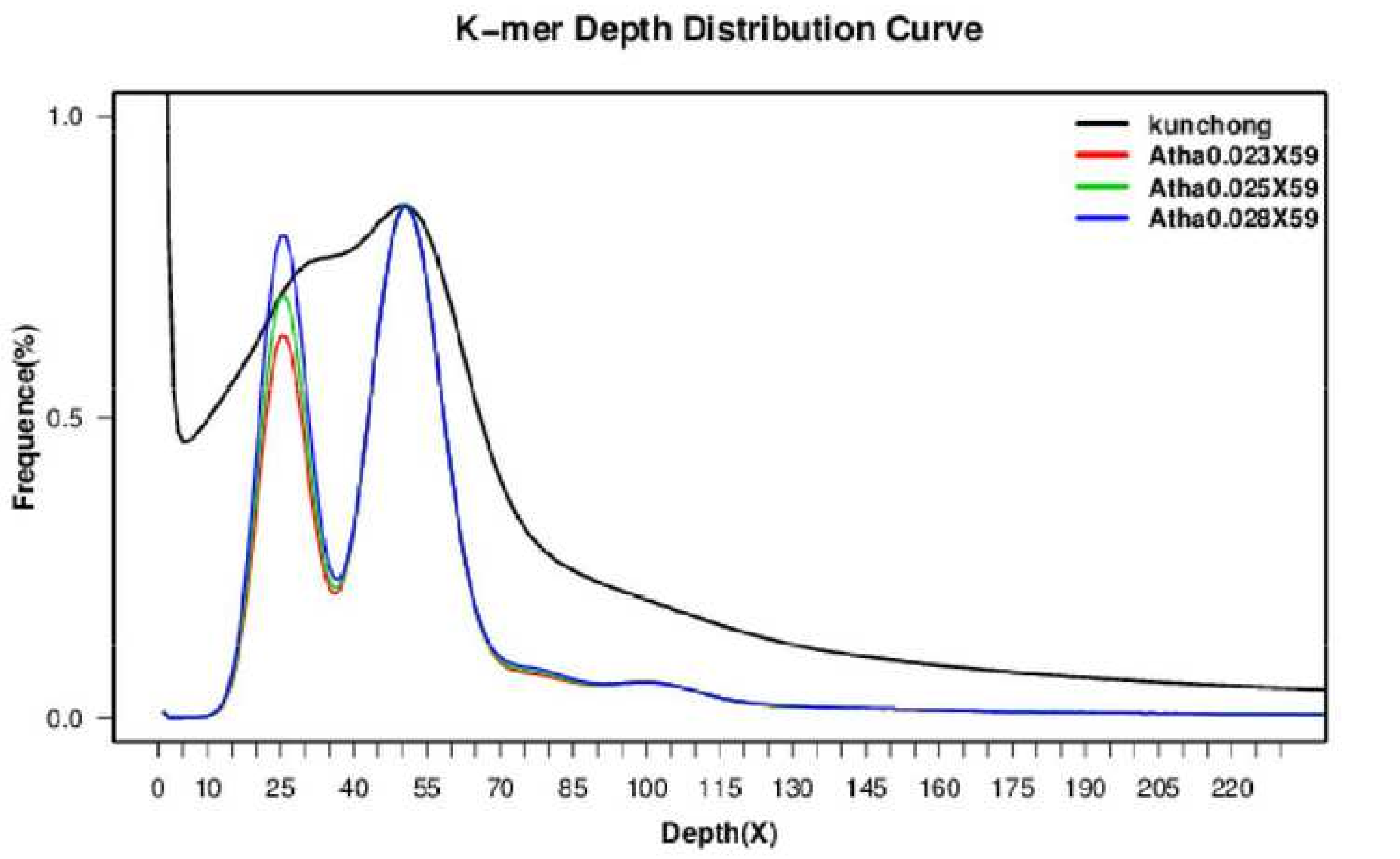

Figure S2

Click here to access/download;Figure;Figure S2.tif

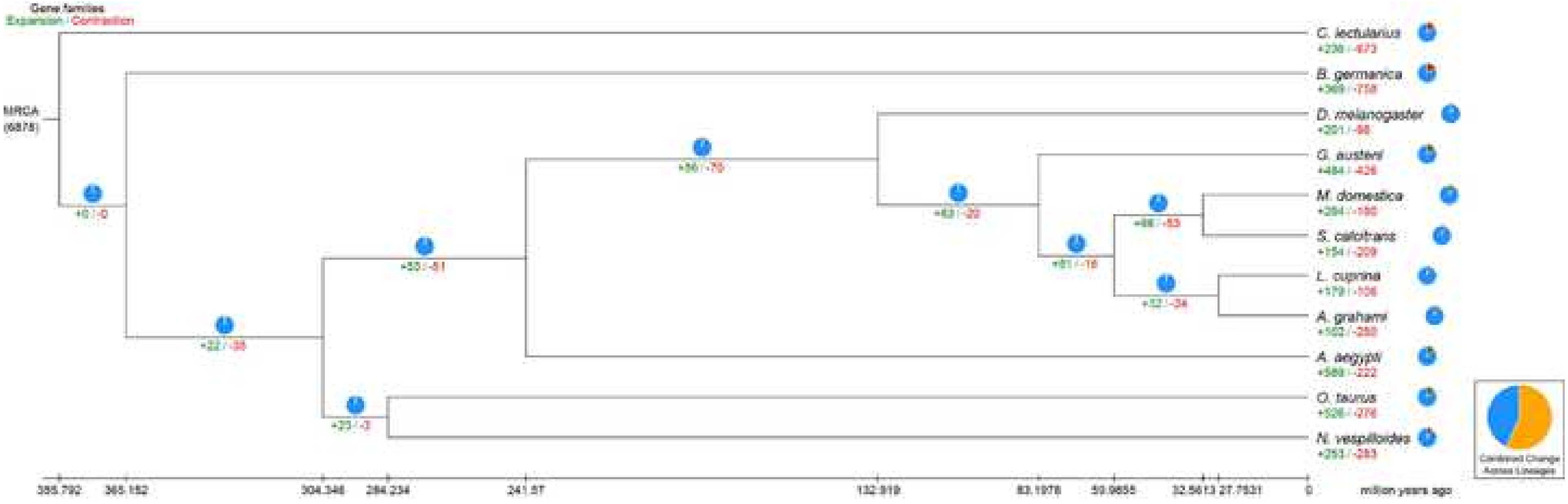

Figure S3

Click here to access/download;Figure;Figure S3.tif

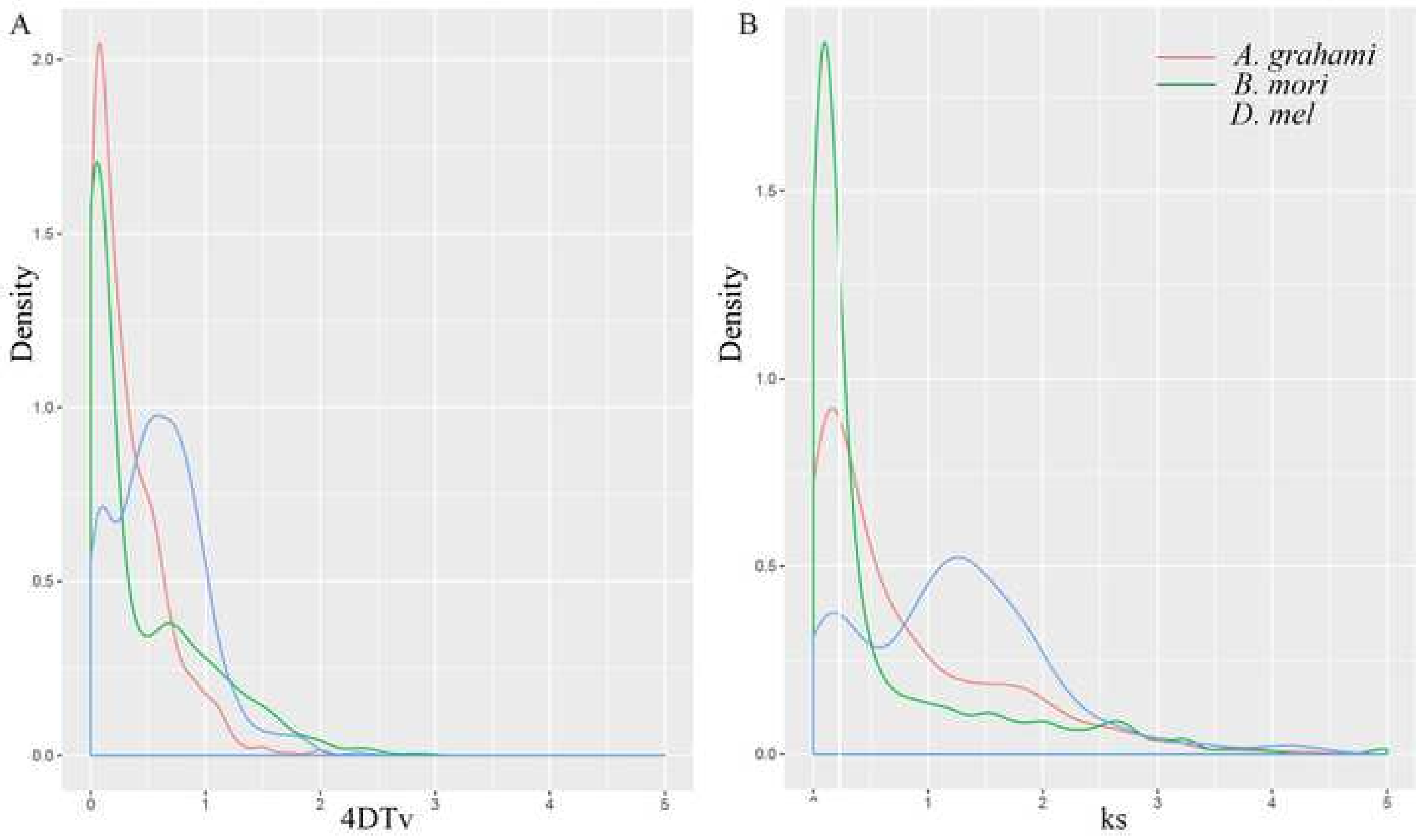

supplementary table S1-10

Click here to access/download
Supplementary Material
supplementary table 20190227.docx

supplementary table S11

Click here to access/download
Supplementary Material
Table S11.xlsx

Cover letter Click here to access/download;Personal Cover;Cover letter.doc

Dear Editor,

We would like to submit the enclosed manuscript entitled "**Chromosomal-level genome assembly of *Aldrichina grahami*, a forensically important blow fly**" for possible publication in the journal of ***GigaScience***.

Flies of Calliphoridae belong to the most frequent entomological evidence applied in **forensic cases**. ***Aldrichina grahami*** has some unique biological characteristics which obviously different from the other blow fly species. It has great potential application value in forensic research and practice like estimation on the minimum postmortem interval (min**PMI**) and other aspects. But a qualified genomes resource of it is still unavailable.

Here we provide a genome assembly with high quality of *A. grahami* generated by **Pacifc BioSciences (PacBio) Sequel** sequencing platform and **Hi-C** technology. It represents the first **chromosome-level** genome resource in calyptratae. This robust genome reference should facilitate the development of research on *A. grahami* and other **necrophagous** blow fly species. **Forensic entomological studies** based on qualified genome resource will definitely consolidate the reliability of entomological evidence and promote its application in **law suit**.

All authors have reviewed the final version of the manuscript and approve it for publication. This manuscript has not been published in whole or in part nor is it being considered for publication elsewhere.

Funding

The present study was supported by grant of the National Natural Science Foundation of China (81571855) and Science Foundation of Hunan Province (2017SK2015).

Ethical approval

None.

Conflict of interest

All authors declare that no competing interests in present work.

Address: School of Basic Medical sciences, Central South University, Changsha 410013, Hunan, China

E-mail: cjf_jifeng@163.com

Tel: +86 731 82650414
