# Supplementary material for: Chromosome-level genome assembly of Aldrichina grahami, a forensically important blowfly
Source: Gigascience. 2020 Mar 19;9(3):giaa020. doi: 10.1093/gigascience/giaa020 (PMC7081965; doi:10.1093/gigascience/giaa020)
Supplement: giaa020_GIGA-D-19-00066_Revision_1 [file giaa020_giga-d-19-00066_revision_1.pdf]

GigaScience

Chromosomal-level genome assembly of Aldrichina grahami, a forensically important blow fly

--Manuscript Draft--

**Manuscript Number:**	GIGA-D-19-00066R1
**Full Title:**	Chromosomal-level genome assembly of Aldrichina grahami, a forensically important blow fly
**Article Type:**	Data Note
**Funding Information:**	National Natural Science Foundation of China (81571855) — Pro. Jifeng Cai<br>Science Foundation of Hunan Province (2017SK2015) — Pro. Jifeng Cai
**Abstract:**	Abstract<br>Background: Blow flies (Diptera: Calliphoridae) are the most common entomological evidence found in forensic investigation. Aldrichina grahami is a forensically related blow fly species with some unique biological characteristics which distinguish it from other blow fly species. Its development rate, pattern and life cycle can provide valuable information for estimation of the minimum postmortem interval (minPMI).<br>Findings: Here we provide a chromosomal-level genome assembly of A. grahami which was generated by Pacific BioSciences (PacBio) sequencing platform and chromosome conformation capture (Hi-C) technology. A total of 50.15 Gb clean reads of A. grahami genome was generated. Program FALCON and Wtdbg, as the common assembly tools for PacBio long reads, were utilized to construct the genome of A. grahami. And it resulted in an assembly of 600 Mb and 1604 contigs, including 1.93 Mb contig N50. We predicted 12823 protein-coding genes and 99.8% of it was functionally annotated based on data of de novo genome (SRA: PRJNA513084) and transcriptome (SRA: SRX5207346) of A. grahami. Gene families clustering and phylogenetic reconstruction were performed based on co-analyses with other 10 insect species. A Hi-C sequencing and analysis generated a nearly chromosomal-level assembly within 6 chromosomes and scaffold N50 of 104.7 Mb. Nearly 96.4% of these scaffolds was anchored to A. grahami genome total contig bases.<br>Conclusions: The present study provides a robust genome reference of A. grahami which adds vital genetic information for nonhuman forensic genomics, and facilitates to future research on A. grahami and other necrophagous blow fly species used in forensic medicine.
**Corresponding Author:**	Jifeng Cai<br><br>CHINA
**Corresponding Author Secondary Information:**	
**Corresponding Author's Institution:**	
**Corresponding Author's Secondary Institution:**	
**First Author:**	Fanming Meng
**First Author Secondary Information:**	
**Order of Authors:**	Fanming Meng
	Jifeng Cai
	Zhuoying Liu
	Han Han
	Dmitrijs Finkelbergs
	Yangshuai Jiang

Powered by Editorial Manager® and ProduXion Manager® from Aries Systems Corporation

	Mingfei Zhu
	Chao Chen
	Yadong Guo
	Yang Wang
	Zongyi Sun
**Order of Authors Secondary Information:**	
**Response to Reviewers:**	Dear Editor Zauner and reviewers,<br>We would like to express our great appreciation to you and reviewers for constructive comments and suggestions on our manuscript. Those are really valuable and helpful for revising and improving our paper.<br>Here we are submitting the revised version of the manuscript “Chromosomal-level genome assembly of Aldrichina grahami, a forensically important blow fly” by Meng et al. (GIGA-D-19-00066). We have studied those comments carefully and tried our best to improve the manuscript. We hope that the correction will meet with approval.<br>In this revised version, we have provided additional test and analyses to corresponding parts as reviewers suggested. We also have polished the writing and had native English speaker to review the manuscript. Changes were marked all in RED.<br>We integrated some additional analyses into the result of chromosome assembly after Hi-C sequencing as the reviewer suggested. For a more logical structure of the manuscript, we moved the section of “Chromosome assembly using Hi-C data” into the front of the “conclusion” section. The order of figures and tables were changed correspondingly. At last, we made a point-to-point response to the reviewers' comments.<br>Thanks again to the reviewers and editors of GigaScience for patiently revised our previous submission and giving us the opportunity to make improvement. We are looking forward to hearing from you.<br>Sincerely yours!<br><br>Jifeng Cai, on behalf of all authors<br>Forensic Science department, School of Basic Medicine, Central South University.<br>Changsha, Hunan Province, China<br>Email: cjf_jifeng@163.com<br><br>Reviewer #1:<br>Abstract:<br>Line 21: Clarify/rephrase introductory sentence<br>RESPONSE: We are very sorry for the ambiguous expression. The sentence has been rephrased. (Line 25)<br><br>Line 25: Change "provides" to "provide" - verb tense error<br>RESPONSE: It has been changed. (Line 29)<br><br>Line 26: Please elaborate on "forensic issues"<br>RESPONSE: Great thanks to the reviewer's advice. We rethought this sentence and found it may inappropriate to state the value of A. grahami on ‘other forensic issues’ based on limited reports and studies about this species so far. The most important meaning of fly developmental rate, pattern and life cycle is to provide information for minPIM estimation. We deleted the statement on ‘other forensic issues.’ (Line 30)<br><br>Line 28 & 94: "Pacific Biosciences" is mis-spelled<br>RESPONSE: It has been corrected. (Line 32, 101,142)<br><br>Line 29: Change "Totally" to "A total of"<br>RESPONSE: Changed. (Line 33)<br><br>Line 30: Elaborate. FALCON and Wtdbg are genome sequence assemblers for PacBio long reads<br>RESPONSE: Many thanks for the reviewer's suggestion. We have revised this sentence, and elaborated them in the section of “Genome survey and Genome assembly ”. (Line 34, 181)

	Reference<br>Eid J, Fehr A, Gray J, Luong K, Lyle J, Otto G, et al. Real-time DNA sequencing from single polymerase molecules. Science. 2009; 323 5910: 133-8. doi:10.1126/science.1162986.<br><br>Line 30: Change "was" to "were" - verb tense error<br>RESPONSE: Changed. (Line 35)<br><br>Line 34 & 190: Clarify "Nearly 96.4% of these scaffolds was anchored to A. grahami genome"<br>RESPONSE: Thank you for your suggestion. We have revised this sentence both in the Abstract and the section "Chromosome assembly using Hi-C data". (Line 43, 376)<br><br>Line 36: Clarify what "de novo and transcriptome" data was used for genome annotation. Are these data deposited and available online?<br>RESPONSE: We are sorry for didn't provide these information in the previous version. In fact, all the data of de novo genome and transcriptome of A. grahimi have been deposited into NCBI SRA database. It is available and can be located by accession number of PRJNA513084 (de novo genome) and SRX5207346 (transcriptome) now. We have described them in the new manuscript. (Line 39)<br><br>Line 40: Change "add" to "adds", and "facilitate" to "facilitates" - verb tense errors<br>RESPONSE: Changed as suggested. Thank you so much for your patient revision. (Line 45)<br><br>Line 41: "Necrophgous" is mis-spelled<br>RESPONSE: Changed. (Line 48)<br><br>Data Description:<br>Line 47: Clarify/rephrase sentence<br>RESPONSE: Rephrased. (Line 52)<br><br>Line 52: Is "it" A. grahami or the minPMI - please clarify<br>RESPONSE: We are sorry for this ambiguous phrase. We have rephrased the sentence to avoid misunderstanding. (Line 55)<br><br>Line 57: Clarify/rephrase sentence<br>RESPONSE: Rephrased. (Line 61)<br><br>Line 57: When referring to "life history" do you mean life cycle?<br>RESPONSE: As the reviewer pointed out, "life cycle" is more appropriate than "life history" to express the meaning. Thank you. (Line 62)<br><br>Figure 1: Remove "remain" from figure legend<br>RESPONSE: Changed. (Line 66)<br><br>Line 72: Combine both sentences, or rephrase<br>RESPONSE: Rephrased. (Line 76)<br><br>Line 72: When referring to "whole life history" do you mean whole life cycle?<br>RESPONSE: As the reviewer mentioned above, it should be "life cycle". (Line 76)<br><br>Line 74: When referring to "season of death" do you mean reason of death?<br>RESPONSE: Thank you for your kind reminding. We previously used "season of death" to describe that A. grahami could be utilized to infer the death event happened in which season. This information could narrow the time range of investigation. To avoid misunderstanding, we rephrased this sentence. (Line 79)<br><br>Line 76: Clarify and elaborate why these studies indicate great potential application value<br>RESPONSE: Thank you very much for your advice. We have clarified and elaborated on this information here, and added brief description in the new version of our manuscript. (Line 80)<br>Many efforts had been given to identify human DNA material from the gut of larvae

Powered by Editorial Manager® and ProduXion Manager® from Aries Systems Corporation

which feed on the corpse. The gut of larvae could be good ‘container’ for human DNA material to some range. The human DNA containing in larvae gut can be used to identify a missing body or prove the presumed association with the entomological evidence of a corpse (interpreting evidence used for forensic investigation). For instance, the corpse was removed and only maggots were discovered in the scene. And sometimes insect evidences from one crime scene was divided and sent to different investigators.

Cuticular hydrocarbons, including more than 100 compounds, are the major components of insect cuticle. Previous studies have demonstrated that the composition of cuticular hydrocarbons vary by maturity or age. And this variation had been analyzed in some forensic important species of fly larvae and used for PMI estimation. The cuticular hydrocarbons of larvae of A. grahami were also reported as a potential indicator to its development and had the potential value in the forensic investigation on PMI.

Reference

Zehner R, Amendt J and Krettek R. STR typing of human DNA from fly larvae fed on decomposing bodies. Journal of forensic sciences. 2004;49 2:337-40.
Li K, Ye GY, Zhu JY and Hu C. Detection of food source by PCR analysis of the gut contents of Aldrichina grahami (Aldrich) (Diptera: Calliphoridae) during post-feeding period. Insect Sci. 2007; 14 1:47-52. doi:10.1111/j.1744-7917.2007.00124.x.
Xu H, Ye GY, Xu Y, Hu C and Zhu GH. Age-dependent changes in cuticular hydrocarbons of larvae in Aldrichina grahami (Aldrich) (Diptera: Calliphoridae). Forensic science international. 2014; 242: 236-41. doi:10.1016/j.forsciint.2014.07.003.
Moore HE, Adam CD and Drijfhout FP. Potential Use of Hydrocarbons for Aging Lucilia sericata Blowfly Larvae to Establish the Postmortem Interval. Journal of forensic sciences. 2013; 58 2:404-12. doi:10.1111/1556-4029.12016.

Line 80 & 84 & 91 & 109 & 236 & 305: Suggest to not begin a sentence with 'And'
RESPONSE: Changed. Thank you so much for your patient revision. (Line 87,91,98,124,236,317)

Line 95: As it appears here for the first time, please introduce "Hi-C" with its full name "Chromosome conformation capture"
RESPONSE: Changed as the reviewer suggested. (Line 102)
Genome Sequencing and Assembly:

Line 135: Refer to "SMRT cell" as Sequel SMRT cells, or PacBio SMRT cells
RESPONSE: Changed. (Line 157)

Line 141: How were the Hi-C libraries constructed?
RESPONSE: Thanks for the reviewer’s reminding. The procedure of Hi-C libraries construction has been added in the manuscript. (Line 163-173)

Reference

Rao S S P, Huntley M H, Durand N C, et al. A 3D map of the human genome at kilobase resolution reveals principles of chromatin looping. Cell, 2014, 159(7): 1665-1680.

Line 145: Estimated genome size was calculated using kmer-based calculation. Was there a reason why the kmer length of 17-mers was used (as opposed to >20-mers, or 25-mers which would ensure a more robust estimate). The authors should make this calculation using other kmer-lengths to ensure their calculations are correct.
RESPONSE: Great appreciation to the reviewer’s suggestion and reminding.
1) Before estimating genome size, the length of k-mer should be determined. It should be large enough to ensure that most k-mers are unique in the investigated genome, and small enough also to avoid the overload of computer memory. 17-mer was commonly used in the estimation of genome size. 2) Sequences in 17bp length consisted of four nucleotide base (ATCG) could theoretically form a 4^17, almost 17Gb sequence data in total, which could cover most insect genome assembly (based on statistics of Genome size web resource, http://www.genomesize.com/). 3) Errors can be generated in the sequencing process, and larger k-mer size will enhance the effect of heterozygosity and sequencing error. It is suggested using smaller k-mer size to analysis data with high heterozygosity and sequencing error rates. In addition, to prevent the interference of the palindrome sequence in the performing the De Berjin analyses, odd base number in k-mer length was used.

Reference

Binghang, Liu & Shi, Yujian & Yuan, Jianying & Galaxy, Yuuki & Zhang, Hao & Li, Nan & Li, Zhenyu & Chen, Yanxiang & Mu, Desheng & Parkin, Isobel. (2013). Estimation of genomic characteristics by analyzing k-mer frequency in de novo genome projects.

Table 1: "Pac Bio" is mis-spelled
RESPONSE: Changed. (Table 1)

Line 175: Suggest replacing "proved" with "supported"
RESPONSE: Changed as suggested. Thanks. (Line 206)

Line 198: Which D. melanogaster genome was used? Please reference
RESPONSE: The genome of D. melanogaster was downloaded from the following link https://www.ncbi.nlm.nih.gov/genome/47. We have added this link as reference in new manuscript. (Line 383)

Line 200: Please elaborate on MCScanX methodology
RESPONSE: The MCScanX methodology has been elaborated in the revised manuscript. (Line 383-386)
Reference
Wang Y, Tang H, DeBarry J D, et al. MCScanX: a toolkit for detection and evolutionary analysis of gene synteny and collinearity. Nucleic acids research, 2012, 40(7): e49-e49.
Functional Prediction and Genome Annotation:

Line 209: Change "was" to "were" - verb tense error
RESPONSE: Changed. (Line 221)

Line 211: Microsatellite is used here, instead of SSR - suggest consistent use of one term
RESPONSE: Thank you for your suggestion. It has been hanged. (Line 223)

Table 2: Suggest clarifying the description of "Other*"
RESPONSE: Thank you for your suggestion. We have clarified the description of "Other*" in the note of Table 2. (Line 241)

Line 237: Which D. melanogaster gene model?
RESPONSE: The gene model used in present manuscript is under the following link https://www.ncbi.nlm.nih.gov/genome/47. We cited it in the manuscript. (Line 251)

Line 247: Spelling error "homologys"
RESPONSE: Changed. (Line 260)

Line 248: Spelling error "finial"
RESPONSE: Changed. (Line 261)

Table 3: Suggest replacing "Swissprot" with "Swiss-Prot", "Trembl" with "TrEMBL", "Kegg" with "KEGG", "InterProscan" with "InterProScan". What does "nr" stand for? Non-redundant? Please clarify.
RESPONSE: Thanks a lot for your kind suggestion. We have changed all the database names to the formal pattern both in the manuscript and additional files. The ‘nr’ used in the manuscript is refer to ‘Non-Redundant Protein Sequence Database’. Thank you for pointing out this problem. (Line 265-273, table 3)

Evolutionary analyses:
Line 277: Change "exploring" to "explore" - verb tense error
RESPONSE: Changed. (Line 346)

Line 280: Please reference these genomic resources
RESPONSE: The links of these resources were referenced and also listed in the additional file Table S7. (Line 292)

Line 282: Why was BLASTP done independently prior to OrthoMCL?
RESPONSE: Thank you for pointing out this problem. The previous statement was

Powered by Editorial Manager® and ProduXion Manager® from Aries Systems Corporation

ambiguous. In fact, the OrthoMCL starts with all-against-all BLASTP comparisons of a set of protein sequences from genomes. We have rephrased it. (Line 292)
Reference
Li L, Stoeckert C J, Roos D S. OrthoMCL: identification of ortholog groups for eukaryotic genomes. Genome research, 2003, 13(9): 2178-2189.

Line 287: Clarify what "statistics results" are referred to here
RESPONSE: The previous statement was inappropriate. It should not be a ‘statistics results’. We have rephrased this statement. Thanks for your comment. (Line 298)

Line 293: These phylogenetic analyses are not clear. Please add detail.
RESPONSE: The phylogenetic analyses are described in the Lines 338-340.

Line 306: How were these analyses performed?
RESPONSE: These analyses were performed by an online Venn drawing tool. We have added this description in the new manuscript. (Line 316)

Figure 6: Spelling error "notes"
RESPONSE: Changed. (Line 342)

Line 334: Change "was" to "were" - verb tense error
RESPONSE: Changed. (Line 347)

Line 337: Is "extinct" the correct term?
RESPONSE: Thank you for your reminding. We have changed it to “lost”. (Line 350)

Line 342: Change "was" to "were" - verb tense error
RESPONSE: Changed. (Line 356)

Line 344: Suggest replacing "Blastp" with "BLASTP" to be consistent with line 282
RESPONSE: Changed as suggested. Thanks (Line 357)
Conclusion:

Line 353: Change "forensically" to "forensic" - verb tense error
RESPONSE: Changed. (Line 411)

Line 354: Spelling error "recourse"
RESPONSE: Changed. (Line 412)

Line 362: Provide references for this statement, as this is the first mention of repellent development.
RESPONSE: It is really true as Reviewer suggested. Thanks a lot to the reviewer for pointing out this insufficient statement. The common method to prevent and treat flystrike was relied on insecticides. But the rapid emergence of resistance in blowflies is becoming problematic. So repellent could be a better choice and predicting method to prevent flysrtike. For there has no more documentation on repellent development in our present manuscript, and it’s not the main focus for we work, we would like to delete this phrase. (Line 418)

References
A vast number of references cited in the text did not match the reference list. Will the RRID citations be replaced with URLs?
RESPONSE: Thank you so much for pointing out this mistake in our references. It was caused by auto-update of citation index at different personal PC which has different reference assemblage. We have carefully revised all the references in this new submitted manuscript. Thanks again.

Supplementary Material:
Table S1: What does "inter size" refer to?
RESPONSE: A mistyping input. It should be ‘insert’. We have changed it.

Fig S2: Spelling error "Brach"
RESPONSE: Changed. (Line 432)

Powered by Editorial Manager® and ProduXion Manager® from Aries Systems Corporation

Table S8: Please confirm gene count numbers. The number for L. cuprina is incorrect.
RESPONSE: Thank you for your kind advice. These genome resources listed in Table S8 were downloaded from the website, except A. grahami. And the gene numbers of each species were generated by our analyses on these resources by ourselves but not referenced from literature. For example, the version of GCF_000699065.1 of Lucilia cuprina was used here. When analyzing the length and number of gene in L. cuprina genome, the non-coding RNA (lncRNA, ncRNA, tRNA etc.) were removed in the annotation file. After this filtering procedure, 15536 protein-coding genes were obtained finally, which different from the literature reported (14554 genes, Clare A. Anstead et al, 2014).

Reviewer #2: The authors do an admirable job of assembling and annotating a blow fly genome. This is an interesting species to study and if published this genome will be a valuable resource for further blow fly research. There are some interpretations and analyses that should be improved before publication.
RESPONSE: We would like to give our great appreciation to you for your positive comments on our work. And your suggestions have priceless helpful and encouragement on improving our manuscript. According to your advice, we have amended the relevant parts and add the additional analyses as you suggested in the new version of the manuscript.

Reviewer #2: There are some minor language issues throughout the document that could be helped by another round of editing, especially when deciding to use singular versus plural forms of a word. There are also some misuses of words (ex. Recourse vs. Resource). Similarly, it would help to standardize language in the paper. For instance, PacBio or Pacbio?
RESPONSE: Thanks for reviewer's suggestion. We have revised the language problem of the whole manuscript. And we have corrected those misuses of proper nouns to the formal pattern. For example, Pacbio to PacBio，Kegg to KEGG, Trembl to TrEMBL, etc. (Line 265-273, table 3)

Reviewer #2: The authors do a good job of justifying the study of this species. In forensic entomology, it is worth noting that there are some assumptions associated with interpreting a minimum PMI, one of which the authors note later in the manuscript. It may help to read Tarone and Sanford 2017 to help clarify the conditional nature of PMImin interpretations in forensic entomology; especially if myiasis is associated with this fly.
RESPONSE: Thanks a lot for your kind advice. We highly agree with the author's (Tarone and Sanford, 2017) statements on the utility of terminology in forensic entomology study and practice. To avoid the possibly over- or underestimate of PMI, certain assumptions should be given before any particular use of terminology in forensic investigation or studies of the forensic entomologist, like minPMI, TOC, or PCI etc. And we will pay our attention to the new progress on this topic in the future. Here, in this revised version of our manuscript, we modified the statement related to minPMI and referenced the article (Tarone and Sanford 2017). (Line 61,64)

Reviewer #2: It would help to provide information regarding how the species was identified. Where/when/how was it collected? How was it identified? Is there a voucher associated with the specimen?
RESPONSE: Thank you very much for pointing out this insufficient description. The information was provided as the reviewer suggested. The first generation of A. grahami was collected by baits of beef liver, from City Changsha, Hunan province of China in March of 2017. They were brought back to the lab and breed up until eggs were laid down on supplied food resource. Then the eggs were collected and hatched, which were used to establish the laboratory population for further research. The species identification was performed through both the morphological and molecular method. We followed the morphological description on literature of Fan (1992) to distinguish the species. Then cytochrome oxidase geneI(COI) was used as molecular marker and was amplified (Primer F: 5-TACAATTTATCGCCTAAACTTCAGCC-3; R: 5-CATTTCAAGCTGTGTAAGCATC-3) from the DNA extraction of A. grahami. The product of amplification was sequenced and did the BLAST search on NCBI website. And it was recognized as A. grahami finally. The specimens used in the following genome sequencing test were all belong to the descendant of the original established laboratory population. All voucher specimens were assigned with a unique code and

Powered by Editorial Manager® and ProduXion Manager® from Aries Systems Corporation

	deposited in forensic insect herbarium of department of forensic science, Central South University, Changsha. We have added brief descriptions and results into the new version of the manuscript. (Line 106-115)<br>Reference<br>Fan, Z. D. 1992. Key to the common flies of China, Science publishing house, Beijing, China.<br><br>Reviewer #2: Similarly, what was specifically sequenced? Males? Females? Both?<br>RESPONSE: The samples used for genome survey, genome sequencing and Hi-C in present work were new emerged and unmated females. And samples used for transcriptom sequencing were new emerged individual of both sexes. We have added this information into relevant parts of the new version of manuscript. (Line 126-132)<br><br>Reviewer #2: With respect to genome size calculated in the document: There are different ways of assessing genome size. Not surprisingly, people that use specific techniques tend to prefer their method for estimating genome size. Many researchers tend to use PCR or sequence to estimate genome size, while others use flow cytometry (and some have used Feulgen densitometry). These methods do not always agree. One appealing aspect of non-PCR / sequencing methods is that they may not be as sensitive to issues associated with repeat sequences. If one evaluates Lucilia cuprina genome size by sequencing versus cytometry, there are very different measures. One can interpret this as cytometry overestimating genome size or as sequencing underestimating it. One feels most comfortable with an assembled genome size when the measures converge. It may be worth doing some cytometry on the strain. If not possible, it is at least worth mentioning that it was not done and that sometimes these measures do not agree.<br>As a good example of what I am discussing above, see: Comparisons with Caenorhabditis (~100 Mb) and Drosophila (~175 Mb) Using Flow Cytometry Show Genome Size in Arabidopsis to be ~157 Mb and thus ~25 % Larger than the Arabidopsis Genome Initiative Estimate of ~125 Mb.<br>MICHAEL D. BENNETT ILIA J. LEITCH H. JAMES PRICE J. SPENCER JOHNSTON Annals of Botany, Volume 91, Issue 5, April 2003, Pages 547-557<br>RESPONSE: Thank you so much for your constructive suggestion and reminding on the method of estimation on genome size. As you suggested, we performed the flow cytometry on the both sexes of A. grahami and compared the results with the previous one based on K-mer analysis. We used fruit fly as the internal stander. And single head of male or female A. grahami was sampled following the procedure of literature (PICARD, JOHNSTON & TARONE, 2012) and performed flow cytometry. The genome sizes of male (667.5 ± 6.013Mb, N=6) and female (682.5 ± 13.64Mb, N=6) has no significant difference (P-value 0.3388). But it is about 15.9% larger than the K-mer based genome size (582.63Mb) that exhibits a similar situation described in the paper you suggested. And it is also 12.5% larger than the assembly genome size (600MB). We add these results to the revised version, and describe the analyzing procedure in an additional file Figure S2. Thanks again for your suggestion on the reference literature and example of study. (Line 208-217)<br>Reference<br>Picard, C. J., J. S. Johnston, and A. M. Tarone. Genome Sizes of Forensically Relevant Diptera. Journal of Medical Entomology 49.1(2012):192-197.<br><br>Reviewer #2: Since the authors have produced predicted chromosomes, there are some obvious missing analyses. For instance, what Muller elements correspond to what chromosomes? In addition, are there repeats, or gene families, gene gain/loss, or genes with high rates of evolution that are biased on certain chromosomes or regions of certain chromosomes? Is there a predicted sex chromosome?<br>RESPONSE: We are very grateful to your suggestion. To check the similarity between A. grahami genome and the published fruit fly (D. melanogaster) genome. We identified the Muller elements on the assembled genome and summarized the results in Figure 6A and Supplementary Table 11 and added the outcomes and the explanations at lines 338-391. The result shows that, the major part of pseudochromosome of A. grahami is collinear with one Muller element of D. melanogaster (AgChr01-Muller B, AgChr02-Muller D, AgChr03-Muller A, AgChr04-Muller C, AgChr06-Muller E), except Chr05. Chr05 had one corresponding region on Muller D and E respectively. And Muller E largely corresponded to AgChr05 and AgChr06. No A. grahami chromosome was collinear with Muller F. For the sex

	chromosome, since Muller A (chromosome X of fruit fly) was uniquely located on Chr03 of A. grahami, thus we expect it should represent the sex chromosome of A. grahami. In addition, we investigated the distributions of long terminal repeat (LTR), gene family expansion or contraction, and genes under positive selection, et al. on the genome using a window size of 1 Mb across each chromosome and plotted the distributions in Figure 6B and add the corresponding explanations in the figure note. We haven't found gene number enrichment for any particular chromosomes - all the chromosomes contain a gene density of around 20 genes/per Mb. However, the results showed that longer chromosomes tend to contain higher number of LRT, except for the case of Chr05. In addition, we noticed that the LTR enriched on specific regions of each chromosome where could present the centromere locations. We also summarized the genome regions that contain a significant greater number of positive genes or gene families (expansion or contraction) in Table S11. A brief description has been added at Lines 392-400. Thanks again.<br><br>Reviewer #2: The presence and absence of Phormia regina in different analyses is conspicuous. Its absence in genome evolution analyses is especially problematic, as it would help resolve issues related to tribe differences in blow flies. It should be included in analyses. If not, please explain why it was not. It may have the lowest BUSCO score in the set analyzed, but it is also highly related to this fly compared to the other flies analyzed. A blow fly targeted analysis is justified.<br>RESPONSE: Thank you for your suggestion. But the uploaded GFF of Phormia regina following the link on NCBI website (https://www.ncbi.nlm.nih.gov/genome/36631) is not available. So we did not use it in our analysis. The relevant data of Phormia regina used in this manuscript is referenced from previous report. And we are also looking forward to add genome resource of this highly related species in our further study.<br><br>Reviewer #2: Are there citations for all genomes used in this analysis? Please confirm that all are published.<br>RESPONSE: Thank you for your advice. We add those genome resource used in present paper as references in the revised version. These genome resources also can be downloaded following the links listed in the additional file.<br><br>Reviewer #2: I would suggest checking citation orders. Some do not appear to line up with their context in the manuscript.<br>RESPONSE: Many thanks for your suggestion. We were also aware of the mismatches references in previous version. It was caused by an auto-upload of references list by software at different computer. We carefully check the reference list in the new version of our manuscript.
**Additional Information:**	
**Question**	**Response**
Are you submitting this manuscript to a special series or article collection?	No
**Experimental design and statistics**<br><br>Full details of the experimental design and statistical methods used should be given in the Methods section, as detailed in our Minimum Standards Reporting Checklist. Information essential to interpreting the data presented should be made available in the figure legends.<br><br>Have you included all the information requested in your manuscript?	Yes

**Resources**<br><br>A description of all resources used, including antibodies, cell lines, animals and software tools, with enough information to allow them to be uniquely identified, should be included in the Methods section. Authors are strongly encouraged to cite Research Resource Identifiers (RRIDs) for antibodies, model organisms and tools, where possible.<br><br>Have you included the information requested as detailed in our Minimum Standards Reporting Checklist?	Yes
**Availability of data and materials**<br><br>All datasets and code on which the conclusions of the paper rely must be either included in your submission or deposited in publicly available repositories (where available and ethically appropriate), referencing such data using a unique identifier in the references and in the "Availability of Data and Materials" section of your manuscript.<br><br>Have you have met the above requirement as detailed in our Minimum Standards Reporting Checklist?	Yes

Powered by Editorial Manager® and ProduXion Manager® from Aries Systems Corporation

Manuscript
Click here to access/download;Manuscript;maitext20190726.docx
Click here to view linked References

Chromosomal-level genome assembly of *Aldrichina grahami*, a forensically important blow fly

Fanming Meng[1], Zhuoying Liu[1], Han Han[1], Dmitrijs Finkelbergs[1], Yangshuai Jiang[1], Mingfei Zhu[2], Yang Wang[2], Zongyi Sun[2], Chao Chen[3], Yadong Guo[1], Jifeng Cai[1]*

[1] School of Basic Medicine, Central South University, Changsha, Hunan Pro, China

[2] Nextomics Biosciences, Wuhan, Hubei Pro, China

[3] Institute of Apicultural Research, Chinese Academy of Agricultural Sciences

* Corresponding authors

Emails:

F. M: mengfanming1984@163.com;

Z. L: 214872404@qq.com;

H. H: 583538543@qq.com;

D. F: dfinkelbergs@yahoo.com

Y. J: 1464804060@qq.com

M. Z: zhumingfei@grandomics.com

Y. W: wangyang-1@grandomics.com

Z. S: sunzongyi@grandomics.com

C. C: chenchaoiar@163.com

Y. G: gdy82@126.com

J. C: cjf_jifeng@163.com

Abstract

Background: Blow flies (Diptera: Calliphoridae) are the most common entomological evidence found in forensic investigation. *Aldrichina grahami* is a forensically related blow fly species with some unique biological characteristics which distinguish it from other blow fly species. Its development rate, pattern and life cycle can provide valuable information for estimation of the minimum postmortem interval (minPMI).

Findings: Here we provide a chromosomal-level genome assembly of *A. grahami* which was generated by Pacific BioSciences (PacBio) sequencing platform and chromosome conformation capture (Hi-C) technology. A total of 50.15 Gb clean reads of *A. grahami* genome was generated. Program FALCON and Wtdbg, as the common assembly tools for PacBio long reads, were utilized to construct the genome of *A. grahami*. And it resulted in an assembly of 600 Mb and 1604 contigs, including 1.93 Mb contig N50. We predicted 12823 protein-coding genes and 99.8% of it was functionally annotated based on data of *de novo* genome (SRA: PRJNA513084) and transcriptome (SRA: SRX5207346) of *A. grahami*. Gene families clustering and phylogenetic reconstruction were performed based on co-analyses with other 10 insect species. A Hi-C sequencing and analysis generated a nearly chromosomal-level assembly within 6 chromosomes and scaffold N50 of 104.7 Mb. Nearly 96.4% of these scaffolds was anchored to *A. grahami* genome total contig bases.

Conclusions: The present study provides a robust genome reference of *A. grahami* which adds vital genetic information for nonhuman forensic genomics, and facilitates to future research on *A. grahami* and other necrophagous blow fly species used in forensic medicine.

Keywords: *Aldrichina grahami*; Blow fly; Necrophagous; Forensic entomology; Minimum postmortem interval; Genome assembly

Data Description

Background

Forensic entomology focuses on the application of insects and other arthropods in the medical legal investigation. Studies of developmental rate of insect colonizers on corpse and insect succession pattern during corpse decaying process, can assist the estimation on the minimum postmortem interval (minPMI) which represents the main tasks of forensic investigation [1-3]. In addition, insect evidence can assist the detection and recognition of wounds, estimation on the time length of neglect or abuse, as well as investigating the death reason [4-7]. The most important group of insects for forensic investigation is Diptera, especially those necrophagous fly species of Calliphoridae [8, 9]. Flies of this fauna, usually called blow fly, consist of many species with the parasite lifestyle or feeding on decaying corpses [10, 11]. The reliable life cycle of necrophagous flies can provide vital information for forensic entomologists or investigators to infer a relative accurate minPMI under certain assumptions [8, 12-14].

Figure 1. Female adult of *Aldrichina grahami* on the corpse.

Aldrichina grahami (Aldrich, 1930; NCBI:txid252811, homotypic synonym: *Calliphora grahami*) (Fig. 1) is a common blow fly species indigenous in East Asia [15, 16] and has expanded to the American continent in the past several decades [17-19]. This blow fly usually breeds on carcasses or feces, and contaminate human food [15]. *A. grahami* is a forensically important insect because of its necrophagous behavior, preference of seasonal distribution, and particularly unique characteristics of

tolerance to low temperature than other necrophagous flies [20-22]. In early spring and late autumn, when the ambient temperature is relatively lower, *A. grahami* is frequently the first species to colonize on the corpse. And this species can be the only colonizer in some extreme cases [23, 24]. The information of seasonal distribution pattern of *A. grahami* could be applied as a potential time indicator for the season of death happened, especially in the period of other insects are inactive [22, 25]. Moreover, the successful extraction and identification of human DNA material from *A. grahami* and other blow fly larvae gut contents provide important information for missing corpse or interpreting evidence used for forensic investigation [26, 27]. And age-dependently altering pattern of cuticular hydrocarbons in larvae cuticle indicates its great potential application value in forensic investigation [28, 29]. In addition to the forensic importance, cases of myiasis caused by *A. grahami* from China have been reported routinely, especially after people traveled back from undeveloped regions [30-33]. This blow fly species is also a potential transmitter of pathogens, like H5N1 influenza virus, which could cause serious public health problems in animals and human being [34].

Development of insect biochemistry and physiology prompts our deeper understanding of *A. grahami* [35-38]. The primary application of nuclear material is focused on distinguishing *A. grahami* from other sibling Diptera species [39-42]. Developmental patterns of *A. grahami* under different environment conditions were also described by researchers [20, 21]. Nonetheless, the genome of *A. grahami* is still unavailable, which impedes further applications in forensic research. Previous studies indicated that the variation in the genetic level among geographic populations of the blow fly has unignorable influence on time length of development and life history of a fly species [43-45]. It is also recommended that the investigation on such forensic

problems should be based on the existence of high-quality genome reference of one species [46-48]. Here we provide a chromosome-scale scaffolding of assembly of this forensically important blow fly generated by Pacific BioSciences (PacBio) sequencing platform and chromosome conformation capture (Hi-C) method and hope to promote the future research of forensic and medical science.

Genome Sequencing and Assembly

Sample preparation

The first generation of *A. grahami* was collected by baits of beef liver, from Changsha (Hunan Province, China) in March of 2017. The species identification was performed through morphological and molecular methods. We followed the morphological description on literature of Fan (1992) to distinguish species [15]. Then cytochrome oxidase gene I (*COI*) was used as a molecular marker and amplified (Primer F: 5-TACAATTTATCGCCTAAACTTCAGCC-3; R: 5-CATTTCAAGCTGTGTAAGCATC-3) from the DNA of *A. grahami* by previous method [39]. The product of amplification was sequenced (ABI 3730xl, USA) and the sequence was searched by BLAST on the NCBI website. And it was recognized as *A. grahami* finally. The blow flies were bred for more than 20 generations in the lab of the school of basic medicine, Central South University. The new emerged and unmated female adults were used for DNA extraction.

After sample collection, tissues were immediately immersed into liquid nitrogen and stored at -80℃. DNA was extracted using the Cetyltrimethyl Ammonium Bromide (CTAB) method followed the introduction of Size-Selected 20 kb SMRTbell™ Libraries for genomic DNA preparation. The quality of the extracted

genomic DNA was checked using gel electrophoresis with 0.7% agarose. Then Nanodrop spectrophotometer (Thermo Fisher Scientific) was used to calculate the DNA purity. The concentration of extracted material was examined by Qubit fluorimeter (Invitrogen, Carlsbad, CA, USA).

New males and females were sampled for transcriptome sequencing. After extraction quality control and library construction, the Illumina Hiseq X10 platform was used to perform the RNA-seq. Five new female adults, which were dissected and removed wings and gut, were used for library construction.

Every voucher specimen was assigned with a unique code and all of them were deposited in forensic insect herbarium of the department of forensic science, Central South University, Changsha.

Library construction and sequencing

Two libraries were constructed before sequencing. Firstly a library of short-insert length (400 bp) was constructed by Illumina TruSeq Nano DNA Library Prep Kits. The short-insert size library sequencing was performed on the Illumina HiSeq X Ten instrument at Genetron Health (Beijing, China) with whole-genome shotgun sequencing (WGS) strategy. A total of 46.05 Gb raw data were collected and subsequently filtered. Finally, 42.4 G clean data for short reads were generated (Table S1).

The long reads library of 20 kb was prepared using a SMRTbell DNA Template Prep Kit 1.0 (PacBio p/n 100-259-100). Genomic DNA material was mechanically sheared to generate DNA fragments of approximately 20 kb by using a Covaris G-TUBE™ (Kbiosciences p/n 520079). Polishing enzymes were used for sheared genomic DNA damage- and end-repair. SMRTbell template was generated by

blunt-end ligation reaction under exonuclease treatment and fragments with proper size were selected by Blue Pippin device (Sage Science, Inc., Beverly, MA, USA) subsequently. The DNA 12000 Kit for Agilent Bioanalyzer 2100 (Agilent p/n 5067-1508) was used to figure out the distribution of fragments with different sizes.

The Sequel Binding Kit 2.0 (PacBio p/n 100-862-200) was used to bind prepared DNA template libraries to the Sequel Polymerase 2.0 in preparation for sequencing on the Sequel System, and finally formed a DNA polymerase/template complex according to the manufacturer's instructions. The enrichment of the larger fragments was improved by the MagBead (PacBio p/n 100-125-900) method. The long-insert size (20 kb) library was sequenced on PacBio Sequel platform with Sequel SMRT cells 1M v2 (PacBio p/n101–008-000), which has one movie of 600 minutes per Sequel SMRT cell at the Genome Center of Nextomics (Wuhan, China). A total of 7 Sequel SMRT cells were processed and the raw data were filtered according to the introduction of the sequencing platform to remove low-quality bases or reads with adapters in the default parameters. In total, 50.15 Gb of long reads clean data were obtained (Table S1). The average length and the N50 of long subreads were 10.51 kb and 15.97 kb respectively.

Hi-C libraries were constructed for *A. grahami* according to the improved Hi-C procedures [49]. The single cell was made by trituration and filtration after treated with 1% formaldehyde solution in PBS buffer at room temperature for 10 minutes to induce crosslinking. The reaction was quenched by adding 2.5M glycine to 0.2M for 5 minutes. Nuclei were digested with 100 units of MboI and marked by biotin-14-dCTP (Invitrogen), and then ligated by T4 DNA Ligase. After reversal of crosslinks, ligated DNA was purified and sheared to a length of 300-600 base pairs, at which point ligation junctions were pulled down by streptavidin beads and prepped for

high-throughput sequencing. Sequencing was performed using the Illumina NovaSeq 6000 Sequencing System (San Diego, CA, USA) with PE150, yielding 74.24 Gb raw data (Table S1).

Genome survey and Genome assembly

The genome size was estimated based on equation $G = k_{num} / k_{depth}$, where the k_{num} was the total number of 17-mers, k_{depth} denoted the peak frequency of 17-mers estimated, and G represented the estimated genome size. Using Jellyfish (v2.1.3, RRID: SCR 005491) [50], 17-mers number was counted as 29,131,491,603 from short clean reads, and the k_{depth} was 50. Therefore, the genome size of *A. grahami* was estimated as 582.63 Mb according to the above equation and the heterozygosity rate of the *A. grahami* genome was approximately 2.5% (Table S2, Fig. S1). FALCON is specifically designed to perform *de novo* assembly for PacBio long reads with about 15% random errors [51]. PacBio long reads were corrected with FALCON (v0.4) and assembled with Wtdbg (v1.2.8) [52, 53], the initial assembly was approximately 596.65 Mb in length, with 1.93 Mb N50 contig. For further improvement on the accuracy of the reference assembly, following steps of polishing strategies were performed for the initial assembly. The pbalign (v.0.3.0) with default parameters was used for Quiver error-correction, and generated an error-corrected genome assembly of PacBio long reads. We used BWA (v0.7.12, RRID: SCR 010910) to map short reads to this assembly. Then it was polished with Pilon (v1.21, RRID: SCR 014731) to generate the second iteration of the assembled genome [54]. Finally, we obtained a polished assembly genome in size of 600.09 Mb, with a 1.93 Mb N50 contig and 1604 contigs (Table 1, Table S3). So far, the present genome has the longest N50 contig length among all the published genome assembly of calyptratae flies of Diptera.

Table. 1 An overview comparison of genome assembly and structure features in five calyptratae flies of Diptera.

Parameter	*A. grahami*	*L. cuprina*	*G. morsitans*	*M. domestica*	*Ph. regina* (♀)
Sequencing platform	PacBio	Illumina	454/Illumina	Illumina	454/PacBio
Genome size (Mb)	600	458	366	692	550
No.of contigs/Scaffolds	1,604/7	74,043/4,436	-/13,807	-/20487	192,662/-
Contig N50 (kb)	1930	744.4	50	12	7.9
GC level (%)	31	29.3	34.1	35.1	26.2
Repetitive regions (%)	48.02	57.8	-	55	8.11
Function annotation (gene number; %)	12,791; 99.8	12,160; 83.6	12,308; 99.5	14180;92.3	7792;94
Sequencing depth	86×	100×	160×	90×	44×
Completeness (BUSCO/CEGMA; %)	99.2	96	99	98	93.6

Genome completeness was assessed by BUSCO or CEGMA. Four genomes of calyptratae fly species were selected, as *L. cuprina* [55], *G. morsitans* [56], *M. domestica* [57] and *Ph. regina* [45]. The genome version of *Ph. regina* female adult was chosen.

For the *A. grahami* genome, the assembly genome size (600 Mb) was almost same to the genome size (582.63 Mb) estimated in 17-mer analysis. The GC content and coverage of the sequencing depth of the genome assembly were analyzed by the GC Depth analyses to check the sequencing quality and to remove potentially contaminated contigs from other species. The completeness of the assembly was evaluated by BUSCO (v3.0, RRID: SCR 015008). The result of BUSCO analysis supported that our assembly covered 99.2% complete and 0.7% partial insect BUSCOs, with only 0.5% missed (Table S4).

We also performed flow cytometry with propidium iodide staining to estimate the genome size of *A. grahami*. Fruit fly was used as the internal control with DNA content (pictogram: pg, 1 pg = 978 Mb) of 1C = 0.18 pg [58]. The samples were

prepared following the procedure of the previous study [59]. The flow-cytometry was performed using Accuri C6 (BD, USA) with a 488nm laser. Data were processed by FlowJo software (v7.6) (Fig. S2). The estimated genome sizes of male (667.5 ± 6.013 Mb, N = 6) and female (682.5 ± 13.64 Mb, N = 6) have no significant difference (P-value = 0.3388). No sexual dimorphism was found. But it is about 15.9% larger than the K-mer based genome size (582.63 Mb), 12.5% larger than the assembly genome size (600.09 MB).

Functional Prediction and Genome Annotation

Analysis of repeat genes

Simple sequence repeats (SSRs) are a sort of short repeat sequences about 1-6 base pairs wildly distributed in genomes. SSRs in the blow fly genome were identified using the MIcroSAtellite Identification Tool (MISA, RRID: SCR 010765) [60]. MISA can distinguish and locate simple and complicated SSRs. The latter is always inserted by a certain number of nucleic acid bases. In total, 322266 SSRs were found in the *A. grahami* genome.

We also analyzed the tandem repeats and transposable elements (TEs) of genome repetitive sequences in *A. grahami*. A Tandem Repeats Finder (TRF, v4.09) approach was used to annotate the tandem repeats [61]. A combination of *de novo*-based and homology-based strategy was utilized in DNA and protein levels to identify TEs. Firstly we used RepeatModeler (v1.0.8, RRID: SCR 015027) [62] to construct a *de novo* repeat DNA library, where a repeat consensus data set including classification information was constructed. The potential TEs assembly was searched in both the *de novo* based repeat library and a custom TE library (Repbase 23.08) using RepeatMasker (v4.0.6, RRID: SCR 012954) [62]. RepeatProteinMask which

embedded in the RepeatMasker package was used to compare the transposable element protein with WU_BLASTX engine.

Overall, repetitive sequences accounted for 48.02% of the *A. grahami* genome, and 43.69% repetitive sequences were TEs. Repeat sequence of DNA consisted 11.65% of the *A. grahami* genome as the most abundant repeat class (Table 2).

Table 2 Statistics of repeat sequence analyses

Type	RepeatMasker		LTR finder		RepeatProteinMask		RepeatModeler		Combined TEs	
	Length (Mb)	% in genome	Length (Mb)	% in genome	Length (Mb)	% in genome	Length (Mb)	% in genome	Length (Mb)	% in genome
DNA	42174497	7.03	0	0	41341346	6.89	50464704	8.41	69933653	11.65
LINE	10505716	1.75	0	0	19838372	3.31	26169690	4.36	34333817	5.72
LTR	4789075	0.8	15966332	2.66	5778730	0.96	1229900	0.2	21249831	3.54
SINE	51914	0.01	0	0	0	0	453547	0.08	446000	0.07
Other*	12169475	2.02	0	0	7698698	1.28	50424873	8.4	78096062	13.02
Unknown*	161876	0.03	0	0	0	0	50424873	16	84103655	14.02
Total	69852553	11.64	15966332	2.66	74657146	12.44	224757686	37.45	288163018	48.02

***Other is sequences with annotation but not belonging to the above sorts of repetitive genes, such as satellites, simple repeats and etc.; unknown represents sequences cannot be classified.**

Gene prediction and functional annotation

A *de novo* based, homolog-based and RNAseq-based gene prediction methods were used to identify protein-coding genes in the *A. grahami* genome assembly together. Augustus (v2.4, RRID: SCR 008417) [63], GlimmerHMM (v3.0.4, RRID: SCR 002654) [64], Genemark (RRID: SCR 011930) [65] and SNAP (RRID: SCR 002127) [66] were used to the *de novo*-based gene prediction with default parameters. All of these software packages were trained using the *D. melanogaster* gene model before gene prediction [67]. GeMoMa (v1.3.1) was used to annotate genes referring *D. melanogaster*, *Glossina austeni*, *Lucilia cuprina*, *Stomoxys calcitrans* and *Musca domestica* from GenBank (Table S5) [68]. PASA (v2.0.2, RRID: SCR 014656) were

used to perform the RNAseq-based gene prediction [69]. Finally, the results from the three approaches were integrated using EVidenceModeler (EVM) (v1.1.1, RRID: SCR 014659) [69]. When conducting the EVM integration, PASA-predicted transcripts from unigenes and GeMoMa-predicted homologous transcripts were given higher weights than the *de novo* predicted transcripts. The gene set was aligned to the transposon database by TransposonPSI (v08222010) with default parameters to remove genes containing transposon [70]. Genes of transposon homologies were removed from the final gene assemblage. In total, 12823 protein-coding genes were identified in *A. grahami* genome, with an average of 13240.43 bp in length, 4.62 exons per gene (Table S6).

Gene functions of the predicted protein coding genes were annotated by two strategies. First, those predicted protein sequences were aligned to Swiss-Prot and TrEMBL protein databases using Blastall under the best match parameter [71]. The pathways of predicted sequences were extracted from the KEGG Automatic Annotation Server (v2.1) [72]. Then the annotation of motifs and domains were obtained by searching opening databases of InterPro with InterProScan (v5.24, RRID: SCR 005829).These databases includes Pfam (32.0, RRID: SCR 004726), ProDom (v2006.1, RRID: SCR 006969), PRINTS (v42.0, RRID: SCR 003412), PANTHER (v12.0, RRID: SCR 004869), SMRT (v7.1), and PROSITE (v2018_02, RRID: SCR 003457) [73, 74]. The two parts of results were combined to form the final data set. In summary, 12791 genes were annotated with at least 1 related function, which accounted for about 99.8% of predicated protein-coding genes (12823) of *A. grahami* (Table 3). Additionally, the annotation of non-coding RNA genes set was also performed based on the RNA-seq data of *A. grahami* transcriptome data (6.6G). The data set was aligned to the non-coding database Rfam (v14.0, RRID: SCR 007891) to

annotate genes of rRNA, snRNA and miRNA firstly. Then tRNA sequence was predicted using tRNAscan-SE (v2.0, RRID: SCR 010835) [75]. The rRNA and subunits were predicted by RNAmmer (v1.2) [76]. Finally, a total of 126 miRNAs, 21 rRNAs, 192 snRNAs and 859 tRNAs genes were annotated (Table S7).

Table 3 Function annotation of protein coding genes of *A. grahami*

	**Type**	**Number**	**Percent (%)**
**Annotation**	Swiss-Prot	9648	75.2
	TrEMBL	12721	99.2
	KEGG	5247	40.9
	KOG	8252	64.4
	GO	7518	58.6
	InterProScan	10488	81.8
	Nr*	12780	99.7
**Total**	Annotated	12791	99.8
	Gene	12823	-

*Nr: Non-Redundant Protein Sequence Database

Evolutionary analyses

Gene family and phylogenetic analyses

The species selected for gene family prediction was based on genomic models, classification background, feeding habits or life styles like *necrophagias*, polyphagia, *parasitism* or *hematophagia*. The genomic resource of *D. melanogaster, Lucilia cuprina*, *Musca domestica*, *Stomoxys calcitrans*, *Glossina austeni*, *Onthophagus taurus*, *Nicrophorus vespilloides*, *Blattella germanica*, *Cimex lectularius*, *Aedes aegypti* was used (Table S5) [67, 77-85]. The OrthoMCL (RRID: SCR 007839) was employed to predict gene family [86]. Firstly, the amino acid sequence of the longest transcript of each gene was selected from *A. grahami* and the selected insect species,

then aligned reciprocally with the BLASTP (RRID: SCR 001010) plug-in on NCBI with an e-value threshold of $1e^{-5}$. Then, the alignment results were clustered into family groups with default parameters. Finally, the orthologous gene families from each selected species were identified (Fig. 2). According to the results, *A. grahami* genome contents the fewest unique genes and gene families compared to other 10 species used in the analysis (Table 4).

Figure 2. Gene family comparison between *A. grahami* and other insect species

In total, 2989 single-copy gene families conserved in these 11 species were extracted. Each gene family was aligned in the amino-acid level using the MAFFT program (v7) firstly [87]. All the sequence alignments were then reversely translated to nucleotide alignments, and regions with low sequencing quality were filtered out by Gblocks (v0.91, RRID: SCR 015945) subsequently. Then, RAxML (v8.2.11, RRID: SCR 006086) was used to build phylogenetic trees with a GTR+GAMMA model for nucleotide sequences [88]. A total of 100 bootstrap replicates were employed to assess branch reliability in RAxML. *C. lectularius* was set as outgroup.

Table 4 Genome families of *A. grahami* and other insect species

Species	Genes Number	Genes number in families	Unclustered genes number	Family number	Unique Families number	Average genes number per family
A.aegypti	14,539	12,790	1,749	8,703	484	1.47
A.grahami	12,823	11,985	838	10,413	56	1.15
B.germanica	28,670	19,219	9,451	9,424	1,286	2.04
C.lectularius	11,890	9,718	2,172	8,104	250	1.2
D.melanogaster	13,872	11,442	2,430	9,707	237	1.18
G.austeni	19,722	12,012	7,710	9,794	355	1.23
L.cuprina	15,232	13,872	1,360	11,339	586	1.22
M.domestica	14,236	12,914	1,322	10,718	141	1.2
N.vespilloides	12,385	10,933	1,452	8,971	168	1.22
O.taurus	14,374	12,656	1,718	9,227	371	1.37

S. calcitrans	13,469	12,371	1,098	10,473	117	1.18

Unclustered genes and unique families represent the specific genes and families corresponding to each species.

In addition, we separated 9 selected species into different groups based on their diet habits like necrophagia, coprophagia, hematophagia and polyphagia (Table S8). Orthologous genes of each species were also separated as a single assemblage. The intersection of orthologous genes of *A. grahami* with those Diptera species and other non-Diptera species were calculated respectively using online Draw Venn Diagram [89]. These results may provide candidate genes for future research on the necrophagous life-style of *A. grahami* (Fig. 3).

Figure 3. Venn diagram of orthologous gene families. (A) The intersection between *A. grahami* and other Diptera species. (B) The intersection between *A. grahami* and other non-Diptera species of different diet habits.

Divergence time and gene family expansion / contraction

The estimation of divergence time was based on the results of the gene family clustering. Four-fold degenerate sites were extracted from the alignment of coding sequences of 2989 identified single-copy gene family. The PAML mcmctree program (v4.5, RRID: SCR 014932) was used to determine divergence times with the calculation of approximate likelihood test, molecular clock and substitution model of REV [90]. The primary parameters of mcmctree were set as clock = 2， RootAge = < 4， model = 7, BDparas = 110， kappa_gamma = 62， alpha_gamma = 11， rgene_gamma = 23.606， sigma2_gamma = 11.03. Calibrations of fossil evidence were retrieved from the TimeTree database to infer the evolutionary timescale [91].

In the phylogenetic analysis, *A. grahami* was clustered with *L. cuprina*, and

further clustered into the branch of Calliphoridae which next to Muscidae represented by *M. domestica, S. calcitrans*. This result was consistent with the blow fly species taxonomy. *A. grahami* diverged from the common ancestor with *L. cuprina* around 26 million years ago (Fig. 4).

Figure 4. **The estimation on divergence times. The numbers besides the dots of topological branches are the divergent time to present-day (Million years ago, Mya). Red dots represent the calibration time from fossil evidence. The right lists each family name.**

To further explore the gene family change under natural selecting pressure, the gene family expanding and contracting analyses were performed using CAFE program (RRID:SCR 005983) [92]. The result indicated that the expansion and contraction were happened in 102 and 280 gene families in *A. grahami* genome respectively. Additionally 198 gene families were lost from the genome (Table S9, Fig. S3).

Analysis of whole-genome duplication (WGD)

We used four-fold synonymous third-codon transversion (4DTv) estimation [93] and Ks (a measure of the substitutions per synonymous site) distribution [94] to detect WGD events in *A. grahami* genome. To this end, paralogous sequences of *A. grahami*, *Bombyx mori* and *D. melanogaster*, were identified with OrthoMCL [86]. Then, protein sequences of these insects were aligned mutually with BLASTP (using an E-value threshold of $\leq 1e^{-5}$) to identify conserved paralogs in each species. Finally, potential WGD events in each genome were evaluated based on their 4DTv and Ks distribution. The WGD analysis suggested that *A. grahami* experienced the same recent WGD events as *B. mori* (Fig. S4).

Chromosome assembly using Hi-C data

To generate a chromosomal level assembly of the genome, we took advantage of sequencing data from the Hi-C library. The Hi-C library was sequenced on the Illumina NanoSeq 6000 (Illumina, CA, USA), generating 495 million Hi-C read pairs. After filtering out low-quality sequences (quality scores ≤ 15), adaptor sequences, sequences shorter than 30 bp by fastp (v0.12.6, RRID: SCR 016962) [95] and the clean paired-end reads were mapped to the draft sequence assembled by bowtie2 (v.2.3.2, RRID: SCR 005476) [96] to get the unique mapped paired-end reads. As a result, 102 million uniquely mapped paired-end reads were generated, of which 62.26% were valid interaction pairs validated (Table S10). Combined with the valid Hi-C data, we subsequently employed the LACHESIS *de novo* assembly pipeline to produce chromosome-level scaffolds. As shown in Figure 5, the assembled sequence was anchored onto the 6 pseudo-chromosomes with lengths ranging from 57.97 Mb to 112.16 Mb (Table S11). The assembled pseudo-chromosomes (578,212,361 bp) with 104.65 Mb N50 scaffold accounted for 96.4% of the total contig bases (600,090,062 bp) (Table S3).

Figure 5. Hi-C interaction matrix maps within and among 6 chromosomes. The contact density was illustrated by the color bar with red (high density) to white (low density).

The similarity between *A. grahami* genome and the published fruit fly (*D. melanogaster*) genome was analyzed [67]. The protein-coding genes from each

genome was aligned using BLASTP with an E-value threshold of $1e^{-10}$, and then the blast results combined the GFF format files of the two genomes were performed using MCScanX [97].

The collinearity between *A. grahami* and *D. melanogaster* genome was shown in Fig.6A. The pseudochromosomes of *A. grahami* and corresponding Muller elements of *D. melanogaster* were listed (Table S11). For the sex chromosome, since Muller A (chromosome X of fruit fly) was uniquely located on AgChr03 of *A. grahami*, thus we expect it should represent the sex chromosome of *A. grahami*.

In addition, we investigated the distributions of long terminal repeat (LTR), gene family expansion or contraction, and genes under positive selection, et al. on the genome using a window size of 1 Mb across each chromosome and plotted the distributions in Fig. 6B by Circos (RRID: SCR 011798). We haven't found gene number enrichment for any particular chromosomes - all the chromosomes contain a gene density of around 20 genes/per Mb. However, the results showed that longer chromosomes tend to contain higher number of LRT, except for the case of Chr05. In addition, we noticed that the LTR enriched on specific regions of each chromosome where could present the centromere locations (Table S11).

Figure 6. Collinerity and gene clustering of *A. grahami* genome. (A) Collinear relationship between *A. grahami* and *D. melanogaster* genomes. The blue bar represents *A. grahami* genome and the grey one represents fruit fly genome. (B) Gene density distribution on chromosomes of *A. grahami*. The outer blue circle indicates the chromosomes. The inner yellow, light blue, green and orange circle represent the LTR, expanded gene family, contracted gene family and positive selected gene respectively. Window size = 1 Mb.

Conclusion

Using long reads *de novo* technology of PacBio Sequel sequencing platform and Hi-C sequencing technology, we successfully assembled the robust draft genome of *A.*

grahami. This reference genome is the first chromosome-level genome assembly in calyptratae. It will facilitate further genomic research of other fly species of forensic importance, and promote the transition from forensic genetics to forensic genomics [48]. This draft genome resource will be a helpful tool for advancing the study on the evolution of *A. grahami* genome. It will deepen our understanding to *A. grahami*'s unique biological characteristics, such as low-temperature tolerance, seasonal distribution, necrophagous diet habit, and how this Asia indigenous species intrude into other regions of the world. Forensic entomological studies based on qualified genome resource will reinforce the reliability of entomological evidence and promote its application in the law-suit [98].

Availability of supporting data

Genome and transcriptom are available in the NCBI SRA database (project accession: **PRJNA513084, SRA: SRX5207346**).

Additional flies

Additional File Figure S1. 17-mer Depth Distribution Curve. The x-axis represents the k-mer depth; the y-axis represent k-mer depth frequency; *Arabidopsis thaliana* (Atha for short) was set as reference.

Additional File Figure S2. Estimation of genome size of *A. grahami* by flow cytometry. Genome size (bp) was calculated from DNA content (pg) following the formula: GAg= (FAg/FDm) × GDm. GAg, DNA content of *A. grahami*, GDm, DNA content of *D. melanogaster*, FAg, fluorescence value of *A. grahami*, FDm, fluorescence value of *D. melanogaster*.

Additional File Figure S3. Expansion and contraction at the gene family level.

Branch length represents divergent time; Pie chart illustrates percentage of expansion and contraction; '+/-'means gene gain / loss.

Additional File Figure S4. Whole-genome duplication analysis of *A. grahami*, *B. mori* and *D. melanogaster*.

Additional File Table S1. Information of sequencing platform and output data.

Additional File Table S2. Genome size estimation and Heterozygosity based on 17 k-mer.

Additional File Table S3. Statistics results of genome assembly correction.

Additional File Table S4. Assessment on assembly completeness.

Additional File Table S5. Genome resource of 10 insect species for comparable genomics analysis.

Additional File Table S6. Comparison of *A. grahami* and other fly species on protein coding genes structure and statistics.

Additional File Table S7. Functional annotation of non-coding RNA genes.

Additional File Table S8. Diet habit of 9 selected insect species.

Additional File Table S9. Statistics of gene family expansion and contraction

Additional File Table S10. Statistics of the Hi-C assembly of the *A. grahami* genome.

Additional File Table S11. Genome-wide characteristic on pseudochromosomes of the *A. grahami*.

Abbreviations

PMImin: minimum postmortem interval; *COI*: cytochrome oxidase gene I; CTAB: Cetyltrimethyl Ammonium Bromide; WGS: Whole-genome shotgun

sequencing; Hi-C: Chromosome conformation capture; SSR: Simple sequence repeats; TEs: Transposable elements; TRF: Tandem Repeats Finder; LTR: long terminal repeat; Mya: Million years ago; 4DTv: four-fold synonymous third-codon transversion; BUSCO: benchmarking universal single-copy orthologs; GO: gene ontology; KEGG: Kyoto Encyclopedia of Genes and Genomes; SMRT: single-molecule real time; WGD: Whole-genome duplication

Competing interests

All authors declare that no competing interests.

Funding

The present study was supported by grant of the National Natural Science Foundation of China (81571855) and Science Foundation of Hunan Province (2017SK2015).

Author contributions

F. M., J.C. designed the project. F. M., M. Z., Y. W., and C.C. analyzed the data. H.H., Z. L., Y. J. prepared the samples and conducted the experiments. F. M., D.F., Z. S., wrote and revised the manuscript. J.C. supervised the whole program and coordinated the group. Y. G. provided material and equipment for breeding of insects.

Reference

1. Catts EP, Goff ML. Forensic entomology in criminal investigations. Annual review of entomology. 1992;37:253-72. doi:10.1146/annurev.en.37.010192.001345.
2. Benecke M. A brief history of forensic entomology. Forensic science international. 2001;120 1-2:2-14.

3. Schoenly KA. statistical analysis of successional patterns in carrion-arthropod assemblages: implications for forensic entomology and determination of the postmortem interval. Journal of forensic sciences. 1992;37 6:1489-513.
4. Tomberlin JK, Mohr R, Benbow ME, Tarone AM, VanLaerhoven S. A roadmap for bridging basic and applied research in forensic entomology. Annual review of entomology. 2011;56:401-21. doi:10.1146/annurev-ento-051710-103143.
5. Benecke M, Lessig R. Child neglect and forensic entomology. Forensic science international. 2001;120 1-2:155-9.
6. Campobasso CP, Gherardi M, Caligara M, Sironi L, Introna F. Drug analysis in blowfly larvae and in human tissues: a comparative study. International journal of legal medicine. 2004;118 4:210-4. doi:10.1007/s00414-004-0448-1.
7. Castner LC, Byrd JH. Insects of Forensic Importance. In: Castner LC, Byrd JH, editor. Forensic entomology : the utility of arthropods in legal investigations. Boca Raton, London: CRC Press; 2009. p. 44-6.
8. Anderson GS. Minimum and maximum development rates of some forensically important Calliphoridae (Diptera). Journal of forensic sciences. 2000;45 4:824-32.
9. Harvey ML, Gaudieri S, Villet MH, Dadour IR. A global study of forensically significant calliphorids: Implications for identification. Forensic science international. 2008;177 1:66-76. doi:10.1016/j.forsciint.2007.10.009.
10. Norris KR. The Bionomics of Blow Flies. Annreventomol. 1965;10 1:47-68.
11. Baumgartner DL, Greenberg B. The Genus Chrysomya (Diptera: Calliphoridae) in the New World. Journal of medical entomology. 1984;21 1:105-13.
12. Tarone AM, Sanford MR. Is PMI the Hypothesis or the Null Hypothesis? Journal of medical entomology. 2017;54 5:1109-15. doi:10.1093/jme/tjx119.
13. Tarone AM, Picard CJ, Spiegelman C, Foran DR. Population and temperature effects on Lucilia sericata (Diptera: Calliphoridae) body size and minimum development time. Journal of medical entomology. 2011;48 5:1062-8.
14. Zhao B, Wen C, Qi LL, Wang H, Wang J. [Biological characteristics of calliphoridae and its application in forensic medicine]. Fa yi xue za zhi. 2013;29 6:447-50.
15. Fan ZD. Key to the common flies of China. Beijing, China: Science publishing house; 1992.
16. Aldrich JM. New two-winged flies of the family Calliphoridae from China. Proceedings of the United States National Museum. 1930.
17. Dodge HR. Identifying common flies. Public health reports. 1953;68 3:345-50.
18. Nunez-Vazquez C, Tomberlin J, Garcia-Martinez O. First Record of the Blow Fly Calliphora grahami from Mexico. Southwestern Entomologist. 2010;35 3:313-6. doi:Doi 10.3958/059.035.0310.
19. Whitworth T. Keys to the genera and species of blow flies (Diptera : Calliphoridae) of America North of Mexico. P Entomol Soc Wash. 2006;108 3:689-725.
20. Wang Y, Zhang YN, Liu C, Hu GL, Wang M, Yang LJ, et al. Development of Aldrichina grahami (Diptera: Calliphoridae) at Constant Temperatures. Journal of medical entomology. 2018;55 6:1402-9. doi:10.1093/jme/tjy128.
21. Chen W, Yang L, Ren L, Shang Y, Wang S, Guo Y. Impact of Constant Versus Fluctuating Temperatures on the Development and Life History Parameters of Aldrichina grahami (Diptera: Calliphoridae). Insects. 2019;10 7 doi:10.3390/insects10070184.

22. Kurahashi H, Kawai S, Shudo C, Wada Y. Seasonal prevalence of adult fly and life cycle of aldrichina grahami (aldrich) in tokyo. Medical Entomology & Zoology. 1984;35 3:261-7.

23. Guo YD, Cai JF, Tang ZC, Feng XO, Lin Z, Yong F, et al. Application of Aldrichina grahami (Diptera, Calliphoridae) for forensic investigation in central-south China. Romanian Journal Of Legal Medicine. 2011;19 1:55-8. doi:10.4323/rjlm.2011.55.

24. Wang JF, Hu C, Min JX, Chen YC, Li JT. Chronometrical morphology of Aldrichina grahami and its application in the determination of postmortem interval. Acta Entomol Sin. 2002;45 2002:265-70.

25. Kurahashi H, Kawai S, Shudo C. Seasonal migration of Japanese blow flies, Aldrichina grahami (Aldrich) and Calliphora nigribarbis Vollenhoven, observed by a mark and recapture method on Hachijo Island, Tokyo. Medical Entomology & Zoology. 1991; 42:57-9.

26. Zehner R, Amendt J, Krettek R. STR typing of human DNA from fly larvae fed on decomposing bodies. Journal of forensic sciences. 2004;49 2:337-40.

27. Li K, Ye GY, Zhu JY, Hu C. Detection of food source by PCR analysis of the gut contents of Aldrichina grahami (Aldrich) (Diptera: Calliphoridae) during post-feeding period. Insect Sci. 2007;14 1:47-52. doi:10.1111/j.1744-7917.2007.00124.x.

28. Xu H, Ye GY, Xu Y, Hu C, Zhu GH. Age-dependent changes in cuticular hydrocarbons of larvae in Aldrichina grahami (Aldrich) (Diptera: Calliphoridae). Forensic science international. 2014;242:236-41. doi:10.1016/j.forsciint.2014.07.003.

29. Moore HE, Adam CD, Drijfhout FP. Potential Use of Hydrocarbons for Aging Lucilia sericata Blowfly Larvae to Establish the Postmortem Interval. Journal of forensic sciences. 2013;58 2:404-12. doi:10.1111/1556-4029.12016.

30. Liu YL. A case report of gastrointestinal myiasis caused by Aldrichina grahami. Acta Medicinae Universitatis Scientiae et Technologiae Huazhong. 1980;2:81-2.

31. Li XL, Xu ZQ. A case of human gastrointestinal myiasis. Bulletin of Disease Control & Prevention. 2006;21 1:107.

32. Cao XL, Sang YH, Yang YL, Wang S. Comprehensive analyses on Chinese human myiasis cases of 2003-2013. Guide of China Medicine. 2015;8:37-9.

33. Lachish T, Marhoom E, Mumcuoglu KY, Tandlich M, Schwartz E. Myiasis in Travelers. Journal of travel medicine. 2015;22 4:232-6. doi:10.1111/jtm.12203.

34. Sawabe K, Hoshino K, Isawa H, Sasaki T, Hayashi T, Tsuda Y, et al. Detection and isolation of highly pathogenic H5N1 avian influenza A viruses from blow flies collected in the vicinity of an infected poultry farm in Kyoto, Japan, 2004. The American journal of tropical medicine and hygiene. 2006;75 2:327-32.

35. Miura K, Takaya T, Koshiba K. The effect of biotin deficiency on the biosynthesis of the fatty acids in a blowfly, Aldrichina grahami during metamorphosis under aseptic conditions. Archives internationales de physiologie et de biochimie. 1967;75 1:65-76.

36. Tohoru H, Akira W, Kazuo Miura. Properties and regulation of xanthine dehydrogenase of a blowfly, Aldrichina grahami. Insect Biochemistry, 1977, 7(4):317-322. 1977;7 4:317-22.

37. Wadano A MK. Urate oxidase in the blowfly, Aldrichina grahami. Insect Biochemistry. 1976;6 3:321-5.

38. Wadano A, Miura, K, Ihara, H, Kondo N, Taniguchi, MA. Purification and some properties of isocitrate dehydrogenase of a blowfly aldrichina grahami. Comparative Biochemistry and Physiology B. 1989;94 1:189-94.

39. Meng FM, Ren LP, Wang Z, Deng J, Guo YD, Chen C, et al. Identification of Forensically Important Blow Flies (Diptera: Calliphoridae) in China Based on COI. Journal of medical entomology. 2017;54 5:1193-200. doi:10.1093/jme/tjx105.
40. Zaidi F, Wei SJ, Shi M, Chen XX. Utility of multi-gene loci for forensic species diagnosis of blowflies. Journal of insect science. 2011;11.
41. Park SH, Park CH, Zhang Y, Piao H, Chung U, Kim SY, et al. Using the Developmental Gene Bicoid to Identify Species of Forensically Important Blowflies (Diptera: Calliphoridae). BioMed research international. 2013; doi:Artn 53805110.1155/2013/538051.
42. Zhu ZY, Liao HD, Ling J, Guo YD, Cai JF, Ding YJ. The complete mitochondria genome of Aldrichina grahami (Diptera: Calliphoridae). Mitochondrial DNA B. 2016;1:107-9. doi:10.1080/23802359.2015.1137847.
43. Gallagher MB, Sandhu S, Kimsey R. Variation in developmental time for geographically distinct populations of the common green bottle fly, Lucilia sericata (Meigen). Journal of forensic sciences. 2010;55 2:438-42. doi:10.1111/j.1556-4029.2009.01285.x.
44. Hu Y, Yuan X, Zhu F, Lei C. Development time and size-related traits in the oriental blowfly, chrysomya megacephala along a latitudinal gradient from china. Journal of Thermal Biology. 2010;35 7:366-71.
45. Andere A, Platt RN, Ray DA and Picard CJ. Genome sequence of Phormia regina Meigen (Diptera: Calliphoridae): implications for medical, veterinary and forensic research. BMC genomics. 2016;17 1:842. doi:10.1186/s12864-016-3187-z.
46. Zajac BK, Amendt J, Verhoff MA, Zehner R. Dating Pupae of the Blow Fly Calliphora vicina Robineau-Desvoidy 1830 (Diptera: Calliphoridae) for Post Mortem Interval-Estimation: Validation of Molecular Age Markers. Genes. 2018;9 3 doi:Artn 15310.3390/Genes9030153.
47. Arenas M, Pereira F, Oliveira M, Pinto N, Lopes AM, Gomes V, et al. Forensic genetics and genomics: Much more than just a human affair. PLoS genetics. 2017;13 9:e1006960. doi:10.1371/journal.pgen.1006960.
48. Kayser M, Parson W. Transitioning from Forensic Genetics to Forensic Genomics. Genes. 2017;9 1 doi:10.3390/genes9010003.
49. Rao SSP, Huntley MH, Durand NC, Stamenova EK, Bochkov ID, Robinson JT, et al. A 3D Map of the Human Genome at Kilobase Resolution Reveals Principles of Chromatin Looping. Cell. 2014;159 7:1665-80. doi:10.1016/j.cell.2014.11.021.
50. Marcais G, Kingsford C. A fast, lock-free approach for efficient parallel counting of occurrences of k-mers. Bioinformatics. 2011;27 6:764-70. doi:10.1093/bioinformatics/btr011.
51. Eid J, Fehr A, Gray J, Luong K, Lyle J, Otto G, et al. Real-time DNA sequencing from single polymerase molecules. Science. 2009;323 5910:133-8. doi:10.1126/science.1162986.
52. WTDBG package：https://github.com/ruanjue/wtdbg. (Accessed 10 Jan 2018).
53. Falcon: https://github.com/PacificBiosciences/FALCON.
54. Walker BJ, Abeel T, Shea T, Priest M, Abouelliel A, Sakthikumar S, et al. Pilon: an integrated tool for comprehensive microbial variant detection and genome assembly improvement. PloS one. 2014;9 11:e112963. doi:10.1371/journal.pone.0112963.
55. Anstead CA, Korhonen PK, Young ND, Hall RS, Jex AR, Murali SC, et al. Lucilia cuprina genome unlocks parasitic fly biology to underpin future interventions. Nature communications. 2015;6:7344. doi:10.1038/ncomms8344.
56. Watanabe J, Hattori M, Berriman M, Lehane MJ, Hall N, Solano P, et al. Genome sequence of

the tsetse fly (Glossina morsitans): vector of African trypanosomiasis. Science. 2014;344 6182:380-6. doi:10.1126/science.1249656.

57. Scott JG, Warren WC, Beukeboom LW, Bopp D, Clark AG, Giers SD, et al. Genome of the house fly, Musca domestica L., a global vector of diseases with adaptations to a septic environment. Genome biology. 2014;15 10:466. doi:10.1186/s13059-014-0466-3.

58. Bennett MD, Leitch IJ, Price HJ, Johnston JS. Comparisons with Caenorhabditis (approximately 100 Mb) and Drosophila (approximately 175 Mb) using flow cytometry show genome size in Arabidopsis to be approximately 157 Mb and thus approximately 25% larger than the Arabidopsis genome initiative estimate of approximately 125 Mb. Annals of botany. 2003;91 5:547-57. doi:10.1093/aob/mcg057.

59. Picard CJ, Johnston JS, Tarone AM. Genome sizes of forensically relevant Diptera. Journal of medical entomology. 2012;49 1:192-7. doi:10.1603/me11075.

60. Thiel T, Michalek W, Varshney RK, Graner A. Exploiting EST databases for the development and characterization of gene-derived SSR-markers in barley (Hordeum vulgare L.). TAG Theoretical and applied genetics Theoretische und angewandte Genetik. 2003;106 3:411-22. doi:10.1007/s00122-002-1031-0.

61. Benson G. Tandem repeats finder: a program to analyze DNA sequences. Nucleic acids research. 1999;27 2:573-80. doi:10.1093/nar/27.2.573.

62. Bedell JA, Korf I, Gish W. MaskerAid: a performance enhancement to RepeatMasker. Bioinformatics. 2000;16 11:1040-1. doi:10.1093/bioinformatics/16.11.1040.

63. Stanke M, Waack S. Gene prediction with a hidden Markov model and a new intron submodel. Bioinformatics. 2003;19 Suppl 2:ii215-25. doi:10.1093/bioinformatics/btg1080.

64. Majoros WH, Pertea M, Salzberg SL. TigrScan and GlimmerHMM: two open source ab initio eukaryotic gene-finders. Bioinformatics. 2004;20 16:2878-9. doi:10.1093/bioinformatics/bth315.

65. Besemer J, Borodovsky M. GeneMark: web software for gene finding in prokaryotes, eukaryotes and viruses. Nucleic acids research. 2005;33 Web Server issue:W451-4. doi:10.1093/nar/gki487.

66. Johnson AD, Handsaker RE, Pulit SL, Nizzari MM, O'Donnell CJ, De Bakker PI. SNAP: a web-based tool for identification and annotation of proxy SNPs using HapMap. Bioinformatics. 2008;24 24:2938-9. doi:10.1093/bioinformatics/btn564.

67. NCBI Genome. https://www.ncbi.nlm.nih.gov/genome/47.

68. Keilwagen J, Wenk M, Erickson JL, Schattat MH, Grau J, Hartung F. Using intron position conservation for homology-based gene prediction. Nucleic acids research. 2016;44 9 doi:ARTN e8910.1093/nar/gkw092.

69. Haas BJ, Salzberg SL, Zhu W, Pertea M, Allen JE, Orvis J, et al. Automated eukaryotic gene structure annotation using EVidenceModeler and the program to assemble spliced alignments. Genome biology. 2008;9 1 doi:Artn R710.1186/Gb-2008-9-1-R7.

70. Yagi M, Kosugi S, Hirakawa H, Ohmiya A, Tanase K, Harada T, et al. Sequence Analysis of the Genome of Carnation (Dianthus caryophyllus L.). DNA Research. 2014;21 3:231-41. doi:10.1093/dnares/dst053.

71. Bairoch A, Apweiler R, Wu CH, Barker WC, Boeckmann B, Ferro S, et al. The universal protein resource (UniProt). Nucleic acids research. 2005;33:D154-D9. doi:10.1093/nar/gki070.

72. Kanehisa M, Goto S. KEGG: Kyoto Encyclopedia of Genes and Genomes. Nucleic acids

research. 2000;28 1:27-30. doi:Doi 10.1093/Nar/28.1.27.

73. Hunter S, Apweiler R, Attwood TK, Bairoch A, Bateman A, Binns D, et al. InterPro: the integrative protein signature database. Nucleic acids research. 2009;37:D211-D5. doi:10.1093/nar/gkn785.

74. Zdobnov EM, Apweiler R. InterProScan - an integration platform for the signature-recognition methods in InterPro. Bioinformatics. 2001;17 9:847-8. doi:DOI 10.1093/bioinformatics/17.9.847.

75. Lowe TM, Eddy SR. tRNAscan-SE: a program for improved detection of transfer RNA genes in genomic sequence. Nucleic acids research. 1997;25 5:955-64. doi:10.1093/nar/25.5.955.

76. Lagesen K, Hallin P, Rodland EA, Staerfeldt HH, Rognes T, Ussery DW. RNAmmer: consistent and rapid annotation of ribosomal RNA genes. Nucleic acids research. 2007;35 9:3100-8. doi:10.1093/nar/gkm160.

77. NCBI Genome. https://www.ncbi.nlm.nih.gov/genome/12732

78. NCBI Genome. https://www.ncbi.nlm.nih.gov/genome/14461.

79. NCBI Genome. https://www.ncbi.nlm.nih.gov/genome/11278

80. NCBI Genome. https://www.ncbi.nlm.nih.gov/genome/11279.

81. NCBI Genome. https://www.ncbi.nlm.nih.gov/genome/12827.

82. NCBI Genome. https://www.ncbi.nlm.nih.gov/genome/13223.

83. NCBI Genome. https://www.ncbi.nlm.nih.gov/genome/16689.

84. NCBI Genome. https://www.ncbi.nlm.nih.gov/genome/40824.

85. NCBI Genome. https://www.ncbi.nlm.nih.gov/genome/44.

86. Li L, Stoeckert CJ, Roos DS. OrthoMCL: Identification of ortholog groups for eukaryotic genomes. Genome research. 2003;13 9:2178-89. doi:10.1101/gr.1224503.

87. Katoh K, Standley DM. MAFFT multiple sequence alignment software version 7: improvements in performance and usability. Molecular biology and evolution. 2013;30 4:772-80. doi:10.1093/molbev/mst010.

88. Stamatakis A. RAxML-VI-HPC: Maximum likelihood-based phylogenetic analyses with thousands of taxa and mixed models. Bioinformatics. 2006;22 21:2688-90. doi:10.1093/bioinformatics/btl446.

89. Draw Venn Diagram. http://bioinformatics.psb.ugent.be/webtools/Venn/.

90. Yang Z. PAML: a program package for phylogenetic analysis by maximum likelihood. Computer applications in the biosciences : CABIOS. 1997;13 5:555-6.

91. Hedges SB, Dudley J, Kumar S. TimeTree: a public knowledge-base of divergence times among organisms. Bioinformatics. 2006;22 23:2971-2. doi:10.1093/bioinformatics/btl505.

92. De Bie T, Cristianini N, Demuth JP, Hahn MW. CAFE: a computational tool for the study of gene family evolution. Bioinformatics. 2006;22 10:1269-71. doi:10.1093/bioinformatics/btl097.

93. Kimura M. A simple method for estimating evolutionary rates of base substitutions through comparative studies of nucleotide sequences. Journal of molecular evolution. 1980;16 2:111-20.

94. Blanc G, Wolfe KH. Widespread paleopolyploidy in model plant species inferred from age distributions of duplicate genes. The Plant cell. 2004;16 7:1667-78. doi:10.1105/tpc.021345.

95. Chen S, Zhou Y, Chen Y, Gu J. fastp: an ultra-fast all-in-one FASTQ preprocessor. Bioinformatics. 2018;34 17:i884-i90. doi:10.1093/bioinformatics/bty560.

96. Langmead B, Salzberg SL. Fast gapped-read alignment with Bowtie 2. Nature methods. 2012;9 4:357-9. doi:10.1038/nmeth.1923.

97. Wang Y, Tang H, Debarry JD, Tan X, Li J, Wang X, et al. MCScanX: a toolkit for detection and evolutionary analysis of gene synteny and collinearity. Nucleic acids research. 2012;40 7:e49. doi:10.1093/nar/gkr1293.

98. Jager AC, Alvarez ML, Davis CP, Guzman E, Han Y, Way L, et al. Developmental validation of the MiSeq FGx Forensic Genomics System for Targeted Next Generation Sequencing in Forensic DNA Casework and Database Laboratories. Forensic science international Genetics. 2017;28:52-70. doi:10.1016/j.fsigen.2017.01.011.

Click here to access/download;Table;supplementary table 20190726.docx

Table S1. Information of sequencing platform and output data

Platform	Library type	Library number	Library size (bp)	Data size (Gb)
Illumina Hiseq Xten	Short insert size		400	46.05
PacBio Sequel	Long insert size	7	20000	50.15
Illumina NovaSeq 6000	Hi-C	2	150	74.24

Table S2. Genome size estimation and Heterozygosity based on 17 k-mer

Sample	k-mer	kmer_num	kmer_depth	Genome size (Mb)	Heterozygosity (%)
A. grahami	17	29,131,491,603	50	582.63	2.5

Table S3. Statistics of genome assembly

Stat Type	Contig Length	Contig Number	Scaffold Length	Scaffold Number*
N50	1,925,180	79	104,650,035	3
N60	1,455,152	114	104,176,436	4
N70	948,798	165	104,176,436	4
N80	616,470	242	/	/
N90	274,886	387	/	/
Longest	12,234,297	1	112,158,196	1
Total	600,090,062	1,604	600,249,762	7
Length>1kb	600,090,062	1,604	600,249,762	7
Length>2kb	600,090,062	1,604	600,249,762	7
Length>5kb	600,062,286	1,598	600,249,762	7

*The assembly based on results of Hi-C sequencing was also adds in this table to show the statistics of scaffolds.

Table S4. Assessment on assembly completeness

Type	Number	Percent (%)
Complete BUSCOs (C)	1,645	99.2
Complete and single-copy BUSCOs (S)	1,633	98.5
Complete and duplicated BUSCOs (D)	12	0.7
Fragmented BUSCOs (F)	5	0.3
Missing BUSCOs (M)	8	0.5
Total BUSCO groups searched	1,658	100

Table S5. Genome resource of 10 insect species for comparable genomics analysis.

Species list	de novo data link
Drosophila melanogaster	https://www.ncbi.nlm.nih.gov/genome/47
Lucilia cuprina	https://www.ncbi.nlm.nih.gov/genome/12732
Musca domestica	https://www.ncbi.nlm.nih.gov/genome/14461
Stomoxys calcitrans	https://www.ncbi.nlm.nih.gov/genome/11278
Aedes aegypti	https://www.ncbi.nlm.nih.gov/genome/44
Blattella germanica	https://www.ncbi.nlm.nih.gov/genome/13223
Cimex lectularius	https://www.ncbi.nlm.nih.gov/genome/11279
Glossina austeni	https://www.ncbi.nlm.nih.gov/genome/16689
Nicrophorus vespilloides	https://www.ncbi.nlm.nih.gov/genome/40824
Onthophagus taurus	https://www.ncbi.nlm.nih.gov/genome/12827

Table S6. Comparison of *A grahami* and other fly species on protein coding genes structure and statistics

Specie	Total number of gene	Average transcript length (bp)	Average CDS length (bp)	Average exons number per gene	Average exon length (bp)	Average intron length (bp)
A. grahami	12,823	13,240.43	1,669.56	4.62	361.16	3,193.92
D. melanogaster	13,918	4,597.06	1,560.59	3.9	400.29	1,047.56
G. austeni	19,725	5,058.51	1,243.50	4.37	284.4	1,131.27
L. cuprina	15,536	6,989.99	1,584.56	3.9	406.15	1,863.01
M. domestica	14,888	11,464.01	1,570.65	4.11	381.94	3,178.79
S. calcitrans	13,982	19,503.86	1,648.55	4.28	384.78	5,436.46

Table S7. Functional annotation of non-coding RNA genes

Type	Copy Number	Average Length (bp)	Total Length (bp)	Percentage (%) of Genome
rRNA	21	1473.43	30942	0.002613
18S	4	2443	9772	0.000825
28S	6	3237.67	19426	0.00164
5.8S	8	174.88	1399	0.000118
5S	3	115	345	0.000029
snRNA	192	134.86	25894	0.002186
CD-box	72	124.57	8969	0.000757
HACA-box	31	118.23	3665	0.000309
splicing	89	148.99	13260	0.00112
miRNA	126	87.22	10990	0.000928
tRNA	859	76.52	65731	0.00555

Table S8. Diet habit of 9 selected insect species

Order	Species name	Diet habit
Blattaria	*B. germanica*	polyphagia

Hemiptera	*C. lectularius*	hematophagia
Coleoptera	*N. vespilloides*	necrophagia
Coleoptera	*O. taurus*	coprophagia
Diptera	*S. calcitrans*	hematophagia
Diptera	*A.aegypti*	hematophagia
Diptera	*G. austeni*	hematophagia
Diptera	*L. cuprina*	necrophagia, facultative parasitism
Diptera	*M. domestica*	necrophagia, coprophagia

Table S10. Statistics of the Hi-C assembly of the *A. grahami* genome

Sample	Statistics
Clean Bases(bp)	71,001,944,196
Clean Paired-end Reads	243,128,013
Unique Mapped Paired-end Reads	101,626,567
Unique Mapped Ratio (%)	41.80

Valid Paired-end Reads	63,268,612
Valid Rate (%)	62.26

Table S9. Statistics

Branch	Branch Length	N
C.lectularius	385.792	6330
B.germanica	365.152	6243
D.melanogaster	132.919	6822
G.austeni	83.1975	6550
M.domestica	32.561	6719
S.calcitrans	32.561	6714
S.calcitrans|M.domestica	27.4242	6855
L.cuprina	27.7528	6817
A.grahami	27.7528	6680
A.grahami|L.cuprina	32.2324	6878
A.grahami,L.cuprina|S.calcitrans,M.domestica	23.2123	6878
A.grahami,L.cuprina,S.calcitrans,M.domestica|G.austeni	49.7217	6878
A.grahami,L.cuprina,S.calcitrans,M.domestica,G.austeni |D.melanogaster	108.651	6878
A.aegypti	241.57	6703
A.aegypti |A.grahami,L.cuprina,S.calcitrans,M.domestica,G.austeni,D.melanogaster	62.7756	6878
O.taurus	284.233	6677
N.vespilloides	284.233	6695
N.vespilloides |O.taurus	20.1125	6878
N.vespilloides,O.taurus |A.aegypti,A.grahami,L.cuprina,S.calcitrans, M.domestica,G.austeni,D.melanogaster	60.8059	6878
N.vespilloides,O.taurus,A.aegypti,A.grahami,L.cuprina,S.calcitrans, M.domestica,G.austeni,D.melanogaster |B.germanica	20.6402	6878
C.lectularius	385.792	6330
B.germanica	365.152	6243
D.melanogaster	132.919	6822
G.austeni	83.1975	6550

*** Branch, brances on evolutionary tree; N, number of gene family; Gene Gain/Family, average gene exp**

# of gene family expansion and contraction

Expansion			Contractions			Ext	
Families	Genes	Gene Gain / Family	Families	Genes	Gene Loss / Family	Families	Genes
236	329	1.39407	674	733	1.08754	548	582
369	513	1.39024	759	801	1.05534	635	652
201	257	1.27861	98	106	1.08163	56	59
484	574	1.18595	426	465	1.09155	328	334
284	340	1.19718	180	182	1.01111	136	138
154	200	1.2987	209	213	1.01914	141	141
66	97	1.4697	53	55	1.03774	23	23
179	217	1.21229	106	112	1.0566	61	63
102	152	1.4902	280	291	1.03929	198	206
32	37	1.15625	24	25	1.04167	0	0
51	64	1.2549	16	16	1	0	0
63	65	1.03175	20	41	2.05	0	0
86	98	1.13953	70	92	1.31429	0	0
589	1011	1.71647	222	227	1.02252	175	176
53	75	1.41509	51	61	1.19608	0	0
526	792	1.5057	276	291	1.05435	201	207
253	394	1.55731	283	319	1.12721	183	193
23	25	1.08696	3	3	1	0	0
22	28	1.27273	35	40	1.14286	0	0
0	0	NA	0	0	NA	0	0
236	329	1.39407	674	733	1.08754	548	582
369	513	1.39024	759	801	1.05534	635	652
201	257	1.27861	98	106	1.08163	56	59
484	574	1.18595	426	465	1.09155	328	334

panding in each family; Avg./Exp=(total genes gained along branch - total genes lost along branch)/to

inction		
Gene Extinct / Family	No change	Avg/Exp
1.06204	5968	-0.05874
1.02677	5750	-0.04187
1.05357	6579	0.021954
1.01829	5968	0.015848
1.01471	6391	0.022972
1	6492	0.189
1	6759	0.6106
1.03279	6593	0.015266
1.0404	6496	-0.0221
NA	6822	0.1745
NA	6811	0.6979
NA	6795	0.3489
NA	6722	0.872
1.00571	6067	0.113987
NA	6774	0.2035
1.02985	6076	0.072841
1.05464	6342	0.01904
NA	6852	0.3199
NA	6821	0.174
NA	6878	0
1.06204	5968	-0.05874
1.02677	5750	-0.04187
1.05357	6579	0.021954
1.01829	5968	0.015848

tal genes at ancestral node of branch.

Click here to access/download;Table;Table S11.xlsx

'able S11. Genome-wide characteristic on pseudoc

	Muller element of *D. melanogaster*	Length	Scaf Num	all genes	Density (/mb)	positively	Density (/mb)
Chr01	Muller B	112,158,196	226	2119	18.89	23	0.205068
Chr02	Muller D	109,743,127	180	2411	21.97	32	0.29159
Chr03	Muller A	104,650,035	162	2424	23.16	29	0.277114
Chr04	Muller C	104,176,436	114	2499	23.99	21	0.201581
Chr05	0	89,513,788	61	1804	20.15	16	0.178743
Chr06	Muller E	57,970,779	50	1305	22.51	10	0.172501

hromosomes of the *A. graham*

expansion	Density (/mb)	contraction	Density (/mb)	LTR	Density (/mb)
50	0.445799	21	0.187236	14529	129.5402
54	0.492058	18	0.164019	11038	100.5803
51	0.487339	20	0.191113	8843	84.50069
65	0.623942	40	0.383964	9936	95.37666
43	0.480373	26	0.290458	9998	111.6923
43	0.741753	10	0.172501	3758	64.82576

Figure 1

Click here to access/download;Figure;Figure 1.pdf

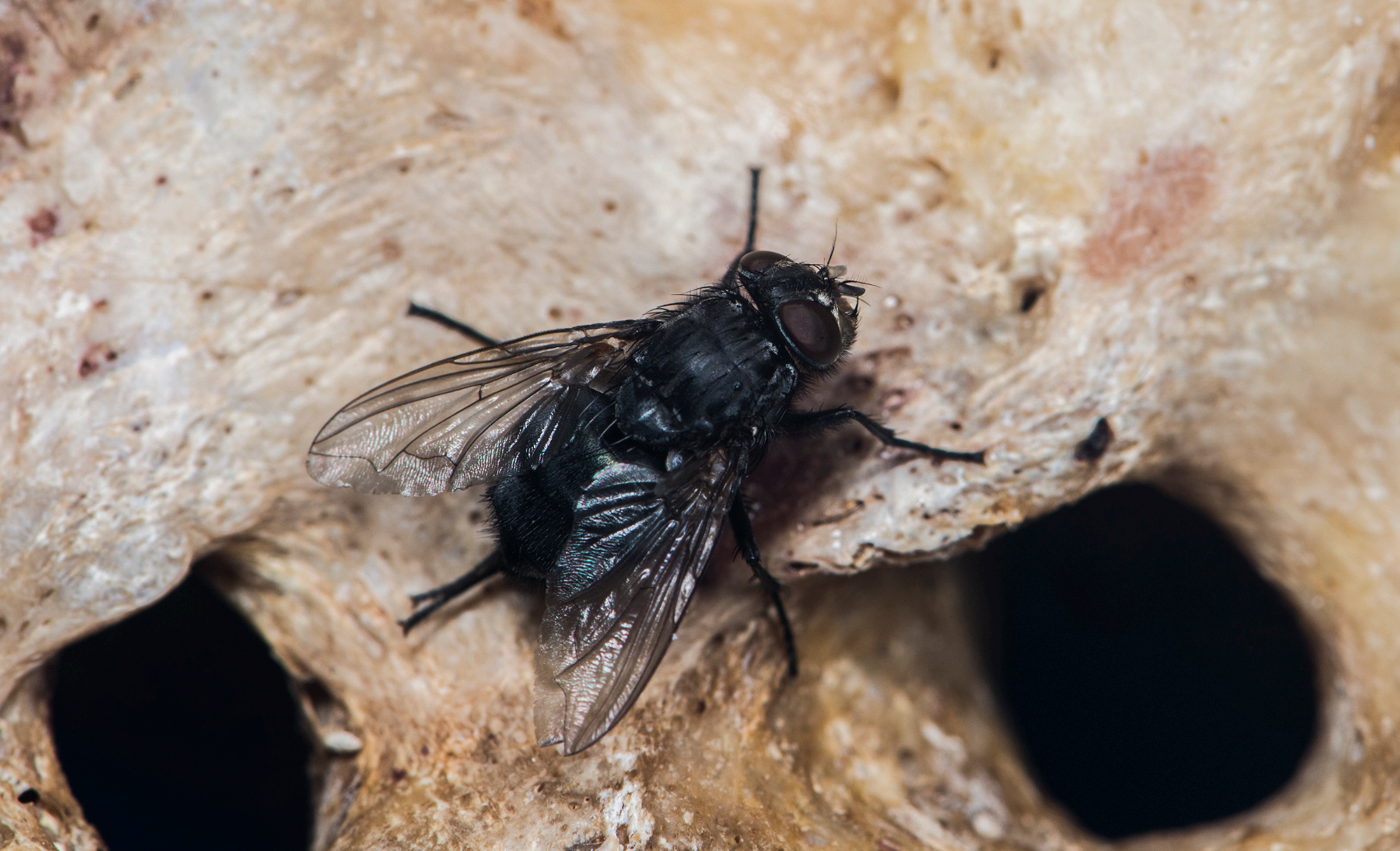

Figure 2

Click here to access/download;Figure;Figure 2.tif

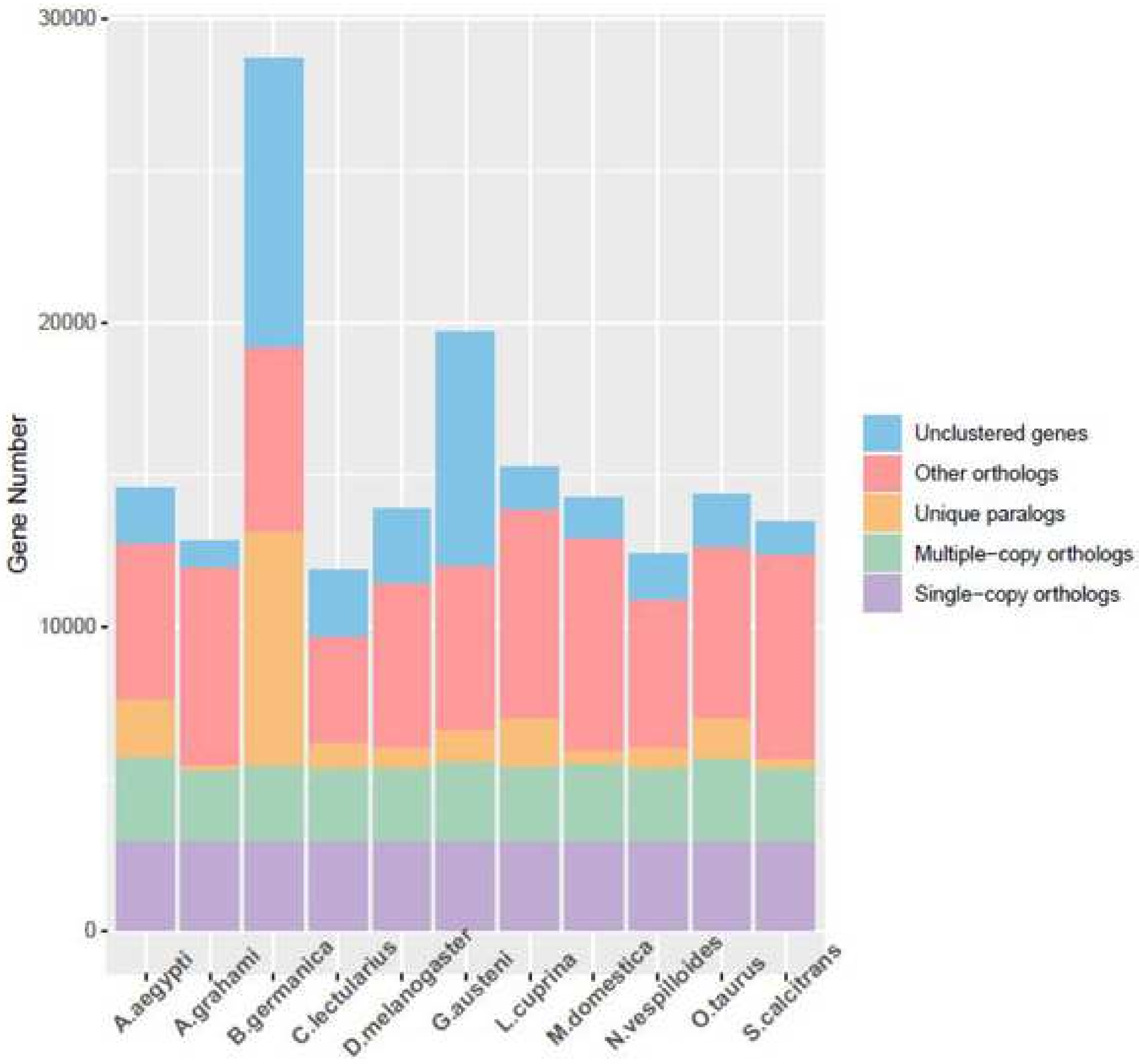

Figure 3

Click here to access/download;Figure;Figure 3.tif

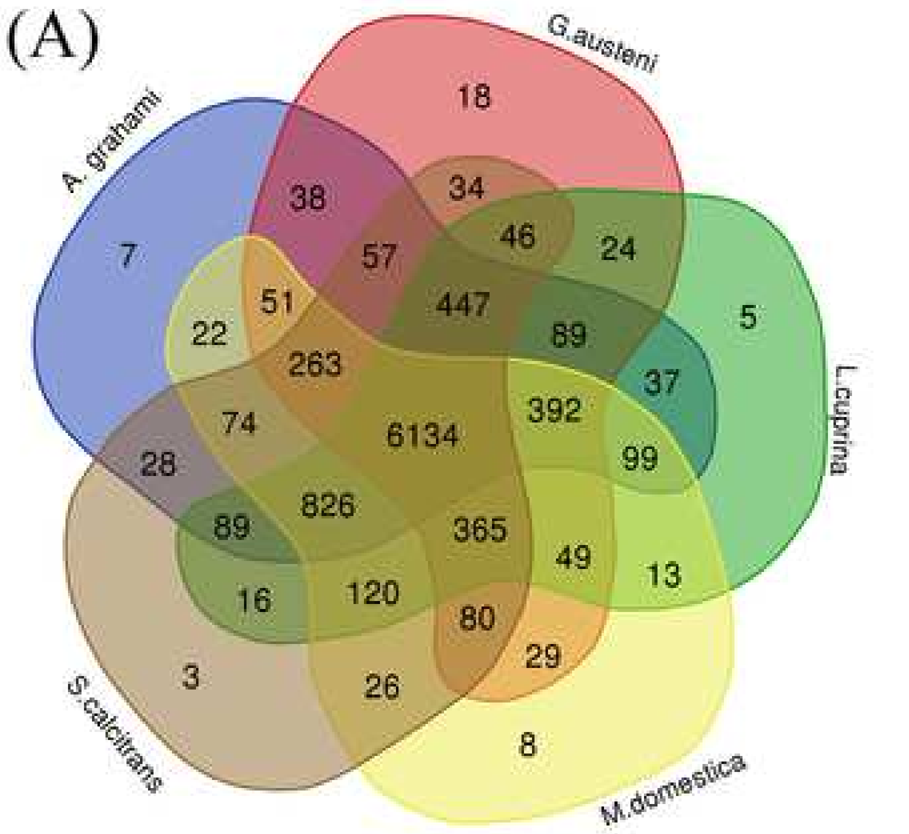

Figure 4

Click here to access/download;Figure;Figure 4.tif

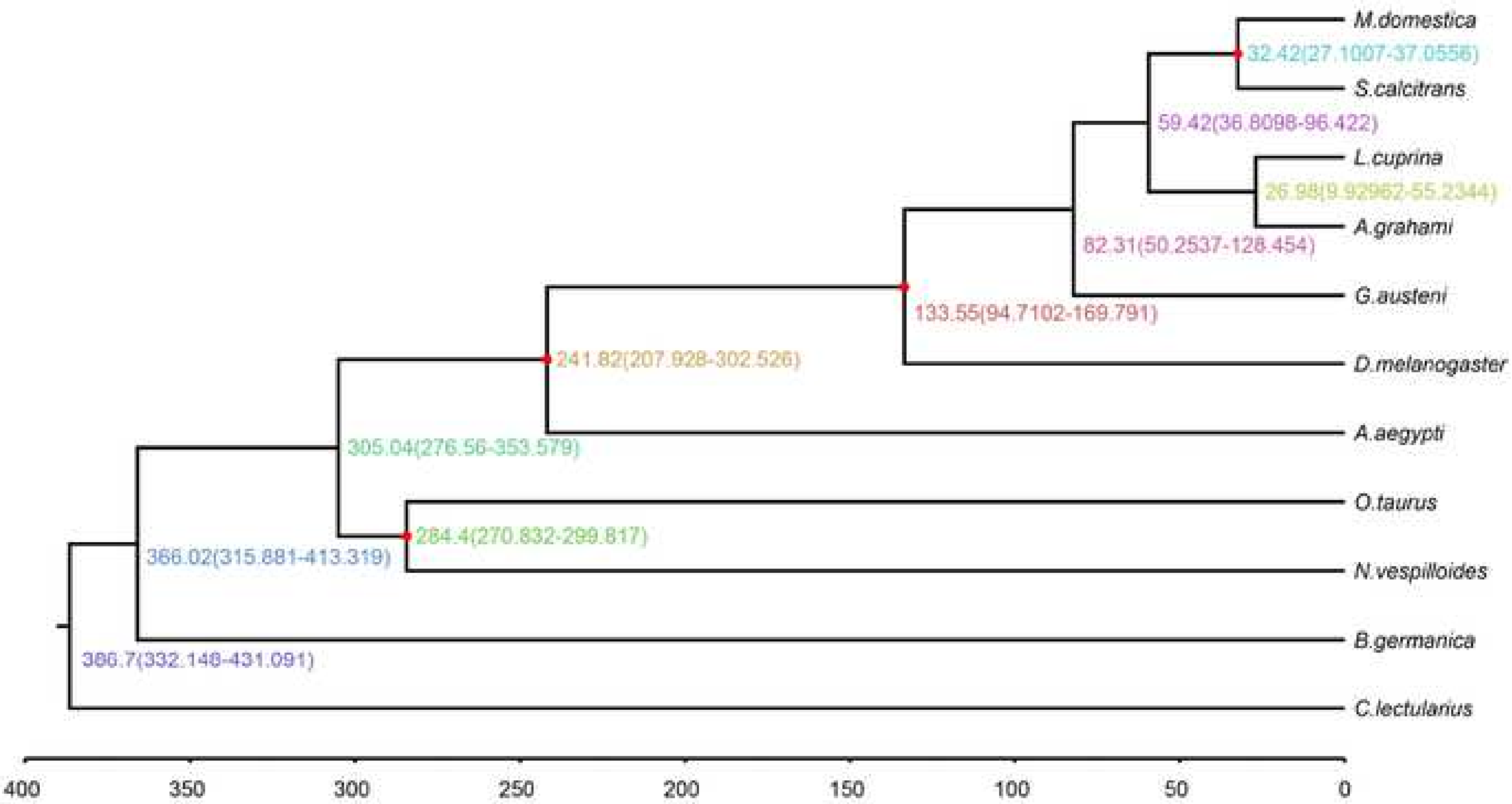

Figure 5

Click here to access/download;Figure;Figure 5.tif

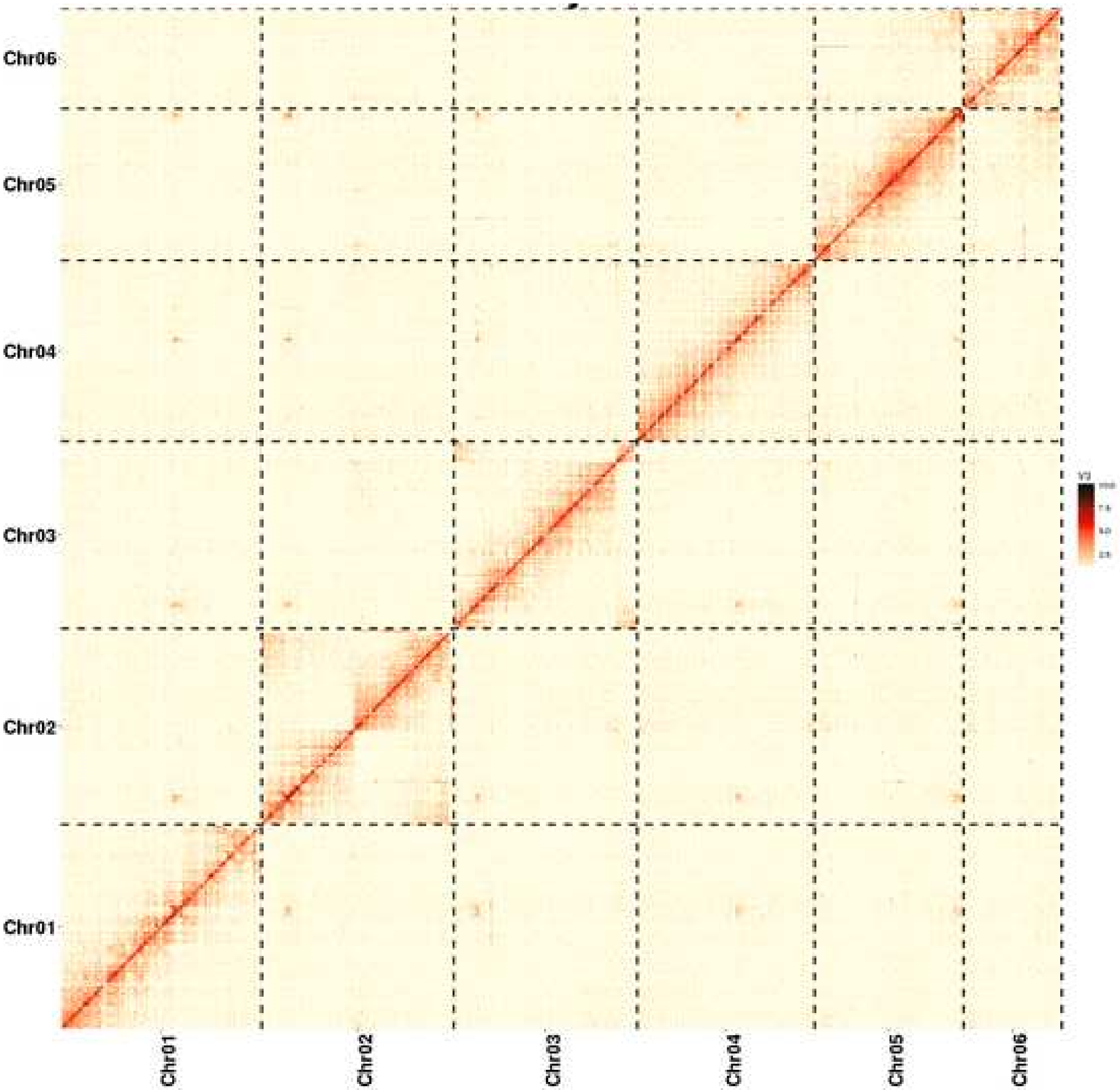

Figure 6

Click here to access/download;Figure;Figure 6.tif

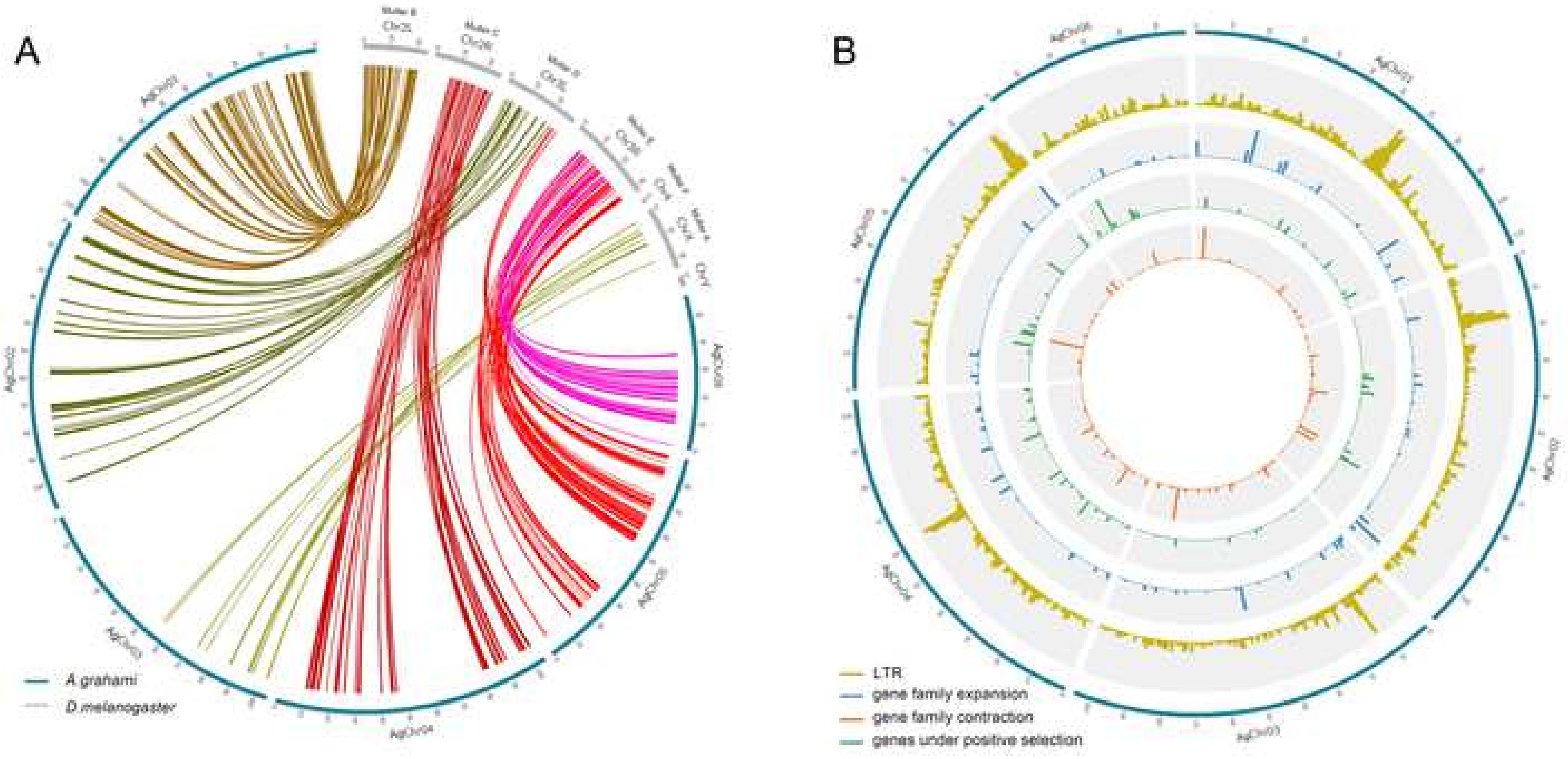

Figure S1

Click here to access/download;Figure;Figure S1.tif

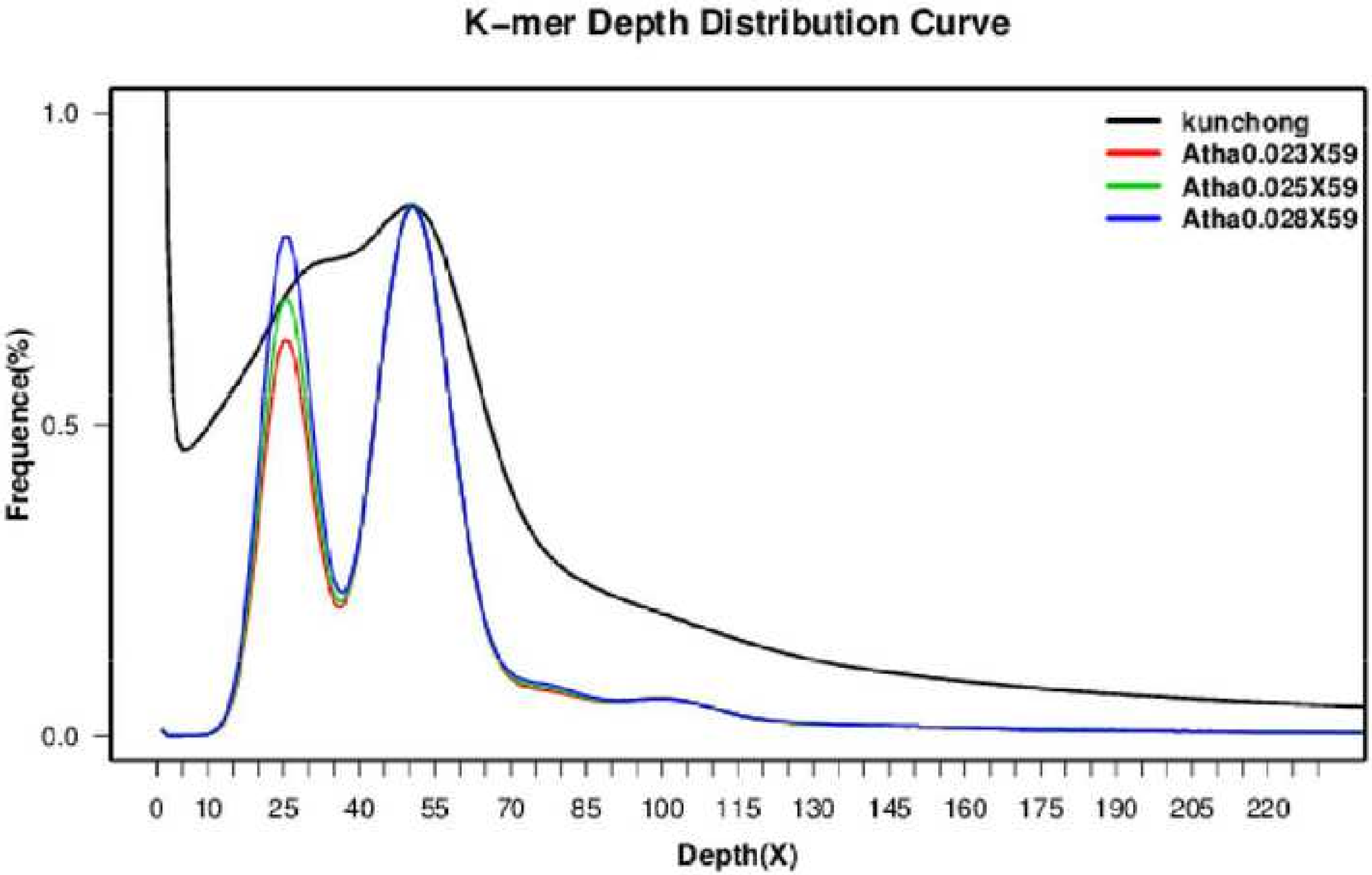

Figure S2

Click here to access/download;Figure;Figure S2.tif

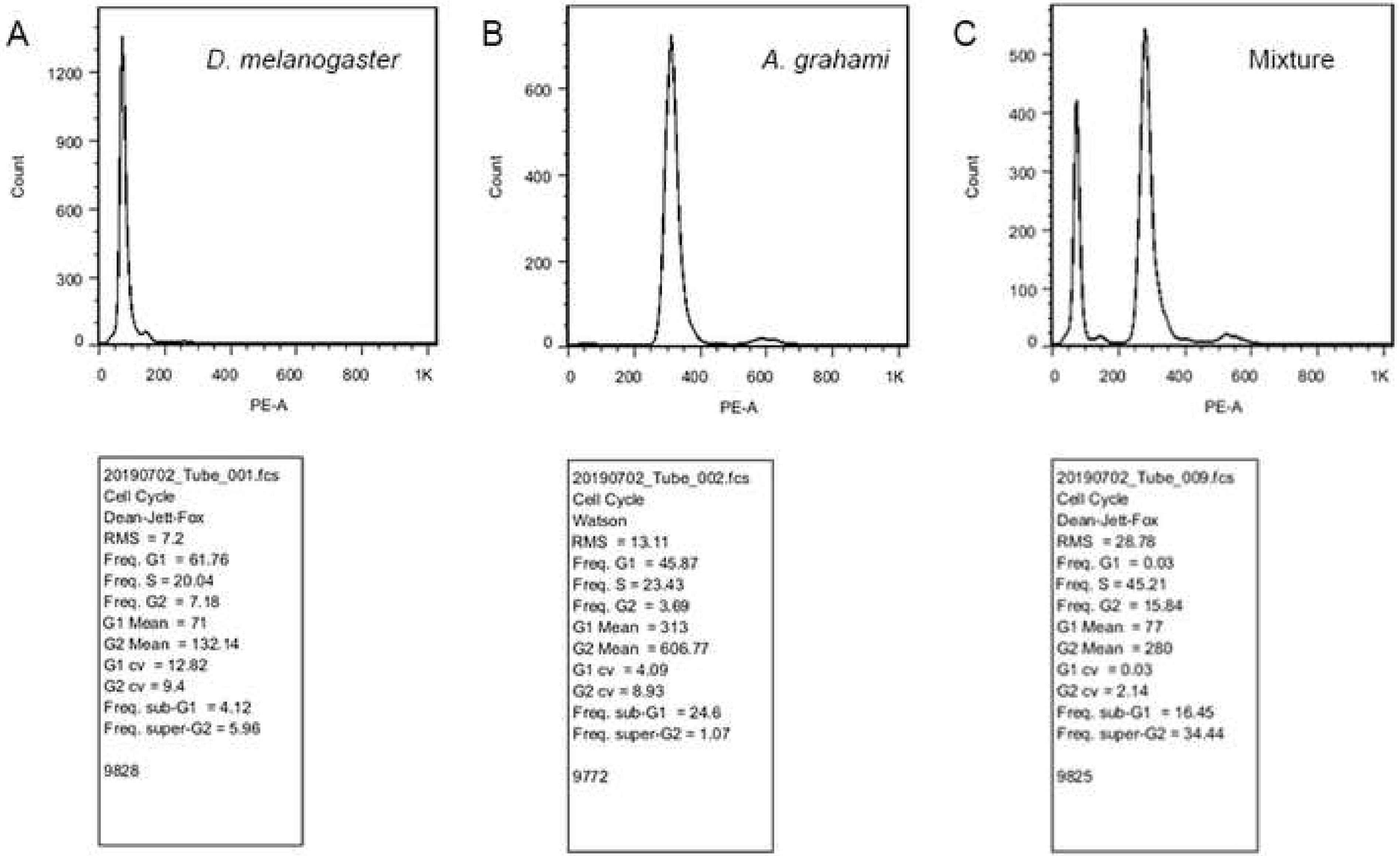

Figure S3

Click here to access/download;Figure;Figure S3.tif

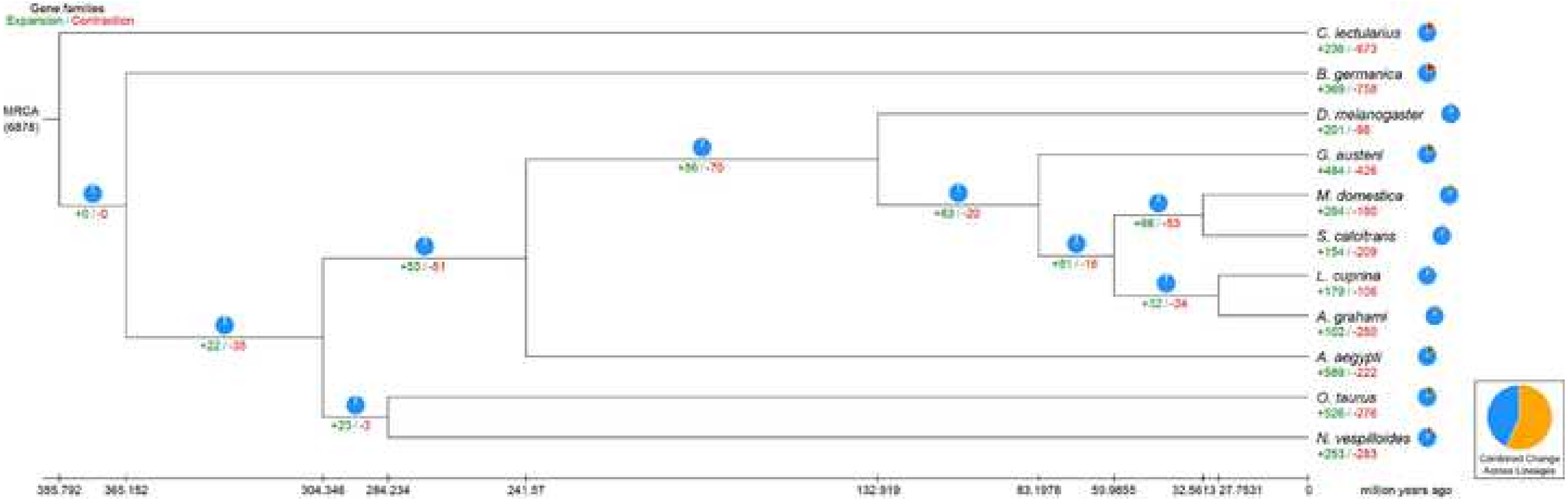

Figure S4

Click here to access/download;Figure;Figure S4.tif

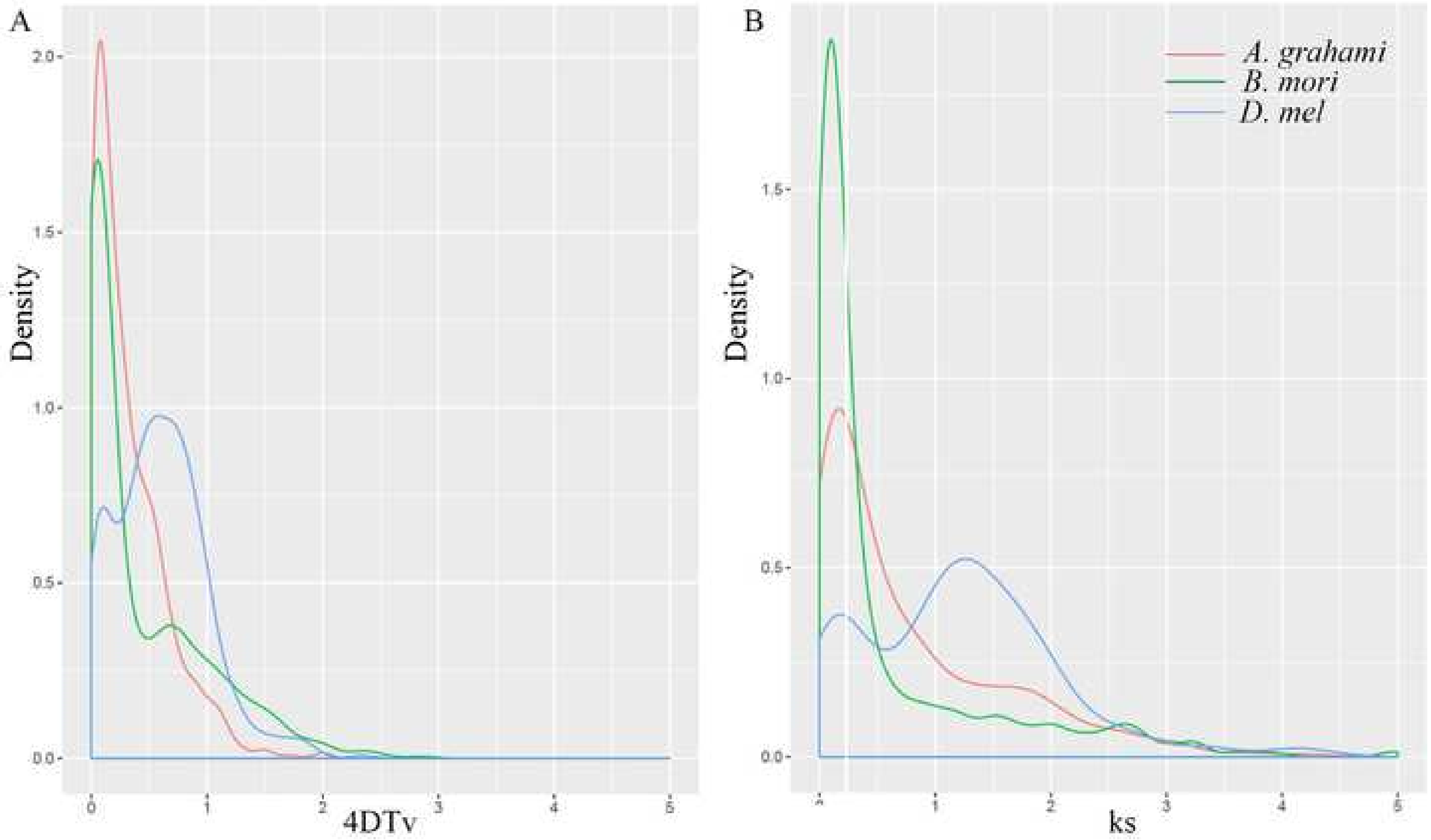

Cover letter Click here to access/download;Personal Cover;Cover letter.doc

Dear Editor,

We would like to submit the enclosed manuscript entitled "**Chromosomal-level genome assembly of *Aldrichina grahami*, a forensically important blow fly**" for possible publication in the journal of ***GigaScience***.

Flies of Calliphoridae belong to the most frequent entomological evidence applied in **forensic cases**. ***Aldrichina grahami*** has some unique biological characteristics which obviously different from the other blow fly species. It has great potential application value in forensic research and practice like estimation on the minimum postmortem interval (min**PMI**) and other aspects. But a qualified genomes resource of it is still unavailable.

Here we provide a genome assembly with high quality of *A. grahami* generated by **Pacifc BioSciences (PacBio) Sequel** sequencing platform and **Hi-C** technology. It represents the first **chromosome-level** genome resource in calyptratae. This robust genome reference should facilitate the development of research on *A. grahami* and other **necrophagous** blow fly species. **Forensic entomological studies** based on qualified genome resource will definitely consolidate the reliability of entomological evidence and promote its application in **law suit**.

All authors have reviewed the final version of the manuscript and approve it for publication. This manuscript has not been published in whole or in part nor is it being considered for publication elsewhere.

Funding

The present study was supported by grant of the National Natural Science Foundation of China (81571855) and Science Foundation of Hunan Province (2017SK2015).

Ethical approval

None.

Conflict of interest

All authors declare that no competing interests in present work.

Address: School of Basic Medical sciences, Central South University, Changsha 410013, Hunan, China

E-mail: cjf_jifeng@163.com

Tel: +86 731 82650414
