# Supplementary material for: Chromosome-level genome assembly of Aldrichina grahami, a forensically important blowfly
Source: Gigascience. 2020 Mar 19;9(3):giaa020. doi: 10.1093/gigascience/giaa020 (PMC7081965; doi:10.1093/gigascience/giaa020)
Supplement: giaa020_GIGA-D-19-00066_Revision_2 [file giaa020_giga-d-19-00066_revision_2.pdf]

GigaScience

Chromosomal-level genome assembly of Aldrichina grahami, a forensically important blow fly

--Manuscript Draft--

**Manuscript Number:**	GIGA-D-19-00066R2
**Full Title:**	Chromosomal-level genome assembly of Aldrichina grahami, a forensically important blow fly
**Article Type:**	Data Note
**Funding Information:**	National Natural Science Foundation of China (81571855) — Pro. Jifeng Cai<br>Science Foundation of Hunan Province (2017SK2015) — Pro. Jifeng Cai
**Abstract:**	(see below)
**Corresponding Author:**	Jifeng Cai

Funding Information:

Funder	Recipient
National Natural Science Foundation of China (81571855)	Pro. Jifeng Cai
Science Foundation of Hunan Province (2017SK2015)	Pro. Jifeng Cai

Abstract:

Background : Blow flies (Diptera: Calliphoridae) are the most commonly found entomological evidence in the forensic investigation. Aldrichina grahami with some unique biological characteristics that distinguish it from other blow flies is a blow fly species of forensic importance. Its development rate, pattern and life cycle can provide valuable information for the estimation of the minimum postmortem interval (minPMI).
Findings : Herein we provide a chromosomal-level genome assembly of A. grahami that was generated by Pacific BioSciences (PacBio) sequencing platform and chromosome conformation capture (Hi-C) technology. A total of 50.15 Gb clean reads of A. grahami genome were generated. Programs FALCON and Wtdbg, as the common assembly tools for PacBio long reads, were utilized to construct the genome of A. grahami which resulted in an assembly of 600 Mb and 1604 contigs with N50 size of 1.93 Mb. We predicted 12823 protein-coding genes, 99.8% of that was functionally annotated based on de novo genome (SRA: PRJNA513084) and transcriptome (SRA: SRX5207346) of A. grahami . According to the co-analysis with 10 other insect species, the clustering and phylogenetic reconstruction of the gene families were performed. Using Hi-C sequencing, a chromosomal-level assembly of 6 chromosomes was generated with scaffold N50 of 104.7 Mb. Of these scaffolds, nearly 96.4% were anchored to the total A. grahami genome contig bases.
Conclusions : The present study provides a robust genome reference of the A. grahami which supplements vital genetic information for the nonhuman forensic genomics, and facilitates the future research of A. grahami and other necrophagous blow fly species used in forensic medicine.

Background : Blow flies (Diptera: Calliphoridae) are the most commonly found entomological evidence in the forensic investigation. A. grahami with some unique biological characteristics that distinguish it from other blow flies is a blow fly species of forensic importance. Its development rate, pattern and life cycle can provide valuable information for the estimation of the minimum postmortem interval (minPMI).
Findings : Herein we provide a chromosomal-level genome assembly of A. grahami that was generated by Pacific BioSciences (PacBio) sequencing platform and chromosome conformation capture (Hi-C) technology. A total of 50.15 Gb clean reads of A. grahami genome were generated. Programs FALCON and Wtdbg, as the common assembly tools for PacBio long reads, were utilized to construct the genome of A. grahami which resulted in an assembly of 600 Mb and 1604 contigs with N50 size of 1.93 Mb. We predicted 12823 protein-coding genes, 99.8% of that was functionally annotated based on de novo genome (SRA: PRJNA513084) and transcriptome (SRA: SRX5207346) of A. grahami . According to the co-analysis with 10 other insect species, the clustering and phylogenetic reconstruction of the gene families were performed. Using Hi-C sequencing, a chromosomal-level assembly of 6 chromosomes was generated with scaffold N50 of 104.7 Mb. Of these scaffolds, nearly 96.4% were anchored to the total A. grahami genome contig bases.
Conclusions : The present study provides a robust genome reference of the A. grahami which supplements vital genetic information for the nonhuman forensic genomics, and facilitates the future research of A. grahami and other necrophagous blow fly species used in forensic medicine.

Corresponding Author: Jifeng Cai

Powered by Editorial Manager® and ProduXion Manager® from Aries Systems Corporation

	CHINA
**Corresponding Author Secondary Information:**	
**Corresponding Author's Institution:**	
**Corresponding Author's Secondary Institution:**	
**First Author:**	Fanming Meng
**First Author Secondary Information:**	
**Order of Authors:**	Fanming Meng
	Zhuoying Liu
	Jifeng Cai
	Han Han
	Dmitrijs Finkelbergs
	Yangshuai Jiang
	Mingfei Zhu
	Chao Chen
	Yadong Guo
	Yang Wang
	Zongyi Sun
**Order of Authors Secondary Information:**	
**Response to Reviewers:**	Dear Editor Zauner and reviewers,<br>We would like to express our great appreciation to you and reviewers for those constructive comments and suggestions on our manuscript, which provide really valuable and helpful for revising and improving our paper.<br>Here we are resubmitting the manuscript “Chromosomal-level genome assembly of Aldrichina grahami, a forensically important blow fly” by Meng et al. (GIGA-D-19-00066R1) in the 2ed round revision. Because it is the last chance for the potential approval of our manuscript, we have studied comments carefully and tried our best to improve the manuscript. We hope that the correction will meet with approval.<br>In this revised version, we would like to response the questions you highlighted in your letter as below:<br>1) Comparative genomic analyses: Please make another effort to get hold of the Phormia regina data. As the reviewer says, if an NCBI link is not working, you should try to contact the support team or the authors directly.<br>Response: As the reviewer 2 suggested, we tried our best to contact the submitter of Phormia regina genome data. And the author kindly supplied it to us. The Phormia regina genome was finally add to the analysis of corresponding parts in the new version of our manuscript. Related tables and figures were all changed correspondingly.<br>2) flow cytometry: Please carefully consider the latest comment of reviewer 2, Aaron Tarone, who is an expert for this subject matter.<br>Response: We carefully rechecked the results of this part and corrected the manuscript according to the Reviewer 2’s suggestions. We provided the species information which suggested by the reviewer, and recalculated the C-value and related results described in our manuscript. And we also added more details to illustrate our results which we think would meet the reviewer’s advices.<br>3) Please improve the discussion of Muller elements, see reviewer 2' advice<br>Response: We further studied the references and researches suggested by reviewer 2 which provide important information to understanding the issue of Muller elements.<br>To meet the reviewers suggestion and draw a more reliable conclusion, we tried other strategies to determine the sex chromosome and corresponding Muller element of A. grahami (please see details in response to reviewer 2). But no useful information generated by bioinformatics methods. We also tried the software and method

recommended by the reviewer 2, but they required the genome data of both sexes. The genome de novo and Hi-C sequencing of A. grahami were performed on female adults only. So we would like to do this test in our next future research, which requires additional sampling and re-sequencing step for de novo sequencing procedure. Based on the above, we would like to withdraw the conclusion of 'AgChr3 is the X-chromosome of A. grahami' which included in our previous manuscript, due to lack of supporting information.
Though we cannot make an assumption on which chromosome should be the X chromosome, the importance of the chromosome-level assembled A. grahami genome should facilitate to answer this question in the future research. Since present A. grahami genome is the only published chromosome level genome resource of blow fly with forensic importance, it also provide an important genome data to perform a reliable collinearity analysis between A. grahami and other sibling species of the calyptratae. A discussion on this issue was added in the new manuscript also.
4) Language: I would recommend to recruit help from a native speaker who is familiar with the biological context, to correct mistakes and unclear expressions in your revised paper before you resubmit to us.
Response: The new submitted version of our manuscript was carefully revised by native speaker. And we can provide the Certificate of English Proofreading. Changes were marked all in RED.
Thanks again to the reviewers and editors of GigaScience for patiently revised our previous submission and giving us the opportunity to make improvement. We are looking forward to hearing from you.
Sincerely yours!

Reviewer reports:

Reviewer #1: This R1 manuscript describes the sequencing, assembly and analysis of the genome of Aldrichina grahami, a forensically important blow fly. The authors used long-read PacBio sequencing and Hi-C scaffolding technology to produce chromosome-level scaffolds. Considering its forensic importance, the draft genome of A. grahami will be a valuable resource in comparative genomics analyses of other necrophagous dipterans, and arthropods more broadly.
Thank you to the authors for addressing each point in the reviewer's reports, and taking the time to perform the suggested changes/analyses. I believe the resulting R1 manuscript is of a much higher quality.
However, herewith is a list of some of the issues that still need to be addressed.
Response 1: We really appreciate your positive comment. And your suggestions give great help for us to make further improvement of our manuscript. All the correction and revision was list as below.

Reviewer #1: Line 25/26: Clarify/rephrase introductory sentence as it is still not clear
Response: Thanks for your patience on our expression. We change it as below (Line 25).
Blow flies (Diptera: Calliphoridae) are the most commonly found entomological evidence in the forensic investigation.

Reviewer #1: Line 43: Clarify "Nearly 96.4% of these scaffolds was anchored to A. grahami genome" as it is still not clear. Is there a different way to describe this?
Response: Thank you for your suggestion. We changed it to "Using Hi-C sequencing, a chromosomal-level assembly of 6 chromosomes was generated with scaffold N50 of 104.7 Mb. Of these scaffolds, nearly 96.4% were anchored to the total A. grahami genome contig bases." (Line:43)

Reviewer #1: Line 52: Clarify "medical legal investigation" and how arthropods are used
Response: Thanks for the reviewer's suggestion. The glossary "medical legal" was inappropriate. We changed it to "medicolegal" (Line 53). We tried to answer the reviewer's the question simply as below.
The main test of forensic entomology was to deals with arthropod involved in crimes such as murder, suicide, rape and physical abuse [1]. It was also described as medicolegal or medicocriminal forensic entomology [2].
Several areas of forensic entomology have progressed considerably in the past few decades. We use the estimation on the PMI, which represents the main tasks of

forensic investigation, to make an example of how entomological evidence be used in medicolegal investigation. One can use entomological data to estimate the PMI in two basic ways. First, insects are usually the first organisms to find and locate corpse. Often blow flies, could oviposit on carrion within the first few hours or even minutes after death if given access [3]. So the determination of the age of developing maggots on corpse, which just like a biological clock, is the basis for the PMI estimation. During the earlier stage of decomposition, the estimation could be based on the time length needed for particular species to develop, which collected at the death scene [4]. Second, corpse is a temporary resource in an opening environment. It could be exploited by a wide diversity of organisms ranging from microbes to vertebrate scavengers. Arthropods usually constitute the major fauna on the corpse, and insects predominate as the most constant, diverse, and conspicuous group under most situation. The arthropod community in and around a corpse changes by a rather predictable succession pattern as the decomposition going on. PMI estimation is based on the change of composition of the arthropod community for it relates to such expected successional patters [5]. This second basic approach to determining the PMI is referred to those corpses in advanced stages of decomposition.
For other arthropods groups, like mites, a number of species are parasitic on humans. Their survival on the body after death can be used to calculate the PMI. The scabies mite (Sarcoptes) can survive for a few days after the death of its host but not more than 2 weeks post-mortem [6]. Demodex is a common inhabitant of human face hair follicles and can be found alive 55 h after the death of their host [7-8]. Acarines are frequently seasonal so that their presence can effectively provide ‘date stamp’ evidence.
Crustacean (cribs or shrimps) were usually found on corpse in marine situation with other invertebrate species. But no classic succession was observed in contrast with insect colonization in terrestrial environments. Most invertebrate fauna were opportunistic scavengers. The fauna on the remains, the decomposition and the feeding patterns are valuable in identifying the environment in which the body has decomposed and identifying the origin of marks (feeding) on the remains [9].

Reference
[1] Smith, K. G. V. 1986. A manual of forensic entomology. London: British Museum (Natural History), Comstock. 205 pp.
[2] Hall, R. D. 1991 . Medicocriminal entomology. See Ref. 16, pp. 1-8
[3] Catts E. P. Problems in estimating the postmortem interval in death investigations l[J]. Journal of Agricultural Entomology, 1992, 9(4):245-255.
[4] Catts E P , Goff M L . Forensic entomology in criminal investigations [J]. Annual Review of Entomology, 1992, 37(1):253-272.
[5] Goff, M. L., Flynn, M. M. 1991. Determination of postmortem interval by arthropod succession: a case study from the Hawaiian Islands. Forensic Sci. 36:607-14
[6] Mellanby K (1943) Scabies. Oxford University Press, Oxford
[7] Ozdemir MH, Aksoy U, Akisu C et al (2003) Investigating Demodex in forensic autopsy cases. Forensic Sci Int 135:226–231. doi:10.1016/S0379-0738(03)00216-0
[8] Ozdemir MH, Aksoy U, Sonmez E et al (2005) Prevalence of Demodex in health personnel working in autopsy room. Am J Forensic Med Pathol 26:18–23.
[9] Amendt J, Goff M L, Campobasso C P, et al. Current concepts in forensic entomology[M]. 2010. p267-268.

Reviewer #1: Table 2: Suggest clarifying the description of "and etc"
Response: Thank you for suggestion. Other is sequences with annotation but not belonging to the above sorts of repetitive genes, such as satellites, simple repeats, retroposon, artefact, helitron and low complexity repeats. Changed in the manuscript also.(Line 246)

Reviewer #1: Line 393: Why 'et al.'?
Response: Sorry for this ambiguous writing. All analysis included in this part were distributions of long terminal repeat (LTR), gene family expansion or contraction, and genes under positive selection. It has been changed. (Line 405)

Reviewer #1: Line 416-418: Please clarify closing sentence.
Response: Thank you for your suggestion on this point. We revised the closing sentence to make it more clearly and readable (Line 430), and try to explain the sentence to the reviewer.
Based on qualified genome resource, studies of forensically important blow fly species

will reinforce the reliability of entomological evidence and promote its application in the legal criminal investigation.
A crucial test for some forensic cases is the identification of geographic distribution of insect samples. This identification largely depends on the existence of genetic data in different regions, enough genetic differentiation between regions and the quality of the analytical method. Some promising findings exist, such as the application of different genetic markers. And phylogenetic analyses have successfully been admitted as evidence in legal criminal cases [1]. But it is suggested that we are still far from a foundational validation of this approach to be used in legal cases. Limitations come from different aspects like the insufficiency of reference databases, lacking of reference genome sequences, metadata, and representative genetic diversity coverage [2]. We think a high-quality genome resource will provide a valuable reference genome for a particular insect group. And the following population studies will explore additional genetic markers that can characterize its geographic distributions. The genetic analyses based on large datasets (such as whole genomes) will definitely provide high statistical confidence which can be useful in legal criminal cases [3].
Reference
[1]Bhattacharya S. Science in court: Disease detectives. Nature. 2014; 506(7489):424–6. https://doi.org/10.1038/506424a PMID: 24572408.
[2]Sjodin A, Broman T, Melefors O, Andersson G, Rasmusson B, Knutsson R, et al. The need for highquality whole-genome sequence databases in microbial forensics. Biosecur Bioterror. 2013; 11 Suppl1:S78–86. https://doi.org/10.1089/bsp.2013.0007 PMID: 23971825.
[3]Jager AC, Alvarez ML, Davis CP, Guzman E, Han Y, Way L, et al. Developmental validation of the MiSeq FGx forensic genomics system for targeted next generation sequencing in forensic DNA casework and database laboratories. Forensic science international Genetics. 706 2017; 28: 52-70. doi:10.1016/j.fsigen.2017.01.011.

Reviewer #2: The authors did a good job of beginning to address the issues raised in the previous reviews. However, there are some areas of concern to address with the new round.

1. The authors should make more of an attempt to either standardize or justify specific comparative genomic comparisons they are making. The excuse for not including Phormia regina in all analyses seems wrong. If NCBI has a faulty link, authors should work with NCBI to fix it and gain access to the genome. Also, did the manuscript authors try contacting the authors of the Phormia regina genome paper to see if they can provide?
Response: We greatly appreciate your suggestion and recommendation on this point. As you suggested, we tried our best to contact the submitter of Phormia regina genome. The submitter kindly provided the genome data of Phormia regina to us. So we add the Phormia regina genome to all the comparative genomic analysis steps, including homologous gene family, phylogenetic analysis, evolutionary history, and genes gain and lose, to gain a more complete and reliable result. The results were added to relative parts in the new version of manuscript respectively (line 349). And changes were also made in figures and tables accordingly (Figure 2, 4, S2, S3, Table 4, S5, S6, S9, S11). Thanks again for your suggestion and we thought this supplementary work could make an improvement to our study.

2. The authors are to be commended for their reevaluation of genome size based on flow cytometry. However, a few items are concerning in their description and implementation of the work. (1) First, in supplemental data the authors clarify they used Drosophila melanogaster as the reference genome, but they do not report the genus or species name in the main text. Since D. virilis is a common genome size reference, the authors should clarify which species (and strain) they analyzed to identify genome size. It would also help to see the genome size the authors are assuming (175 Mb for D. melanogaster?). (2) The CV values for the histograms appear to be unacceptably large (I believe <3 is considered good practice). (3) Finally, when one looks at the genome size histograms there appears to be a linearity issues with the analysis. The peaks for 2C/4C do not appear with 4C as 2x of 2C. This can be a problem with some cytometers, which do not all report fluorescence in a linear manner. This could indicate a biased genome size estimate.
Response: Thanks a lot for your suggestion on this part of work. It provided an opportunity for us to supplement more information which related to the results. We

supplemented the species information of which we used as reference genome. And according to you suggestion, we rechecked the result part of flow cytometry test (Line 214-222). And we would like to describe it in details as below.
The fruit fly species we used in flow cytometry test was Drosophila melanogaster (strain w118), which C-value was assumed as 0.18 pg (175Mb). We add this information into the new version of manuscript. For the genome size histograms, we rechecked the results in Flow Jo software and output the data with CV value <3. And accordingly, we recalculated the C-value of A.grahami based on the above C-value of Drosophila melanogaster and results of flow cytometry. Changes can be found in Line 218. For the question of linearity issue, on the histograms of Drosophila melanogaster, the 4C (mean=138) peak was actually next to the main 2C peak (mean=71) and overlap was existed between these two peaks. The 4C peak is relative lower than 2C but do exist (also see Michael D. et al Annals of Botany, 2003). We marked the 2C/4C peak on the histogram of the supplementary figure S2. And it could be clearly recognized by the cell cycle analyses in Flow Jo.

3. Muller element analyses are good, but authors need to delve into the literature on this more. Muller F is sometimes a sex chromosome. Others can be associated with sex in other species too. See work by Doris Bachtrog, Rich Meisel, and Max Scott for higher flies (including blow flies and house/stable flies). In addition, coverage analysis might help authors determine elements that differ in coverage between the sexes. It is not sufficient to imply a sex chromosome because it is the sex chromosome in Drosophila melanogaster.
Response: Great thanks to you for your advices and suggested reference work on this issue. After studying those researches closely, we agree that we cannot draw a conclusion that AgChr03 is the sex chromosome of A. grahami according to current limit information, although it is corresponding to the ChrX in D.melanogaster in the collinearity analysis. Based on previous research work as you suggested and other related studies of evolution of sex chromosome and Muller elements in the diptera, orthologs of genes located on the fourth chromosome in D. melanogaster (Muller F) are X-linked in some non-drosophilids or Calyptratae [1-2]. It appears that the Muller element F was an ancestral X chromosome in Diptera [1,3]. Based on the Muller element mapping of the D. melanogaster orthologs, Rich Meisel assigned house fly genes that are conserved as one-to-one orthologs with D. melanogaster to house fly chromosomes [4]. The situation in Max Scott's study of Lucilia cuprina appears that the Muller F of D.melanogaster can be highly mapped to the ChrX of the former [5]. However, the collinearity of chromosome between D. melanogaster and A. grahami exhibited Chr4 of D.melanogaster has no collinear position on A. grahami chromosome in present work. Since the assembled chromosome of A. grahami in present paper is the only published resource in calyptratae so far, we cannot perform the same analysis between A. grahami and other sibling species. And limitation of the software MCSCAN in performing collinearity analysis made it hard to draw a definite conclusion by a single result [6].
To get more information, we searched for other strategies to infer the sex chromosome of A. grahami. First, we tried to use the contigs of P. regina's ChrX to align with A. grahami genome assemblage. So we downloaded the predicted contigs list of ChrX of P. regina described in J. Picard's study (2016) [8]. But only 143 contigs sequences could be recognized from the genome resource of P. regina which deposited on NCBI. And merely 2 contigs could be aligned to chromosomes of A. grahami. Second, we download the ChrX specific genes of L. cuprina documented in study of Maxwell J. Scott (2018) [5], and blasted against the A. grahami genome. But no useful information generated either. We further downloaded the software CQ-calculate, which has been used in determine X/Y chromosome of the Phomia regina [7]. But the software needs both the male and female genome resource to perform the prediction according to its instruction [8]. And the coverage analysis which recommended by the reviewer 2 also requires the genome data of two sexes. The genome de novo and Hi-C sequencing of A. grahami were performed on female adults only. Therefore, current data do not yield convincing information on sex chromosomes. However, this is an interesting question for future research, which involves additional samples and resequencing.,
Based on the above, we would like to withdraw the conclusion of AgChr03 is the sex chromosome of A. grahami. And since the Muller F of A. grahami is not distinguishable in present work, we cannot make an assumption on which chromosome should be the X chromosome either. We add a brief discussion in the new version of manuscript (Line 400).

Powered by Editorial Manager® and ProduXion Manager® from Aries Systems Corporation

[1] Linger, Rebecca J. , et al. "Dosage compensation of X-linked muller element F genes but not X-linked transgenes in the Australian sheep blowfly." PLOS ONE 10.10(2015):e0141544-.
[2] Landeen, Emily & Presgraves, Daven. (2013). Evolution: From autosomes to sex chromosomes - and back. Current biology : CB. 23. R848-50. 10.1016/j.cub.2013.08.021.
[3] Vicoso B, Bachtrog D. Reversal of an ancient sex chromosome to an autosome in Drosophila. Nature.2013; 499(7458):332–5. doi: 10.1038/nature12235 PMID: 23792562; PubMed Central PMCID:PMC4120283.
[4] Richard P. Meisel, Jeffrey G. Scott, Andrew G. Clark, Transcriptome differences between alternative sex determining genotypes in the house fly, Musca domestica, Genome Biology and Evolution, Volume 7, Issue 7, July 2015, Pages 2051–2061,
[5] Davis R J , Belikoff E J , Scholl E H , et al. no blokes, Is Essential for male viability and X chromosome gene expression in the Australian sheep blowfly[J]. Current Biology, 2018:S0960982218306079-.
[6]Wang Y , Tang H , Debarry J D , et al. MCScanX: a toolkit for detection and evolutionary analysis of gene synteny and collinearity[J]. Nucleic Acids Research, 2012, 40(7):e49-e49.
[7] Andere A A , Platt R N , Ray D A , et al. Genome sequence of Phormia regina Meigen (Diptera: Calliphoridae): implications for medical, veterinary and forensic research[J]. BMC Genomics, 2016, 17(1):842.
[8] Hall AB, Qi Y, Timoshevskiy V, Sharakhova MV, Sharakhov IV, Tu Z. Six novel Y chromosome genes in Anopheles mosquitoes discovered by independently sequencing males and females. BMC Genomics. 2013;14:273. Published 2013 Apr 23. doi:10.1186/1471-2164-14-273

Minor

Q:Uningnorable is a difficult word to read through. Maybe replace with "a detectable and potentially important"
Response: Corrected (Line 97).

Q:Report COI sequence to a database (Nucleotide database on NCBI?)
Response: We have reported the COI sequence to the Nucleotide database on NCBI, and got an accession number of MN537823 (Line 117).

Q:Provide the specific voucher number for the specimens submitted as vouchers to the museum
Response: We provided all the information of voucher number into a supplementary table (Table S12) which corresponds to the voucher samples restored in the forensic insect herbarium of the Department of Forensic Science.

Q:Why were wings/gut used to provide DNA? Or was DNA isolated without wings and gut?
Response: Wings and gut were not used in DNA extraction. They were removed from the adult body firstly. The remaining parts were used for DNA extraction. The sentence has been rephrased (Line 131).

Q:Why do authors sometimes italicize necrophagia/parasitism/hematophagia?
Response: Sorry for these incorrect typing. It has been corrected (Line 294).

Q: More justification for parameter settings on divergence time analyses would help.
Response: Thank you for your suggestion. As you suggested, we revised this part of description in a detailed way to make it more readable. Changes can be found in Line 338-347 of the new manuscript.
The PAML mcmctree program (v4.5, RRID: SCR 014932) was used to determine divergence times with the calculation of approximate likelihood test, molecular clock and substitution model of REV [90]. The primary parameters of mcmctree were set as clock = 2 (independent rates model following a log-normal distribution), RootAge = < 4 (400 Myr for a calibration to the root of the phylogenetic tree) , model = 7 (the substitution model, REV), BDparas = 110 (default value is 110, parameters controlling the birth-death process), kappa_gamma = 62 (transition/transversion rate ratio), alpha_gamma = 11 (gamma shape parameter for variable rates among sites),

	rgene_gamma = 23.606 (Dirichlet-gamma prior for the mean substitution rate), sigma2_gamma = 11.03 (Dirichlet-gamma prior for the rate drift parameter). (Line 338)<br><br>Q: On line 146 - which fragments were proper size?<br>Response: Sorry for the inadequate information. It should be descripted as 'the fragments with length larger than 15Kb were selected by the Blue Pippin device'. We corrected it in the manuscript (Line 150).
Additional Information:	
Question	Response
Are you submitting this manuscript to a special series or article collection?	No
**Experimental design and statistics**<br><br>Full details of the experimental design and statistical methods used should be given in the Methods section, as detailed in our Minimum Standards Reporting Checklist. Information essential to interpreting the data presented should be made available in the figure legends.<br><br>Have you included all the information requested in your manuscript?	Yes
**Resources**<br><br>A description of all resources used, including antibodies, cell lines, animals and software tools, with enough information to allow them to be uniquely identified, should be included in the Methods section. Authors are strongly encouraged to cite Research Resource Identifiers (RRIDs) for antibodies, model organisms and tools, where possible.<br><br>Have you included the information requested as detailed in our Minimum Standards Reporting Checklist?	Yes
**Availability of data and materials**<br><br>All datasets and code on which the conclusions of the paper rely must be either included in your submission or deposited in publicly available repositories	Yes

Powered by Editorial Manager® and ProduXion Manager® from Aries Systems Corporation

(where available and ethically appropriate), referencing such data using a unique identifier in the references and in the “Availability of Data and Materials” section of your manuscript.<br><br>Have you have met the above requirement as detailed in our Minimum Standards Reporting Checklist?	

Manuscript

Click here to view linked References

Click here to access/download;Manuscript;maitext20191020.docx

Chromosomal-level genome assembly of *Aldrichina grahami*, a forensically important blow fly

Fanming Meng[1], Zhuoying Liu[1], Han Han[1], Dmitrijs Finkelbergs[1], Yangshuai Jiang[1], Mingfei Zhu[2], Yang Wang[2], Zongyi Sun[2], Chao Chen[3], Yadong Guo[1], Jifeng Cai[1]*

[1] School of Basic Medicine, Central South University, Changsha, Hunan Pro, China

[2] Nextomics Biosciences, Wuhan, Hubei Pro, China

[3] Institute of Apicultural Research, Chinese Academy of Agricultural Sciences

* Corresponding authors

Emails:

F. M: mengfanming1984@163.com;

Z. L: 214872404@qq.com;

H. H: 583538543@qq.com;

D. F: dfinkelbergs@yahoo.com

Y. J: 1464804060@qq.com

M. Z: zhumingfei@grandomics.com

Y. W: wangyang-1@grandomics.com

Z. S: sunzongyi@grandomics.com

C. C: chenchaoiar@163.com

Y. G: gdy82@126.com

J. C: cjf_jifeng@163.com

Abstract

Background: Blow flies (Diptera: Calliphoridae) are the most commonly found entomological evidence in the forensic investigation. *Aldrichina grahami* with some unique biological characteristics that distinguish it from other blow flies is a blow fly species of forensic importance. Its development rate, pattern and life cycle can provide valuable information for the estimation of the minimum postmortem interval (minPMI).

Findings: Herein we provide a chromosomal-level genome assembly of *A. grahami* that was generated by Pacific BioSciences (PacBio) sequencing platform and chromosome conformation capture (Hi-C) technology. A total of 50.15 Gb clean reads of *A. grahami* genome were generated. Programs FALCON and Wtdbg, as the common assembly tools for PacBio long reads, were utilized to construct the genome of *A. grahami* which resulted in an assembly of 600 Mb and 1604 contigs with N50 size of 1.93 Mb. We predicted 12823 protein-coding genes, 99.8% of that was functionally annotated based on *de novo* genome (SRA: PRJNA513084) and transcriptome (SRA: SRX5207346) of *A. grahami*. According to the co-analysis with 10 other insect species, the clustering and phylogenetic reconstruction of the gene families were performed. Using Hi-C sequencing, a chromosomal-level assembly of 6 chromosomes was generated with scaffold N50 of 104.7 Mb. Of these scaffolds, nearly 96.4% were anchored to the total *A. grahami* genome contig bases.

Conclusions: The present study provides a robust genome reference of the *A. grahami* which supplements vital genetic information for the nonhuman forensic genomics, and facilitates the future research of *A. grahami* and other necrophagous blow fly species used in forensic medicine.

Keywords: *Aldrichina grahami*; Blow fly; Necrophagous; Forensic entomology; Minimum postmortem interval; Genome assembly

Data Description

Background

Forensic entomology focuses on the application of insects and other arthropods in the medicolegal investigation. Studying of the development rate of insect colonizers on the corpse and insect succession pattern during corpse decomposing, can assist the estimation of the minimum postmortem interval (minPMI), which represents the main task of the forensic investigation [1-3]. In addition, insect evidence is helpful in the detection and recognition of wounds, estimation of the time length of neglect or abuse, and investigation of the cause of death [4-7]. The most important group of insects for forensic investigation is the Diptera, especially those necrophagous fly species of Calliphoridae [8, 9]. Flies of this fauna, usually called blow fly, consist of many species with the parasitic life-style or necrophagous life-style [10, 11]. The reliable life cycle of these necrophagous flies can provide vital information for forensic entomologists or investigators to infer a relatively accurate minPMI under certain assumptions [8, 12-14].

Figure 1. Female adult of *Aldrichina grahami* on the corpse.

Aldrichina grahami (Aldrich, 1930; NCBI:txid252811, homotypic synonym: *Calliphora grahami*) (Fig. 1) is a common blow fly species indigenous to East Asia [15, 16] which has expanded to the American continent in the past several decades [17-19]. It usually breeds on carcasses or feces, posing a potential threat of contaminate human food [15]. *A. grahami* is a forensically important insect because of its necrophagous behavior, seasonal distribution, and particularly unique

characteristic of low temperature tolerance, all of which distinguish it from other necrophagous flies [20-22]. *A. grahami* is frequently the first species to colonize the corpse in early spring and late autumn, when the ambient temperature is relatively lower. In some extreme cases this species can be the only colonizer [23, 24]. The information provided by seasonal distribution pattern of *A. grahami* could be applied as a potential 'season stamp' of the time of death in the PMI estimation, especially in the period when other insects are inactive [22, 25]. Moreover, the successful extraction and identification of human DNA material from gut contents of *A. grahami* and other blow fly larvae can provide important information about the missing corpse or help to interpreting the evidence used for forensic investigation [26, 27]. Age-dependent altering pattern of cuticular hydrocarbons in larvae cuticle has great application potential in the forensic investigation [28, 29]. Besides the forensic importance, cases of myiasis caused by *A. grahami* have been reported routinely in China, especially when people travel back from the undeveloped regions [30-33]. This blow fly species is also a potential transmitter of pathogens, such as H5N1 influenza virus, which could cause serious public health problems in animals and human beings [34].

Researches of insect biochemistry and physiology prompt our deeper understanding of *A. grahami* [35-38]. Nuclear materials are primarily applied to distinguish *A. grahami* from other sibling Diptera species [39-42]. Several researchers have described the developmental patterns of *A. grahami* under different environment conditions [20, 21]. Nonetheless, the genome of *A. grahami* is still unavailable, which impedes its further applications in forensic research. Previous studies have indicated the a detectable and potentially important influence of the variation in the genetic level on the time length of development and life cycle of the fly species among

geographic populations of the blow fly [43-45]. It was also recommended that the investigation of such forensic investigations should be based on high-quality genome reference of the investigated fly species [46-48]. Here we provide a chromosome-scale scaffolding of the genome assembly of this forensically important blow fly using Pacific BioSciences (PacBio) sequencing platform and chromosome conformation capture (Hi-C) method, which promotes the future research of forensic and medical science.

Genome Sequencing and Assembly

Sample preparation

The first generation of *A. grahami* was collected using beef liver as baits, in Changsha (Hunan Province, China) in March 2017. The species identification was performed through morphological and molecular methods. The fly species were distinguished following the morphological description found in literature of Fan's study (1992) [15]. Then cytochrome oxidase gene I (*COI*) as a molecular marker was amplified from the DNA of *A. grahami* using the previously mentioned method (Primer F: 5-TACAATTTATCGCCTAAACTTCAGCC-3; R: 5-CATTTCAAGCTGTGTAAGCATC-3) [39]. After sequencing the amplification product (ABI 3730xl, USA), the result was searched by BLAST and deposited into the NCBI website (Accession number: MN537823). It was recognized as the *A. grahami*. The blow flies were bred for more than 20 generations in the laboratory of the School of Basic Medicine, Central South University. The new emerged and unmated female adults were used for the DNA extraction.

After the sample collection, the used tissues were immediately immersed into the

liquid nitrogen and stored at -80℃. DNA was extracted using the Cetyltrimethyl Ammonium Bromide (CTAB) method followed by the introduction of Size-Selected 20 kb SMRTbell^{TM} Libraries for genomic DNA preparation. The quality of the extracted genomic DNA was checked using gel electrophoresis with 0.7% agarose. Then Nanodrop spectrophotometer (Thermo Fisher Scientific) was used to calculate the DNA purity. The concentration of extracted material was examined by Qubit fluorimeter (Invitrogen, Carlsbad, CA, USA).

New males and females were sampled for the transcriptome sequencing. After the extraction quality control and library construction, the Illumina Hiseq X10 platform was used to perform the RNA-seq. Five new female adults with their wings dissected and gut removed, were used for library construction.

Every voucher specimen was assigned with a unique code. All of the specimens were deposited in the forensic insect herbarium of the Department of Forensic Science, Central South University, Changsha.

Library construction and sequencing

Two libraries were constructed before sequencing. Firstly a library of short-insert length (400 bp) was constructed by Illumina TruSeq Nano DNA Library Prep Kits. The short-insert library sequencing was performed on the Illumina HiSeq X Ten instrument at Genetron Health (Beijing, China) using whole-genome shotgun sequencing (WGS) strategy. A total of 46.05 Gb of raw data were collected and subsequently filtered. Finally, 42.4 Gb of clean data for short reads were generated (Table S1).

The long reads library of 20 kb was prepared using a SMRTbell DNA Template Prep Kit 1.0 (PacBio p/n 10Tal-259-100). DNA fragments of approximately 20Kb

were generated by shearing genomic DNA material using a Covaris G-TUBE™ (Kbiosciences p/n 520079). The sheared genomic DNA was damage-repaired and end-repaired using polishing enzymes. The blunt-end ligation resulted from the exonuclease treatment was used to generate SMRTbell template. After that, fragments with proper size (⩾15Kb) were subsequently selected by Blue Pippin device (Sage Science, Inc., Beverly, MA, USA). The DNA 12000 Kit for Agilent Bioanalyzer 2100 (Agilent p/n 5067-1508) was used to figure out the distribution of fragments with different sizes.

The prepared DNA template libraries were bound to the Sequel Polymerase 2.0 using Sequel Binding Kit 2.0 (PacBio p/n 100-862-200) in preparation for sequencing on the Sequel System. Finally, a DNA polymerase/template complex formed according to the manufacturer's instructions. The enrichment of the larger fragments was improved by the MagBead (PacBio p/n 100-125-900) method. The long-insert size (20 kb) library was sequenced on PacBio Sequel platform with Sequel SMRT cells 1M v2 (PacBio p/n101–008-000), which has one movie of 600 minutes per Sequel SMRT cell at the Genome Center of Nextomics (Wuhan, China). A total of 7 Sequel SMRT cells were processed and the raw data were filtered based on the sequencing platform with the default parameters to remove low-quality bases or reads with adapters. In total, 50.15 Gb of long reads clean data were obtained (Table S1). The average length and the N50 of long subreads were 10.51 kb and 15.97 kb respectively.

Hi-C libraries were constructed for *A. grahami* according to the improved Hi-C procedures [49]. After treated with 1% formaldehyde solution in PBS buffer at room temperature for 10 minutes to induce crosslinking, the single cell was made by trituration and filtration. The reaction was quenched by adding 2.5M glycine to 0.2M

solution for 5 minutes. Nuclei were digested with 100 units of MboI, marked by biotin-14-dCTP (Invitrogen), and then ligated by T4 DNA Ligase. After reversal of crosslinks, ligated DNA was purified and sheared to a length of 300-600 base pairs, at which point ligation junctions were pulled down by streptavidin beads and prepared for high-throughput sequencing. Sequencing was performed using the Illumina NovaSeq 6000 Sequencing System (San Diego, CA, USA) with PE150, yielding 74.24 Gb raw data (Table S1).

Genome survey and Genome assembly

The genome size was estimated based on the equation $G = k_{num} / k_{depth}$, where the k_{num} was the total number of 17-mers, k_{depth} denoted the peak frequency of 17-mers estimated, and G represented the estimated genome size. Using Jellyfish (v2.1.3, RRID: SCR 005491) [50], the number of 17-mers was counted as 29,131,491,603 from short clean reads, and the k_{depth} was 50. Therefore, the genome size of *A. grahami* was estimated as 582.63 Mb according to the above equation and the heterozygosity rate of the *A. grahami* genome was approximately 2.5% (Table S2, Fig. S1). FALCON is specifically designed to perform *de novo* assembly for PacBio long reads with about 15% random errors [51]. After correction with FALCON (v0.4), the PacBio long reads were assembled with Wtdbg (v1.2.8) [52, 53], obtaining an initial assembly with the length of approximately 596.65 Mb and N50 contig of 1.93 Mb. To further improve the accuracy of the reference assembly, following steps of polishing strategies were performed for the initial assembly. The pbalign (v.0.3.0) with default parameters was used for Quiver error-correction, generating an error-corrected genome assembly of PacBio long reads. We used BWA (v0.7.12, RRID: SCR 010910) to map short reads to the error-corrected assembly. Then it was polished with Pilon

(v1.21, RRID: SCR 014731) to generate the second iteration of the assembled genome [54]. Finally, we obtained a polished assembly genome with size of 600.09 Mb, including N50 contig of 1.93 Mb and 1604 contigs (Table 1, Table S3). So far, the present genome has the longest N50 contig length among all the published genome assemblies of calyptratae flies of Diptera.

Table. 1 An overview comparison of genome assembly and structure features in five calyptratae flies of Diptera.

Parameter	*A. grahami*	*L. cuprina*	*G. morsitans*	*M. domestica*	*P. regina* (♀)
Sequencing platform	PacBio	Illumina	454/Illumina	Illumina	454/PacBio
Genome size (Mb)	600	458	366	692	550
No.of contigs/Scaffolds	1,604/7	74,043/4,436	-/13,807	-/20487	192,662/-
Contig N50 (kb)	1930	744.4	50	12	7.9
GC level (%)	31	29.3	34.1	35.1	26.2
Repetitive regions (%)	48.02	57.8	-	55	8.11
Function annotation (gene number; %)	12,791; 99.8	12,160; 83.6	12,308; 99.5	14180;92.3	7792;94
Sequencing depth	86×	100×	160×	90×	44×
Completeness (BUSCO/CEGMA; %)	99.2	96	99	98	93.6

Genome completeness was assessed by BUSCO or CEGMA. Four genomes of calyptratae fly species were selected, as *L. cuprina* [55], *G. morsitans* [56], *M. domestica* [57] and *P. regina* [45]. The genome version of *Ph. regina* female adult was chosen.

For the *A. grahami* genome, the assembly genome size (600 Mb) was almost the same as the genome size (582.63 Mb) estimated in 17-mer analysis. The sequencing quality was checked and the potential contaminated contigs from other species were removed based on the GC content and the depth of coverage of the genome assembly that analyzed by the GC Depth analysis. The completeness of the assembly was evaluated by BUSCO (v3.0, RRID: SCR 015008). The result of BUSCO analysis

indicated that our assembly covered 99.2% complete and 0.7% partial insect BUSCOs, with only 0.5% missed (Table S4).

We also performed flow cytometry with propidium iodide staining to estimate the genome size of *A. grahami*. *Drosophila melanogaster* (strain w118) was used as the internal control with DNA content (pictogram: pg, 1 pg = 978 Mb) of 1C = 0.18 pg (175 Mb) [58]. The samples were prepared following the procedure of the previous study [59]. The flow-cytometry was carried out using Accuri C6 (BD, USA) with a 488nm laser. Data were processed by FlowJo software (v7.6) (Fig. S2). The estimated genome sizes of male (679.2 ± 7.582 Mb, N = 6) and female (696.4 ± 6.618 Mb, N = 6) have no significant difference (P-value = 0.1183), showing no sexual dimorphism. However, it is about 18.1% larger than the K-mer based genome size (582.63 Mb), and 14.6% larger than the assembly genome size (600.09 MB).

Functional Prediction and Genome Annotation

Analysis of repeat genes

Simple sequence repeats (SSRs) are repeating sequences of 1-6 base pairs of DNA that exist extensively in genomes. SSRs in the blow fly genome were identified using the MIcroSAtellite Identification Tool (MISA, RRID: SCR 010765) [60]. MISA can distinguish and locate simple and complicated SSRs. The latter is always inserted by a certain number of nucleic acid bases. In total, 322266 SSRs were found in the *A. grahami* genome.

We also analyzed the repetitive sequences in the *A. grahami* genome including in tandem repeats and transposable elements (TEs). A Tandem Repeats Finder (TRF, v4.09) was used to annotate the tandem repeats [61]. A combination of *de novo* and homology-based approach was utilized to identify TEs at both the DNA and protein

levels. First, we used RepeatModeler (v1.0.8, RRID: SCR 015027) [62] to construct a *de novo* repeat DNA library, which built a repeat consensus database with classification information. The similar TEs were then searched against the known Pepbase library (Repbase 23.08) and *de novo* based repeat library using RepeatMasker (v4.0.6, RRID: SCR 012954) [62]. RepeatProteinMask within the RepeatMasker package was used to search against the TE protein database using a WU_BLASTX engine.

Overall, *A. grahami* genome comprised 48.02% repetitive sequences, of which 43.69% were TEs. DNA with repetitive sequence accounted for 11.65% of the *A. grahami* genome, representing the most abundant repeat class (Table 2).

Table 2 Statistics of repeat sequence analysis

Type	RepeatMasker		LTR finder		RepeatProteinMask		RepeatModeler		Combined TEs	
	Length (Mb)	% in genome	Length (Mb)	% in genome	Length (Mb)	% in genome	Length (Mb)	% in genome	Length (Mb)	% in genome
DNA	42174497	7.03	0	0	41341346	6.89	50464704	8.41	69933653	11.65
LINE	10505716	1.75	0	0	19838372	3.31	26169690	4.36	34333817	5.72
LTR	4789075	0.8	15966332	2.66	5778730	0.96	1229900	0.2	21249831	3.54
SINE	51914	0.01	0	0	0	0	453547	0.08	446000	0.07
Other*	12169475	2.02	0	0	7698698	1.28	50424873	8.4	78096062	13.02
Unknown*	161876	0.03	0	0	0	0	50424873	16	84103655	14.02
Total	69852553	11.64	15966332	2.66	74657146	12.44	224757686	37.45	288163018	48.02

***Other represents sequences with annotation but not belonging to the above types of repetitive genes, such as satellites, simple repeats, retroposon, artefact, helitron and low complexity repeats; unknown represents sequences that cannot be classified.**

Gene prediction and functional annotation

The protein-coding genes in the *A. grahami* genome assembly were identified using *de novo*-based, homology-based and RNA-seq-based gene prediction methods. Augustus (v2.4, RRID: SCR 008417) [63], GlimmerHMM (v3.0.4, RRID: SCR

002654) [64], Genemark (RRID: SCR 011930) [65] and SNAP (RRID: SCR 002127) [66], all trained for the *D. melanogaster* gene model before the gene prediction [67], were used in the *de novo*-based gene prediction with default parameters. GeMoMa (v1.3.1) was used to perform the annotation of protein coding based on the annotation of genes of *D. melanogaster*, *Glossina austeni*, *Lucilia cuprina*, *Stomoxys calcitrans* and *Musca domestica* from GenBank (Table S5) [68]. PASA (v2.0.2, RRID: SCR 014656) was used to perform the RNA-seq-based gene prediction [69]. Finally, the results from the three approaches were integrated using EVidenceModeler (EVM) (v1.1.1, RRID: SCR 014659) [69]. When conducting the EVM integration, PASA-predicted transcripts from unigenes and GeMoMa-predicted homologous transcripts were given higher weights than the *de novo* predicted transcripts. The gene set was aligned to the transposon database by TransposonPSI (v08222010) with default parameters [70]. Any gene of homology to transposons was removed from the final gene set. In total, 12823 protein-coding genes were identified in *A. grahami* genome, with an average of 13240.43 bp in length, 4.62 exons per gene (Table S6).

Gene functions of the predicted protein-coding genes were annotated using two strategies. First, those predicted protein sequences were aligned to Swiss-Prot and TrEMBL protein databases using Blastall with the best match parameters [71]. The pathways of the predicted genes sequences were extracted from the KEGG Automatic Annotation Server (v2.1) [72]. Then the annotation of motifs and domains was achieved by using InterProScan (v5.24, RRID: SCR 005829) to search the open databases including Pfam (32.0, RRID: SCR 004726), ProDom (v2006.1, RRID: SCR 006969), PRINTS (v42.0, RRID: SCR 003412), PANTHER (v12.0, RRID: SCR 004869), SMRT (v7.1), and PROSITE (v2018_02, RRID: SCR 003457) [73, 74]. The results of the two parts were combined to form the final data set. In summary, 12791

genes were annotated with at least 1 related function, which accounted for about 99.8% of predicated protein-coding genes (12823) of *A. grahami* (Table 3). Additionally, the annotation of non-coding RNA genes set was also performed based on the RNA-seq data of *A. grahami* transcriptome data (6.6G). The rRNA, snRNA and miRNA were annotated using the non-coding database Rfam (v14.0, RRID: SCR 007891). Then the tRNA sequence was annotated using tRNAscan-SE (v2.0, RRID: SCR 010835) [75]. The rRNA and subunits were predicted by RNAmmer (v1.2) [76]. As a result, a total of 126 miRNAs, 21 rRNAs, 192 snRNAs and 859 tRNAs genes were annotated (Table S7).

Table 3 Function annotation of protein coding genes of *A. grahami*

	Type	Number	Percent (%)
**Annotation**	Swiss-Prot	9648	75.2
	TrEMBL	12721	99.2
	KEGG	5247	40.9
	KOG	8252	64.4
	GO	7518	58.6
	InterProScan	10488	81.8
	Nr*	12780	99.7
**Total**	Annotated	12791	99.8
	Gene	12823	-

*Nr: Non-Redundant Protein Sequence Database

Evolutionary analyses

Gene family and phylogenetic analyses

For the prediction of gene family, several species were selected based on genomic models, classification background, feeding habits or life-styles such as necrophagia, polyphagia, parasitism or hematophagia. The genomic resource of *D.*

melanogaster, Lucilia cuprina, Musca domestica, Stomoxys calcitrans, Glossina austeni, Onthophagus taurus, Nicrophorus vespilloides, Blattella germanica, Cimex lectularius, Aedes aegypti were used (Table S5) [67, 77-85]. The OrthoMCL (RRID: SCR 007839) was employed to identify the gene families [86]. First, the amino acid sequence of the longest transcript of each gene was selected from *A. grahami* and other selected insect species. Then it was aligned reciprocally with the BLASTP (RRID: SCR 001010) plug-in on NCBI with a threshold of e-value less than $1e^{-5}$. After that, the alignment results were clustered into family groups with default parameters. Finally, the orthologous gene families from each selected species were identified (Fig. 2). According to the results, *A. grahami* genome contains the fewest unique genes and gene families compared to other 10 species used in the analysis (Table 4).

Figure 2. Gene family comparison between *A. grahami* and other insect species

In total, 2989 single-copy gene families were identified among these 11 species. First, each gene family was aligned using the MAFFT program (v7) at the amino-acid level [87]. All the sequence alignments were then reversely translated to nucleotide sequences. The poorly aligned positions and divergent regions were subsequently trimmed with Gblocks (v0.91, RRID: SCR 015945). Then, RAxML (v8.2.11, RRID: SCR 006086) was used to construct phylogenetic trees using the GTR+GAMMA model for nucleotide sequences [88] with 100 bootstrap replicates assessing the branch reliability of RAxML. *C. lectularius* was set as the outgroup.

Table 4 Genome families of *A. grahami* and other insect species

Species	Genes Number	Genes number In Families	Unclustered Genes number	Family Number	Unique Families Number	Average Genes Per Family

A.aegypti	14539	12810	1729	8701	485	1.47
A.grahami	12823	12033	790	10424	53	1.15
B.germanica	28670	19323	9347	9449	1286	2.04
C.lectularius	11890	9743	2147	8104	250	1.2
D.melanogaster	13872	11469	2403	9694	235	1.18
G.austeni	19722	12205	7517	9867	350	1.24
L.cuprina	15232	13915	1317	11364	560	1.22
M.domestica	14236	12968	1268	10713	133	1.21
N.vespilloides	12385	10948	1437	8961	164	1.22
O.taurus	14374	12674	1700	9222	372	1.37
P.regina (F)	8312	7536	776	6670	18	1.13
P.regina (M)	9490	7781	1709	6838	34	1.14
S.calcitrans	13469	12411	1058	10445	115	1.19

Unclustered genes and unique families represent the specific genes and families corresponding to each species.

In addition, 9 selected species were separated into different groups based on their dietary habits such as necrophagia, coprophagia, hematophagia and polyphagia (Table S8). Orthologous genes of each species were also separated as a single assemblage. The shared orthologous genes of clusters of *A. grahami* with other Diptera species and other non-Diptera species were displayed using online Draw Venn Diagram [89]. The results may provide candidate genes for the future research of the necrophagous life-style of *A. grahami* (Fig. 3).

Figure 3. Venn diagram of orthologous gene families. (A) The intersection between *A. grahami* and other Diptera species. (B) The intersection between *A. grahami* and other non-Diptera species with different diet habits.

Divergence time and gene family expansion / contraction

The estimation of divergence time was based on the results of the gene family clustering. Four-fold degenerate sites were extracted from the alignment of coding sequences of 2989 identified single-copy gene families. The PAML MCMCTree

program (v4.5, RRID: SCR 014932) was used to estimate divergence times with the calculation of approximate likelihood test, molecular clock and substitution model of REV [90]. The primary parameters of MCMCTree were set as clock = 2 (an independent rates model following a log-normal distribution), RootAge = < 4 (400 Myr for a calibration on the root of the phylogenetic tree) , model = 7 (the substitution model, REV), BDparas = 110 (default value was used here, parameters controlling the birth-death process), kappa_gamma = 62 (transition/transversion rate ratio), alpha_gamma = 11 (gamma shape parameter for variable rates among sites), rgene_gamma = 23.606 (Dirichlet-gamma prior for the mean substitution rate), sigma2_gamma = 11.03 (Dirichlet-gamma prior for the rate drift parameter). Calibrations of fossil evidence were retrieved from the TimeTree database to infer the evolutionary timescale [91].

In the phylogenetic analysis, *A. grahami* and *L. cuprina* were clustered together at first. Then with *P. regina*, it was clustered into the branch of Calliphoridae, which is next to the family Muscidae represented by *M. domestica* and *S. calcitrans*. This result is consistent with the blow fly species taxonomy that *A. grahami* diverged with *L. cuprina* from the common ancestor around 26 million years ago (Fig. 4).

Figure 4. The estimation on divergence times. The numbers besides the dots of topological branches are the divergent time to present-day (Million years ago, Mya). Red dots represent the calibration time from fossil evidence. The right lists each family name.

To further explore the gene family change under natural selection pressure, the expansion and contraction of gene families were identified using CAFE program (RRID:SCR 005983) [92]. The result revealed 102 expanded and 280 contracted gene families in *A. grahami* genome. Additionally 198 gene families were lost from the

genome (Table S9, Fig. S3).

Analysis of whole-genome duplication (WGD)

We used four-fold synonymous third-codon transversion (4DTv) [93] and Ks (a measure of synonymous substitution rate) estimation [94] to detect WGD events in *A. grahami* genome. To this end, paralogous sequences of *A. grahami*, *Bombyx mori* and *D. melanogaster*, were identified with OrthoMCL [86]. Then, protein sequences of these insects were aligned against each other with BLASTP (using an E-value threshold of $\leq 1e^{-5}$) to identify conserved paralogs in each species. Finally, potential WGD events in each genome were evaluated based on their 4DTv and Ks distribution. The WGD analysis suggested that *A. grahami* may have experienced the same recent WGD events as *B. mori* (Fig. S4).

Chromosome assembly using Hi-C data

To generate a chromosomal level assembly of the genome, Hi-C fragment libraries were constructed. The Hi-C libraries were sequenced on the Illumina NonaSeq 6000 (Illumina, CA, USA), generating 495 million Hi-C paired-end reads. After low-quality sequences (quality scores $\leq$ 15), adaptor sequences, and sequences shorter than 30 bp were filtered out using fastp (v0.12.6, RRID: SCR 016962) [95], the clean paired-end reads were mapped to the draft assembled sequence by bowtie2 (v.2.3.2, RRID: SCR 005476) [96] to get the unique mapped paired-end reads. As a result, 102 million uniquely mapped paired-end reads were generated, of which 62.26% were valid interaction pairs (Table S10). Combined with the valid Hi-C data, we subsequently utilized the LACHESIS *de novo* assembly pipeline to produce

chromosome-level scaffolds. As shown in Figure 5, the assembled sequence was anchored onto the 6 pseudo-chromosomes with lengths ranging from 57.97 to 112.16 Mb (Table S11). The assembled pseudo-chromosomes (578,212,361 bp) accounted for 96.4% of the genome sequences (600,090,062 bp), with scaffold N50 values of 104.65 Mb N50 scaffold (Table S3).

Figure 5. Hi-C interaction matrix maps within and among 6 chromosomes. The contact density was illustrated by the color bar with red (high density) to white (low density).

The similarity between *A. grahami* genome and the published fruit fly (*D. melanogaster*) genome was analyzed [67]. The protein-coding genes from each genome were aligned using BLASTP with threshold of E-value less than $1e^{-10}$. Then the results were combined with the GFF format files of the two genomes using MCScanX [97].

The collinearity between *A. grahami* and *D. melanogaster* genome was shown in Fig.6A. The pseudochromosomes of *A. grahami* and the corresponding Muller elements of *D. melanogaster* were listed (Table S11). The Muller F was reported as the X-chromosome linked in some calyptratae species [98, 99]. But in present study, it is hard to distinguish which assembled chromosome of *A. grahami* should be the Muller F according to the collinearity analysis. Further effort should be made to determine the sex chromosome of *A. grahami*.

In addition, we investigated the distributions of long terminal repeat (LTR), gene family expansion or contraction, and genes under positive selection on the genome using a window size of 1 Mb across each chromosome and plotted the distributions in

Fig. 6B by Circos (RRID: SCR 011798). There was no enrichment of genes for any particular chromosomes. All the chromosomes contain a gene density of around 20 genes/per Mb. However, the results showed that longer chromosomes tend to contain higher number of LTR, except for the case of Chr05. In addition, we noticed that the LTR were enriched on the specific regions of each chromosome where it could represent the centromere locations (Table S11).

Figure 6. Collinerity and gene clustering of *A. grahami* genome. (A) Collinear relationship between *A. grahami* and *D. melanogaster* genomes. The blue bar represents *A. grahami* genome and the grey one represents fruit fly genome. (B) Gene density distribution on chromosomes of *A. grahami*. The outer blue circle indicates the chromosomes. The inner yellow, light blue, green and orange circle represent the LTR, expanded gene family, contracted gene family and positive selected gene respectively. Window size = 1 Mb.

Conclusion

In this study, we have successfully assembled the robust draft genome of *A. grahami* through long reads *de novo* technology and Hi-C sequencing technology using the PacBio Sequel sequencing platform. This reference genome is the first chromosome-level genome assembly in calyptratae which will facilitate further genomic research of other fly species of forensic importance, and promote the transition from forensic genetics to forensic genomics [48]. This draft genome resource will be a helpful tool for advancing the study on the evolution of *A. grahami* genome. It will deepen our understanding of *A. grahami*'s unique biological characteristics, such as low-temperature tolerance, seasonal distribution, necrophagous dietart habit, and its intrusion into other regions of the world. Based on qualified genome resource, studies of forensically important blow fly species will reinforce the reliability of entomological evidence and promote its application in the legal criminal investigation [100].

Availability of supporting data

1. Genome and transcriptome are available in the NCBI SRA database (project accession: **PRJNA513084, SRA: SRX5207346**).

2. Voucher samples information of present work was list in Table S12.

Additional flies

Additional File Figure S1. 17-mer Depth Distribution Curve. The x-axis represents the k-mer depth; the y-axis represent k-mer depth frequency; *Arabidopsis thaliana* (Atha for short) was set as reference.

Additional File Figure S2. Estimation of genome size of *A. grahami* by flow cytometry. Genome size (bp) was calculated from DNA content (pg) following the formula: GAg= (FAg/FDm) × GDm. GAg, DNA content of *A. grahami*, GDm, DNA content of *D. melanogaster*, FAg, fluorescence value of *A. grahami*, FDm, fluorescence value of *D. melanogaster*.

Additional File Figure S3. Expansion and contraction at the gene family level. Branch length represents divergent time; Pie chart illustrates percentage of expansion and contraction; '+/-'means gene gain / loss.

Additional File Figure S4. Whole-genome duplication analysis of *A. grahami*, *B. mori* and *D. melanogaster*.

Additional File Table S1. Information of sequencing platform and output data.

Additional File Table S2. Genome size estimation and Heterozygosity based on 17 k-mer.

Additional File Table S3. Statistics results of genome assembly correction.

Additional File Table S4. Assessment on assembly completeness.

Additional File Table S5. Genome resource of 10 insect species for comparable genomics analysis.

Additional File Table S6. Comparison of *A. grahami* and other fly species on protein coding genes structure and statistics.

Additional File Table S7. Functional annotation of non-coding RNA genes.

Additional File Table S8. Diet habit of 9 selected insect species.

Additional File Table S9. Statistics of gene family expansion and contraction

Additional File Table S10. Statistics of the Hi-C assembly of the *A. grahami* genome.

Additional File Table S11. Genome-wide characteristic on pseudochromosomes of the *A. grahami*.

Additional File Table S12. Information of voucher samples used in present study.

Abbreviations

PMImin: minimum postmortem interval; *COI*: cytochrome oxidase gene I; CTAB: Cetyltrimethyl Ammonium Bromide; WGS: Whole-genome shotgun sequencing; Hi-C: Chromosome conformation capture; SSR: Simple sequence repeats; TEs: Transposable elements; TRF: Tandem Repeats Finder; LTR: long terminal repeat; Mya: Million years ago; 4DTv: four-fold synonymous third-codon transversion; BUSCO: benchmarking universal single-copy orthologs; GO: gene ontology; KEGG: Kyoto Encyclopedia of Genes and Genomes; SMRT: single-molecule real time; WGD: Whole-genome duplication

Competing interests

All authors declare that no competing interests.

Funding

The present study was supported by grant of the National Natural Science Foundation of China (81571855) and Science Foundation of Hunan Province (2017SK2015).

Author contributions

F. M., J.C. designed the project. F. M., M. Z., Y. W., and C.C. analyzed the data. H.H., Z. L., Y. J. prepared the samples and conducted the experiments. F. M., D.F., Z. S., wrote and revised the manuscript. J.C. supervised the whole program and coordinated the group. Y. G. provided material and equipment for breeding of insects.

Reference

1. Catts EP, Goff ML. Forensic entomology in criminal investigations. Annual review of entomology. 1992;37:253-72. doi:10.1146/annurev.en.37.010192.001345.
2. Benecke M. A brief history of forensic entomology. Forensic science international. 2001;120 1-2:2-14.
3. Schoenly KA. statistical analysis of successional patterns in carrion-arthropod assemblages: implications for forensic entomology and determination of the postmortem interval. Journal of forensic sciences. 1992;37 6:1489-513.
4. Tomberlin JK, Mohr R, Benbow ME, Tarone AM, VanLaerhoven S. A roadmap for bridging basic and applied research in forensic entomology. Annual review of entomology. 2011;56:401-21. doi:10.1146/annurev-ento-051710-103143.
5. Benecke M, Lessig R. Child neglect and forensic entomology. Forensic science international. 2001;120 1-2:155-9.
6. Campobasso CP, Gherardi M, Caligara M, Sironi L, Introna F. Drug analysis in blowfly larvae and in human tissues: a comparative study. International journal of legal medicine. 2004;118 4:210-4. doi:10.1007/s00414-004-0448-1.
7. Castner LC, Byrd JH. Insects of Forensic Importance. In: Castner LC, Byrd JH, editor. Forensic entomology : the utility of arthropods in legal investigations. Boca Raton, London: CRC Press; 2009. p. 44-6.
8. Anderson GS. Minimum and maximum development rates of some forensically important Calliphoridae (Diptera). Journal of forensic sciences. 2000;45 4:824-32.
9. Harvey ML, Gaudieri S, Villet MH, Dadour IR. A global study of forensically significant

calliphorids: Implications for identification. Forensic science international. 2008;177 1:66-76. doi:10.1016/j.forsciint.2007.10.009.

10. Norris KR. The Bionomics of Blow Flies. Annreventomol. 1965;10 1:47-68.

11. Baumgartner DL, Greenberg B. The Genus Chrysomya (Diptera: Calliphoridae) in the New World. Journal of medical entomology. 1984;21 1:105-13.

12. Tarone AM, Sanford MR. Is PMI the Hypothesis or the Null Hypothesis? Journal of medical entomology. 2017;54 5:1109-15. doi:10.1093/jme/tjx119.

13. Tarone AM, Picard CJ, Spiegelman C, Foran DR. Population and temperature effects on Lucilia sericata (Diptera: Calliphoridae) body size and minimum development time. Journal of medical entomology. 2011;48 5:1062-8.

14. Zhao B, Wen C, Qi LL, Wang H, Wang J. [Biological characteristics of calliphoridae and its application in forensic medicine]. Fa yi xue za zhi. 2013;29 6:447-50.

15. Fan ZD. Key to the common flies of China. Beijing, China: Science publishing house; 1992.

16. Aldrich JM. New two-winged flies of the family Calliphoridae from China. Proceedings of the United States National Museum. 1930.

17. Dodge HR. Identifying common flies. Public health reports. 1953;68 3:345-50.

18. Nunez-Vazquez C, Tomberlin J, Garcia-Martinez O. First Record of the Blow Fly Calliphora grahami from Mexico. Southwestern Entomologist. 2010;35 3:313-6. doi:Doi 10.3958/059.035.0310.

19. Whitworth T. Keys to the genera and species of blow flies (Diptera : Calliphoridae) of America North of Mexico. P Entomol Soc Wash. 2006;108 3:689-725.

20. Wang Y, Zhang YN, Liu C, Hu GL, Wang M, Yang LJ, et al. Development of Aldrichina grahami (Diptera: Calliphoridae) at Constant Temperatures. Journal of medical entomology. 2018;55 6:1402-9. doi:10.1093/jme/tjy128.

21. Chen W, Yang L, Ren L, Shang Y, Wang S, Guo Y. Impact of Constant Versus Fluctuating Temperatures on the Development and Life History Parameters of Aldrichina grahami (Diptera: Calliphoridae). Insects. 2019;10 7 doi:10.3390/insects10070184.

22. Kurahashi H, Kawai S, Shudo C, Wada Y. Seasonal prevalence of adult fly and life cycle of aldrichina grahami (aldrich) in tokyo. Medical Entomology & Zoology. 1984;35 3:261-7.

23. Guo YD, Cai JF, Tang ZC, Feng XO, Lin Z, Yong F, et al. Application of Aldrichina grahami (Diptera, Calliphoridae) for forensic investigation in central-south China. Romanian Journal Of Legal Medicine. 2011;19 1:55-8. doi:10.4323/rjlm.2011.55.

24. Wang JF, Hu C, Min JX, Chen YC, Li JT. Chronometrical morphology of Aldrichina grahami and its application in the determination of postmortem interval. Acta Entomol Sin. 2002;45 2002:265-70.

25. Kurahashi H, Kawai S, Shudo C. Seasonal migration of Japanese blow flies, Aldrichina grahami (Aldrich) and Calliphora nigribarbis Vollenhoven, observed by a mark and recapture method on Hachijo Island, Tokyo. Medical Entomology & Zoology. 1991; 42:57-9.

26. Zehner R, Amendt J, Krettek R. STR typing of human DNA from fly larvae fed on decomposing bodies. Journal of forensic sciences. 2004;49 2:337-40.

27. Li K, Ye GY, Zhu JY, Hu C. Detection of food source by PCR analysis of the gut contents of Aldrichina grahami (Aldrich) (Diptera: Calliphoridae) during post-feeding period. Insect Sci. 2007;14 1:47-52. doi:10.1111/j.1744-7917.2007.00124.x.

28. Xu H, Ye GY, Xu Y, Hu C, Zhu GH. Age-dependent changes in cuticular hydrocarbons of larvae

in Aldrichina grahami (Aldrich) (Diptera: Calliphoridae). Forensic science international. 2014;242:236-41. doi:10.1016/j.forsciint.2014.07.003.

29. Moore HE, Adam CD, Drijfhout FP. Potential Use of Hydrocarbons for Aging Lucilia sericata Blowfly Larvae to Establish the Postmortem Interval. Journal of forensic sciences. 2013;58 2:404-12. doi:10.1111/1556-4029.12016.

30. Liu YL. A case report of gastrointestinal myiasis caused by Aldrichina grahami. Acta Medicinae Universitatis Scientiae et Technologiae Huazhong. 1980;2:81-2.

31. Li XL, Xu ZQ. A case of human gastrointestinal myiasis. Bulletin of Disease Control & Prevention. 2006;21 1:107.

32. Cao XL, Sang YH, Yang YL, Wang S. Comprehensive analyses on Chinese human myiasis cases of 2003-2013. Guide of China Medicine. 2015;8:37-9.

33. Lachish T, Marhoom E, Mumcuoglu KY, Tandlich M, Schwartz E. Myiasis in Travelers. Journal of travel medicine. 2015;22 4:232-6. doi:10.1111/jtm.12203.

34. Sawabe K, Hoshino K, Isawa H, Sasaki T, Hayashi T, Tsuda Y, et al. Detection and isolation of highly pathogenic H5N1 avian influenza A viruses from blow flies collected in the vicinity of an infected poultry farm in Kyoto, Japan, 2004. The American journal of tropical medicine and hygiene. 2006;75 2:327-32.

35. Miura K, Takaya T, Koshiba K. The effect of biotin deficiency on the biosynthesis of the fatty acids in a blowfly, Aldrichina grahami during metamorphosis under aseptic conditions. Archives internationales de physiologie et de biochimie. 1967;75 1:65-76.

36. Tohoru H, Akira W, Kazuo Miura. Properties and regulation of xanthine dehydrogenase of a blowfly, Aldrichina grahami. Insect Biochemistry, 1977, 7(4):317-322. 1977;7 4:317-22.

37. Wadano A MK. Urate oxidase in the blowfly, Aldrichina grahami. Insect Biochemistry. 1976;6 3:321-5.

38. Wadano A, Miura, K, Ihara, H, Kondo N, Taniguchi, MA. Purification and some properties of isocitrate dehydrogenase of a blowfly aldrichina grahami. Comparative Biochemistry and Physiology B. 1989;94 1:189-94.

39. Meng FM, Ren LP, Wang Z, Deng J, Guo YD, Chen C, et al. Identification of Forensically Important Blow Flies (Diptera: Calliphoridae) in China Based on COI. Journal of medical entomology. 2017;54 5:1193-200. doi:10.1093/jme/tjx105.

40. Zaidi F, Wei SJ, Shi M, Chen XX. Utility of multi-gene loci for forensic species diagnosis of blowflies. Journal of insect science. 2011;11.

41. Park SH, Park CH, Zhang Y, Piao H, Chung U, Kim SY, et al. Using the Developmental Gene Bicoid to Identify Species of Forensically Important Blowflies (Diptera: Calliphoridae). BioMed research international. 2013; doi:Artn 53805110.1155/2013/538051.

42. Zhu ZY, Liao HD, Ling J, Guo YD, Cai JF, Ding YJ. The complete mitochondria genome of Aldrichina grahami (Diptera: Calliphoridae). Mitochondrial DNA B. 2016;1:107-9. doi:10.1080/23802359.2015.1137847.

43. Gallagher MB, Sandhu S, Kimsey R. Variation in developmental time for geographically distinct populations of the common green bottle fly, Lucilia sericata (Meigen). Journal of forensic sciences. 2010;55 2:438-42. doi:10.1111/j.1556-4029.2009.01285.x.

44. Hu Y, Yuan X, Zhu F, Lei C. Development time and size-related traits in the oriental blowfly, chrysomya megacephala along a latitudinal gradient from china. Journal of Thermal Biology. 2010;35 7:366-71.

45. Andere A, Platt RN, Ray DA and Picard CJ. Genome sequence of Phormia regina Meigen (Diptera: Calliphoridae): implications for medical, veterinary and forensic research. BMC genomics. 2016;17 1:842. doi:10.1186/s12864-016-3187-z.
46. Zajac BK, Amendt J, Verhoff MA, Zehner R. Dating Pupae of the Blow Fly Calliphora vicina Robineau-Desvoidy 1830 (Diptera: Calliphoridae) for Post Mortem Interval-Estimation: Validation of Molecular Age Markers. Genes. 2018;9 3 doi:Artn 15310.3390/Genes9030153.
47. Arenas M, Pereira F, Oliveira M, Pinto N, Lopes AM, Gomes V, et al. Forensic genetics and genomics: Much more than just a human affair. PLoS genetics. 2017;13 9:e1006960. doi:10.1371/journal.pgen.1006960.
48. Kayser M, Parson W. Transitioning from Forensic Genetics to Forensic Genomics. Genes. 2017;9 1 doi:10.3390/genes9010003.
49. Rao SSP, Huntley MH, Durand NC, Stamenova EK, Bochkov ID, Robinson JT, et al. A 3D Map of the Human Genome at Kilobase Resolution Reveals Principles of Chromatin Looping. Cell. 2014;159 7:1665-80. doi:10.1016/j.cell.2014.11.021.
50. Marcais G, Kingsford C. A fast, lock-free approach for efficient parallel counting of occurrences of k-mers. Bioinformatics. 2011;27 6:764-70. doi:10.1093/bioinformatics/btr011.
51. Eid J, Fehr A, Gray J, Luong K, Lyle J, Otto G, et al. Real-time DNA sequencing from single polymerase molecules. Science. 2009;323 5910:133-8. doi:10.1126/science.1162986.
52. WTDBG package： https://github.com/ruanjue/wtdbg. (Accessed 10 Jan 2018).
53. Falcon: https://github.com/PacificBiosciences/FALCON.
54. Walker BJ, Abeel T, Shea T, Priest M, Abouelliel A, Sakthikumar S, et al. Pilon: an integrated tool for comprehensive microbial variant detection and genome assembly improvement. PloS one. 2014;9 11:e112963. doi:10.1371/journal.pone.0112963.
55. Anstead CA, Korhonen PK, Young ND, Hall RS, Jex AR, Murali SC, et al. Lucilia cuprina genome unlocks parasitic fly biology to underpin future interventions. Nature communications. 2015;6:7344. doi:10.1038/ncomms8344.
56. Watanabe J, Hattori M, Berriman M, Lehane MJ, Hall N, Solano P, et al. Genome sequence of the tsetse fly (Glossina morsitans): vector of African trypanosomiasis. Science. 2014;344 6182:380-6. doi:10.1126/science.1249656.
57. Scott JG, Warren WC, Beukeboom LW, Bopp D, Clark AG, Giers SD, et al. Genome of the house fly, Musca domestica L., a global vector of diseases with adaptations to a septic environment. Genome biology. 2014;15 10:466. doi:10.1186/s13059-014-0466-3.
58. Bennett MD, Leitch IJ, Price HJ, Johnston JS. Comparisons with Caenorhabditis (approximately 100 Mb) and Drosophila (approximately 175 Mb) using flow cytometry show genome size in Arabidopsis to be approximately 157 Mb and thus approximately 25% larger than the Arabidopsis genome initiative estimate of approximately 125 Mb. Annals of botany. 2003;91 5:547-57. doi:10.1093/aob/mcg057.
59. Picard CJ, Johnston JS, Tarone AM. Genome sizes of forensically relevant Diptera. Journal of medical entomology. 2012;49 1:192-7. doi:10.1603/me11075.
60. Thiel T, Michalek W, Varshney RK, Graner A. Exploiting EST databases for the development and characterization of gene-derived SSR-markers in barley (Hordeum vulgare L.). TAG Theoretical and applied genetics Theoretische und angewandte Genetik. 2003;106 3:411-22. doi:10.1007/s00122-002-1031-0.
61. Benson G. Tandem repeats finder: a program to analyze DNA sequences. Nucleic acids

research. 1999;27 2:573-80. doi:10.1093/nar/27.2.573.

62. Bedell JA, Korf I, Gish W. MaskerAid: a performance enhancement to RepeatMasker. Bioinformatics. 2000;16 11:1040-1. doi:10.1093/bioinformatics/16.11.1040.

63. Stanke M, Waack S. Gene prediction with a hidden Markov model and a new intron submodel. Bioinformatics. 2003;19 Suppl 2:ii215-25. doi:10.1093/bioinformatics/btg1080.

64. Majoros WH, Pertea M, Salzberg SL. TigrScan and GlimmerHMM: two open source ab initio eukaryotic gene-finders. Bioinformatics. 2004;20 16:2878-9. doi:10.1093/bioinformatics/bth315.

65. Besemer J, Borodovsky M. GeneMark: web software for gene finding in prokaryotes, eukaryotes and viruses. Nucleic acids research. 2005;33 Web Server issue:W451-4. doi:10.1093/nar/gki487.

66. Johnson AD, Handsaker RE, Pulit SL, Nizzari MM, O'Donnell CJ, De Bakker PI. SNAP: a web-based tool for identification and annotation of proxy SNPs using HapMap. Bioinformatics. 2008;24 24:2938-9. doi:10.1093/bioinformatics/btn564.

67. NCBI Genome. https://www.ncbi.nlm.nih.gov/genome/47.

68. Keilwagen J, Wenk M, Erickson JL, Schattat MH, Grau J, Hartung F. Using intron position conservation for homology-based gene prediction. Nucleic acids research. 2016;44 9 doi:ARTN e8910.1093/nar/gkw092.

69. Haas BJ, Salzberg SL, Zhu W, Pertea M, Allen JE, Orvis J, et al. Automated eukaryotic gene structure annotation using EVidenceModeler and the program to assemble spliced alignments. Genome biology. 2008;9 1 doi:Artn R710.1186/Gb-2008-9-1-R7.

70. Yagi M, Kosugi S, Hirakawa H, Ohmiya A, Tanase K, Harada T, et al. Sequence Analysis of the Genome of Carnation (Dianthus caryophyllus L.). DNA Research. 2014;21 3:231-41. doi:10.1093/dnares/dst053.

71. Bairoch A, Apweiler R, Wu CH, Barker WC, Boeckmann B, Ferro S, et al. The universal protein resource (UniProt). Nucleic acids research. 2005;33:D154-D9. doi:10.1093/nar/gki070.

72. Kanehisa M, Goto S. KEGG: Kyoto Encyclopedia of Genes and Genomes. Nucleic acids research. 2000;28 1:27-30. doi:Doi 10.1093/Nar/28.1.27.

73. Hunter S, Apweiler R, Attwood TK, Bairoch A, Bateman A, Binns D, et al. InterPro: the integrative protein signature database. Nucleic acids research. 2009;37:D211-D5. doi:10.1093/nar/gkn785.

74. Zdobnov EM, Apweiler R. InterProScan - an integration platform for the signature-recognition methods in InterPro. Bioinformatics. 2001;17 9:847-8. doi:DOI 10.1093/bioinformatics/17.9.847.

75. Lowe TM, Eddy SR. tRNAscan-SE: a program for improved detection of transfer RNA genes in genomic sequence. Nucleic acids research. 1997;25 5:955-64. doi:10.1093/nar/25.5.955.

76. Lagesen K, Hallin P, Rodland EA, Staerfeldt HH, Rognes T, Ussery DW. RNAmmer: consistent and rapid annotation of ribosomal RNA genes. Nucleic acids research. 2007;35 9:3100-8. doi:10.1093/nar/gkm160.

77. NCBI Genome. https://www.ncbi.nlm.nih.gov/genome/12732

78. NCBI Genome. https://www.ncbi.nlm.nih.gov/genome/14461.

79. NCBI Genome. https://www.ncbi.nlm.nih.gov/genome/11278

80. NCBI Genome. https://www.ncbi.nlm.nih.gov/genome/11279.

81. NCBI Genome. https://www.ncbi.nlm.nih.gov/genome/12827.

82. NCBI Genome. https://www.ncbi.nlm.nih.gov/genome/13223.
83. NCBI Genome. https://www.ncbi.nlm.nih.gov/genome/16689.
84. NCBI Genome. https://www.ncbi.nlm.nih.gov/genome/40824.
85. NCBI Genome. https://www.ncbi.nlm.nih.gov/genome/44.
86. Li L, Stoeckert CJ, Roos DS. OrthoMCL: Identification of ortholog groups for eukaryotic genomes. Genome research. 2003;13 9:2178-89. doi:10.1101/gr.1224503.
87. Katoh K, Standley DM. MAFFT multiple sequence alignment software version 7: improvements in performance and usability. Molecular biology and evolution. 2013;30 4:772-80. doi:10.1093/molbev/mst010.
88. Stamatakis A. RAxML-VI-HPC: Maximum likelihood-based phylogenetic analyses with thousands of taxa and mixed models. Bioinformatics. 2006;22 21:2688-90. doi:10.1093/bioinformatics/btl446.
89. Draw Venn Diagram. http://bioinformatics.psb.ugent.be/webtools/Venn/.
90. Yang Z. PAML: a program package for phylogenetic analysis by maximum likelihood. Computer applications in the biosciences : CABIOS. 1997;13 5:555-6.
91. Hedges SB, Dudley J, Kumar S. TimeTree: a public knowledge-base of divergence times among organisms. Bioinformatics. 2006;22 23:2971-2. doi:10.1093/bioinformatics/btl505.
92. De Bie T, Cristianini N, Demuth JP, Hahn MW. CAFE: a computational tool for the study of gene family evolution. Bioinformatics. 2006;22 10:1269-71. doi:10.1093/bioinformatics/btl097.
93. Kimura M. A simple method for estimating evolutionary rates of base substitutions through comparative studies of nucleotide sequences. Journal of molecular evolution. 1980;16 2:111-20.
94. Blanc G, Wolfe KH. Widespread paleopolyploidy in model plant species inferred from age distributions of duplicate genes. The Plant cell. 2004;16 7:1667-78. doi:10.1105/tpc.021345.
95. Chen S, Zhou Y, Chen Y, Gu J. fastp: an ultra-fast all-in-one FASTQ preprocessor. Bioinformatics. 2018;34 17:i884-i90. doi:10.1093/bioinformatics/bty560.
96. Langmead B, Salzberg SL. Fast gapped-read alignment with Bowtie 2. Nature methods. 2012;9 4:357-9. doi:10.1038/nmeth.1923.
97. Wang Y, Tang H, Debarry JD, Tan X, Li J, Wang X, et al. MCScanX: a toolkit for detection and evolutionary analysis of gene synteny and collinearity. Nucleic acids research. 2012;40 7:e49. doi:10.1093/nar/gkr1293.
98. Linger R J, Belikoff E J, Scott M J. Dosage Compensation of X-Linked Muller Element F Genes but Not X-Linked Transgenes in the Australian Sheep Blowfly. PLOS ONE. 2015; 10.10(2015):e0141544.
99. Landeen EL, Presgraves DC. Evolution: From Autosomes to Sex Chromosomes - and Back. Current biology. 2017;CB. 23. R848-50. 10.1016/j.cub.2013.08.021
100. Jager AC, Alvarez ML, Davis CP, Guzman E, Han Y, Way L, et al. Developmental validation of the MiSeq FGx Forensic Genomics System for Targeted Next Generation Sequencing in Forensic DNA Casework and Database Laboratories. Forensic science international Genetics. 2017;28:52-70. doi:10.1016/j.fsigen.2017.01.011.

Table S9. Stati

Branch	Branch Length	N	Families
C.lectularius	355.288	5587	204
B.germanica	341.947	5559	370
A.aegypti	238.846	5789	488
D.melanogaster	141.291	5845	181
G.austeni	82.4772	5763	487
M.domestica	31.9392	5772	262
S.calcitrans	31.9392	5776	132
S.calcitrans |*M.domestica*	23.7435	5858	74
P.regina (M)	5.2424	4097	189
P.regina (F)	5.2424	4036	128
P.regina (F)|*P.regina* (M)	23.3645	4374	114
L.cuprina	14.4816	5836	168
A.grahami	14.4816	5761	98
A.grahami |*L.cuprina*	14.1253	5870	39
A.grahami ,*L.cuprina* |*P.regina* (F),*P.regina* (M)	27.0758	5870	17
A.grahami ,*L.cuprina* ,*P.regina* (F),*P.regina* |*S.calcitrans* ,*M*	26.7946	5870	38
A.grahami ,*L.cuprina* ,*P.regina(F)* ,*P.regina* ,*S.calcitrans* ,*l*	58.8136	5870	80
A.grahami ,*L.cuprina* ,*P.regina* (F),*P.regina* ,*S.calcitrans* ,*l*	97.5555	5870	86
A.grahami,*L.cuprina*,*P.regina* (F),*P.regina*,*S.calcitrans*,*M.*	70.0372	5870	54
N.vespilloides	283.738	5771	222
O.taurus	283.738	5779	461
O.taurus |*N.vespilloides*	25.1456	5870	18
O.taurus,*N.vespilloides* |*A.grahami*,*L.cuprina*,*P.regina(F)*,.	33.0629	5870	9
O.taurus,*N.vespilloides*,*A.grahami*,*L.cuprina*,*P.regina(F)*,*l*	13.3412	5870	0

*** Branch, brances on evolutionary tree; N, number of gene family; Gene Gain/Family, average ge**

istics of gene family expansion and contraction

Expansions		Contractions			Extinctions	
Genes	Gene Gain/Family	Families	Genes	Gene Loss/Family	Families	Genes
274	1.34314	415	469	1.13012	283	311
505	1.36486	436	482	1.1055	311	329
867	1.77664	132	139	1.05303	81	82
239	1.32044	71	78	1.09859	25	28
581	1.19302	188	216	1.14894	107	108
327	1.24809	131	131	1	86	86
173	1.31061	139	140	1.00719	82	82
113	1.52703	48	50	1.04167	12	12
198	1.04762	314	324	1.03185	277	279
131	1.02344	374	376	1.00535	338	338
134	1.17544	1654	1717	1.03809	1496	1514
210	1.25	72	75	1.04167	34	34
150	1.53061	174	183	1.05172	109	116
47	1.20513	29	29	1	0	0
17	1	30	31	1.03333	0	0
44	1.15789	19	19	1	0	0
82	1.025	18	41	2.27778	0	0
94	1.09302	69	88	1.27536	0	0
76	1.40741	48	60	1.25	0	0
352	1.58559	200	236	1.18	99	110
719	1.55965	177	188	1.06215	91	95
20	1.11111	3	3	1	0	0
10	1.11111	22	23	1.04545	0	0
0	NA	0	0	NA	0	0

ene expanding in each family; Avg./Exp=(total genes gained along branch - total genes lost along branch)/to

[illegible]s		
Gene Extinct/Family	No Change	Avg. Exp.
1.09894	5251	-0.0332198
1.05788	5064	0.00391823
1.01235	5250	0.12402
1.12	5618	0.0274276
1.00935	5195	0.0621806
1	5465	0.0333901
1	5587	0.00562181
1	5748	0.0107325
1.00722	3871	-0.0214651
1	3872	-0.0417376
1.01203	4102	-0.269676
1	5630	0.0229983
1.06422	5598	-0.00562181
NA	5802	0.00306644
NA	5823	-0.00238501
NA	5813	0.00425894
NA	5772	0.00698467
NA	5715	0.00102215
NA	5768	0.00272572
1.11111	5448	0.0197615
1.04396	5232	0.09046
NA	5849	0.00289608
NA	5839	-0.00221465
NA	5870	0

[illegible]otal genes at ancestral node of branch.

 Click here to access/download;Table;Table S11.xlsx

'able S11. Genome-wide characteristic on pseudoc

	Muller element of *D. melanogaster*	Length	Scaf Num	all genes	Density (/mb)	positively	Density (/mb)
Chr01	Muller B	112,158,196	226	2119	18.89	23	0.205068
Chr02	Muller D	109,743,127	180	2411	21.97	32	0.29159
Chr03	Muller A	104,650,035	162	2424	23.16	29	0.277114
Chr04	Muller C	104,176,436	114	2499	23.99	21	0.201581
Chr05	_	89,513,788	61	1804	20.15	16	0.178743
Chr06	Muller E	57,970,779	50	1305	22.51	10	0.172501

hromosomes of the *A. graham*

expansion	Density (/mb)	contraction	Density (/mb)	LTR	Density (/mb)
50	0.445799	21	0.187236	14529	129.5402
54	0.492058	18	0.164019	11038	100.5803
51	0.487339	20	0.191113	8843	84.50069
65	0.623942	40	0.383964	9936	95.37666
43	0.480373	26	0.290458	9998	111.6923
43	0.741753	10	0.172501	3758	64.82576

Figure 1

Click here to access/download;Figure;Figure 1.pdf

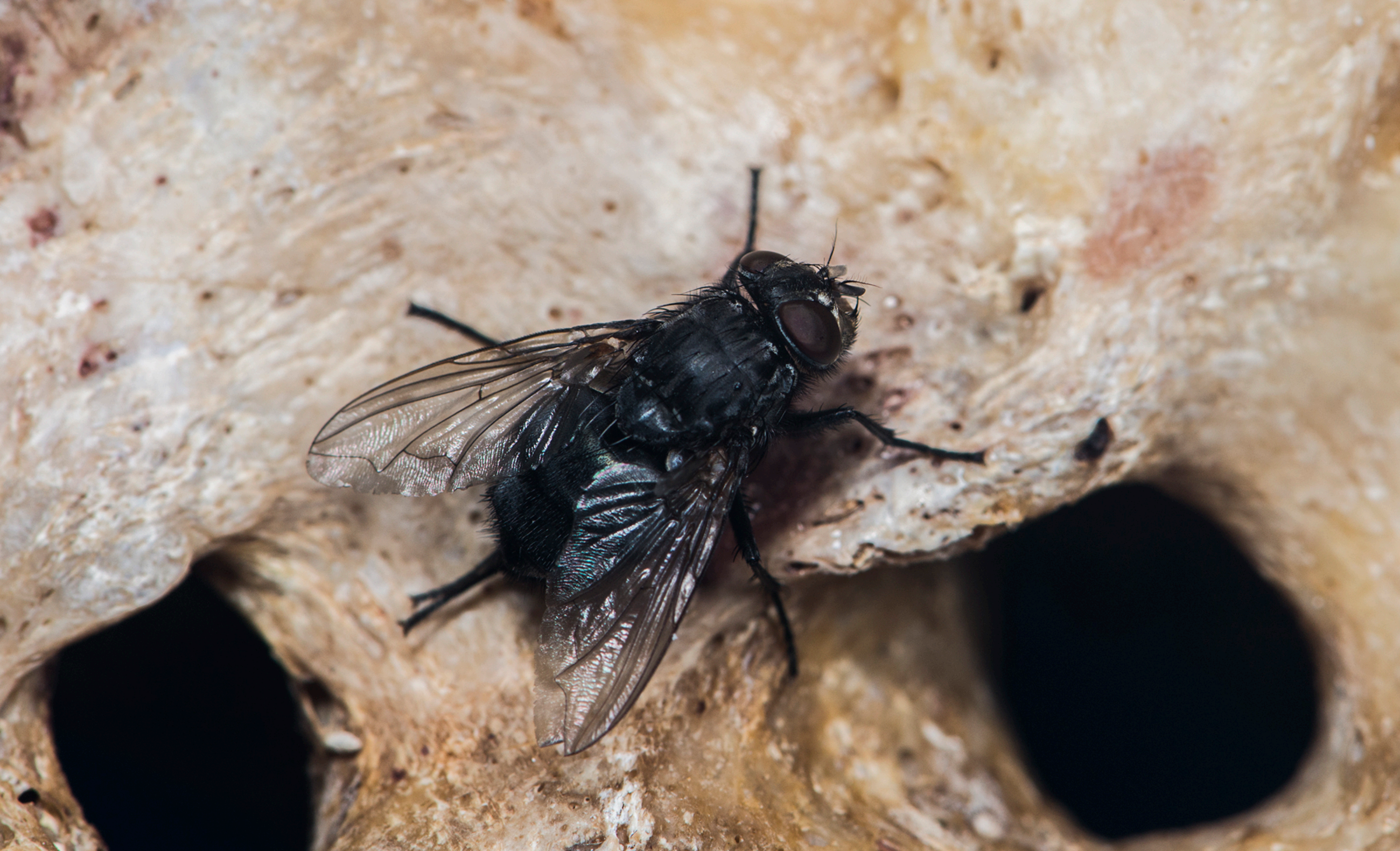

Figure 2

Click here to access/download;Figure;Figure 2.pdf

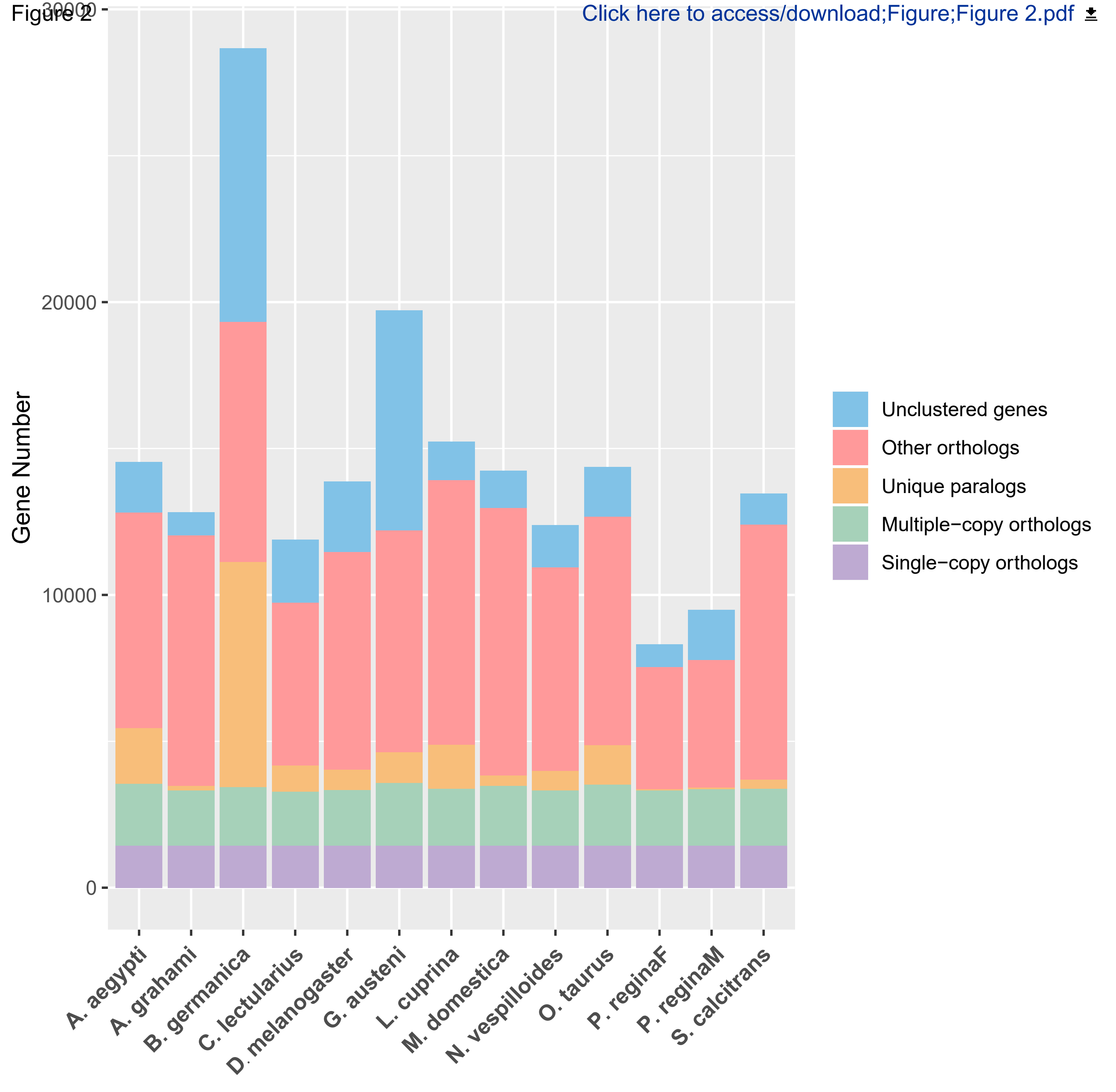

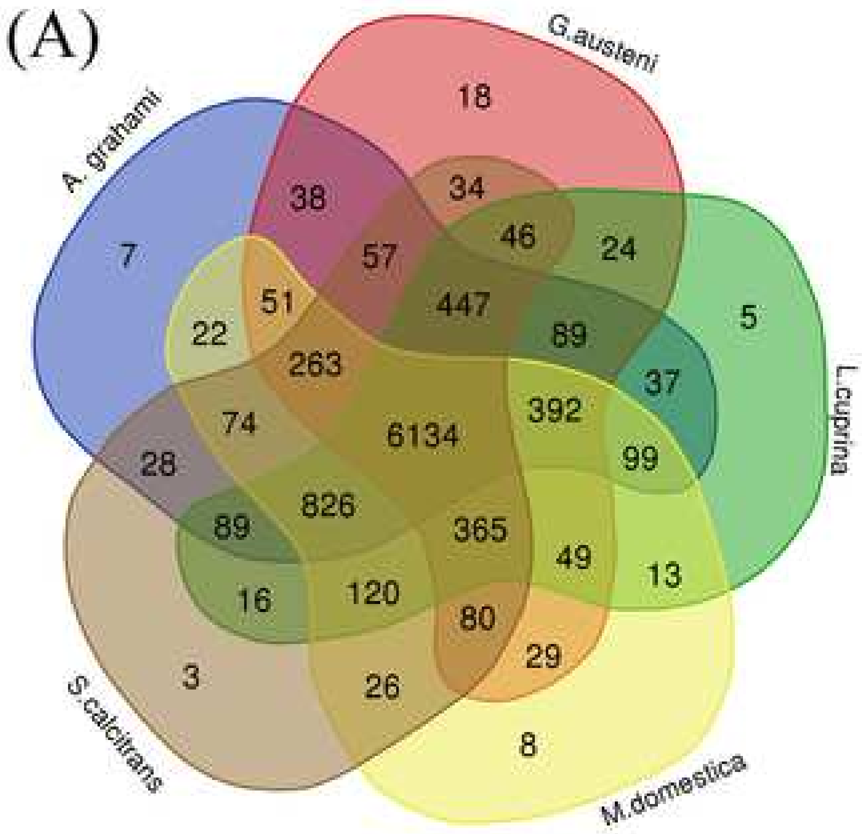
(A)
G.austeni
A. grahami
L.cuprina
M.domestica
S.calcitrans
18
38
34
46
24
7
57
447
5
51
22
89
263
37
392
74
6134
99
28
826
365
89
49
13
16
120
80
29
3
26
8
(B)
N.vespilloides
A. grahami
O.taurus
B.germanica
C.lectularius
8
133
15
149
45
343
154
1154
6
136
149
399
343
140
557
171
4290
131
127
256
378
170
81
8
23
41
40
13
5
15
9

Figure 4

Click here to access/download;Figure;Figure 4.png

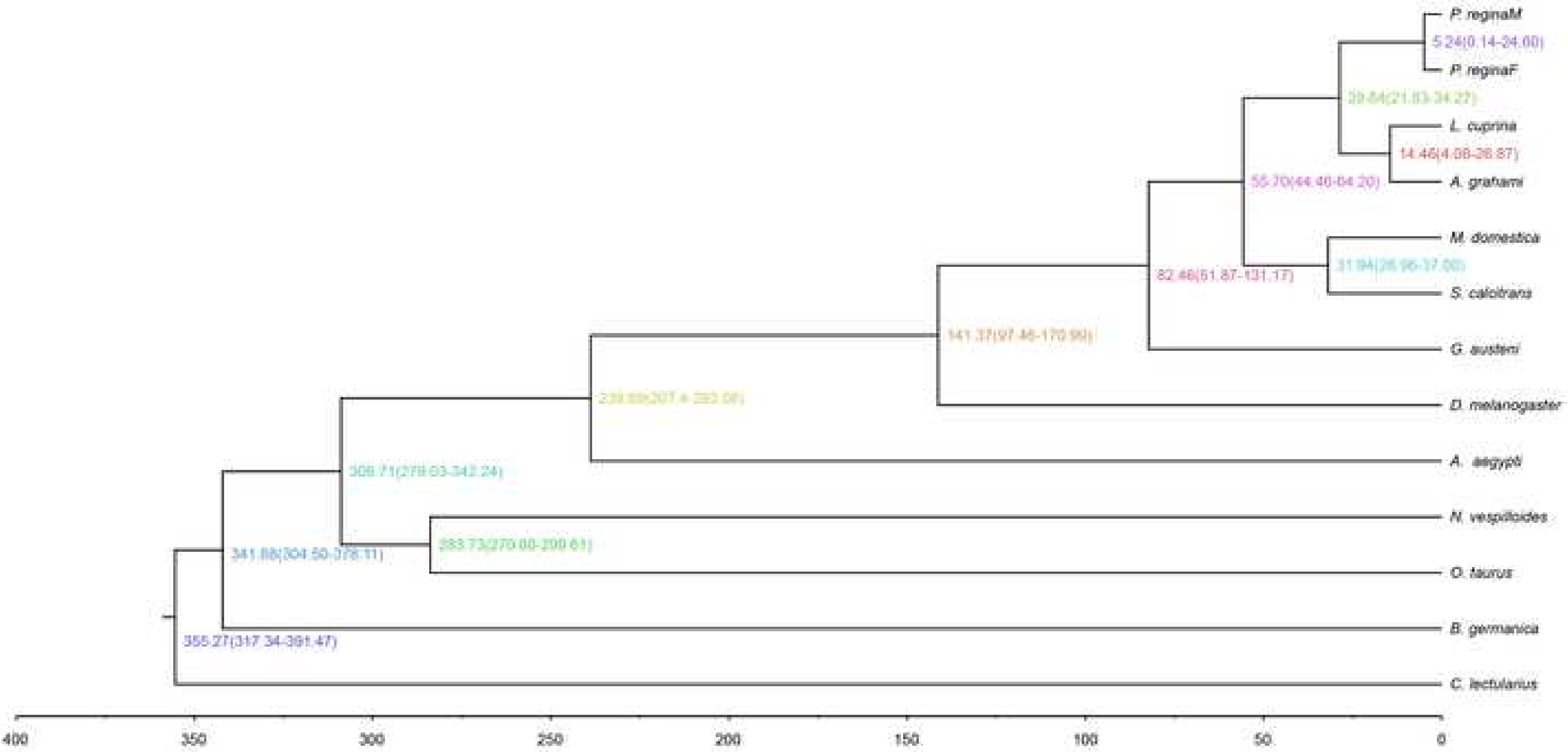

Figure 5

Click here to access/download;Figure;Figure 5.pdf

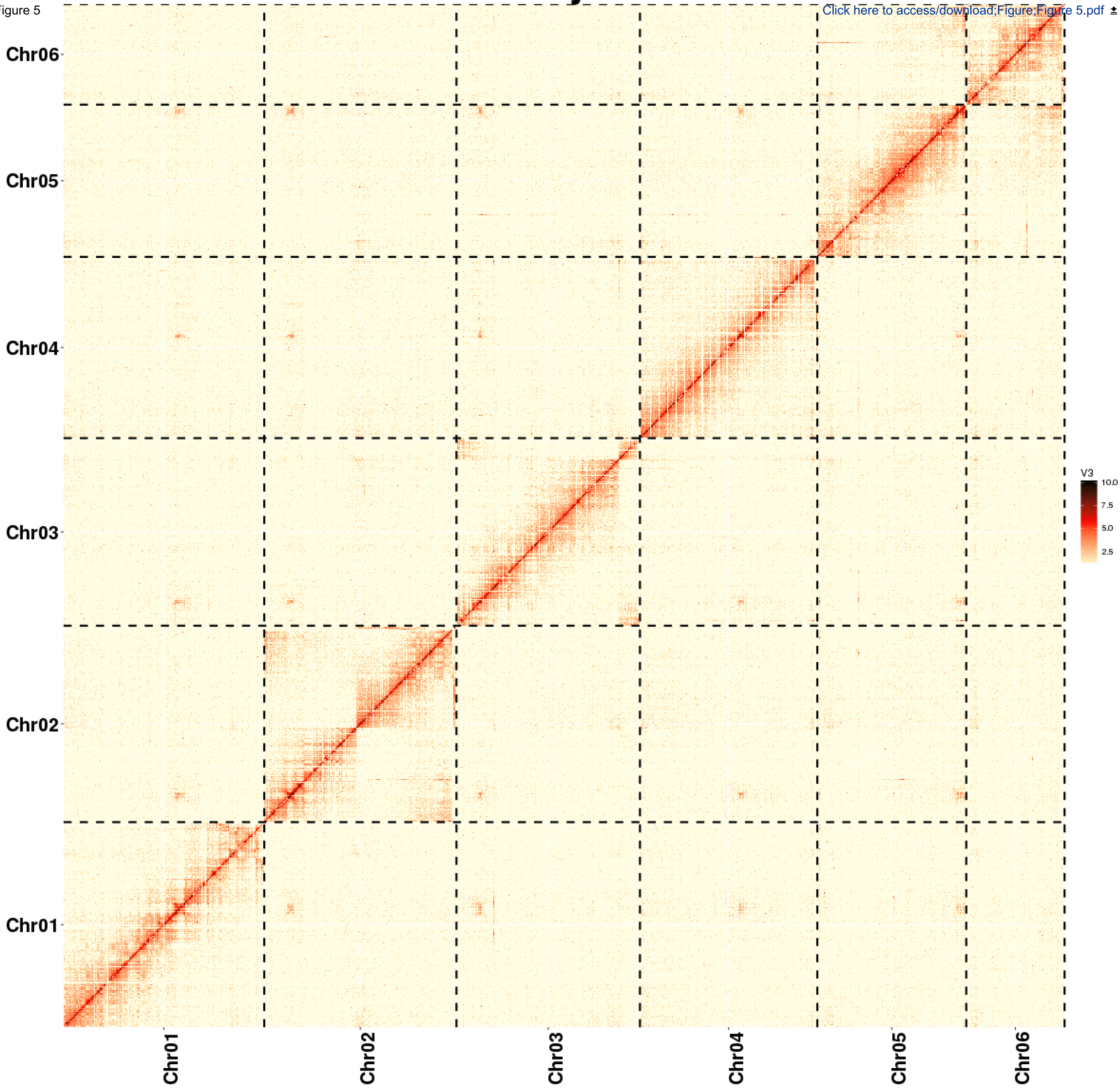

Figure 6

Click here to access/download;Figure;Figure 6.jpg

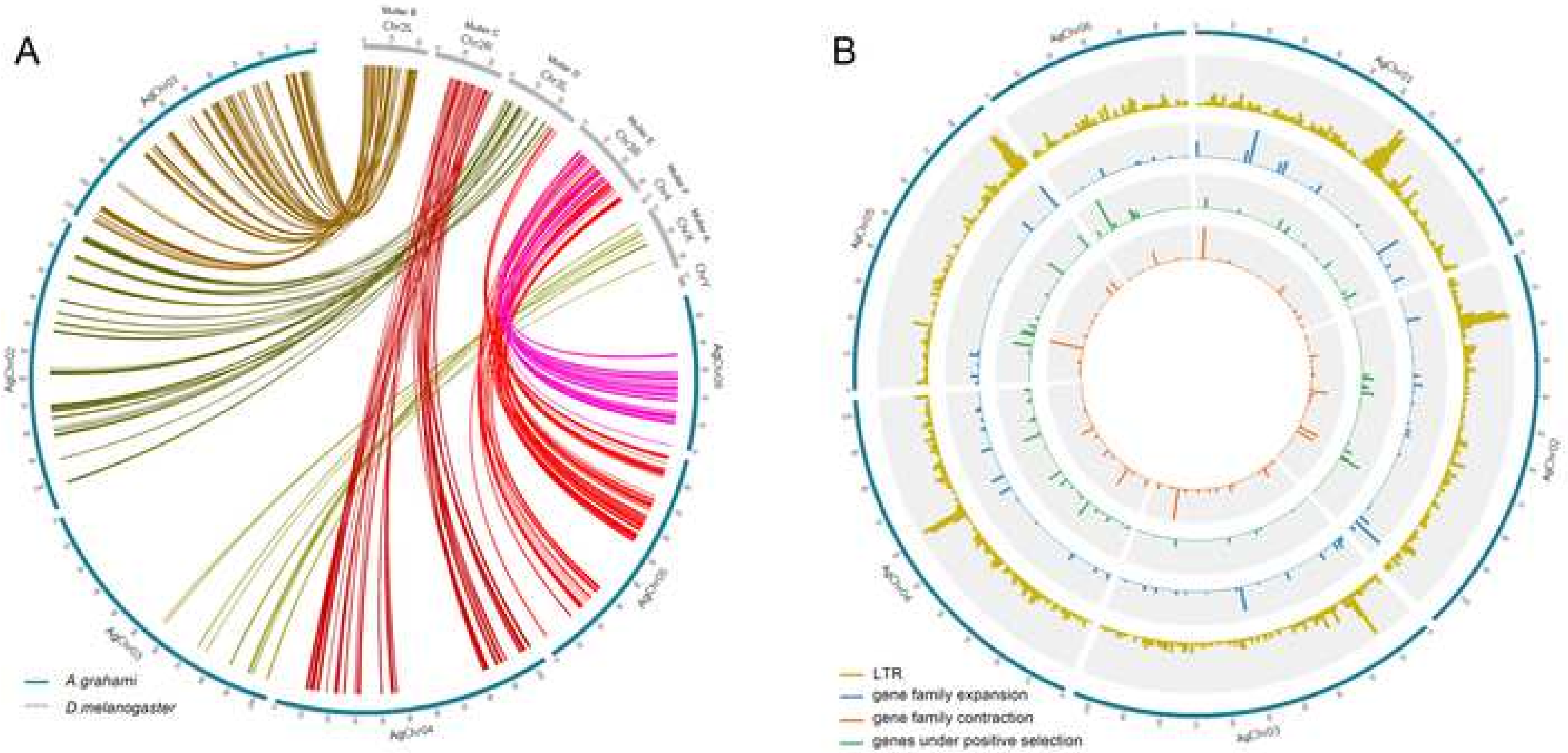

Figure S1

Click here to access/download;Figure;Figure

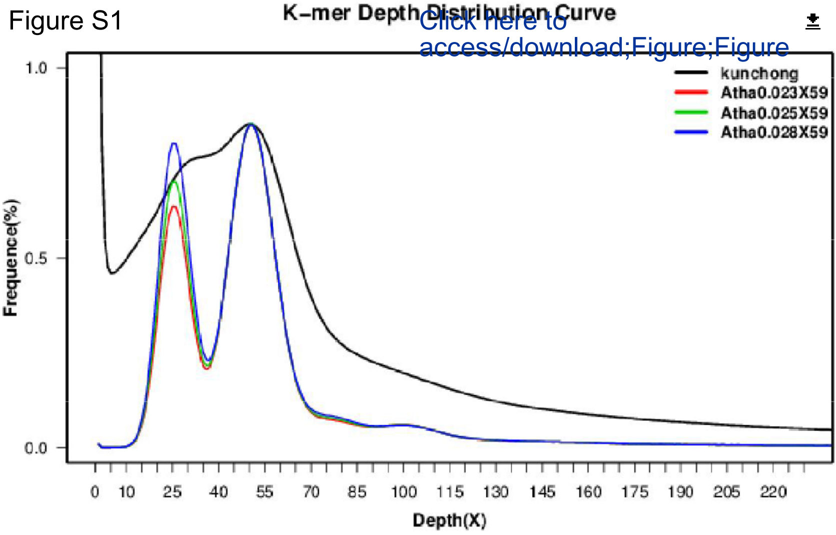

Figure S2

Click here to access/download;Figure;Figure S2.tif

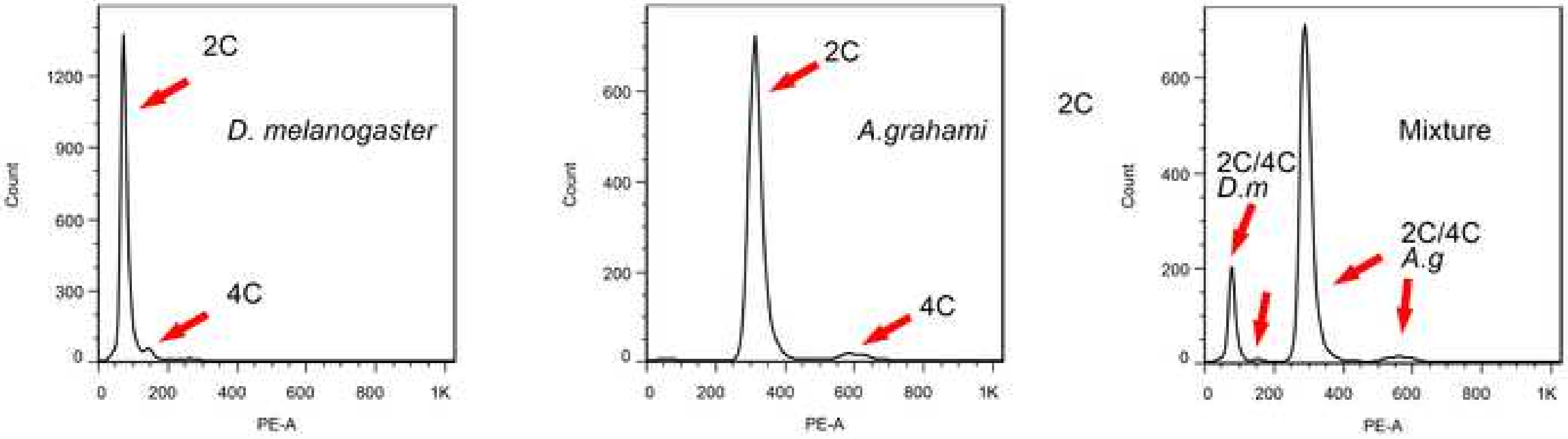

20190702_Tube_001.fcs
Cell Cycle
Dean-Jett-Fox
RMS = 26.37
Freq. G1 = 14.03
Freq. S = 49.62
Freq. G2 = 0
G1 Mean = 71.3
G2 Mean = 145
G1 cv = 2.99
G2 cv = 2.95
Freq. sub-G1 = 27.79
Freq. super-G2 = 6.6

9891

20190702_Tube_002.fcs
Cell Cycle
Watson
RMS = 15.83
Freq. G1 = 32.41
Freq. S = 33.52
Freq. G2 = 1.18
G1 Mean = 312
G2 Mean = 602
G1 cv = 2.87
G2 cv = 3
Freq. sub-G1 = 29.16
Freq. super-G2 = 2.57

9808

20190702_Tube_004.fcs
Cell Cycle
Dean-Jett-Fox
RMS = 18.41
Freq. G1 = 2.38
Freq. S = 21.57
Freq. G2 = 32.3
G1 Mean = 77
G2 Mean = 286
G1 cv = 2.93
G2 cv = 2.88
Freq. sub-G1 = 5.19
Freq. super-G2 = 36.31

9873

Figure S3

Click here to access/download;Figure;Figure S3.tif

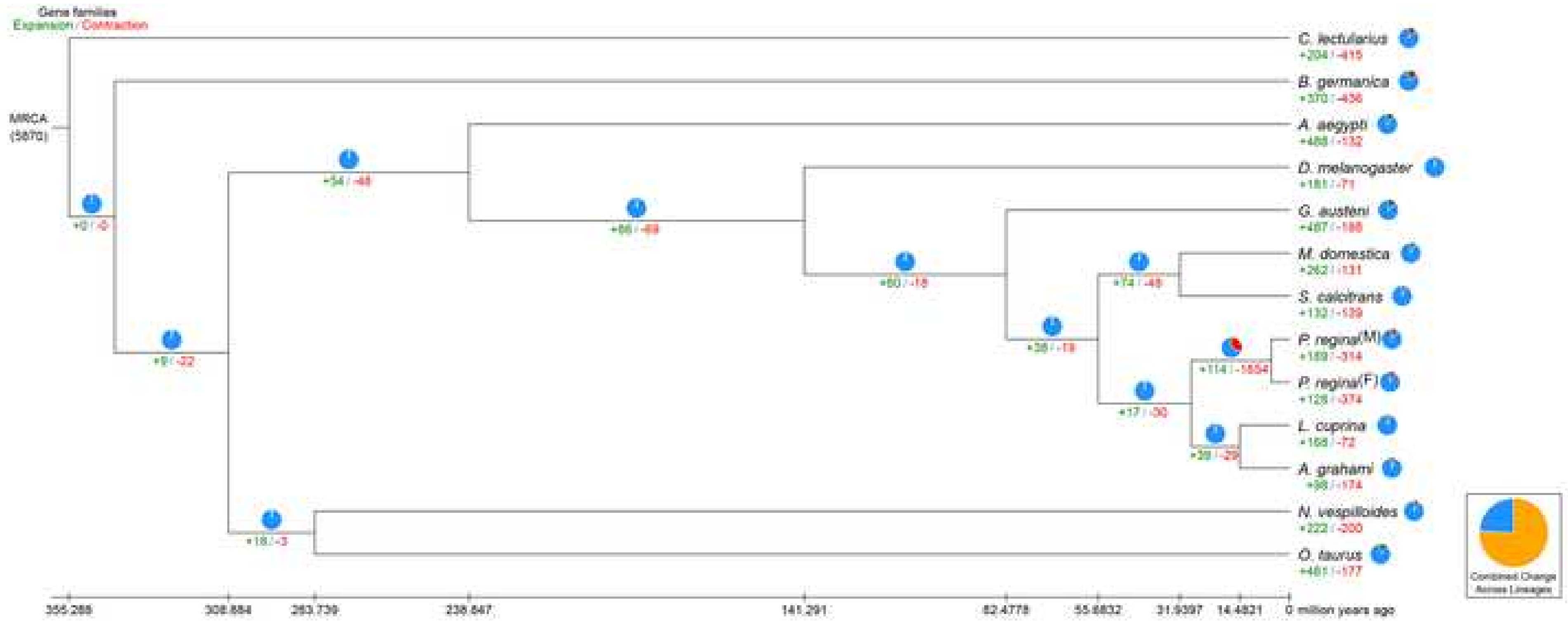

Figure S4

Click here to access/download;Figure;Figure S4.pdf

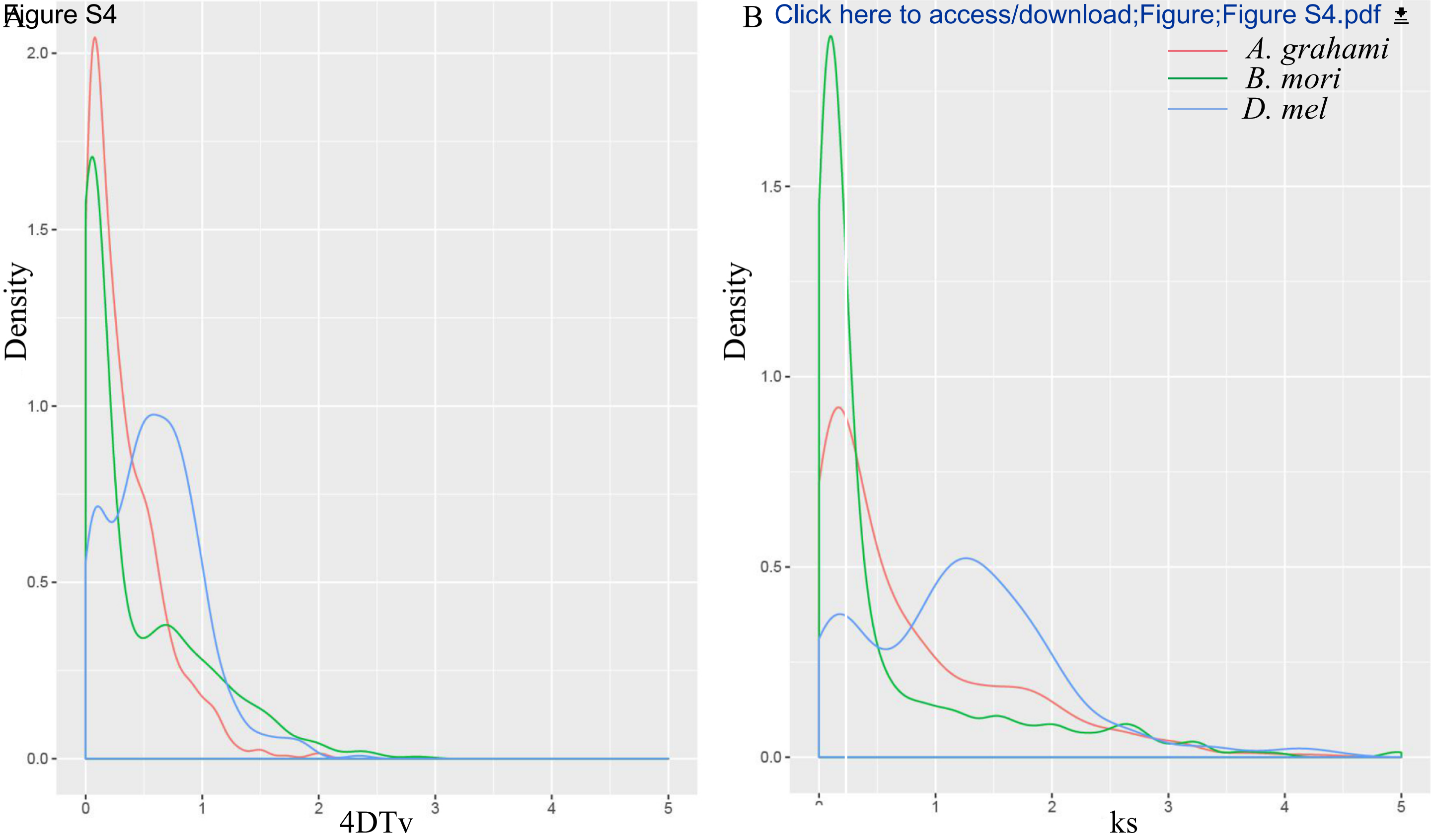

Supplementary tables

Click here to access/download
Supplementary Material
supplementary table 20191020.docx

Cover letter Click here to access/download;Personal Cover;cover letter 20191020.docx

Dear Editor Zauner and reviewers,

We would like to express our great appreciation to you and reviewers for those constructive comments and suggestions on our manuscript, which provide really valuable and helpful for revising and improving our paper.

Here we are resubmitting the manuscript “Chromosomal-level genome assembly of *Aldrichina grahami*, a forensically important blow fly” by Meng et al. (GIGA-D-19-00066R1) in the 2ed round revision. Because it is the last chance for the potential approval of our manuscript, and for the importance of genome resource of this forensically related fly species, we have studied comments carefully and tried our best to improve the manuscript. We hope that the correction will meet with approval.

In this revised version, we response the questions you highlighted in your letter firstly. And after carefully study and revision, we tried to answer all the reviewers’ questions and corrected the related parts as reviewers recommended. The new submitted version of our manuscript was also carefully revised by native speaker. And we can provide the Certificate of English Proofreading. Changes were marked all in RED.

Thanks again to the reviewers and editors of *GigaScience* for patiently revised our previous submission and giving us the opportunity to make improvement. We are looking forward to hearing from you.

Sincerely yours!

Jifeng Cai, on behalf of all authors
Forensic Science department, School of Basic Medicine, Central South University.
Changsha, Hunan Province, China
Email: cjf_jifeng@163.com
