# Supplementary material for: Chromosome-level genome assembly of Aldrichina grahami, a forensically important blowfly
Source: Gigascience. 2020 Mar 19;9(3):giaa020. doi: 10.1093/gigascience/giaa020 (PMC7081965; doi:10.1093/gigascience/giaa020)
Supplement: giaa020_GIGA-D-19-00066_Revision_3 [file giaa020_giga-d-19-00066_revision_3.pdf]

GigaScience

Chromosomal-level genome assembly of Aldrichina grahami, a forensically important blow fly

--Manuscript Draft--

**Manuscript Number:**	GIGA-D-19-00066R3
**Full Title:**	Chromosomal-level genome assembly of Aldrichina grahami, a forensically important blow fly
**Article Type:**	Data Note
**Funding Information:**	National Natural Science Foundation of China (81571855) — Pro. Jifeng Cai<br>Science Foundation of Hunan Province (2017SK2015) — Pro. Jifeng Cai
**Abstract:**	Background : Blow flies (Diptera: Calliphoridae) are the most commonly found entomological evidence in the forensic investigation. Distinguished from other blow flies, Aldrichina grahami with some unique biological characteristics is species of forensic importance. Its development rate, pattern and life cycle can provide valuable information for the estimation of the minimum postmortem interval (minPMI).<br>Findings : Herein we provide a chromosomal-level genome assembly of A. grahami that was generated by Pacific BioSciences (PacBio) sequencing platform and chromosome conformation capture (Hi-C) technology. A total of 50.15 Gb clean reads of the A. grahami genome were generated. Programs FALCON and Wtdbg were utilized to construct the genome of A. grahami , resulting in an assembly of 600 Mb and 1604 contigs with an N50 size of 1.93 Mb. We predicted 12823 protein-coding genes, 99.8% of which was functionally annotated based on the de novo genome (SRA: PRJNA513084) and transcriptome (SRA: SRX5207346) of A. grahami . According to the co-analysis with 10 other insect species, the clustering and phylogenetic reconstruction of the gene families were performed. Using Hi-C sequencing, a chromosomal-level assembly of 6 chromosomes was generated with scaffold N50 of 104.7 Mb. Of these scaffolds, nearly 96.4% were anchored to the total A. grahami genome contig bases.<br>Conclusions : The present study provides a robust genome reference of the A. grahami which supplements vital genetic information for the nonhuman forensic genomics, and facilitates the future research of A. grahami and other necrophagous blow fly species used in forensic medicine.
**Corresponding Author:**	Jifeng Cai<br><br>CHINA
**Corresponding Author Secondary Information:**	
**Corresponding Author's Institution:**	
**Corresponding Author's Secondary Institution:**	
**First Author:**	Fanming Meng
**First Author Secondary Information:**	
**Order of Authors:**	Fanming Meng<br>Zhuoying Liu<br>Jifeng Cai<br>Han Han<br>Dmitrijs Finkelbergs<br>Yangshuai Jiang

Powered by Editorial Manager® and ProduXion Manager® from Aries Systems Corporation

	Mingfei Zhu
	Chao Chen
	Yadong Guo
	Yang Wang
	Zongyi Sun
**Order of Authors Secondary Information:**	
**Response to Reviewers:**	Dear Editor,<br>We are happy to hear the letter from you and we would like to give our great appreciation to you and all the reviewers again, as well as the Data editor of GigaScience. In the version of revise, we followed the suggestions as you listed, then made briefly response to reviewer 2. Please check it as below.<br><br>Your suggestions<br>- Please have a look at the reviewers' final comments (see below) and consider briefly addressing them in the discussion section of your paper.<br>A: we made some briefly description and revision as the reviewer 2 suggested, and accepted the reviewer's advises for our future research<br><br>- Please carefully revise your manuscript for English language use and grammar, preferably with the help of a native speaker.<br>A: We invited native speaker who have experience of research article writing to polish the final version of our manuscript.<br><br>- Please mention the NCBI taxon ID for the species in the methods section (I believe it is NCBI:txid252811 , but please double check).<br>A: The NCBI taxon ID of Aldrichina grahami has been rechecked and confirmed as NCBI:txid252811. And it can be located by the following link (https://www.ncbi.nlm.nih.gov/Taxonomy/Browser/wwwtax.cgi?id=252811)<br><br>- At this point, you can remove any highlighting in red that was made for the purpose of review.<br>A: Changed as you suggested.<br><br>- Our data curators will contact you shortly to prepare the supporting dataset which will be hosted via our database GigaDB. Prior to publication, please include a citation to your upcoming GigaDB dataset (including the DOI link) to your reference list, and cite this in the data availability section and elsewhere in the manuscript, where appropriate.<br>A: We have completed the information of dataset on GigaDB following the guide of data curator, and cited our data linke of DigaDB as reference in the final version of our manuscript.<br><br><br>Reviewer reports:<br>Reviewer #2: The authors have done an admirable job of addressing reviews. They have justified their cytometric estimates of genome size in much more detail and have addressed my questions about their claims regarding sex chromosomes.<br><br>Small comments for consideration:<br>1. I believe the genome size of D. melanogaster of 175 Mb is linked to the originally sequenced strain. There may be subtle differences between it and W1118. For example, see Ellis et al. 2014 in PLoS Genetics for variation in the DGRP strains of Drosophila melanogaster. Surely the authors are close enough to the true value for the purposes of this paper, but it is worth remembering. For future reference, given the size of this genome, D. virilis may have been a better choice as it is closer to the size of what is of interest to the authors. Not necessary for this paper, but something to consider for future projects if authors continue to do this sort of work.<br><br>A: Thanks a lot for your further advice on this detail. After revised this manuscript, we do plan to explore the genome size of several forensic related fly species both by genome survey and cytometric. For the reference genome size, we would like to use blood of domestic chicken, D.melanogaster (w118), and D. virilis separately, and make

Powered by Editorial Manager® and ProduXion Manager® from Aries Systems Corporation

	a comparison between them. Although it is not our major research area, we would like to gain some knowledge of genome size of this insect group. And finally thanks again for all this valuable information you provided to us. That's really helpful.<br><br>2. The fonts in some of the figures are low resolution and hard to read.<br>A: All the figures used in the last version of manuscript have been replaced with large enough resolution for readable.
Additional Information:	
Question	Response
Are you submitting this manuscript to a special series or article collection?	No
**Experimental design and statistics**<br><br>Full details of the experimental design and statistical methods used should be given in the Methods section, as detailed in our Minimum Standards Reporting Checklist. Information essential to interpreting the data presented should be made available in the figure legends.<br><br>Have you included all the information requested in your manuscript?	Yes
**Resources**<br><br>A description of all resources used, including antibodies, cell lines, animals and software tools, with enough information to allow them to be uniquely identified, should be included in the Methods section. Authors are strongly encouraged to cite Research Resource Identifiers (RRIDs) for antibodies, model organisms and tools, where possible.<br><br>Have you included the information requested as detailed in our Minimum Standards Reporting Checklist?	Yes
**Availability of data and materials**<br><br>All datasets and code on which the conclusions of the paper rely must be either included in your submission or deposited in publicly available repositories	Yes

Powered by Editorial Manager® and ProduXion Manager® from Aries Systems Corporation

(where available and ethically appropriate), referencing such data using a unique identifier in the references and in the “Availability of Data and Materials” section of your manuscript.<br><br>Have you have met the above requirement as detailed in our Minimum Standards Reporting Checklist?	

Manuscript
Click here to access/download;Manuscript;blowfly_forpublication.docx

Chromosome-level genome assembly of *Aldrichina grahami*, a forensically important blow fly

Fanming Meng[1], Zhuoying Liu[1], Han Han[1], Dmitrijs Finkelbergs[1], Yangshuai Jiang[1], Mingfei Zhu[2], Yang Wang[2], Zongyi Sun[2], Chao Chen[3], Yadong Guo[1], Jifeng Cai[1]*

[1] School of Basic Medicine, Central South University, Changsha, Hunan Pro, China

[2] Nextomics Biosciences, Wuhan, Hubei Pro, China

[3] Institute of Apicultural Research, Chinese Academy of Agricultural Sciences

* Corresponding author

Emails:

F. M: mengfanming1984@163.com;

Z. L: 214872404@qq.com;

H. H: 583538543@qq.com;

D. F: dfinkelbergs@yahoo.com

Y. J: 1464804060@qq.com

M. Z: zhumingfei@grandomics.com

Y. W: wangyang-1@grandomics.com

Z. S: sunzongyi@grandomics.com

C. C: chenchaoiar@163.com

Y. G: gdy82@126.com

J. C: cjf_jifeng@163.com

Abstract

Background: Blow flies (Diptera: Calliphoridae) are the most commonly found entomological evidence in forensic investigations. Distinguished from other blow flies, *Aldrichina grahami* has some unique biological characteristics and is a species of forensic importance. Its development rate, pattern and life cycle can provide valuable information for the estimation of the minimum postmortem interval (minPMI).

Findings: Herein we provide a chromosomal-level genome assembly of *A. grahami* that was generated by Pacific BioSciences (PacBio) sequencing platform and chromosome conformation capture (Hi-C) technology. A total of 50.15 Gb clean reads of the *A. grahami* genome were generated. FALCON and Wtdbg were utilized to construct the genome of *A. grahami*, resulting in an assembly of 600 Mb and 1604 contigs with an N50 size of 1.93 Mb. We predicted 12823 protein-coding genes, 99.8% of which was functionally annotated based on the *de novo* genome (SRA: PRJNA513084) and transcriptome (SRA: SRX5207346) of *A. grahami*. According to the co-analysis with ten other insect species, clustering and phylogenetic reconstruction of gene families were performed. Using Hi-C sequencing, a chromosomal-level assembly of 6 chromosomes was generated with scaffold N50 of 104.7 Mb. Of these scaffolds, nearly 96.4% were anchored to the total *A. grahami* genome contig bases.

Conclusions: The present study provides a robust genome reference for *A. grahami* which supplements vital genetic information for nonhuman forensic genomics, and facilitates the future research of *A. grahami* and other necrophagous blow fly species used in forensic medicine.

Keywords: *Aldrichina grahami*; Blow fly; Necrophagous; Forensic entomology; Minimum postmortem interval; Genome assembly

Data Description

Background

Forensic entomology focuses on the application of insects and other arthropods in the medicolegal investigation. Studying the development rate of insect colonizers on the corpse and insect succession patterns during corpse decomposition can assist in the estimation of the minimum postmortem interval (minPMI), which represents the main task of the forensic investigation [1-3]. In addition, insect evidence is helpful in the detection and recognition of wounds, the estimation of the time length of neglect or abuse, and the investigation of the cause of death [4-7]. The most important group of insects for forensic investigation is the Diptera, especially necrophagous fly species of Calliphoridae [8, 9]. Flies of this fauna, usually called “blow fly”, consist of many species with a parasitic or necrophagous lifestyle [10, 11]. The reliable life cycle of these necrophagous flies can provide vital information for forensic entomologists or investigators to infer a relatively accurate minPMI under certain assumptions [8, 12-14].

Figure 1. Female adult of *Aldrichina grahami* on the corpse.

Aldrichina grahami (Aldrich, 1930; NCBI:txid252811, homotypic synonym: *Calliphora grahami*) (Fig. 1) is a common blow fly species indigenous to East Asia [15, 16] which has expanded to the American continent in the past several decades [17-19]. It usually breeds on carcasses or feces, posing a potential threat of contaminating human food [15]. *A. grahami* is a forensically important insect because of its necrophagous behavior, seasonal distribution, and particularly unique characteristics of low-temperature tolerance, all of which distinguish it from other necrophagous flies [20-22]. *A. grahami* is frequently the first species to colonize the

corpse in early spring and late autumn, when ambient temperature is relatively low. In some extreme cases this species can be the only colonizer [23, 24]. The information provided by the seasonal distribution pattern of *A. grahami* could be applied as a potential ‘season stamp’ of the time of death in the PMI estimation, especially in the period when other insects are inactive [22, 25]. Moreover, the successful extraction and identification of human DNA material from gut contents of *A. grahami* and other blow fly larvae can provide important information about a missing corpse or help to interpret the evidence used for forensic investigation [26, 27]. The age-dependent altering pattern of cuticular hydrocarbons in larvae cuticle has great application potential in the forensic investigation [28, 29]. Besides the forensic importance, cases of myiasis caused by *A. grahami* have been reported routinely in China, especially when people travel back from undeveloped regions [30-33]. This blow fly species is also a potential transmitter of pathogens, such as the H5N1 influenza virus, which could cause serious public health problems in animals and humans [34].

Researches of insect biochemistry and physiology prompt our deeper understanding of *A. grahami* [35-38]. Nuclear materials are primarily applied to distinguish *A. grahami* from sibling Diptera species [39-42]. Several researchers have described the development patterns of *A. grahami* under different environmental conditions [20, 21]. Nonetheless, the genome of *A. grahami* is still unavailable, which impedes its further applications in forensic research. Previous studies have indicated that variation at the genetic level has a detectable and potentially important influence on the length of development and the life cycle of the fly species among different geographic populations [43-45]. It was also recommended that the investigation of such forensic investigations should be based on a high-quality genome reference of the investigated fly species [46-48]. Here we provide a chromosome-scale scaffolding

of the genome assembly of this forensically important blow fly, using the Pacific BioSciences (PacBio) sequencing platform and chromosome conformation capture (Hi-C) method, which promotes the future research of forensic and medical science.

Genome Sequencing and Assembly

Sample preparation

The first generation of *A. grahami* was collected using beef liver as baits, in Changsha (Hunan Province, China) in March 2017. Species identification was performed through morphological and molecular methods. The fly species were distinguished following the morphological description found in the literature of Fan's study (1992) [15]. Then cytochrome oxidase gene I (*COI*) as a molecular marker was amplified from the DNA of *A. grahami* using the previously mentioned method (Primer F: 5-TACAATTTATCGCCTAAACTTCAGCC-3; R: 5-CATTTCAAGCTGTGTAAGCATC-3) [39]. After sequencing the amplification product (ABI 3730xl, USA), the result was searched by BLAST and deposited into the NCBI website (Accession number: MN537823). It was recognized as belonging to *A. grahami*. The blow flies were bred for more than 20 generations in the laboratory of the School of Basic Medicine, Central South University. Newly emerged and unmated female adults were used for DNA extraction.

After sample collection, the used tissues were immediately immersed into liquid nitrogen and stored at -80℃. DNA was extracted using the Cetyltrimethyl Ammonium Bromide (CTAB) method followed by the introduction of Size-Selected 20 Kb SMRTbell^{TM} Libraries for genomic DNA preparation. The quality of the extracted genomic DNA was checked using gel electrophoresis with 0.7% agarose. Then

Nanodrop spectrophotometer (Thermo Fisher Scientific) was used to calculate the DNA purity. The concentration of extracted material was examined by Qubit fluorimeter (Invitrogen, Carlsbad, CA, USA).

New males and females were sampled for transcriptome sequencing. After the extraction quality control and library construction, the Illumina Hiseq X10 platform was used to perform the RNA-seq. Five new female adults with their wings dissected and gut removed, were used for library construction.

Every voucher specimen was assigned with a unique code. All specimens were deposited in the forensic insect herbarium of the Department of Forensic Science, Central South University, Changsha.

Library construction and sequencing

Two libraries were constructed before sequencing. First a library of short-insert length (400 bp) was constructed by Illumina TruSeq Nano DNA Library Prep Kits. The short-insert library sequencing was performed on the Illumina HiSeq X10 instrument at Genetron Health (Beijing, China) using the whole-genome shotgun sequencing (WGS) strategy. A total of 46.05 Gb of raw data were collected and subsequently filtered. Finally, 42.4 Gb of clean data for short reads were generated (Table S1).

The long reads library of 20 Kb was prepared using a SMRTbell DNA Template Prep Kit 1.0 (PacBio p/n 10Tal-259-100). DNA fragments of approximately 20Kb were generated by shearing genomic DNA material using a Covaris G-TUBE^{TM} (Kbiosciences p/n 520079). The sheared genomic DNA was damage-repaired and end-repaired using polishing enzymes. The blunt-end ligation resulted from the exonuclease treatment was used to generate a SMRTbell template. After that,

fragments with proper size (⩾15Kb) were subsequently selected by the Blue Pippin device (Sage Science, Inc., Beverly, MA, USA). The DNA 12000 Kit for Agilent Bioanalyzer 2100 (Agilent p/n 5067-1508) was used for figuring out the distribution of fragments with different sizes.

The prepared DNA template libraries were bound to the Sequel Polymerase 2.0 using Sequel Binding Kit 2.0 (PacBio p/n 100-862-200) in preparation for sequencing on the Sequel System. Finally, a DNA polymerase/template complex formed according to the manufacturer's instructions. The enrichment of the larger fragments was improved by the MagBead (PacBio p/n 100-125-900) method. The long-insert size (20 Kb) library was sequenced on the PacBio Sequel platform with Sequel SMRT cells 1M v2 (PacBio p/n101–008-000), which has one movie of 600 minutes per Sequel SMRT cell at the Genome Center of Nextomics (Wuhan, China). A total of 7 Sequel SMRT cells were processed. To remove low-quality bases or reads with adapters, the raw data were filtered based on the sequencing platform with the default parameters. In total, 50.15 Gb of long reads clean data were obtained (Table S1). The average length and the N50 of long subreads were 10.51 Kb and 15.97 Kb, respectively.

Hi-C libraries were constructed for *A. grahami* according to the improved Hi-C procedures [49]. After treated with a 1% formaldehyde solution in PBS buffer at room temperature for 10 minutes to induce crosslinking, the single cell was made by trituration and filtration. The reaction was quenched by adding 2.5M glycine to 0.2M solution for 5 minutes. Nuclei were digested with 100 units of MboI, marked by biotin-14-dCTP (Invitrogen), and then ligated by T4 DNA Ligase. After the reversal of crosslinks, ligated DNA was purified and sheared to a length of 300-600 base pairs, at which point ligation junctions were pulled down by streptavidin beads and prepared

for high-throughput sequencing. Sequencing was performed using the Illumina NovaSeq 6000 Sequencing System (San Diego, CA, USA) with PE150, yielding 74.24 Gb raw data (Table S1).

Genome survey and Genome assembly

The genome size was estimated based on the equation G = k_{num} / k_{depth}, where the k_{num} was the total number of 17-mers, k_{depth} denoted the peak frequency of 17-mers estimated, and G represented the estimated genome size. Using Jellyfish v2.1.3 (Jellyfish, RRID: SCR 005491) [50], the number of 17-mers was counted as 29,131,491,603 from short clean reads, and the k_{depth} was 50. Therefore, the genome size of *A. grahami* was estimated as 582.63 Mb according to the above equation and the heterozygosity rate of the *A. grahami* genome was approximately 2.5% (Table S2, Fig. S1). FALCON is specifically designed to perform *de novo* assembly for PacBio long reads with about 15% random errors [51]. After correction with FALCON (v0.4), the PacBio long reads were assembled with Wtdbg (v1.2.8) [52, 53], obtaining an initial assembly with the length of approximately 596.65 Mb and N50 contig of 1.93 Mb. To further improve the accuracy of the reference assembly, the following steps of polishing strategies were performed for the initial assembly. The pbalign (v.0.3.0) with default parameters was used for Quiver error-correction, generating an error-corrected genome assembly of PacBio long reads. We used BWA v0.7.12, (BWA, RRID: SCR 010910) to map short reads to the error-corrected assembly. Then it was polished with Pilon v1.21 (Pilon, RRID: SCR 014731) to generate the second iteration of the assembled genome [54]. Finally, we obtained a polished assembly genome with a size of 600.09 Mb, including N50 contig of 1.93 Mb and 1604 contigs (Table 1, Table S3). So far, the present genome has the longest N50 contig length among all the

published genome assemblies of calyptratae flies of Diptera.

Table. 1 An overview comparison of genome assembly and structure features in five calyptratae flies of Diptera.

Parameter	*A. grahami*	*L. cuprina*	*G. morsitans*	*M. domestica*	*P. regina* (♀)
Sequencing platform	PacBio	Illumina	454/Illumina	Illumina	454/PacBio
Genome size (Mb)	600	458	366	692	550
No.of contigs/Scaffolds	1,604/7	74,043/4,436	-/13,807	-/20487	192,662/-
Contig N50 (kb)	1930	744.4	50	12	7.9
GC level (%)	31	29.3	34.1	35.1	26.2
Repetitive regions (%)	48.02	57.8	-	55	8.11
Function annotation (gene number; %)	12,791; 99.8	12,160; 83.6	12,308; 99.5	14180;92.3	7792;94
Sequencing depth	86×	100×	160×	90×	44×
Completeness (BUSCO/CEGMA; %)	99.2	96	99	98	93.6

Genome completeness was assessed by BUSCO or CEGMA. Four genomes of calyptratae fly species were selected, as *L. cuprina* [55], *G. morsitans* [56], *M. domestica* [57] and *P. regina* [45]. The genome version of *Ph. regina* female adult was chosen.

For the *A. grahami* genome, the assembly genome size (600 Mb) was almost the same as the genome size (582.63 Mb) estimated in 17-mer analysis. The sequencing quality was checked and the potentially contaminated contigs from other species were removed based on the GC content and the depth of coverage of the genome assembly analyzed by the GC Depth analysis. The completeness of the assembly was evaluated by BUSCO v3.0 (BUSCO, RRID: SCR 015008). The result of BUSCO analysis indicated that our assembly covered 99.2% complete and 0.7% partial insect BUSCOs, with only 0.5% missed (Table S4).

We also performed flow-cytometry with propidium iodide staining to estimate the genome size of *A. grahami*. *Drosophila melanogaster* (strain w118) was used as

the internal control with DNA content (pictogram: pg, 1 pg = 978 Mb) of 1C = 0.18 pg (175 Mb) [58]. The samples were prepared following the procedures of the previous study [59]. The flow-cytometry was conducted using Accuri C6 (BD, USA) with a 488nm laser. Data were processed by FlowJo software (v7.6) (Fig. S2). The estimated genome sizes of male (679.2 ± 7.582 Mb, N = 6) and female (696.4 ± 6.618 Mb, N = 6) have no significant difference (P-value = 0.1183), showing no sexual dimorphism. However, it is about 18.1% larger than the K-mer based genome size (582.63 Mb), and 14.6% larger than the assembly genome size (600.09 MB).

Functional Prediction and Genome Annotation

Analysis of repeat genes

Simple sequence repeats (SSRs) are repeating sequences of 1-6 base pairs of DNAs that exist extensively in genomes. SSRs in the blow fly genome were identified by the MIcroSAtellite Identification Tool (MISA, RRID: SCR 010765) [60]. MISA can distinguish and locate simple and complicated SSRs, of which the latter is always inserted by a certain number of nucleic acid bases. In total, 322266 SSRs were found in the *A. grahami* genome.

We also analyzed the repetitive sequences in the *A. grahami* genome including in tandem repeats and transposable elements (TEs). A Tandem Repeats Finder (TRF, v4.09) was used to annotate the tandem repeats [61]. A combination of *de novo* and homology-based approach was utilized to identify TEs at both the DNA and protein levels. First, we used RepeatModeler v1.0.8, (RepeatModeler, RRID: SCR 015027) [62] to construct a *de novo* repeat DNA library, which built a repeat consensus database with classification information. Then, the similar TEs were searched against the known Repbase library (Repbase 23.08) and *de novo* based repeat library with

RepeatMasker v4.0.6 (RepeatMasker, RRID: SCR 012954) [62]. RepeatProteinMask within the RepeatMasker package was applied used to search against the TE protein database using a WU_BLASTX engine.

Overall, the *A. grahami* genome comprised 48.02% repetitive sequences, of which 43.69% were TEs. DNA with repetitive sequences accounted for 11.65% of the *A. grahami* genome, representing the most abundant repeat class (Table 2).

Table 2 Statistics of repeat sequence analysis

Type	RepeatMasker		LTR finder		RepeatProteinMask		RepeatModeler		Combined TEs	
	Length (Mb)	% in genome	Length (Mb)	% in genome	Length (Mb)	% in genome	Length (Mb)	% in genome	Length (Mb)	% in genome
DNA	42174497	7.03	0	0	41341346	6.89	50464704	8.41	69933653	11.65
LINE	10505716	1.75	0	0	19838372	3.31	26169690	4.36	34333817	5.72
LTR	4789075	0.8	15966332	2.66	5778730	0.96	1229900	0.2	21249831	3.54
SINE	51914	0.01	0	0	0	0	453547	0.08	446000	0.07
Other*	12169475	2.02	0	0	7698698	1.28	50424873	8.4	78096062	13.02
Unknown*	161876	0.03	0	0	0	0	50424873	16	84103655	14.02
Total	69852553	11.64	15966332	2.66	74657146	12.44	224757686	37.45	288163018	48.02

***Other represents sequences with annotation but not belonging to the above types of repetitive genes, such as satellites, simple repeats, retroposon, artifact, helitron, and low complexity repeats; unknown represents sequences that cannot be classified.**

Gene prediction and functional annotation

The protein-coding genes in the *A. grahami* genome assembly were identified using *de novo*-based, homology-based, and RNA-seq-based gene prediction methods. Augustus v2.4 (Augustus, RRID: SCR 008417) [63], GlimmerHMM v3.0.4 (GlimmerHMM, RRID: SCR 002654) [64], Genemark (Genemark, RRID: SCR 011930) [65] and SNAP (SNAP, RRID: SCR 002127) [66], all trained for the *D. melanogaster* gene model before the gene prediction [67], were used in the *de novo*-based gene prediction with default parameters. GeMoMa (v1.3.1) was used to

perform the annotation of protein-coding based on the annotation of genes of *D. melanogaster*, *Glossina austeni*, *Lucilia cuprina*, *Stomoxys calcitrans* and *Musca domestica* from GenBank (Table S5) [68]. The RNA-seq-based gene prediction was performed by PASA v2.0.2 (PASA, RRID: SCR 014656) [69]. Finally, the results from the three approaches were integrated using EVidenceModeler v1.1.1, (EVM, RRID: SCR 014659) [69]. When conducting the EVM integration, PASA-predicted transcripts from unigenes and GeMoMa-predicted homologous transcripts were given higher weights than the *de novo* predicted transcripts. The gene set was aligned to the transposon database by TransposonPSI (v08222010) with default parameters [70]. Any gene of homology to transposons was removed from the final gene set. A total of 12823 protein-coding genes were identified in the *A. grahami* genome, with an average of 13240.43 bp in length and 4.62 exons per gene (Table S6).

Gene functions of the predicted protein-coding genes were annotated using two strategies. First, those predicted protein sequences were aligned to Swiss-Prot and TrEMBL protein databases using Blastall with the best match parameters [71]. The pathways of the predicted genes sequences were extracted from the KEGG Automatic Annotation Server (v2.1) [72]. Then, the annotation of motifs and domains was achieved by searching the open databases including Pfam 32.0, (Pfam, RRID: SCR 004726), ProDom v2006.1, (ProDom ,RRID: SCR 006969), PRINTS v42.0, (PRINTS, RRID: SCR 003412), PANTHER v12.0 (PANTHER, RRID: SCR 004869), SMRT (v7.1), and PROSITE v2018_02 (PROSITE, RRID: SCR 003457) with InterProScan v5.24 (InterProScan, RRID: SCR 005829) [73, 74]. The final data set was obtained by combining the results of the above two parts. In summary, 12791 genes were annotated with at least 1 related function, which accounted for about 99.8% of predicted protein-coding genes (12823) of *A. grahami* (Table 3). Additionally, the

annotation of non-coding RNA genes set was also performed based on the RNA-seq data of *A. grahami* transcriptome data (6.6G). The rRNA, snRNA, and miRNA were annotated using the non-coding database Rfam v14.0, (Rfam, RRID: SCR 007891). Then the tRNA sequence was annotated using tRNAscan-SE v2.0 (tRNAscan-SE, RRID: SCR 010835) [75]. The rRNA and subunits were predicted by RNAmmer (v1.2) [76]. As a result, a total of 126 miRNAs, 21 rRNAs, 192 snRNAs, and 859 tRNAs genes were annotated (Table S7).

Table 3 Function annotation of protein-coding genes of *A. grahami*

	**Type**	**Number**	**Percent (%)**
**Annotation**	Swiss-Prot	9648	75.2
	TrEMBL	12721	99.2
	KEGG	5247	40.9
	KOG	8252	64.4
	GO	7518	58.6
	InterProScan	10488	81.8
	Nr*	12780	99.7
**Total**	Annotated	12791	99.8
	Gene	12823	-

*Nr: Non-Redundant Protein Sequence Database

Evolutionary analyses

Gene family and phylogenetic analyses

For the prediction of the gene family, several species were selected based on genomic models, classification background, feeding habits or lifestyles such as necrophagia, polyphagia, parasitism or hematophagia. The genomic resource of *D. melanogaster, L. cuprina, M. domestica, Stomoxys calcitrans, G. austeni, Onthophagus taurus, Nicrophorus vespilloides, Blattella germanica, Cimex*

lectularius, Aedes aegypti were used (Table S5) [67, 77-85]. The OrthoMCL (OrthoMCL, RRID: SCR 007839) was employed to identify the gene families [86]. First, the amino acid sequence of the longest transcript of each gene was selected from *A. grahami* and other selected insect species. Then they were aligned reciprocally with the BLASTP (BLASTP, RRID: SCR 001010) plug-in on NCBI with a threshold of e-value less than 1e^{-5}. After that, the alignment results were clustered into family groups with default parameters. Finally, the orthologous gene families from each selected species were identified (Fig. 2). According to the results, the *A. grahami* genome contains the fewest unique genes and gene families compared to the other 10 species used in the analysis (Table 4).

Figure 2. Gene family comparison between *A. grahami* and other insect species

In total, 2989 single-copy gene families were identified among these 11 species. Firstly, each gene family was aligned using the MAFFT program (v7) at the amino-acid level [87]. All the sequence alignments were then reversely translated to nucleotide sequences. The poorly aligned positions and divergent regions were subsequently trimmed with Gblocks v0.91, (Gblocks, RRID: SCR 015945). Then, RAxML v8.2.11(RAxML, RRID: SCR 006086) was used to construct phylogenetic trees using the GTR+GAMMA model for nucleotide sequences [88] with that the branch reliability of RAxML was assessed by 100 bootstrap replicates *C. lectularius* was set as the outgroup.

Table 4 Genome families of *A. grahami* and other insect species

Species	Genes Number	Genes number In Families	Unclustered Genes number	Family Number	Unique Families Number	Average Genes Per Family
A.aegypti	14539	12810	1729	8701	485	1.47
A.grahami	12823	12033	790	10424	53	1.15

B.germanica	28670	19323	9347	9449	1286	2.04
C.lectularius	11890	9743	2147	8104	250	1.2
D.melanogaster	13872	11469	2403	9694	235	1.18
G.austeni	19722	12205	7517	9867	350	1.24
L.cuprina	15232	13915	1317	11364	560	1.22
M.domestica	14236	12968	1268	10713	133	1.21
N.vespilloides	12385	10948	1437	8961	164	1.22
O.taurus	14374	12674	1700	9222	372	1.37
P.regina (F)	8312	7536	776	6670	18	1.13
P.regina (M)	9490	7781	1709	6838	34	1.14
S.calcitrans	13469	12411	1058	10445	115	1.19

Unclustered genes and unique families represent the specific genes and families corresponding to each species.

In addition, 9 selected species were separated into different groups based on their dietary habits such as necrophagia, coprophagia, hematophagia and polyphagia (Table S8). Orthologous genes of each species were also separated as a single assemblage. The shared orthologous genes of the clusters of *A. grahami* with other Diptera species and other non-Diptera species were displayed using the online Draw Venn Diagram [89]. The results may provide candidate genes for the future research of the necrophagous lifestyle of *A. grahami* (Fig. 3).

Figure 3. Venn diagram of orthologous gene families. (A) The intersection between *A. grahami* and other Diptera species. (B) The intersection between *A. grahami* and other non-Diptera species with different dietary habits.

Divergence time and gene family expansion / contraction

The estimation of divergence time was based on the results of the gene family clustering. Four-fold degenerate sites were extracted from the alignment of coding sequences of 2989 identified single-copy gene families. The PAML MCMCTree program v4.5, (PAML, RRID: SCR 014932) was used to estimate divergence times

with the calculation of approximate likelihood test, molecular clock and substitution model of REV [90]. The primary parameters of MCMCTree were set as clock = 2 (an independent rates model following a log-normal distribution), RootAge =< 4 (400 Myr for a calibration on the root of the phylogenetic tree), model = 7 (the substitution model, REV), BDparas = 110 (default value was used here, parameters controlling the birth-death process), kappa_gamma = 62 (transition/transversion rate ratio), alpha_gamma = 11 (gamma shape parameter for variable rates among sites), rgene_gamma = 23.606 (Dirichlet-gamma prior for the mean substitution rate), sigma2_gamma = 11.03 (Dirichlet-gamma prior for the rate drift parameter). Calibrations of fossil evidence were retrieved from the TimeTree database to infer the evolutionary timescale [91].

In the phylogenetic analysis, *A. grahami* and *L. cuprina* were clustered together at first. Then with *P. regina*, it was clustered into the branch of Calliphoridae, which is next to the family Muscidae represented by *M. domestica* and *S. calcitrans*. This result is consistent with the blow fly species taxonomy that *A. grahami* diverged with *L. cuprina* from the common ancestor around 26 million years ago (Fig. 4).

Figure 4. The estimation on divergence times. The numbers beside the dots of topological branches are the divergent time to present-day (Million years ago, Mya). Red dots represent the calibration time from fossil evidence. The right lists each family name.

To further explore the gene family change under natural selection, the expansion and contraction of gene families were identified using CAFE program (CAFÉ, RRID:SCR 005983) [92]. The result revealed 102 expanded and 280 contracted gene families in the *A. grahami* genome. Additionally 198 gene families were lost from the genome (Table S9, Fig. S3).

Analysis of whole-genome duplication (WGD)

We used four-fold synonymous third-codon transversion (4DTv) [93] and Ks (a measure of synonymous substitution rate) estimation [94] to detect WGD events in the *A. grahami* genome. To this end, paralogous sequences of *A. grahami*, *Bombyx mori*, and *D. melanogaster,* were identified with OrthoMCL [86]. Then, protein sequences of these insects were aligned against each other with BLASTP (using an E-value threshold of ≤ $1e^{-5}$) to identify conserved paralogs in each species. Finally, potential WGD events in each genome were evaluated based on their 4DTv and Ks distributions. The WGD analysis suggested that *A. grahami* may have experienced the same recent WGD events as *B. mori* (Fig. S4).

Chromosome assembly using Hi-C data

To generate a chromosomal level assembly of the genome, Hi-C fragment libraries were constructed. The Hi-C libraries were sequenced on the Illumina NonaSeq 6000 (Illumina, CA, USA), generating 495 million Hi-C paired-end reads. After low-quality sequences (quality scores≤15), adaptor sequences, and sequences shorter than 30 bp were filtered out using fastp v0.12.6 (fastp, RRID: SCR 016962) [95], the clean paired-end reads were mapped to the draft assembled sequence by bowtie2 v.2.3.2 (bowtie2, RRID: SCR 005476) [96] to get the unique mapped paired-end reads. As a result, 102 million uniquely mapped paired-end reads were generated, of which 62.26% were valid interaction pairs (Table S10). Combined with the valid Hi-C data, we subsequently utilized the LACHESIS *de novo* assembly pipeline to produce chromosome-level scaffolds. As shown in Figure 5, the assembled

sequence was anchored onto the 6 pseudo-chromosomes with lengths ranging from 57.97 to 112.16 Mb (Table S11). The assembled pseudo-chromosomes (578,212,361 bp) accounted for 96.4% of the genome sequences (600,090,062 bp), with scaffold N50 values of 104.65 Mb (Table S3).

Figure 5. Hi-C interaction matrix maps within and among 6 chromosomes. The contact density was illustrated by the color bar with red (high density) to white (low density).

The similarity between the *A. grahami* genome and the published fruit fly (*D. melanogaster*) genome was analyzed [67]. The protein-coding genes from each genome were aligned using BLASTP with a threshold of E-value less than $1e^{-10}$. Then the results were combined with the GFF format files of the two genomes using MCScanX [97].

The collinearity between *A. grahami* and *D. melanogaster* genome was shown in Fig.6A. The pseudo-chromosomes of *A. grahami* and the corresponding Muller elements of *D. melanogaster* were listed (Table S11). The Muller F was reported as the X-chromosome linked in some calyptratae species [98, 99]. In the present study, however, it is hard to tell from the results of collinearity analysis that which assembled chromosome of *A. grahami* should be the Muller F. Further effort should be made to determine the sex chromosome of *A. grahami*.

In addition, we investigated the distributions of the long terminal repeat (LTR), gene family expansion or contraction, and genes under positive selection on the genome using a window size of 1 Mb across each chromosome and plotted the distributions in Fig. 6B by Circos (Circos, RRID: SCR 011798). There was no

enrichment of genes for any particular chromosomes. All the chromosomes contain a gene density of around 20 genes/per Mb. However, the results showed that longer chromosomes tend to contain a higher number of LTR, except for the case of Chr05. Besides, we noticed that the LTR was enriched in the specific regions of each chromosome where it could represent the centromere locations (Table S11).

Figure 6. Collinearity and gene clustering of the *A. grahami* genome. (A) Collinear relationship between the *A. grahami* and *D. melanogaster* genomes. The blue bar represents the *A. grahami* genome and the grey one represents the fruit fly genome. (B) Gene density distribution on chromosomes of *A. grahami*. The outer blue circle indicates the chromosomes. The inner yellow, light blue, green and orange circle represent the LTR, expanded gene family, contracted gene family and positively selected gene respectively. Window size = 1 Mb.

Conclusion

In this study, we have successfully assembled the robust draft genome of *A. grahami* through long reads *de novo* technology and Hi-C sequencing technology using the PacBio Sequel sequencing platform. This reference genome is the first chromosome-level genome assembly in calyptratae which will facilitate further genomic research of other fly species of forensic importance, and promote the transition from forensic genetics to forensic genomics [48]. This draft genome resource will be beneficial to the advancement of the study about the evolution of *A. grahami* genome. It will deepen our understanding the unique biological characteristics of *A. grahami*, such as low-temperature tolerance, seasonal distribution, necrophagous dietary habit, and its intrusion into other regions of the world. Based on qualified genome resources, studies of forensically important blow fly species will reinforce the reliability of entomological evidence and promote its application in the legal criminal investigation [100].

Availability of supporting data

Genome and transcriptome data of *A. grahami* are available in the NCBI SRA database (project accession: **PRJNA513084, SRA: SRX5207346**) and in the *GigaScience* Database, GigaDB [101]. Voucher samples information of the present work is listed in Table S12.

Additional flies

Additional File Figure S1. 17-mer Depth Distribution Curve. The x-axis represents the k-mer depth; the y-axis represents k-mer depth frequency; *Arabidopsis thaliana* (Atha for short) was set as reference.

Additional File Figure S2. Estimation of genome size of *A. grahami* by flow cytometry. Genome size (bp) was calculated from DNA content (pg) following the formula: GAg= (FAg/FDm) × GDm. GAg, DNA content of *A. grahami*, GDm, DNA content of *D. melanogaster*, FAg, fluorescence value of *A. grahami*, FDm, fluorescence value of *D. melanogaster*.

Additional File Figure S3. Expansion and contraction at the gene family level. Branch length represents divergent time; Pie chart illustrates the percentage of expansion and contraction; '+/-'means gene gain / loss.

Additional File Figure S4. Whole-genome duplication analysis of *A. grahami*, *B. mori* and *D. melanogaster*.

Additional File Table S1. Information on sequencing platform and output data.

Additional File Table S2. Genome size estimation and Heterozygosity based on 17 k-mer.

Additional File Table S3. Statistics results of genome assembly correction.

Additional File Table S4. Assessment on assembly completeness.

Additional File Table S5. Genome resource of 10 insect species for comparable genomics analysis.

Additional File Table S6. Comparison of *A. grahami* and other fly species on protein-coding genes structure and statistics.

Additional File Table S7. Functional annotation of non-coding RNA genes.

Additional File Table S8. Diet habit of 9 selected insect species.

Additional File Table S9. Statistics of gene family expansion and contraction

Additional File Table S10. Statistics of the Hi-C assembly of the *A. grahami* genome.

Additional File Table S11. Genome-wide characteristic on pseudochromosomes of the *A. grahami*.

Additional File Table S12. Information on voucher samples used in the present study.

Abbreviations

PMImin: minimum postmortem interval; *COI*: cytochrome oxidase gene I; CTAB: Cetyltrimethyl Ammonium Bromide; WGS: Whole-genome shotgun sequencing; Hi-C: Chromosome conformation capture; SSR: Simple sequence repeats; TEs: Transposable elements; TRF: Tandem Repeats Finder; LTR: long terminal repeat; Mya: Million years ago; 4DTv: four-fold synonymous third-codon transversion; BUSCO: benchmarking universal single-copy orthologs; GO: gene ontology; KEGG: Kyoto Encyclopedia of Genes and Genomes; SMRT: single-molecule real time; WGD: Whole-genome duplication

Competing interests

All authors declare that no competing interests.

Funding

The present study was supported by the grant of the National Natural Science Foundation of China (81571855) and Science Foundation of Hunan Province (2017SK2015).

Author contributions

F. M., J.C. designed the project. F. M., M. Z., Y. W., and C.C. analyzed the data. H.H., Z. L., Y. J. prepared the samples and conducted the experiments. F. M., D.F., and Z. S. wrote and revised the manuscript. J.C. supervised the whole program and coordinated the group. Y. G. provided material and equipment for the breeding of insects.

Reference

1. Catts EP, Goff ML. Forensic entomology in criminal investigations. Annual review of entomology. 1992;37:253-72. doi:10.1146/annurev.en.37.010192.001345.
2. Benecke M. A brief history of forensic entomology. Forensic science international. 2001;120 1-2:2-14.
3. Schoenly KA. statistical analysis of successional patterns in carrion-arthropod assemblages: implications for forensic entomology and determination of the postmortem interval. Journal of forensic sciences. 1992;37 6:1489-513.
4. Tomberlin JK, Mohr R, Benbow ME, Tarone AM, VanLaerhoven S. A roadmap for bridging basic and applied research in forensic entomology. Annual review of entomology. 2011;56:401-21. doi:10.1146/annurev-ento-051710-103143.
5. Benecke M, Lessig R. Child neglect and forensic entomology. Forensic science international. 2001;120 1-2:155-9.
6. Campobasso CP, Gherardi M, Caligara M, Sironi L, Introna F. Drug analysis in blowfly larvae and in human tissues: a comparative study. International journal of legal medicine. 2004;118 4:210-4. doi:10.1007/s00414-004-0448-1.
7. Castner LC, Byrd JH. Insects of Forensic Importance. In: Castner LC, Byrd JH, editor. Forensic entomology : the utility of arthropods in legal investigations. Boca Raton, London: CRC Press; 2009. p. 44-6.

8. Anderson GS. Minimum and maximum development rates of some forensically important Calliphoridae (Diptera). Journal of forensic sciences. 2000;45 4:824-32.

9. Harvey ML, Gaudieri S, Villet MH, Dadour IR. A global study of forensically significant calliphorids: Implications for identification. Forensic science international. 2008;177 1:66-76. doi:10.1016/j.forsciint.2007.10.009.

10. Norris KR. The Bionomics of Blow Flies. Annreventomol. 1965;10 1:47-68.

11. Baumgartner DL, Greenberg B. The Genus Chrysomya (Diptera: Calliphoridae) in the New World. Journal of medical entomology. 1984;21 1:105-13.

12. Tarone AM, Sanford MR. Is PMI the Hypothesis or the Null Hypothesis? Journal of medical entomology. 2017;54 5:1109-15. doi:10.1093/jme/tjx119.

13. Tarone AM, Picard CJ, Spiegelman C, Foran DR. Population and temperature effects on Lucilia sericata (Diptera: Calliphoridae) body size and minimum development time. Journal of medical entomology. 2011;48 5:1062-8.

14. Zhao B, Wen C, Qi LL, Wang H, Wang J. [Biological characteristics of calliphoridae and its application in forensic medicine]. Fa yi xue za zhi. 2013;29 6:447-50.

15. Fan ZD. Key to the common flies of China. Beijing, China: Science publishing house; 1992.

16. Aldrich JM. New two-winged flies of the family Calliphoridae from China. Proceedings of the United States National Museum. 1930.

17. Dodge HR. Identifying common flies. Public health reports. 1953;68 3:345-50.

18. Nunez-Vazquez C, Tomberlin J, Garcia-Martinez O. First Record of the Blow Fly Calliphora grahami from Mexico. Southwestern Entomologist. 2010;35 3:313-6. doi:Doi 10.3958/059.035.0310.

19. Whitworth T. Keys to the genera and species of blow flies (Diptera : Calliphoridae) of America North of Mexico. P Entomol Soc Wash. 2006;108 3:689-725.

20. Wang Y, Zhang YN, Liu C, Hu GL, Wang M, Yang LJ, et al. Development of Aldrichina grahami (Diptera: Calliphoridae) at Constant Temperatures. Journal of medical entomology. 2018;55 6:1402-9. doi:10.1093/jme/tjy128.

21. Chen W, Yang L, Ren L, Shang Y, Wang S, Guo Y. Impact of Constant Versus Fluctuating Temperatures on the Development and Life History Parameters of Aldrichina grahami (Diptera: Calliphoridae). Insects. 2019;10 7 doi:10.3390/insects10070184.

22. Kurahashi H, Kawai S, Shudo C, Wada Y. Seasonal prevalence of adult fly and life cycle of aldrichina grahami (aldrich) in tokyo. Medical Entomology & Zoology. 1984;35 3:261-7.

23. Guo YD, Cai JF, Tang ZC, Feng XO, Lin Z, Yong F, et al. Application of Aldrichina grahami (Diptera, Calliphoridae) for forensic investigation in central-south China. Romanian Journal Of Legal Medicine. 2011;19 1:55-8. doi:10.4323/rjlm.2011.55.

24. Wang JF, Hu C, Min JX, Chen YC, Li JT. Chronometrical morphology of Aldrichina grahami and its application in the determination of postmortem interval. Acta Entomol Sin. 2002;45 2002:265-70.

25. Kurahashi H, Kawai S, Shudo C. Seasonal migration of Japanese blow flies, Aldrichina grahami (Aldrich) and Calliphora nigribarbis Vollenhoven, observed by a mark and recapture method on Hachijo Island, Tokyo. Medical Entomology & Zoology. 1991; 42:57-9.

26. Zehner R, Amendt J, Krettek R. STR typing of human DNA from fly larvae fed on decomposing bodies. Journal of forensic sciences. 2004;49 2:337-40.

27. Li K, Ye GY, Zhu JY, Hu C. Detection of food source by PCR analysis of the gut contents of

Aldrichina grahami (Aldrich) (Diptera: Calliphoridae) during post-feeding period. Insect Sci. 2007;14 1:47-52. doi:10.1111/j.1744-7917.2007.00124.x.

28. Xu H, Ye GY, Xu Y, Hu C, Zhu GH. Age-dependent changes in cuticular hydrocarbons of larvae in Aldrichina grahami (Aldrich) (Diptera: Calliphoridae). Forensic science international. 2014;242:236-41. doi:10.1016/j.forsciint.2014.07.003.

29. Moore HE, Adam CD, Drijfhout FP. Potential Use of Hydrocarbons for Aging Lucilia sericata Blowfly Larvae to Establish the Postmortem Interval. Journal of forensic sciences. 2013;58 2:404-12. doi:10.1111/1556-4029.12016.

30. Liu YL. A case report of gastrointestinal myiasis caused by Aldrichina grahami. Acta Medicinae Universitatis Scientiae et Technologiae Huazhong. 1980;2:81-2.

31. Li XL, Xu ZQ. A case of human gastrointestinal myiasis. Bulletin of Disease Control & Prevention. 2006;21 1:107.

32. Cao XL, Sang YH, Yang YL, Wang S. Comprehensive analyses on Chinese human myiasis cases of 2003-2013. Guide of China Medicine. 2015;8:37-9.

33. Lachish T, Marhoom E, Mumcuoglu KY, Tandlich M, Schwartz E. Myiasis in Travelers. Journal of travel medicine. 2015;22 4:232-6. doi:10.1111/jtm.12203.

34. Sawabe K, Hoshino K, Isawa H, Sasaki T, Hayashi T, Tsuda Y, et al. Detection and isolation of highly pathogenic H5N1 avian influenza A viruses from blow flies collected in the vicinity of an infected poultry farm in Kyoto, Japan, 2004. The American journal of tropical medicine and hygiene. 2006;75 2:327-32.

35. Miura K, Takaya T, Koshiba K. The effect of biotin deficiency on the biosynthesis of the fatty acids in a blowfly, Aldrichina grahami during metamorphosis under aseptic conditions. Archives internationales de physiologie et de biochimie. 1967;75 1:65-76.

36. Tohoru H, Akira W, Kazuo Miura. Properties and regulation of xanthine dehydrogenase of a blowfly, Aldrichina grahami. Insect Biochemistry, 1977, 7(4):317-322. 1977;7 4:317-22.

37. Wadano A MK. Urate oxidase in the blowfly, Aldrichina grahami. Insect Biochemistry. 1976;6 3:321-5.

38. Wadano A, Miura, K, Ihara, H, Kondo N, Taniguchi, MA. Purification and some properties of isocitrate dehydrogenase of a blowfly aldrichina grahami. Comparative Biochemistry and Physiology B. 1989;94 1:189-94.

39. Meng FM, Ren LP, Wang Z, Deng J, Guo YD, Chen C, et al. Identification of Forensically Important Blow Flies (Diptera: Calliphoridae) in China Based on COI. Journal of medical entomology. 2017;54 5:1193-200. doi:10.1093/jme/tjx105.

40. Zaidi F, Wei SJ, Shi M, Chen XX. Utility of multi-gene loci for forensic species diagnosis of blowflies. Journal of insect science. 2011;11.

41. Park SH, Park CH, Zhang Y, Piao H, Chung U, Kim SY, et al. Using the Developmental Gene Bicoid to Identify Species of Forensically Important Blowflies (Diptera: Calliphoridae). BioMed research international. 2013; doi:Artn 53805110.1155/2013/538051.

42. Zhu ZY, Liao HD, Ling J, Guo YD, Cai JF, Ding YJ. The complete mitochondria genome of Aldrichina grahami (Diptera: Calliphoridae). Mitochondrial DNA B. 2016;1:107-9. doi:10.1080/23802359.2015.1137847.

43. Gallagher MB, Sandhu S, Kimsey R. Variation in developmental time for geographically distinct populations of the common green bottle fly, Lucilia sericata (Meigen). Journal of forensic sciences. 2010;55 2:438-42. doi:10.1111/j.1556-4029.2009.01285.x.

44. Hu Y, Yuan X, Zhu F, Lei C. Development time and size-related traits in the oriental blowfly, chrysomya megacephala along a latitudinal gradient from china. Journal of Thermal Biology. 2010;35 7:366-71.
45. Andere A, Platt RN, Ray DA and Picard CJ. Genome sequence of Phormia regina Meigen (Diptera: Calliphoridae): implications for medical, veterinary and forensic research. BMC genomics. 2016;17 1:842. doi:10.1186/s12864-016-3187-z.
46. Zajac BK, Amendt J, Verhoff MA, Zehner R. Dating Pupae of the Blow Fly Calliphora vicina Robineau-Desvoidy 1830 (Diptera: Calliphoridae) for Post Mortem Interval-Estimation: Validation of Molecular Age Markers. Genes. 2018;9 3 doi:Artn 15310.3390/Genes9030153.
47. Arenas M, Pereira F, Oliveira M, Pinto N, Lopes AM, Gomes V, et al. Forensic genetics and genomics: Much more than just a human affair. PLoS genetics. 2017;13 9:e1006960. doi:10.1371/journal.pgen.1006960.
48. Kayser M, Parson W. Transitioning from Forensic Genetics to Forensic Genomics. Genes. 2017;9 1 doi:10.3390/genes9010003.
49. Rao SSP, Huntley MH, Durand NC, Stamenova EK, Bochkov ID, Robinson JT, et al. A 3D Map of the Human Genome at Kilobase Resolution Reveals Principles of Chromatin Looping. Cell. 2014;159 7:1665-80. doi:10.1016/j.cell.2014.11.021.
50. Marcais G, Kingsford C. A fast, lock-free approach for efficient parallel counting of occurrences of k-mers. Bioinformatics. 2011;27 6:764-70. doi:10.1093/bioinformatics/btr011.
51. Eid J, Fehr A, Gray J, Luong K, Lyle J, Otto G, et al. Real-time DNA sequencing from single polymerase molecules. Science. 2009;323 5910:133-8. doi:10.1126/science.1162986.
52. WTDBG package：https://github.com/ruanjue/wtdbg. (Accessed 10 Jan 2018).
53. Falcon: https://github.com/PacificBiosciences/FALCON.
54. Walker BJ, Abeel T, Shea T, Priest M, Abouelliel A, Sakthikumar S, et al. Pilon: an integrated tool for comprehensive microbial variant detection and genome assembly improvement. PloS one. 2014;9 11:e112963. doi:10.1371/journal.pone.0112963.
55. Anstead CA, Korhonen PK, Young ND, Hall RS, Jex AR, Murali SC, et al. Lucilia cuprina genome unlocks parasitic fly biology to underpin future interventions. Nature communications. 2015;6:7344. doi:10.1038/ncomms8344.
56. Watanabe J, Hattori M, Berriman M, Lehane MJ, Hall N, Solano P, et al. Genome sequence of the tsetse fly (Glossina morsitans): vector of African trypanosomiasis. Science. 2014;344 6182:380-6. doi:10.1126/science.1249656.
57. Scott JG, Warren WC, Beukeboom LW, Bopp D, Clark AG, Giers SD, et al. Genome of the house fly, Musca domestica L., a global vector of diseases with adaptations to a septic environment. Genome biology. 2014;15 10:466. doi:10.1186/s13059-014-0466-3.
58. Bennett MD, Leitch IJ, Price HJ, Johnston JS. Comparisons with Caenorhabditis (approximately 100 Mb) and Drosophila (approximately 175 Mb) using flow cytometry show genome size in Arabidopsis to be approximately 157 Mb and thus approximately 25% larger than the Arabidopsis genome initiative estimate of approximately 125 Mb. Annals of botany. 2003;91 5:547-57. doi:10.1093/aob/mcg057.
59. Picard CJ, Johnston JS, Tarone AM. Genome sizes of forensically relevant Diptera. Journal of medical entomology. 2012;49 1:192-7. doi:10.1603/me11075.
60. Thiel T, Michalek W, Varshney RK, Graner A. Exploiting EST databases for the development and characterization of gene-derived SSR-markers in barley (Hordeum vulgare L.). TAG

Theoretical and applied genetics Theoretische und angewandte Genetik. 2003;106 3:411-22. doi:10.1007/s00122-002-1031-0.

61. Benson G. Tandem repeats finder: a program to analyze DNA sequences. Nucleic acids research. 1999;27 2:573-80. doi:10.1093/nar/27.2.573.

62. Bedell JA, Korf I, Gish W. MaskerAid: a performance enhancement to RepeatMasker. Bioinformatics. 2000;16 11:1040-1. doi:10.1093/bioinformatics/16.11.1040.

63. Stanke M, Waack S. Gene prediction with a hidden Markov model and a new intron submodel. Bioinformatics. 2003;19 Suppl 2:ii215-25. doi:10.1093/bioinformatics/btg1080.

64. Majoros WH, Pertea M, Salzberg SL. TigrScan and GlimmerHMM: two open source ab initio eukaryotic gene-finders. Bioinformatics. 2004;20 16:2878-9. doi:10.1093/bioinformatics/bth315.

65. Besemer J, Borodovsky M. GeneMark: web software for gene finding in prokaryotes, eukaryotes and viruses. Nucleic acids research. 2005;33 Web Server issue:W451-4. doi:10.1093/nar/gki487.

66. Johnson AD, Handsaker RE, Pulit SL, Nizzari MM, O'Donnell CJ, De Bakker PI. SNAP: a web-based tool for identification and annotation of proxy SNPs using HapMap. Bioinformatics. 2008;24 24:2938-9. doi:10.1093/bioinformatics/btn564.

67. NCBI Genome. https://www.ncbi.nlm.nih.gov/genome/47.

68. Keilwagen J, Wenk M, Erickson JL, Schattat MH, Grau J, Hartung F. Using intron position conservation for homology-based gene prediction. Nucleic acids research. 2016;44 9 doi:ARTN e8910.1093/nar/gkw092.

69. Haas BJ, Salzberg SL, Zhu W, Pertea M, Allen JE, Orvis J, et al. Automated eukaryotic gene structure annotation using EVidenceModeler and the program to assemble spliced alignments. Genome biology. 2008;9 1 doi:Artn R710.1186/Gb-2008-9-1-R7.

70. Yagi M, Kosugi S, Hirakawa H, Ohmiya A, Tanase K, Harada T, et al. Sequence Analysis of the Genome of Carnation (Dianthus caryophyllus L.). DNA Research. 2014;21 3:231-41. doi:10.1093/dnares/dst053.

71. Bairoch A, Apweiler R, Wu CH, Barker WC, Boeckmann B, Ferro S, et al. The universal protein resource (UniProt). Nucleic acids research. 2005;33:D154-D9. doi:10.1093/nar/gki070.

72. Kanehisa M, Goto S. KEGG: Kyoto Encyclopedia of Genes and Genomes. Nucleic acids research. 2000;28 1:27-30. doi:Doi 10.1093/Nar/28.1.27.

73. Hunter S, Apweiler R, Attwood TK, Bairoch A, Bateman A, Binns D, et al. InterPro: the integrative protein signature database. Nucleic acids research. 2009;37:D211-D5. doi:10.1093/nar/gkn785.

74. Zdobnov EM, Apweiler R. InterProScan - an integration platform for the signature-recognition methods in InterPro. Bioinformatics. 2001;17 9:847-8. doi:DOI 10.1093/bioinformatics/17.9.847.

75. Lowe TM, Eddy SR. tRNAscan-SE: a program for improved detection of transfer RNA genes in genomic sequence. Nucleic acids research. 1997;25 5:955-64. doi:10.1093/nar/25.5.955.

76. Lagesen K, Hallin P, Rodland EA, Staerfeldt HH, Rognes T, Ussery DW. RNAmmer: consistent and rapid annotation of ribosomal RNA genes. Nucleic acids research. 2007;35 9:3100-8. doi:10.1093/nar/gkm160.

77. NCBI Genome. https://www.ncbi.nlm.nih.gov/genome/12732

78. NCBI Genome. https://www.ncbi.nlm.nih.gov/genome/14461.

79. NCBI Genome. https://www.ncbi.nlm.nih.gov/genome/11278
80. NCBI Genome. https://www.ncbi.nlm.nih.gov/genome/11279.
81. NCBI Genome. https://www.ncbi.nlm.nih.gov/genome/12827.
82. NCBI Genome. https://www.ncbi.nlm.nih.gov/genome/13223.
83. NCBI Genome. https://www.ncbi.nlm.nih.gov/genome/16689.
84. NCBI Genome. https://www.ncbi.nlm.nih.gov/genome/40824.
85. NCBI Genome. https://www.ncbi.nlm.nih.gov/genome/44.
86. Li L, Stoeckert CJ, Roos DS. OrthoMCL: Identification of ortholog groups for eukaryotic genomes. Genome research. 2003;13 9:2178-89. doi:10.1101/gr.1224503.
87. Katoh K, Standley DM. MAFFT multiple sequence alignment software version 7: improvements in performance and usability. Molecular biology and evolution. 2013;30 4:772-80. doi:10.1093/molbev/mst010.
88. Stamatakis A. RAxML-VI-HPC: Maximum likelihood-based phylogenetic analyses with thousands of taxa and mixed models. Bioinformatics. 2006;22 21:2688-90. doi:10.1093/bioinformatics/btl446.
89. Draw Venn Diagram. http://bioinformatics.psb.ugent.be/webtools/Venn/.
90. Yang Z. PAML: a program package for phylogenetic analysis by maximum likelihood. Computer applications in the biosciences : CABIOS. 1997;13 5:555-6.
91. Hedges SB, Dudley J, Kumar S. TimeTree: a public knowledge-base of divergence times among organisms. Bioinformatics. 2006;22 23:2971-2. doi:10.1093/bioinformatics/btl505.
92. De Bie T, Cristianini N, Demuth JP, Hahn MW. CAFE: a computational tool for the study of gene family evolution. Bioinformatics. 2006;22 10:1269-71. doi:10.1093/bioinformatics/btl097.
93. Kimura M. A simple method for estimating evolutionary rates of base substitutions through comparative studies of nucleotide sequences. Journal of molecular evolution. 1980;16 2:111-20.
94. Blanc G, Wolfe KH. Widespread paleopolyploidy in model plant species inferred from age distributions of duplicate genes. The Plant cell. 2004;16 7:1667-78. doi:10.1105/tpc.021345.
95. Chen S, Zhou Y, Chen Y, Gu J. fastp: an ultra-fast all-in-one FASTQ preprocessor. Bioinformatics. 2018;34 17:i884-i90. doi:10.1093/bioinformatics/bty560.
96. Langmead B, Salzberg SL. Fast gapped-read alignment with Bowtie 2. Nature methods. 2012;9 4:357-9. doi:10.1038/nmeth.1923.
97. Wang Y, Tang H, Debarry JD, Tan X, Li J, Wang X, et al. MCScanX: a toolkit for detection and evolutionary analysis of gene synteny and collinearity. Nucleic acids research. 2012;40 7:e49. doi:10.1093/nar/gkr1293.
98. Linger R J, Belikoff E J, Scott M J. Dosage Compensation of X-Linked Muller Element F Genes but Not X-Linked Transgenes in the Australian Sheep Blowfly. PLOS ONE. 2015; 10.10(2015):e0141544.
99. Landeen EL, Presgraves DC. Evolution: From Autosomes to Sex Chromosomes - and Back. Current biology. 2017;CB. 23. R848-50. 10.1016/j.cub.2013.08.021
100. Jager AC, Alvarez ML, Davis CP, Guzman E, Han Y, Way L, et al. Developmental validation of the MiSeq FGx Forensic Genomics System for Targeted Next Generation Sequencing in Forensic DNA Casework and Database Laboratories. Forensic science international Genetics. 2017;28:52-70. doi:10.1016/j.fsigen.2017.01.011.

101. Meng, Liu Z, Cai J, Han H, Finkelbergs D, Jiang Y et al. Supporting data for "Chromosomal-level genome assembly of Aldrichina grahami, a forensically important blow fly". GigaScience Database 2019. http://dx.doi.org/10.5524/100673

 Click here to access/download;Table;Table S9.xlsx

Table S9. Stati

Branch	Branch Length	N	Families
C.lectularius	355.288	5587	204
B.germanica	341.947	5559	370
A.aegypti	238.846	5789	488
D.melanogaster	141.291	5845	181
G.austeni	82.4772	5763	487
M.domestica	31.9392	5772	262
S.calcitrans	31.9392	5776	132
S.calcitrans |*M.domestica*	23.7435	5858	74
P.regina (M)	5.2424	4097	189
P.regina (F)	5.2424	4036	128
P.regina (F)|*P.regina* (M)	23.3645	4374	114
L.cuprina	14.4816	5836	168
A.grahami	14.4816	5761	98
A.grahami |*L.cuprina*	14.1253	5870	39
A.grahami ,*L.cuprina* |*P.regina* (F),*P.regina* (M)	27.0758	5870	17
A.grahami ,*L.cuprina* ,*P.regina* (F),*P.regina* |*S.calcitrans* ,*I*	26.7946	5870	38
A.grahami ,*L.cuprina* ,*P.regina(F)* ,*P.regina* ,*S.calcitrans* ,*I*	58.8136	5870	80
A.grahami ,*L.cuprina* ,*P.regina* (F),*P.regina* ,*S.calcitrans* ,*I*	97.5555	5870	86
A.grahami,*L.cuprina*,*P.regina* (F),*P.regina*,*S.calcitrans*,*M.*	70.0372	5870	54
N.vespilloides	283.738	5771	222
O.taurus	283.738	5779	461
O.taurus |*N.vespilloides*	25.1456	5870	18
O.taurus,*N.vespilloides* |*A.grahami*,*L.cuprina*,*P.regina(F)*,.	33.0629	5870	9
O.taurus,*N.vespilloides*,*A.grahami*,*L.cuprina*,*P.regina(F)*,*I*	13.3412	5870	0

*** Branch, brances on evolutionary tree; N, number of gene family; Gene Gain/Family, average ge**

istics of gene family expansion and contraction

Expansions		Contractions			Extinctions	
Genes	Gene Gain/Family	Families	Genes	Gene Loss/Family	Families	Genes
274	1.34314	415	469	1.13012	283	311
505	1.36486	436	482	1.1055	311	329
867	1.77664	132	139	1.05303	81	82
239	1.32044	71	78	1.09859	25	28
581	1.19302	188	216	1.14894	107	108
327	1.24809	131	131	1	86	86
173	1.31061	139	140	1.00719	82	82
113	1.52703	48	50	1.04167	12	12
198	1.04762	314	324	1.03185	277	279
131	1.02344	374	376	1.00535	338	338
134	1.17544	1654	1717	1.03809	1496	1514
210	1.25	72	75	1.04167	34	34
150	1.53061	174	183	1.05172	109	116
47	1.20513	29	29	1	0	0
17	1	30	31	1.03333	0	0
44	1.15789	19	19	1	0	0
82	1.025	18	41	2.27778	0	0
94	1.09302	69	88	1.27536	0	0
76	1.40741	48	60	1.25	0	0
352	1.58559	200	236	1.18	99	110
719	1.55965	177	188	1.06215	91	95
20	1.11111	3	3	1	0	0
10	1.11111	22	23	1.04545	0	0
0	NA	0	0	NA	0	0

ene expanding in each family; Avg./Exp=(total genes gained along branch - total genes lost along branch)/to

s		
Gene Extinct/Family	No Change	Avg. Exp.
1.09894	5251	-0.0332198
1.05788	5064	0.00391823
1.01235	5250	0.12402
1.12	5618	0.0274276
1.00935	5195	0.0621806
1	5465	0.0333901
1	5587	0.00562181
1	5748	0.0107325
1.00722	3871	-0.0214651
1	3872	-0.0417376
1.01203	4102	-0.269676
1	5630	0.0229983
1.06422	5598	-0.00562181
NA	5802	0.00306644
NA	5823	-0.00238501
NA	5813	0.00425894
NA	5772	0.00698467
NA	5715	0.00102215
NA	5768	0.00272572
1.11111	5448	0.0197615
1.04396	5232	0.09046
NA	5849	0.00289608
NA	5839	-0.00221465
NA	5870	0

otal genes at ancestral node of branch.

 Click here to access/download;Table;Table S11.xlsx

able S11. Genome-wide characteristic on pseudoc

	Muller element of *D. melanogaster*	Length	Scaf Num	all genes	Density (/mb)	positively	Density (/mb)
Chr01	Muller B	112,158,196	226	2119	18.89	23	0.205068
Chr02	Muller D	109,743,127	180	2411	21.97	32	0.29159
Chr03	Muller A	104,650,035	162	2424	23.16	29	0.277114
Chr04	Muller C	104,176,436	114	2499	23.99	21	0.201581
Chr05	_	89,513,788	61	1804	20.15	16	0.178743
Chr06	Muller E	57,970,779	50	1305	22.51	10	0.172501

hromosomes of the *A. graham*

expansion	Density (/mb)	contraction	Density (/mb)	LTR	Density (/mb)
50	0.445799	21	0.187236	14529	129.5402
54	0.492058	18	0.164019	11038	100.5803
51	0.487339	20	0.191113	8843	84.50069
65	0.623942	40	0.383964	9936	95.37666
43	0.480373	26	0.290458	9998	111.6923
43	0.741753	10	0.172501	3758	64.82576

Figure 1

Click here to access/download;Figure;Figure 1.pdf

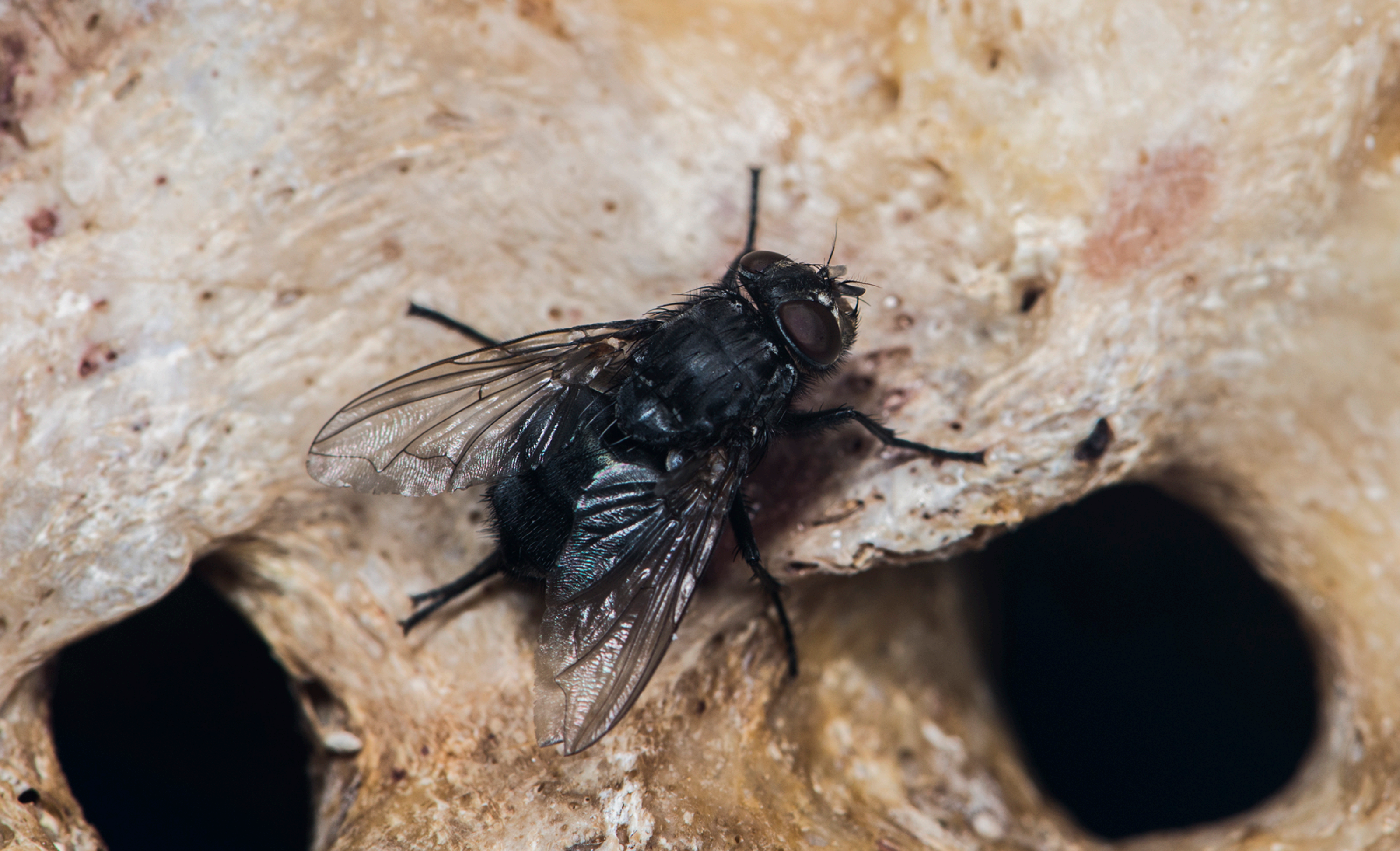

Figure 2

Click here to access/download;Figure;Figure 2.pdf

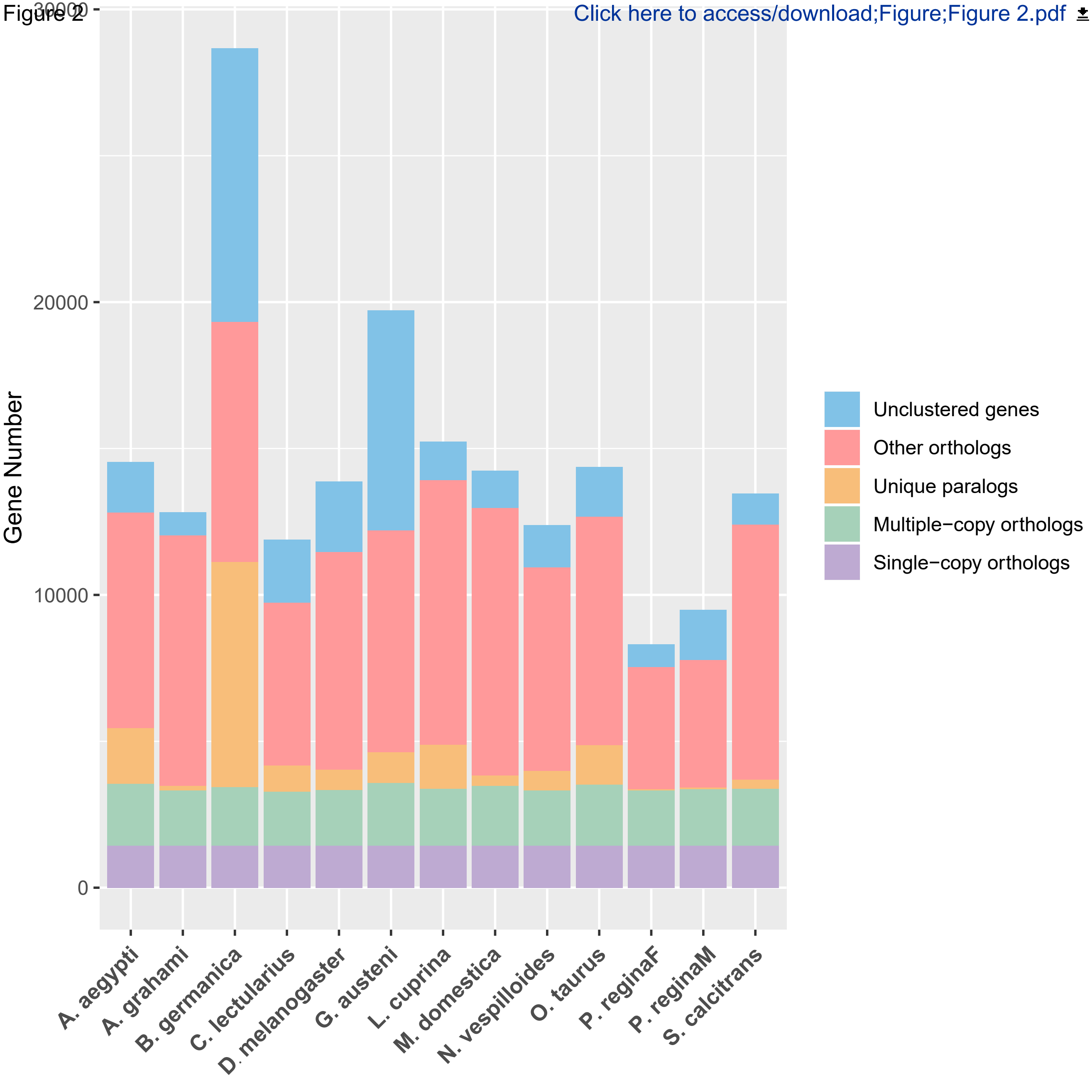

Figure 3

Click here to access/download;Figure;Figure 3.pdf

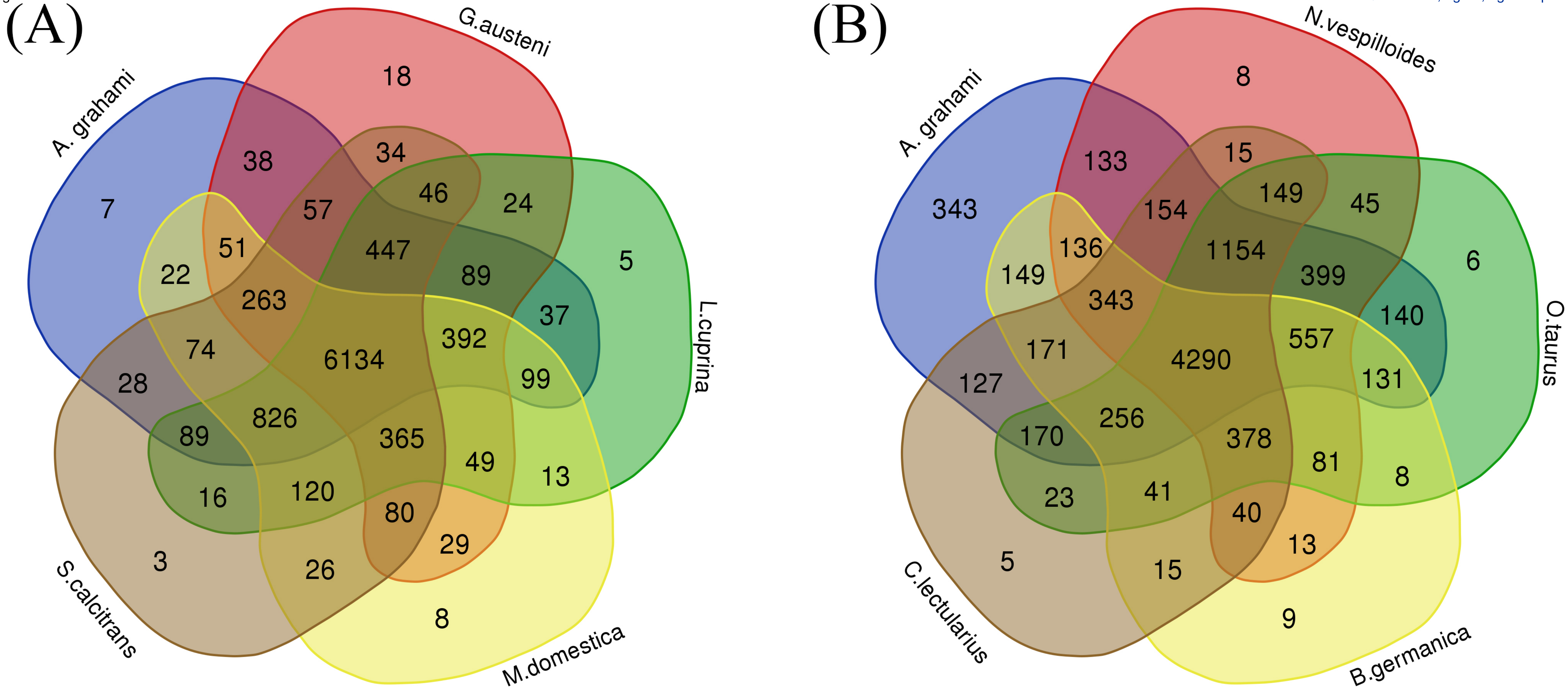

Figure 4

Click here to access/download;Figure;Figure 4.pdf

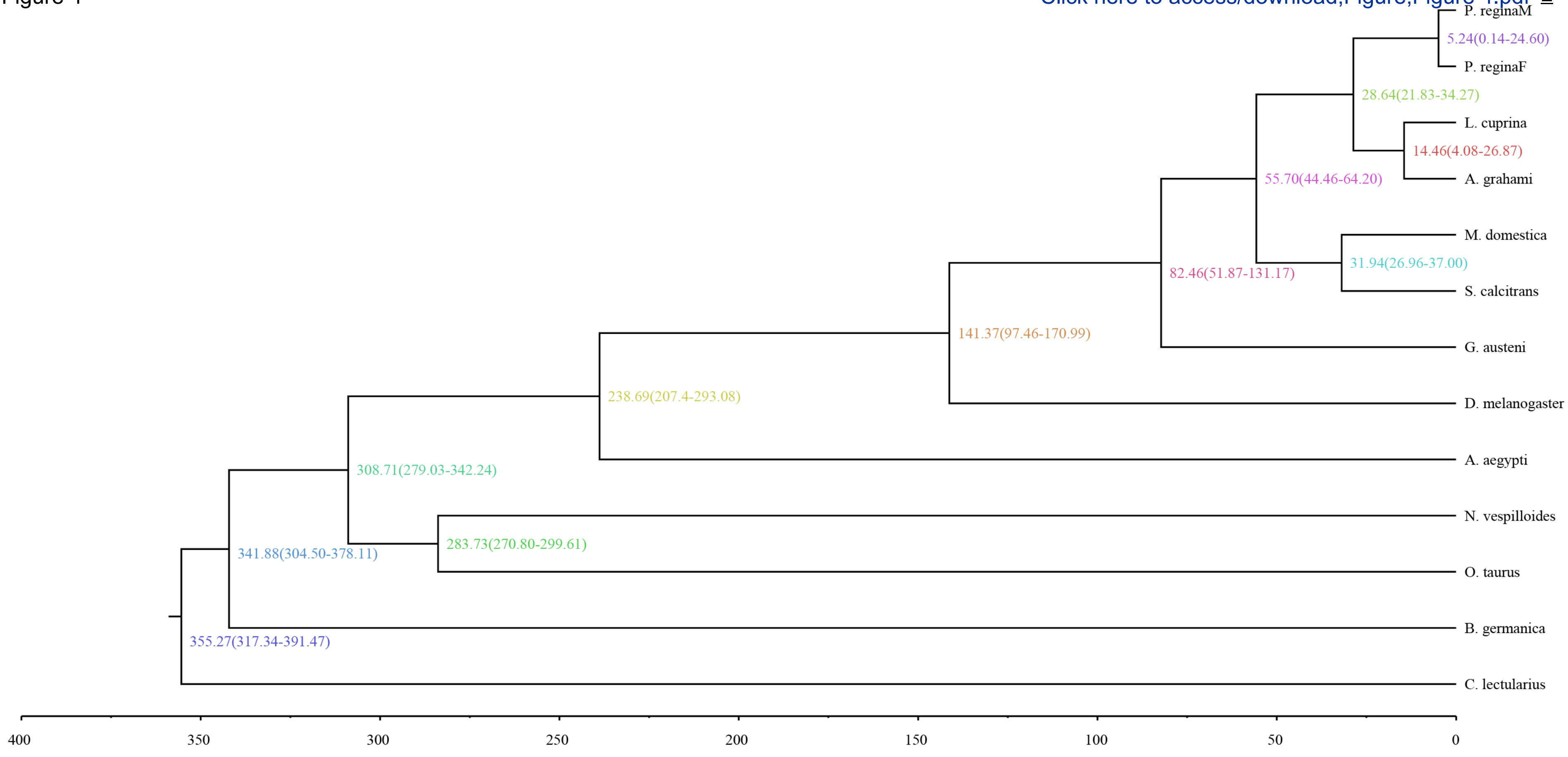

Figure 5

Click here to access/download;Figure;Figure 5.pdf

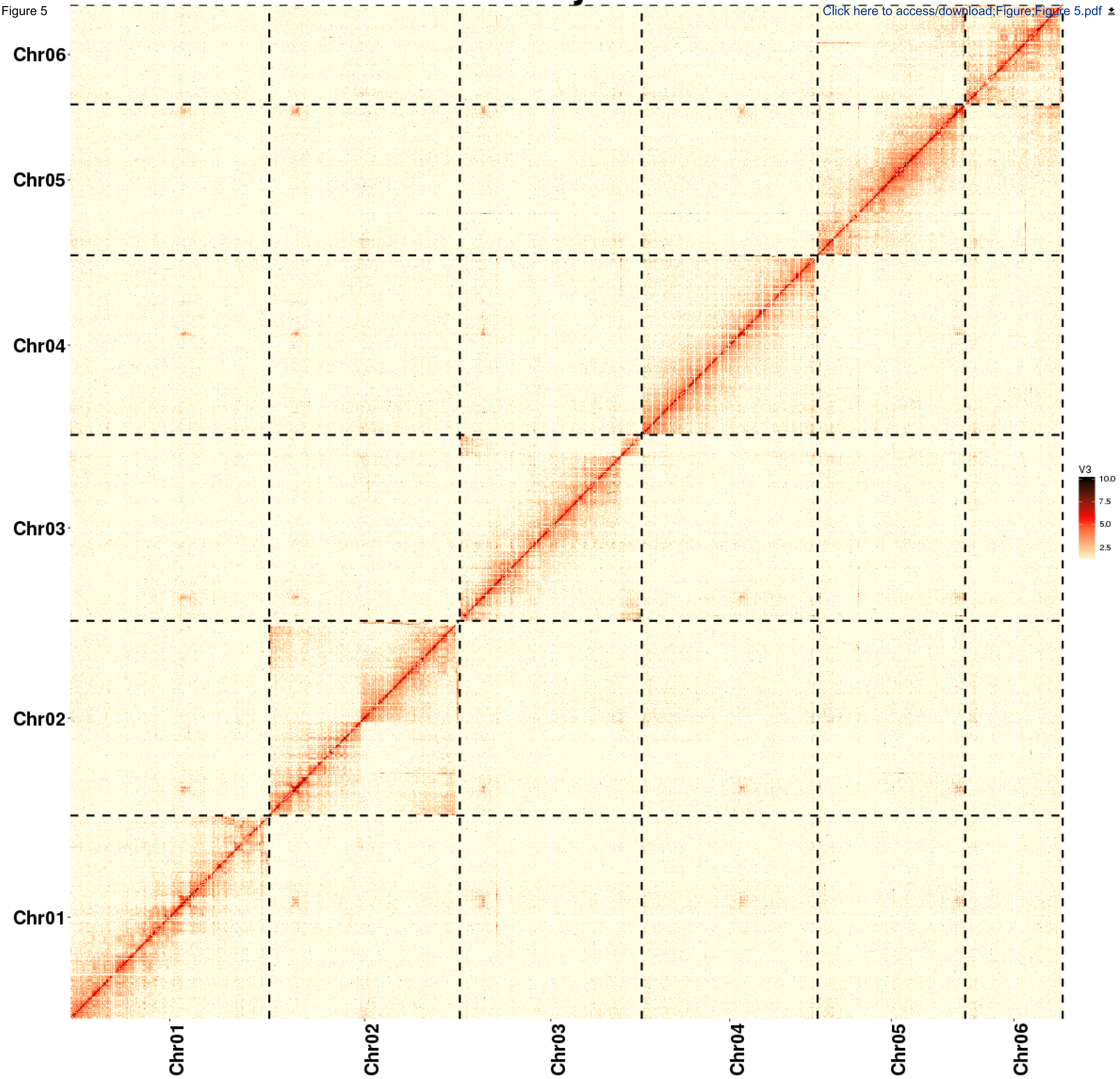

Figure 6

Click here to access/download;Figure;Figure 6.pdf

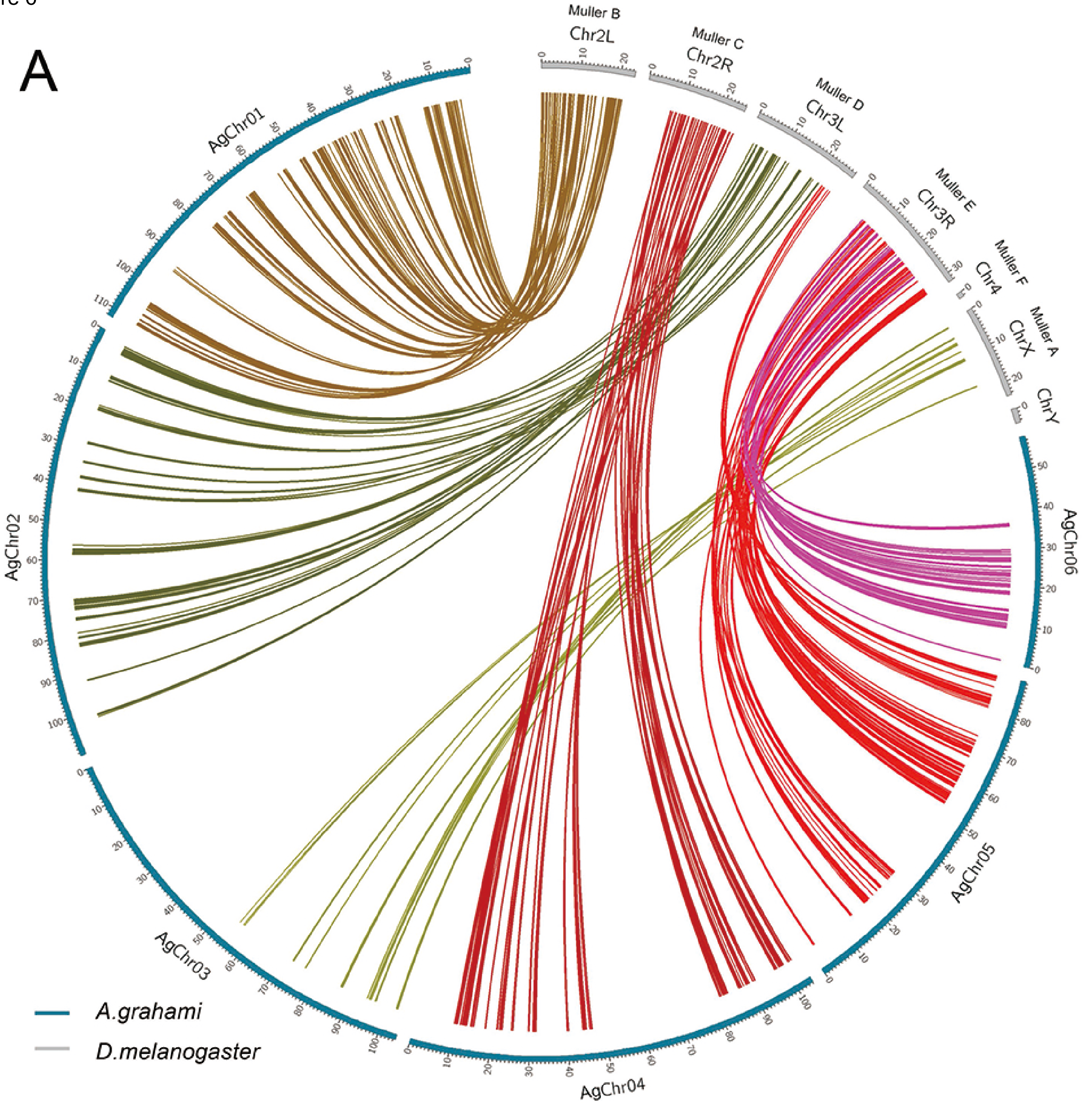

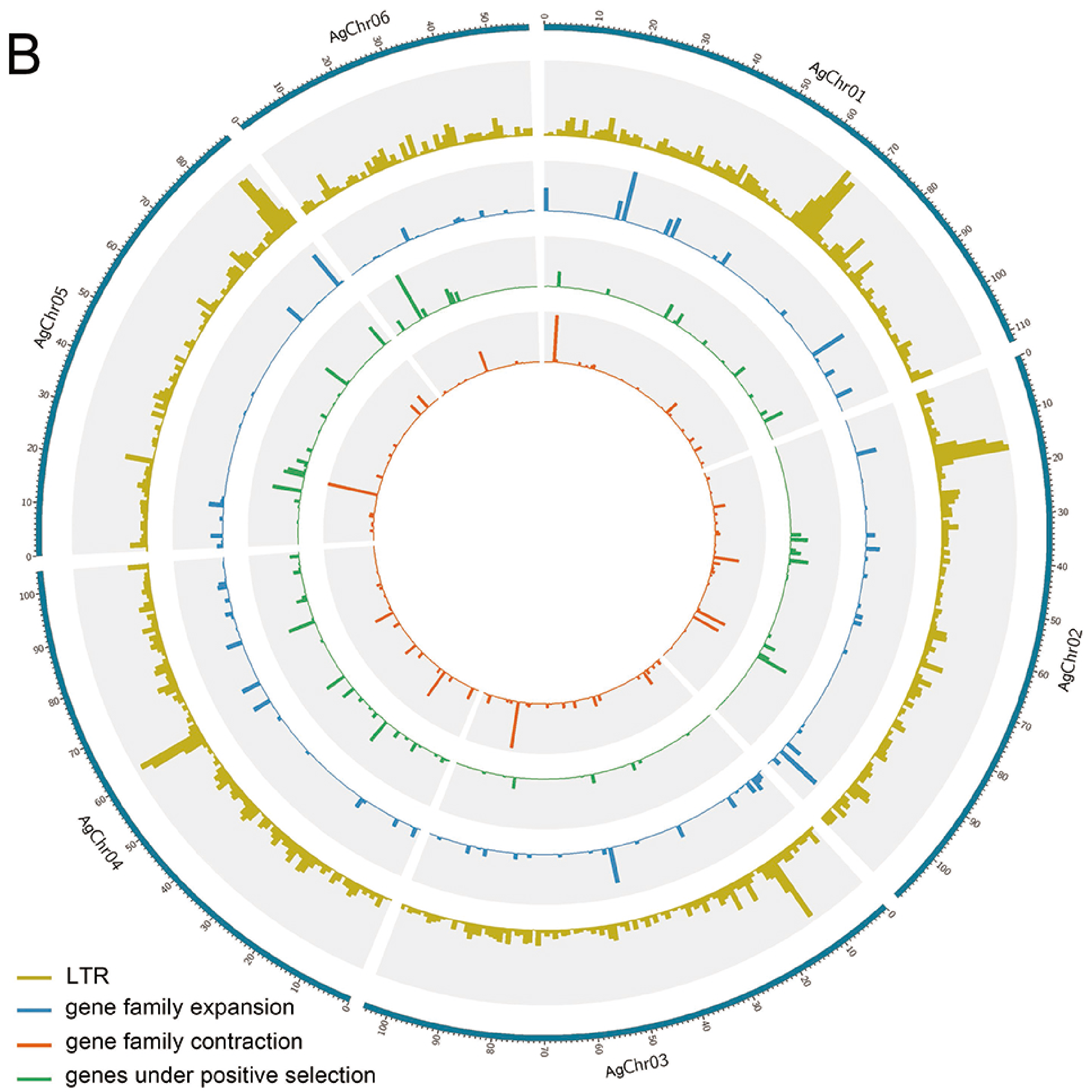

Figure S1

Click here to access/download;Figure;Figure

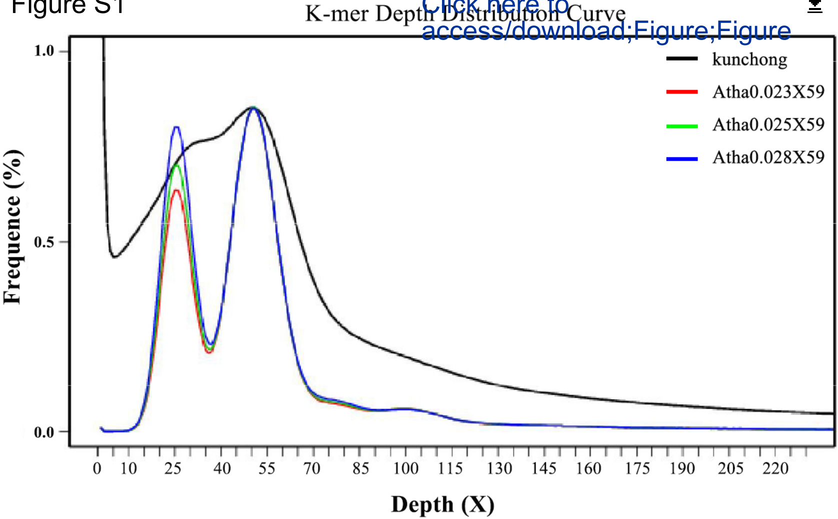

Figure S2

Click here to access/download;Figure;Figure S2.pdf

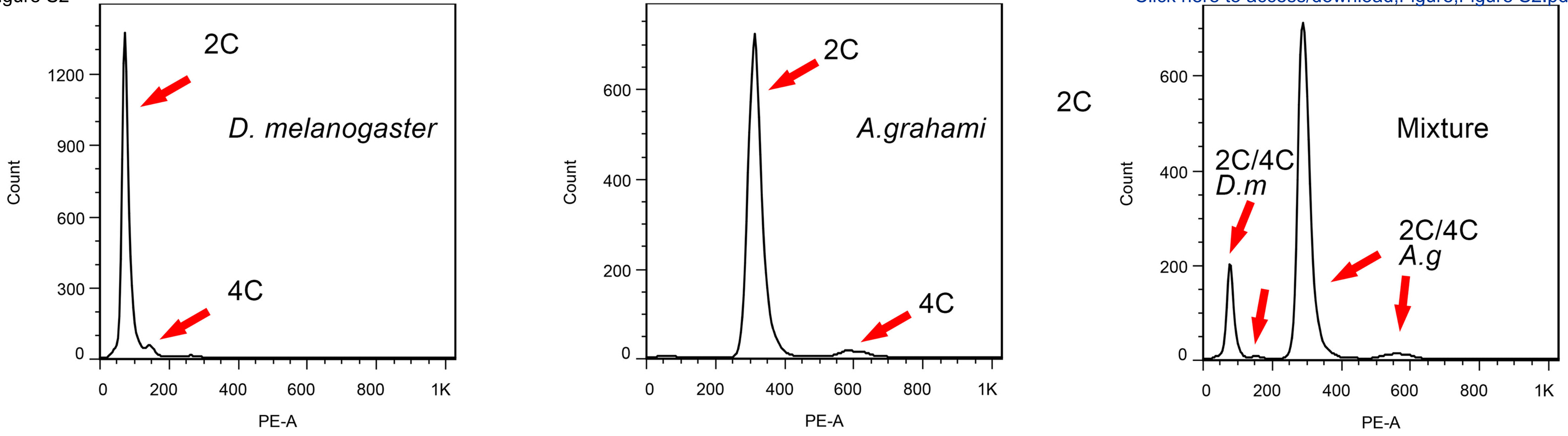

20190702_Tube_001.fcs
Cell Cycle
Dean-Jett-Fox
RMS = 26.37
Freq. G1 = 14.03
Freq. S = 49.62
Freq. G2 = 0
G1 Mean = 71.3
G2 Mean = 145
G1 cv = 2.99
G2 cv = 2.95
Freq. sub-G1 = 27.79
Freq. super-G2 = 6.6

9891

20190702_Tube_002.fcs
Cell Cycle
Watson
RMS = 15.83
Freq. G1 = 32.41
Freq. S = 33.52
Freq. G2 = 1.18
G1 Mean = 312
G2 Mean = 602
G1 cv = 2.87
G2 cv = 3
Freq. sub-G1 = 29.16
Freq. super-G2 = 2.57

9808

20190702_Tube_004.fcs
Cell Cycle
Dean-Jett-Fox
RMS = 18.41
Freq. G1 = 2.38
Freq. S = 21.57
Freq. G2 = 32.3
G1 Mean = 77
G2 Mean = 286
G1 cv = 2.93
G2 cv = 2.88
Freq. sub-G1 = 5.19
Freq. super-G2 = 36.31

9873

Figure S3

Click here to access/download;Figure;Figure S3.pdf

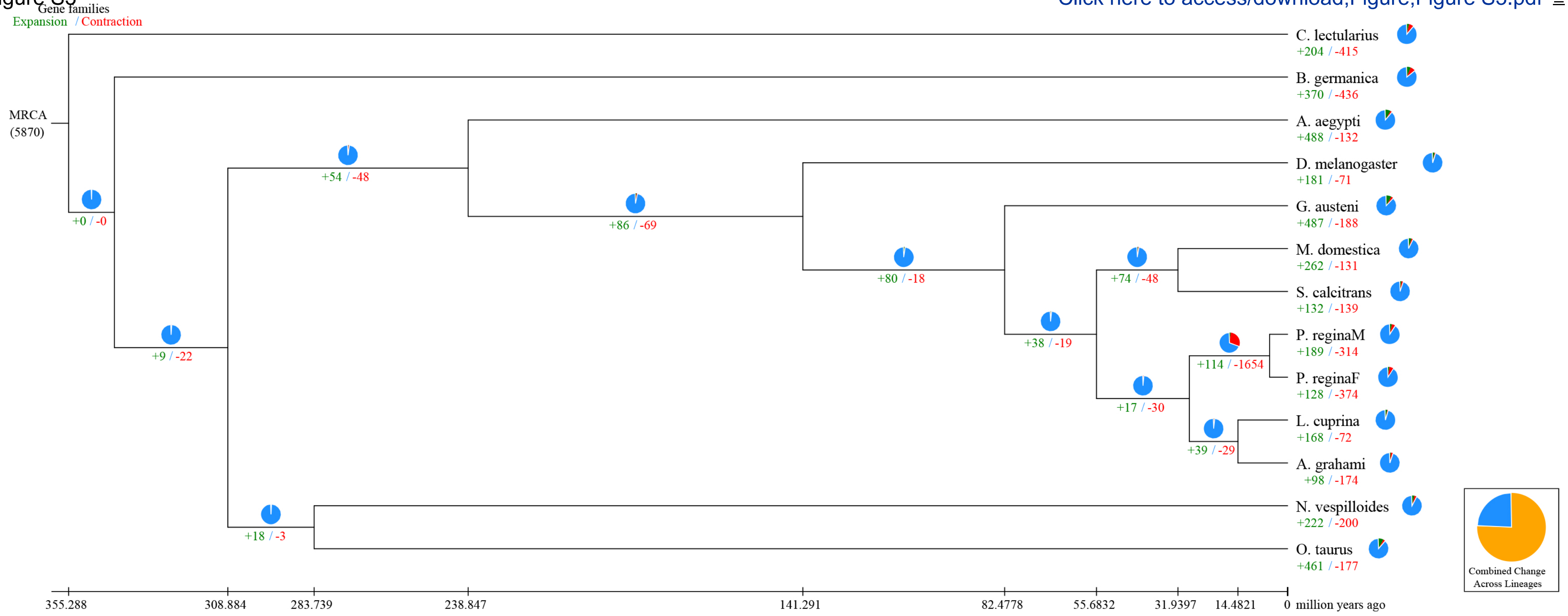

Figure S4

Click here to access/download;Figure;Figure S4.pdf

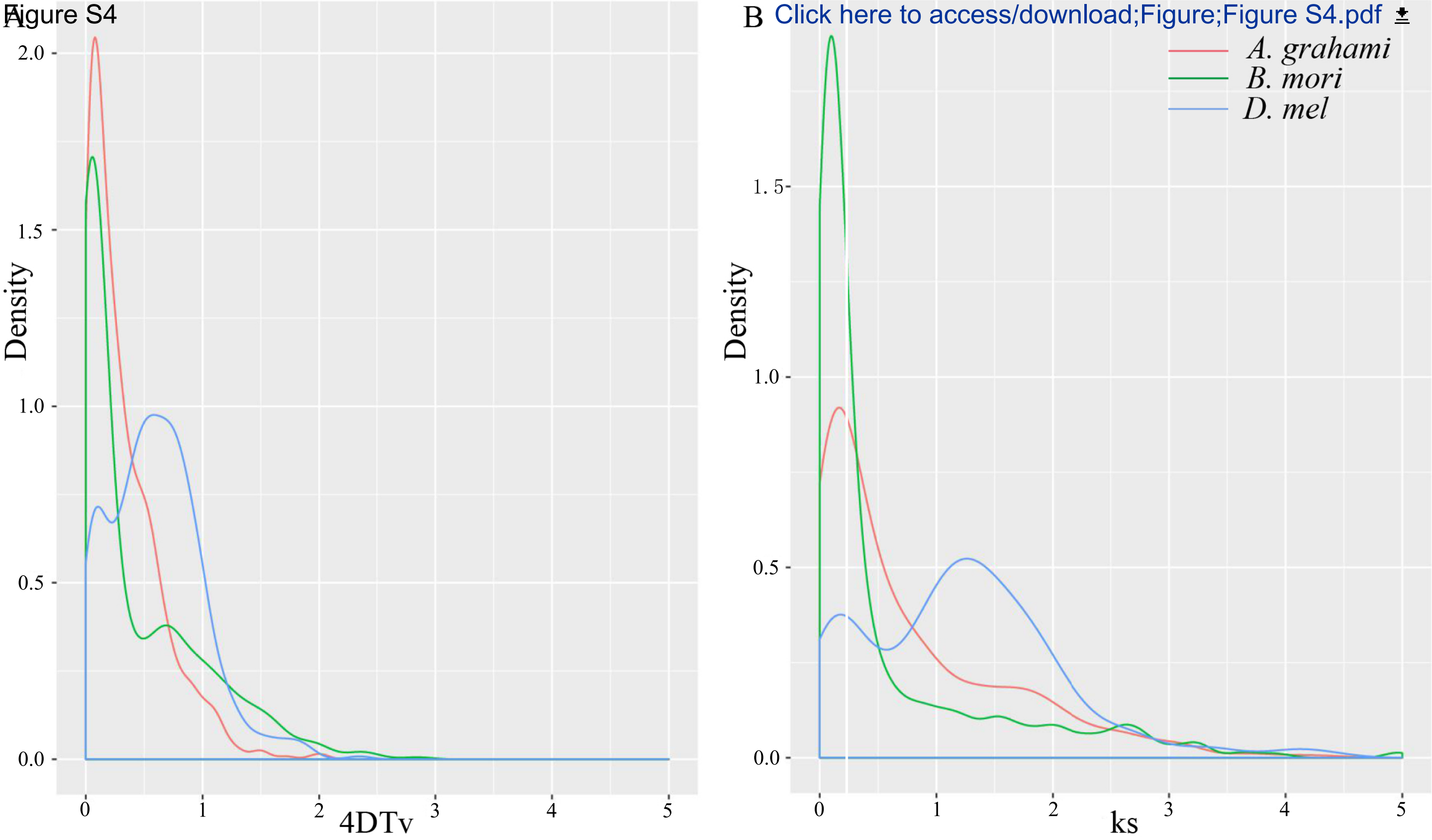

Click here to access/download
Supplementary Material
supplementary table 20200201.docx

Personal Cover Click here to access/download;Personal Cover;cover letter 20200115.docx

Dear Editor,

We are very happy to hear the letter from you and we would like to give our great appreciation to you and all the reviewers again, as well as the Data editor of GigaScience.

Here we are resubmitting the manuscript "Chromosomal-level genome assembly of *Aldrichina grahami*, a forensically important blow fly" by Meng et al. (GIGA-D-19-00066R3) in the 3rd round revision. In this version of revise, we followed all the suggestions as you listed, then made briefly response to reviewer 2. We have completed the supporting dataset information in GigaDB and cited the link in our manuscript. And the manuscript was also rechecked by native speaker again as you suggested.

Thanks again to the all reviewers and editors of GigaScience for patiently revised our previous submission.

Sincerely yours!

Jifeng Cai
