# Supplementary material for: Chromosome-level genome assembly of Aldrichina grahami, a forensically important blowfly
Source: Gigascience. 2020 Mar 19;9(3):giaa020. doi: 10.1093/gigascience/giaa020 (PMC7081965; doi:10.1093/gigascience/giaa020)
Supplement: giaa020_Supplemental_Table [file giaa020_supplemental_table.docx]

**Table S1. Information of sequencing platform and output data**

| **Platform** | **Library type** | **Library number** | **Library size (bp)** | **Data size (Gb)** |
| --- | --- | --- | --- | --- |
| Illumina Hiseq X10 | Short insert size |  | 400 | 46.05 |
| PacBio Sequel | Long insert size | 7 | 20000 | 50.15 |
| Illumina NovaSeq 6000 | Hi-C | 2 | 150 | 74.24 |

**Table S2. Genome size estimation and Heterozygosity based on 17 k-mer**

| **Sample** | **k-mer** | **kmer_num** | **kmer_depth** | **Genome size (Mb)** | | **Heterozygosity (%)** |
| --- | --- | --- | --- | --- | --- | --- |
| *A. grahami* | 17 | 29,131,491,603 | 50 | 582.63 | 2.5 | |

**Table S3. Statistics of genome assembly**

| **Stat Type** | **Contig Length** | **Contig Number** | **Scaffold Length** | **Scaffold Number*** |
| --- | --- | --- | --- | --- |
| N50 | 1,925,180 | 79 | 104,650,035 | 3 |
| N60 | 1,455,152 | 114 | 104,176,436 | 4 |
| N70 | 948,798 | 165 | 104,176,436 | 4 |
| N80 | 616,470 | 242 | / | / |
| N90 | 274,886 | 387 | / | / |
| Longest | 12,234,297 | 1 | 112,158,196 | 1 |
| Total | 600,090,062 | 1,604 | 600,249,762 | 7 |
| Length>1kb | 600,090,062 | 1,604 | 600,249,762 | 7 |
| Length>2kb | 600,090,062 | 1,604 | 600,249,762 | 7 |
| Length>5kb | 600,062,286 | 1,598 | 600,249,762 | 7 |

*The assembly based on results of Hi-C sequencing was also adds in this table to show the statistics of scaffolds.

**Table S4. Assessment on assembly completeness**

| **Type** | **Number** | **Percent (%)** |
| --- | --- | --- |
| Complete BUSCOs (C) | 1,645 | 99.2 |
| Complete and single-copy BUSCOs (S) | 1,633 | 98.5 |
| Complete and duplicated BUSCOs (D) | 12 | 0.7 |
| Fragmented BUSCOs (F) | 5 | 0.3 |
| Missing BUSCOs (M) | 8 | 0.5 |
| Total BUSCO groups searched | 1,658 | 100 |

**Table S5. Genome resource of 10 insect species for comparable genomics analysis.**

| **Species list** | **de novo data link** |
| --- | --- |
| *Drosophila melanogaster* | <https://www.ncbi.nlm.nih.gov/genome/47> |
| *Lucilia cuprina* | <https://www.ncbi.nlm.nih.gov/genome/12732> |
| *Musca domestica* | <https://www.ncbi.nlm.nih.gov/genome/14461> |
| *Stomoxys calcitrans* | <https://www.ncbi.nlm.nih.gov/genome/11278> |
| *Aedes aegypti* | https://www.ncbi.nlm.nih.gov/genome/44 |
| *Blattella germanica* | https://www.ncbi.nlm.nih.gov/genome/13223 |
| *Cimex lectularius* | https://www.ncbi.nlm.nih.gov/genome/11279 |
| *Glossina austeni* | https://www.ncbi.nlm.nih.gov/genome/16689 |
| *Nicrophorus vespilloides* | https://www.ncbi.nlm.nih.gov/genome/40824 |
| *Onthophagus taurus* | https://www.ncbi.nlm.nih.gov/genome/12827 |
| *Phomia regina* | https://www.ncbi.nlm.nih.gov/genome/36631 |

**Table S6. Comparison of *A grahami* and other fly species on protein coding genes structure and statistics**

| **Species** | **Number of gene** | **Average gene length (bp)** | **Average CDS length (bp)** | **Average exons per gene** | | **Average exon length (bp)** | **Average introns per gene** | **Average intro length (bp)** |
| --- | --- | --- | --- | --- | --- | --- | --- | --- |
| *A.grahami* | 12,823 | 13,240.43 | 1,669.56 | 4.62 | 361.16 | | 3.62 | 3,193.92 |
| *D.melanogaster* | 13,918 | 4,597.06 | 1,560.59 | 3.9 | 400.29 | | 2.9 | 1,047.56 |
| *G.austeni* | 19,725 | 5,058.51 | 1,243.50 | 4.37 | 284.4 | | 3.37 | 1,131.27 |
| *L.cuprina* | 15,536 | 6,989.99 | 1,584.56 | 3.9 | 406.15 | | 2.9 | 1,863.01 |
| *M.domestica* | 14,888 | 11,464.01 | 1,570.65 | 4.11 | 381.94 | | 3.11 | 3,178.79 |
| *S.calcitrans* | 13,982 | 19,503.86 | 1,648.55 | 4.28 | 384.78 | | 3.28 | 5,436.46 |
| *P.regina* (M) | 9,490 | 3,599.69 | 1,414.99 | 3.51 | 403.27 | | 2.51 | 870.84 |
| *P.regina* (F) | 8,312 | 4,063.23 | 1,477.94 | 3.76 | 393.06 | | 2.76 | 936.66 |
| *A.aegypti* | 14,613 | 33,770.03 | 1,616.67 | 4.27 | 378.45 | | 3.27 | 9,827.39 |
| *B.germanica* | 28,676 | 16,173.79 | 932.72 | 4.42 | 211.26 | | 3.42 | 4,462.96 |
| *C.lectularius* | 11,936 | 19,718.22 | 1,577.18 | 8.03 | 196.52 | | 7.03 | 2,582.12 |
| *N.vespilloides* | 12,642 | 5,372.83 | 1,521.92 | 5.3 | 287.37 | | 4.3 | 896.4 |
| *O.taurus* | 14,537 | 7,834.32 | 1,523.39 | 4.54 | 335.67 | | 3.54 | 1,783.58 |

**Table S7. Functional annotation of non-coding RNA genes**

| **Type** | **Copy**  **Number** | **Average**  **Length (bp)** | **Total**  **Length (bp)** | **Percentage (%)**  **of Genome** |
| --- | --- | --- | --- | --- |
| rRNA | 21 | 1473.43 | 30942 | 0.002613 |
| 18S | 4 | 2443 | 9772 | 0.000825 |
| 28S | 6 | 3237.67 | 19426 | 0.00164 |
| 5.8S | 8 | 174.88 | 1399 | 0.000118 |
| 5S | 3 | 115 | 345 | 0.000029 |
| snRNA | 192 | 134.86 | 25894 | 0.002186 |
| CD-box | 72 | 124.57 | 8969 | 0.000757 |
| HACA-box | 31 | 118.23 | 3665 | 0.000309 |
| splicing | 89 | 148.99 | 13260 | 0.00112 |
| miRNA | 126 | 87.22 | 10990 | 0.000928 |
| tRNA | 859 | 76.52 | 65731 | 0.00555 |

**Table S8. Diet habit of 9 selected insect species**

| **Order** | **Species name** | **Diet habit** |
| --- | --- | --- |
| Blattaria | *B. germanica* | polyphagia |
| Hemiptera | *C. lectularius* | hematophagia |
| Coleoptera | *N. vespilloides* | necrophagia |
| Coleoptera | *O. taurus* | coprophagia |
| Diptera | *S. calcitrans* | hematophagia |
| Diptera | *A.aegypti* | hematophagia |
| Diptera | *G. austeni* | hematophagia |
| Diptera | *L. cuprina* | necrophagia, facultative parasitism |
| Diptera | *M. domestica* | necrophagia, coprophagia |
| Diptera | *P. regina* | necrophagia |

**Table S10. Statistics of the Hi-C assembly of the *A. grahami* genome**

| **Sample** | **Statistics** |
| --- | --- |
| Clean Bases(bp) | 71,001,944,196 |
| Clean Paired-end Reads | 243,128,013 |
| Unique Mapped Paired-end Reads | 101,626,567 |
| Unique Mapped Ratio (%) | 41.80 |
| Valid Paired-end Reads | 63,268,612 |
| Valid Rate (%) | 62.26 |

**Table S12. Information of voucher samples used in present paper**

| **Type of test** | **Sample type** | **Voucher Number** |
| --- | --- | --- |
| Molecular identification | adult | CUS-MI20170325-0136 |
| Genome survey  De novo sequencing | unmated female adults  unmated female adults | CSU-DS20170905-Ag(F) (01-18) |
| Transcriptom sequencing | female adults  male adults | CSU- TS20170905-Ag(F) (01-05)  CSU-TS20170905-Ag(M) (01-06) |
| Hi-C sequencing | unmated female adults | CSU-HS20171103-Ag(F) (01-10) |
| Flow cytometry | male adults | 20190702_Tube_002  20190702_Tube_003  20190702_Tube_004  20190702_Tube_005  20190702_Tube_006  20190702_Tube_007  20190702_Tube_008 |
|  | female adults | 20190702_Tube_009  20190702_Tube_010  20190702_Tube_011  20190702_Tube_012  20190702_Tube_013  20190702_Tube_014 |
|  | adult *D*. *melanogaster* | 20190702_Tube_001 |
