# Supplementary material for: Chromosome-level genome assembly of Aldrichina grahami, a forensically important blowfly
Source: Gigascience. 2020 Mar 19;9(3):giaa020. doi: 10.1093/gigascience/giaa020 (PMC7081965; doi:10.1093/gigascience/giaa020)
Supplement: giaa020_Response-to-Reviewer_Comments_Revision_1 [file giaa020_response-to-reviewer_comments_revision_1.pdf]

Author's Response To Reviewer Comments

Close

Dear Editor Zauner and reviewers,
We would like to express our great appreciation to you and reviewers for those constructive comments and suggestions on our manuscript, which provide really valuable and helpful for revising and improving our paper.
Here we are resubmitting the manuscript “Chromosomal-level genome assembly of Aldrichina grahami, a forensically important blow fly” by Meng et al. (GIGA-D-19-00066R1) in the 2ed round revision. Because it is the last chance for the potential approval of our manuscript, we have studied comments carefully and tried our best to improve the manuscript. We hope that the correction will meet with approval.
In this revised version, we would like to response the questions you highlighted in your letter as below:
1) Comparative genomic analyses: Please make another effort to get hold of the Phormia regina data. As the reviewer says, if an NCBI link is not working, you should try to contact the support team or the authors directly.
Response: As the reviewer 2 suggested, we tried our best to contact the submitter of Phormia regina genome data. And the author kindly supplied it to us. The Phormia regina genome was finally add to the analysis of corresponding parts in the new version of our manuscript. Related tables and figures were all changed correspondingly.
2) flow cytometry: Please carefully consider the latest comment of reviewer 2, Aaron Tarone, who is an expert for this subject matter.
Response: We carefully rechecked the results of this part and corrected the manuscript according to the Reviewer 2’s suggestions. We provided the species information which suggested by the reviewer, and recalculated the C-value and related results described in our manuscript. And we also added more details to illustrate our results which we think would meet the reviewer’s advices.
3) Please improve the discussion of Muller elements, see reviewer 2' advice
Response: We further studied the references and researches suggested by reviewer 2 which provide important information to understanding the issue of Muller elements.
To meet the reviewers suggestion and draw a more reliable conclusion, we tried other strategies to determine the sex chromosome and corresponding Muller element of A. grahami (please see details in response to reviewer 2). But no useful information generated by bioinformatics methods. We also tried the software and method recommended by the reviewer 2, but they required the genome data of both sexes. The genome de novo and Hi-C sequencing of A. grahami were performed on female adults only. So we would like to do this test in our next future research, which requires additional sampling and re-sequencing step for de novo sequencing procedure. Based on the above, we would like to withdraw the conclusion of ‘AgChr3 is the X-chromosome of A. grahami’ which included in our previous manuscript, due to lack of supporting information.
Though we cannot make an assumption on which chromosome should be the X chromosome, the importance of the chromosome-level assembled A. grahami genome should facilitate to answer this question in the future research. Since present A. grahami genome is the only published chromosome level genome resource of blow fly with forensic importance, it also provide an important genome data to perform a reliable collinearity analysis between A. grahami and other sibling species of the calyptratae. A discussion on this issue was added in the new manuscript also.
4) Language: I would recommend to recruit help from a native speaker who is familiar with the biological context, to correct mistakes and unclear expressions in your revised paper before you resubmit to us.
Response: The new submitted version of our manuscript was carefully revised by native speaker. And we can provide the Certificate of English Proofreading. Changes were marked all in RED.
Thanks again to the reviewers and editors of GigaScience for patiently revised our previous submission and giving us the opportunity to make improvement. We are looking forward to hearing from you.
Sincerely yours!

Reviewer reports:

Reviewer #1: This R1 manuscript describes the sequencing, assembly and analysis of the genome of

Aldrichina grahami, a forensically important blow fly. The authors used long-read PacBio sequencing and Hi-C scaffolding technology to produce chromosome-level scaffolds. Considering its forensic importance, the draft genome of A. grahami will be a valuable resource in comparative genomics analyses of other necrophagous dipterans, and arthropods more broadly.
Thank you to the authors for addressing each point in the reviewer's reports, and taking the time to perform the suggested changes/analyses. I believe the resulting R1 manuscript is of a much higher quality.
However, herewith is a list of some of the issues that still need to be addressed.
Response 1: We really appreciate your positive comment. And your suggestions give great help for us to make further improvement of our manuscript. All the correction and revision was list as below.

Reviewer #1: Line 25/26: Clarify/rephrase introductory sentence as it is still not clear
Response: Thanks for your patience on our expression. We change it as below (Line 25).
Blow flies (Diptera: Calliphoridae) are the most commonly found entomological evidence in the forensic investigation.

Reviewer #1: Line 43: Clarify "Nearly 96.4% of these scaffolds was anchored to A. grahami genome" as it is still not clear. Is there a different way to describe this?
Response: Thank you for your suggestion. We changed it to "Using Hi-C sequencing, a chromosomal-level assembly of 6 chromosomes was generated with scaffold N50 of 104.7 Mb. Of these scaffolds, nearly 96.4% were anchored to the total A. grahami genome contig bases." (Line:43)

Reviewer #1: Line 52: Clarify "medical legal investigation" and how arthropods are used
Response: Thanks for the reviewer's suggestion. The glossary "medical legal" was inappropriate. We changed it to "medicolegal" (Line 53). We tried to answer the reviewer's the question simply as below.
The main test of forensic entomology was to deals with arthropod involved in crimes such as murder, suicide, rape and physical abuse [1]. It was also described as medicolegal or medicocriminal forensic entomology [2].
Several areas of forensic entomology have progressed considerably in the past few decades. We use the estimation on the PMI, which represents the main tasks of forensic investigation, to make an example of how entomological evidence be used in medicolegal investigation. One can use entomological data to estimate the PMI in two basic ways. First, insects are usually the first organisms to find and locate corpse. Often blow flies, could oviposit on carrion within the first few hours or even minutes after death if given access [3]. So the determination of the age of developing maggots on corpse, which just like a biological clock, is the basis for the PMI estimation. During the earlier stage of decomposition, the estimation could be based on the time length needed for particular species to develop, which collected at the death scene [4].
Second, corpse is a temporary resource in an opening environment. It could be exploited by a wide diversity of organisms ranging from microbes to vertebrate scavengers. Arthropods usually constitute the major fauna on the corpse, and insects predominate as the most constant, diverse, and conspicuous group under most situation. The arthropod community in and around a corpse changes by a rather predictable succession pattern as the decomposition going on. PMI estimation is based on the change of composition of the arthropod community for it relates to such expected successional patters [5]. This second basic approach to determining the PMI is referred to those corpses in advanced stages of decomposition.
For other arthropods groups, like mites, a number of species are parasitic on humans. Their survival on the body after death can be used to calculate the PMI. The scabies mite (Sarcoptes) can survive for a few days after the death of its host but not more than 2 weeks post-mortem [6]. Demodex is a common inhabitant of human face hair follicles and can be found alive 55 h after the death of their host [7-8]. Acarines are frequently seasonal so that their presence can effectively provide 'date stamp' evidence.
Crustacean (cribs or shrimps) were usually found on corpse in marine situation with other invertebrate species. But no classic succession was observed in contrast with insect colonization in terrestrial environments. Most invertebrate fauna were opportunistic scavengers. The fauna on the remains, the decomposition and the feeding patterns are valuable in identifying the environment in which the body has decomposed and identifying the origin of marks (feeding) on the remains [9].

Reference
[1] Smith, K. G. V. 1986. A manual of forensic entomology. London: British Museum (Natural History), Comstock. 205 pp.
[2] Hall, R. D. 1991 . Medicocriminal entomology. See Ref. 16, pp. 1-8
[3] Catts E. P. Problems in estimating the postmortem interval in death investigations l[J]. Journal of Agricultural Entomology, 1992, 9(4):245-255.

[4] Catts E P , Goff M L . Forensic entomology in criminal investigations [J]. Annual Review of Entomology, 1992, 37(1):253-272.
[5] Goff, M. L., Flynn, M. M. 1991. Determination of postmortem interval by arthropod succession: a case study from the Hawaiian Islands. Forensic Sci. 36:607-14
[6] Mellanby K (1943) Scabies. Oxford University Press, Oxford
[7] Ozdemir MH, Aksoy U, Akisu C et al (2003) Investigating Demodex in forensic autopsy cases. Forensic Sci Int 135:226–231. doi:10.1016/S0379-0738(03)00216-0
[8] Ozdemir MH, Aksoy U, Sonmez E et al (2005) Prevalence of Demodex in health personnel working in autopsy room. Am J Forensic Med Pathol 26:18–23.
[9] Amendt J, Goff M L, Campobasso C P, et al. Current concepts in forensic entomology[M]. 2010. p267-268.

Reviewer #1: Table 2: Suggest clarifying the description of "and etc"
Response: Thank you for suggestion. Other is sequences with annotation but not belonging to the above sorts of repetitive genes, such as satellites, simple repeats, retroposon, artefact, helitron and low complexity repeats. Changed in the manuscript also.(Line 246)

Reviewer #1: Line 393: Why 'et al.'?
Response: Sorry for this ambiguous writing. All analysis included in this part were distributions of long terminal repeat (LTR), gene family expansion or contraction, and genes under positive selection. It has been changed. (Line 405)

Reviewer #1: Line 416-418: Please clarify closing sentence.
Response: Thank you for your suggestion on this point. We revised the closing sentence to make it more clearly and readable (Line 430), and try to explain the sentence to the reviewer.
Based on qualified genome resource, studies of forensically important blow fly species will reinforce the reliability of entomological evidence and promote its application in the legal criminal investigation.
A crucial test for some forensic cases is the identification of geographic distribution of insect samples. This identification largely depends on the existence of genetic data in different regions, enough genetic differentiation between regions and the quality of the analytical method. Some promising findings exist, such as the application of different genetic markers. And phylogenetic analyses have successfully been admitted as evidence in legal criminal cases [1]. But it is suggested that we are still far from a foundational validation of this approach to be used in legal cases. Limitations come from different aspects like the insufficiency of reference databases, lacking of reference genome sequences, metadata, and representative genetic diversity coverage [2]. We think a high-quality genome resource will provide a valuable reference genome for a particular insect group. And the following population studies will explore additional genetic markers that can characterize its geographic distributions. The genetic analyses based on large datasets (such as whole genomes) will definitely provide high statistical confidence which can be useful in legal criminal cases [3].
Reference
[1]Bhattacharya S. Science in court: Disease detectives. Nature. 2014; 506(7489):424–6. https://doi.org/10.1038/506424a PMID: 24572408.
[2]Sjodin A, Broman T, Melefors O, Andersson G, Rasmusson B, Knutsson R, et al. The need for highquality whole-genome sequence databases in microbial forensics. Biosecur Bioterror. 2013; 11 Suppl1:S78–86. https://doi.org/10.1089/bsp.2013.0007 PMID: 23971825.
[3]Jager AC, Alvarez ML, Davis CP, Guzman E, Han Y, Way L, et al. Developmental validation of the MiSeq FGx forensic genomics system for targeted next generation sequencing in forensic DNA casework and database laboratories. Forensic science international Genetics. 706 2017; 28: 52-70. doi:10.1016/j.fsigen.2017.01.011.

Reviewer #2: The authors did a good job of beginning to address the issues raised in the previous reviews. However, there are some areas of concern to address with the new round.

1. The authors should make more of an attempt to either standardize or justify specific comparative genomic comparisons they are making. The excuse for not including Phormia regina in all analyses seems wrong. If NCBI has a faulty link, authors should work with NCBI to fix it and gain access to the genome. Also, did the manuscript authors try contacting the authors of the Phormia regina genome paper to see if they can provide?
Response: We greatly appreciate your suggestion and recommendation on this point. As you suggested, we tried our best to contact the submitter of Phormia regina genome. The submitter kindly provided the genome data of Phormia regina to us. So we add the Phormia regina genome to all the comparative genomic analysis steps, including homologous gene family, phylogenetic analysis, evolutionary history,

and genes gain and lose, to gain a more complete and reliable result. The results were added to relative parts in the new version of manuscript respectively (line 349). And changes were also made in figures and tables accordingly (Figure 2, 4, S2, S3, Table 4, S5, S6, S9, S11). Thanks again for your suggestion and we thought this supplementary work could make an improvement to our study.

2. The authors are to be commended for their reevaluation of genome size based on flow cytometry. However, a few items are concerning in their description and implementation of the work. (1) First, in supplemental data the authors clarify they used Drosophila melanogaster as the reference genome, but they do not report the genus or species name in the main text. Since D. virilis is a common genome size reference, the authors should clarify which species (and strain) they analyzed to identify genome size. It would also help to see the genome size the authors are assuming (175 Mb for D. melanogaster?). (2) The CV values for the histograms appear to be unacceptably large (I believe <3 is considered good practice). (3) Finally, when one looks at the genome size histograms there appears to be a linearity issues with the analysis. The peaks for 2C/4C do not appear with 4C as 2x of 2C. This can be a problem with some cytometers, which do not all report fluorescence in a linear manner. This could indicate a biased genome size estimate.
Response: Thanks a lot for your suggestion on this part of work. It provided an opportunity for us to supplement more information which related to the results. We supplemented the species information of which we used as reference genome. And according to you suggestion, we rechecked the result part of flow cytometry test (Line 214-222). And we would like to describe it in details as below.
The fruit fly species we used in flow cytometry test was Drosophila melanogaster (strain w118), which C-value was assumed as 0.18 pg (175Mb). We add this information into the new version of manuscript. For the genome size histograms, we rechecked the results in Flow Jo software and output the data with CV value <3. And accordingly, we recalculated the C-value of A.grahami based on the above C-value of Drosophila melanogaster and results of flow cytometry. Changes can be found in Line 218. For the question of linearity issue, on the histograms of Drosophila melanogaster, the 4C (mean=138) peak was actually next to the main 2C peak (mean=71) and overlap was existed between these two peaks. The 4C peak is relative lower than 2C but do exist (also see Michael D. et al Annals of Botany, 2003). We marked the 2C/4C peak on the histogram of the supplementary figure S2. And it could be clearly recognized by the cell cycle analyses in Flow Jo.

3. Muller element analyses are good, but authors need to delve into the literature on this more. Muller F is sometimes a sex chromosome. Others can be associated with sex in other species too. See work by Doris Bachtrog, Rich Meisel, and Max Scott for higher flies (including blow flies and house/stable flies). In addition, coverage analysis might help authors determine elements that differ in coverage between the sexes. It is not sufficient to imply a sex chromosome because it is the sex chromosome in Drosophila melanogaster.
Response: Great thanks to you for your advices and suggested reference work on this issue. After studying those researches closely, we agree that we cannot draw a conclusion that AgChr03 is the sex chromosome of A. grahami according to current limit information, although it is corresponding to the ChrX in D.melanogaster in the collinearity analysis. Based on previous research work as you suggested and other related studies of evolution of sex chromosome and Muller elements in the diptera, orthologs of genes located on the fourth chromosome in D. melanogaster (Muller F) are X-linked in some non-drosophilids or Calyptratae [1-2]. It appears that the Muller element F was an ancestral X chromosome in Diptera [1,3]. Based on the Muller element mapping of the D. melanogaster orthologs, Rich Meisel assigned house fly genes that are conserved as one-to-one orthologs with D. melanogaster to house fly chromosomes [4]. The situation in Max Scott's study of Lucilia cuprina appears that the Muller F of D.melanogaster can be highly mapped to the ChrX of the former [5]. However, the collinearity of chromosome between D. melanogaster and A. grahami exhibited Chr4 of D.melanogaster has no collinear position on A. grahami chromosome in present work. Since the assembled chromosome of A. grahami in present paper is the only published resource in calyptratae so far, we cannot perform the same analysis between A. grahami and other sibling species. And limitation of the software MCSCAN in performing collinearity analysis made it hard to draw a definite conclusion by a single result [6].
To get more information, we searched for other strategies to infer the sex chromosome of A. grahami. First, we tried to use the contigs of P. regina's ChrX to align with A. grahami genome assemblage. So we downloaded the predicted contigs list of ChrX of P. regina described in J. Picard's study (2016) [8]. But only 143 contigs sequences could be recognized from the genome resource of P. regina which deposited on NCBI. And merely 2 contigs could be aligned to chromosomes of A. grahami. Second, we download the ChrX specific genes of L. cuprina documented in study of Maxwell J. Scott (2018) [5], and blasted against the A. grahami genome. But no useful information generated either. We further downloaded the software CQ-calculate, which has been used in determine X/Y chromosome of the Phomia regina [7]. But the software needs both the male and female genome resource to perform the prediction according to its

instruction [8]. And the coverage analysis which recommended by the reviewer 2 also requires the genome data of two sexes. The genome de novo and Hi-C sequencing of A. grahami were performed on female adults only. Therefore, current data do not yield convincing information on sex chromosomes. However, this is an interesting question for future research, which involves additional samples and resequencing.,
Based on the above, we would like to withdraw the conclusion of AgChr03 is the sex chromosome of A. grahami. And since the Muller F of A. grahami is not distinguishable in present work, we cannot make an assumption on which chromosome should be the X chromosome either. We add a brief discussion in the new version of manuscript (Line 400).

[1] Linger, Rebecca J. , et al. "Dosage compensation of X-linked muller element F genes but not X-linked transgenes in the Australian sheep blowfly." PLOS ONE 10.10(2015):e0141544-.
[2] Landeen, Emily & Presgraves, Daven. (2013). Evolution: From autosomes to sex chromosomes - and back. Current biology : CB. 23. R848-50. 10.1016/j.cub.2013.08.021.
[3] Vicoso B, Bachtrog D. Reversal of an ancient sex chromosome to an autosome in Drosophila. Nature.2013; 499(7458):332–5. doi: 10.1038/nature12235 PMID: 23792562; PubMed Central PMCID:PMC4120283.
[4] Richard P. Meisel, Jeffrey G. Scott, Andrew G. Clark, Transcriptome differences between alternative sex determining genotypes in the house fly, Musca domestica, Genome Biology and Evolution, Volume 7, Issue 7, July 2015, Pages 2051–2061,
[5] Davis R J , Belikoff E J , Scholl E H , et al. no blokes, Is Essential for male viability and X chromosome gene expression in the Australian sheep blowfly[J]. Current Biology, 2018:S0960982218306079-.
[6]Wang Y , Tang H , Debarry J D , et al. MCScanX: a toolkit for detection and evolutionary analysis of gene synteny and collinearity[J]. Nucleic Acids Research, 2012, 40(7):e49-e49.
[7] Andere A A , Platt R N , Ray D A , et al. Genome sequence of Phormia regina Meigen (Diptera: Calliphoridae): implications for medical, veterinary and forensic research[J]. BMC Genomics, 2016, 17(1):842.
[8] Hall AB, Qi Y, Timoshevskiy V, Sharakhova MV, Sharakhov IV, Tu Z. Six novel Y chromosome genes in Anopheles mosquitoes discovered by independently sequencing males and females. BMC Genomics. 2013;14:273. Published 2013 Apr 23. doi:10.1186/1471-2164-14-273

Minor

Q:Uningnorable is a difficult word to read through. Maybe replace with "a detectable and potentially important"
Response: Corrected (Line 97).

Q:Report COI sequence to a database (Nucleotide database on NCBI?)
Response: We have reported the COI sequence to the Nucleotide database on NCBI, and got an accession number of MN537823 (Line 117).

Q:Provide the specific voucher number for the specimens submitted as vouchers to the museum
Response: We provided all the information of voucher number into a supplementary table (Table S12) which corresponds to the voucher samples restored in the forensic insect herbarium of the Department of Forensic Science.

Q:Why were wings/gut used to provide DNA? Or was DNA isolated without wings and gut?
Response: Wings and gut were not used in DNA extraction. They were removed from the adult body firstly. The remaining parts were used for DNA extraction. The sentence has been rephrased (Line 131).

Q:Why do authors sometimes italicize necrophagia/parasitism/hematophagia?
Response: Sorry for these incorrect typing. It has been corrected (Line 294).

Q: More justification for parameter settings on divergence time analyses would help.
Response: Thank you for your suggestion. As you suggested, we revised this part of description in a detailed way to make it more readable. Changes can be found in Line 338-347 of the new manuscript. The PAML mcmctree program (v4.5, RRID: SCR 014932) was used to determine divergence times with the calculation of approximate likelihood test, molecular clock and substitution model of REV [90]. The primary parameters of mcmctree were set as clock = 2 (independent rates model following a log-normal distribution), RootAge = < 4 (400 Myr for a calibration to the root of the phylogenetic tree) , model = 7 (the substitution model, REV), BDparas = 110 (default value is 110, parameters controlling the birth-

death process), kappa_gamma = 62 (transition/transversion rate ratio), alpha_gamma = 11 (gamma shape parameter for variable rates among sites), rgene_gamma = 23.606 (Dirichlet-gamma prior for the mean substitution rate), sigma2_gamma = 11.03 (Dirichlet-gamma prior for the rate drift parameter). (Line 338)

Q: On line 146 - which fragments were proper size?
Response: Sorry for the inadequate information. It should be descripted as ‘the fragments with length larger than 15Kb were selected by the Blue Pippin device’. We corrected it in the manuscript (Line 150).

Close
