# Supplementary material for: Chromosome-level genome assembly of Aldrichina grahami, a forensically important blowfly
Source: Gigascience. 2020 Mar 19;9(3):giaa020. doi: 10.1093/gigascience/giaa020 (PMC7081965; doi:10.1093/gigascience/giaa020)
Supplement: giaa020_Response-to-Reviewer_Comments_Revision_2 [file giaa020_response-to-reviewer_comments_revision_2.pdf]

Author's Response To Reviewer Comments

Close

Dear Editor,
We are happy to hear the letter from you and we would like to give our great appreciation to you and all the reviewers again, as well as the Data editor of GigaScience. In the version of revise, we followed the suggestions as you listed, then made briefly response to reviewer 2. Please check it as below.

Your suggestions
- Please have a look at the reviewers' final comments (see below) and consider briefly addressing them in the discussion section of your paper.
A: we made some briefly description and revision as the reviewer 2 suggested, and accepted the reviewer's advises for our future research

- Please carefully revise your manuscript for English language use and grammar, preferably with the help of a native speaker.
A: We invited native speaker who have experience of research article writing to polish the final version of our manuscript.

- Please mention the NCBI taxon ID for the species in the methods section (I believe it is NCBI:txid252811 , but please double check).
A: The NCBI taxon ID of Aldrichina grahami has been rechecked and confirmed as NCBI:txid252811. And it can be located by the following link (https://www.ncbi.nlm.nih.gov/Taxonomy/Browser/wwwtax.cgi?id=252811)

- At this point, you can remove any highlighting in red that was made for the purpose of review.
A: Changed as you suggested.

- Our data curators will contact you shortly to prepare the supporting dataset which will be hosted via our database GigaDB. Prior to publication, please include a citation to your upcoming GigaDB dataset (including the DOI link) to your reference list, and cite this in the data availability section and elsewhere in the manuscript, where appropriate.
A: We have completed the information of dataset on GigaDB following the guide of data curator, and cited our data linke of DigaDB as reference in the final version of our manuscript.

Reviewer reports:
Reviewer #2: The authors have done an admirable job of addressing reviews. They have justified their cytometric estimates of genome size in much more detail and have addressed my questions about their claims regarding sex chromosomes.

Small comments for consideration:
1. I believe the genome size of D. melanogaster of 175 Mb is linked to the originally sequenced strain. There may be subtle differences between it and W1118. For example, see Ellis et al. 2014 in PLoS Genetics for variation in the DGRP strains of Drosophila melanogaster. Surely the authors are close enough to the true value for the purposes of this paper, but it is worth remembering. For future reference, given the size of this genome, D. virilis may have been a better choice as it is closer to the size of what is of interest to the authors. Not necessary for this paper, but something to consider for future projects if authors continue to do this sort of work.

A: Thanks a lot for your further advice on this detail. After revised this manuscript, we do plan to explore the genome size of several forensic related fly species both by genome survey and cytometric. For the reference genome size, we would like to use blood of domestic chicken, D.melanogaster (w118), and D. virilis separately, and make a comparison between them. Although it is not our major research area, we would like to gain some knowledge of genome size of this insect group. And finally thanks again for all this valuable information you provided to us. That's really helpful.

2. The fonts in some of the figures are low resolution and hard to read.
A: All the figures used in the last version of manuscript have been replaced with large enough resolution for readable.

Close
