# Supplementary material for: Chromosome-level genome assembly of Aldrichina grahami, a forensically important blowfly
Source: Gigascience. 2020 Mar 19;9(3):giaa020. doi: 10.1093/gigascience/giaa020 (PMC7081965; doi:10.1093/gigascience/giaa020)
Supplement: giaa020_Response-to_Reviewer_Comments_Original_Submission [file giaa020_response-to_reviewer_comments_original_submission.pdf]

Author's Response To Reviewer Comments

Close

Dear Editor Zauner and reviewers,
We would like to express our great appreciation to you and reviewers for constructive comments and suggestions on our manuscript. Those are really valuable and helpful for revising and improving our paper.
Here we are submitting the revised version of the manuscript “Chromosomal-level genome assembly of Aldrichina grahami, a forensically important blow fly” by Meng et al. (GIGA-D-19-00066). We have studied those comments carefully and tried our best to improve the manuscript. We hope that the correction will meet with approval.
In this revised version, we have provided additional test and analyses to corresponding parts as reviewers suggested. We also have polished the writing and had native English speaker to review the manuscript. Changes were marked all in RED.
We integrated some additional analyses into the result of chromosome assembly after Hi-C sequencing as the reviewer suggested. For a more logical structure of the manuscript, we moved the section of “Chromosome assembly using Hi-C data” into the front of the “conclusion” section. The order of figures and tables were changed correspondingly. At last, we made a point-to-point response to the reviewers’ comments.
Thanks again to the reviewers and editors of GigaScience for patiently revised our previous submission and giving us the opportunity to make improvement. We are looking forward to hearing from you.
Sincerely yours!

Jifeng Cai, on behalf of all authors
Forensic Science department, School of Basic Medicine, Central South University.
Changsha, Hunan Province, China
Email: cjf_jifeng@163.com

Reviewer #1:
Abstract:
Line 21: Clarify/rephrase introductory sentence
RESPONSE: We are very sorry for the ambiguous expression. The sentence has been rephrased. (Line 25)

Line 25: Change "provides" to "provide" - verb tense error
RESPONSE: It has been changed. (Line 29)

Line 26: Please elaborate on "forensic issues"
RESPONSE: Great thanks to the reviewer’s advice. We rethought this sentence and found it may inappropriate to state the value of A. grahami on ‘other forensic issues’ based on limited reports and studies about this species so far. The most important meaning of fly developmental rate, pattern and life cycle is to provide information for minPIM estimation. We deleted the statement on ‘other forensic issues.’ (Line 30)

Line 28 & 94: "Pacific Biosciences" is mis-spelled
RESPONSE: It has been corrected. (Line 32, 101,142)

Line 29: Change "Totally" to "A total of"
RESPONSE: Changed. (Line 33)

Line 30: Elaborate. FALCON and Wtdbg are genome sequence assemblers for PacBio long reads
RESPONSE: Many thanks for the reviewer’s suggestion. We have revised this sentence, and elaborated them in the section of “Genome survey and Genome assembly ”. (Line 34, 181)
Reference
Eid J, Fehr A, Gray J, Luong K, Lyle J, Otto G, et al. Real-time DNA sequencing from single polymerase molecules. Science. 2009; 323 5910: 133-8. doi:10.1126/science.1162986.

Line 30: Change "was" to "were" - verb tense error
RESPONSE: Changed. (Line 35)

Line 34 & 190: Clarify "Nearly 96.4% of these scaffolds was anchored to A. grahami genome"
RESPONSE: Thank you for your suggestion. We have revised this sentence both in the Abstract and the section “Chromosome assembly using Hi-C data”. (Line 43, 376)

Line 36: Clarify what "de novo and transcriptome" data was used for genome annotation. Are these data deposited and available online?
RESPONSE: We are sorry for didn’t provide these information in the previous version. In fact, all the data of de novo genome and transcriptome of A. grahimi have been deposited into NCBI SRA database. It is available and can be located by accession number of PRJNA513084 (de novo genome) and SRX5207346 (transcriptome) now. We have described them in the new manuscript. (Line 39)

Line 40: Change "add" to "adds", and "facilitate" to "facilitates" - verb tense errors
RESPONSE: Changed as suggested. Thank you so much for your patient revision. (Line 45)

Line 41: "Necrophgous" is mis-spelled
RESPONSE: Changed. (Line 48)

Data Description:
Line 47: Clarify/rephrase sentence
RESPONSE: Rephrased. (Line 52)

Line 52: Is "it" A. grahami or the minPMI - please clarify
RESPONSE: We are sorry for this ambiguous phrase. We have rephrased the sentence to avoid misunderstanding. (Line 55)

Line 57: Clarify/rephrase sentence
RESPONSE: Rephrased. (Line 61)

Line 57: When referring to "life history" do you mean life cycle?
RESPONSE: As the reviewer pointed out, “life cycle” is more appropriate than “life history” to express the meaning. Thank you. (Line 62)

Figure 1: Remove "remain" from figure legend
RESPONSE: Changed. (Line 66)

Line 72: Combine both sentences, or rephrase
RESPONSE: Rephrased. (Line 76)

Line 72: When referring to "whole life history" do you mean whole life cycle?
RESPONSE: As the reviewer mentioned above, it should be “life cycle”. (Line 76)

Line 74: When referring to "season of death" do you mean reason of death?
RESPONSE: Thank you for your kind reminding. We previously used “season of death” to describe that A. grahami could be utilized to infer the death event happened in which season. This information could narrow the time range of investigation. To avoid misunderstanding, we rephrased this sentence. (Line 79)

Line 76: Clarify and elaborate why these studies indicate great potential application value
RESPONSE: Thank you very much for your advice. We have clarified and elaborated on this information here, and added brief description in the new version of our manuscript. (Line 80)
Many efforts had been given to identify human DNA material from the gut of larvae which feed on the corpse. The gut of larvae could be good ‘container’ for human DNA material to some range. The human DNA containing in larvae gut can be used to identify a missing body or prove the presumed association with the entomological evidence of a corpse (interpreting evidence used for forensic investigation). For instance, the corpse was removed and only maggots were discovered in the scene. And sometimes insect evidences from one crime scene was divided and sent to different investigators.
Cuticular hydrocarbons, including more than 100 compounds, are the major components of insect cuticle. Previous studies have demonstrated that the composition of cuticular hydrocarbons vary by

maturity or age. And this variation had been analyzed in some forensic important species of fly larvae and used for PMI estimation. The cuticular hydrocarbons of larvae of A. grahami were also reported as a potential indicator to its development and had the potential value in the forensic investigation on PMI.

Reference

Zehner R, Amendt J and Krettek R. STR typing of human DNA from fly larvae fed on decomposing bodies. Journal of forensic sciences. 2004;49 2:337-40.

Li K, Ye GY, Zhu JY and Hu C. Detection of food source by PCR analysis of the gut contents of Aldrichina grahami (Aldrich) (Diptera: Calliphoridae) during post-feeding period. Insect Sci. 2007; 14 1:47-52. doi:10.1111/j.1744-7917.2007.00124.x.

Xu H, Ye GY, Xu Y, Hu C and Zhu GH. Age-dependent changes in cuticular hydrocarbons of larvae in Aldrichina grahami (Aldrich) (Diptera: Calliphoridae). Forensic science international. 2014; 242: 236-41. doi:10.1016/j.forsciint.2014.07.003.

Moore HE, Adam CD and Drijfhout FP. Potential Use of Hydrocarbons for Aging Lucilia sericata Blowfly Larvae to Establish the Postmortem Interval. Journal of forensic sciences. 2013; 58 2:404-12. doi:10.1111/1556-4029.12016.

Line 80 & 84 & 91 & 109 & 236 & 305: Suggest to not begin a sentence with 'And'

RESPONSE: Changed. Thank you so much for your patient revision. (Line 87,91,98,124,236,317)

Line 95: As it appears here for the first time, please introduce "Hi-C" with its full name "Chromosome conformation capture"

RESPONSE: Changed as the reviewer suggested. (Line 102)

Genome Sequencing and Assembly:

Line 135: Refer to "SMRT cell" as Sequel SMRT cells, or PacBio SMRT cells

RESPONSE: Changed. (Line 157)

Line 141: How were the Hi-C libraries constructed?

RESPONSE: Thanks for the reviewer’s reminding. The procedure of Hi-C libraries construction has been added in the manuscript. (Line 163-173)

Reference

Rao S S P, Huntley M H, Durand N C, et al. A 3D map of the human genome at kilobase resolution reveals principles of chromatin looping. Cell, 2014, 159(7): 1665-1680.

Line 145: Estimated genome size was calculated using kmer-based calculation. Was there a reason why the kmer length of 17-mers was used (as opposed to >20-mers, or 25-mers which would ensure a more robust estimate). The authors should make this calculation using other kmer-lengths to ensure their calculations are correct.

RESPONSE: Great appreciation to the reviewer’s suggestion and reminding.

1) Before estimating genome size, the length of k-mer should be determined. It should be large enough to ensure that most k-mers are unique in the investigated genome, and small enough also to avoid the overload of computer memory. 17-mer was commonly used in the estimation of genome size. 2) Sequences in 17bp length consisted of four nucleotide base (ATCG) could theoretically form a 4^17, almost 17Gb sequence data in total, which could cover most insect genome assembly (based on statistics of Genome size web resource, http://www.genomesize.com/). 3) Errors can be generated in the sequencing process, and larger k-mer size will enhance the effect of heterozygosity and sequencing error. It is suggested using smaller k-mer size to analysis data with high heterozygosity and sequencing error rates. In addition, to prevent the interference of the palindrome sequence in the performing the De Berjin analyses, odd base number in k-mer length was used.

Reference

Binghang, Liu & Shi, Yujian & Yuan, Jianying & Galaxy, Yuuki & Zhang, Hao & Li, Nan & Li, Zhenyu & Chen, Yanxiang & Mu, Desheng & Parkin, Isobel. (2013). Estimation of genomic characteristics by analyzing k-mer frequency in de novo genome projects.

Table 1: "Pac Bio" is mis-spelled

RESPONSE: Changed. (Table 1)

Line 175: Suggest replacing "proved" with "supported"

RESPONSE: Changed as suggested. Thanks. (Line 206)

Line 198: Which D. melanogaster genome was used? Please reference

RESPONSE: The genome of D. melanogaster was downloaded from the following link

https://www.ncbi.nlm.nih.gov/genome/47. We have added this link as reference in new manuscript. (Line 383)

Line 200: Please elaborate on MCScanX methodology
RESPONSE: The MCScanX methodology has been elaborated in the revised manuscript. (Line 383-386)
Reference
Wang Y, Tang H, DeBarry J D, et al. MCScanX: a toolkit for detection and evolutionary analysis of gene synteny and collinearity. Nucleic acids research, 2012, 40(7): e49-e49.
Functional Prediction and Genome Annotation:

Line 209: Change "was" to "were" - verb tense error
RESPONSE: Changed. (Line 221)

Line 211: Microsatellite is used here, instead of SSR - suggest consistent use of one term
RESPONSE: Thank you for your suggestion. It has been hanged. (Line 223)

Table 2: Suggest clarifying the description of "Other*"
RESPONSE: Thank you for your suggestion. We have clarified the description of "Other*" in the note of Table 2. (Line 241)

Line 237: Which D. melanogaster gene model?
RESPONSE: The gene model used in present manuscript is under the following link https://www.ncbi.nlm.nih.gov/genome/47. We cited it in the manuscript. (Line 251)

Line 247: Spelling error "homologys"
RESPONSE: Changed. (Line 260)

Line 248: Spelling error "finial"
RESPONSE: Changed. (Line 261)

Table 3: Suggest replacing "Swissprot" with "Swiss-Prot", "Trembl" with "TrEMBL", "Kegg" with "KEGG", "InterProscan" with "InterProScan". What does "nr" stand for? Non-redundant? Please clarify.
RESPONSE: Thanks a lot for your kind suggestion. We have changed all the database names to the formal pattern both in the manuscript and additional files. The 'nr' used in the manuscript is refer to 'Non-Redundant Protein Sequence Database'. Thank you for pointing out this problem. (Line 265-273, table 3)

Evolutionary analyses:
Line 277: Change "exploring" to "explore" - verb tense error
RESPONSE: Changed. (Line 346)

Line 280: Please reference these genomic resources
RESPONSE: The links of these resources were referenced and also listed in the additional file Table S7. (Line 292)

Line 282: Why was BLASTP done independently prior to OrthoMCL?
RESPONSE: Thank you for pointing out this problem. The previous statement was ambiguous. In fact, the OrthoMCL starts with all-against-all BLASTP comparisons of a set of protein sequences from genomes. We have rephrased it. (Line 292)
Reference
Li L, Stoeckert C J, Roos D S. OrthoMCL: identification of ortholog groups for eukaryotic genomes. Genome research, 2003, 13(9): 2178-2189.

Line 287: Clarify what "statistics results" are referred to here
RESPONSE: The previous statement was inappropriate. It should not be a 'statistics results'. We have rephrased this statement. Thanks for your comment. (Line 298)

Line 293: These phylogenetic analyses are not clear. Please add detail.
RESPONSE: The phylogenetic analyses are described in the Lines 338-340.

Line 306: How were these analyses performed?
RESPONSE: These analyses were performed by an online Venn drawing tool. We have added this

description in the new manuscript. (Line 316)

Figure 6: Spelling error "notes"
RESPONSE: Changed. (Line 342)

Line 334: Change "was" to "were" - verb tense error
RESPONSE: Changed. (Line 347)

Line 337: Is "extinct" the correct term?
RESPONSE: Thank you for your reminding. We have changed it to "lost". (Line 350)

Line 342: Change "was" to "were" - verb tense error
RESPONSE: Changed. (Line 356)

Line 344: Suggest replacing "Blastp" with "BLASTP" to be consistent with line 282
RESPONSE: Changed as suggested. Thanks (Line 357)
Conclusion:

Line 353: Change "forensically" to "forensic" - verb tense error
RESPONSE: Changed. (Line 411)

Line 354: Spelling error "recourse"
RESPONSE: Changed. (Line 412)

Line 362: Provide references for this statement, as this is the first mention of repellent development.
RESPONSE: It is really true as Reviewer suggested. Thanks a lot to the reviewer for pointing out this insufficient statement. The common method to prevent and treat flystrike was relied on insecticides. But the rapid emergence of resistance in blowflies is becoming problematic. So repellent could be a better choice and predicting method to prevent flysrtike. For there has no more documentation on repellent development in our present manuscript, and it's not the main focus for we work, we would like to delete this phrase. (Line 418)

References
A vast number of references cited in the text did not match the reference list. Will the RRID citations be replaced with URLs?
RESPONSE: Thank you so much for pointing out this mistake in our references. It was caused by auto-update of citation index at different personal PC which has different reference assemblage. We have carefully revised all the references in this new submitted manuscript. Thanks again.

Supplementary Material:
Table S1: What does "inter size" refer to?
RESPONSE: A mistyping input. It should be 'insert'. We have changed it.

Fig S2: Spelling error "Brach"
RESPONSE: Changed. (Line 432)

Table S8: Please confirm gene count numbers. The number for L. cuprina is incorrect.
RESPONSE: Thank you for your kind advice. These genome resources listed in Table S8 were downloaded from the website, except A. grahami. And the gene numbers of each species were generated by our analyses on these resources by ourselves but not referenced from literature. For example, the version of GCF_000699065.1 of Lucilia cuprina was used here. When analyzing the length and number of gene in L. cuprina genome, the non-coding RNA (lncRNA, ncRNA, tRNA etc.) were removed in the annotation file. After this filtering procedure, 15536 protein-coding genes were obtained finally, which different from the literature reported (14554 genes, Clare A. Anstead et al, 2014).

Reviewer #2: The authors do an admirable job of assembling and annotating a blow fly genome. This is an interesting species to study and if published this genome will be a valuable resource for further blow fly research. There are some interpretations and analyses that should be improved before publication.
RESPONSE: We would like to give our great appreciation to you for your positive comments on our work. And your suggestions have priceless helpful and encouragement on improving our manuscript. According to your advice, we have amended the relevant parts and add the additional analyses as you suggested in the new version of the manuscript.

Reviewer #2: There are some minor language issues throughout the document that could be helped by another round of editing, especially when deciding to use singular versus plural forms of a word. There are also some misuses of words (ex. Recourse vs. Resource). Similarly, it would help to standardize language in the paper. For instance, PacBio or Pacbio?
RESPONSE: Thanks for reviewer's suggestion. We have revised the language problem of the whole manuscript. And we have corrected those misuses of proper nouns to the formal pattern. For example, Pacbio to PacBio, Kegg to KEGG, Trembl to TrEMBL, etc. (Line 265-273, table 3)

Reviewer #2: The authors do a good job of justifying the study of this species. In forensic entomology, it is worth noting that there are some assumptions associated with interpreting a minimum PMI, one of which the authors note later in the manuscript. It may help to read Tarone and Sanford 2017 to help clarify the conditional nature of PMImin interpretations in forensic entomology; especially if myiasis is associated with this fly.
RESPONSE: Thanks a lot for your kind advice. We highly agree with the author's (Tarone and Sanford, 2017) statements on the utility of terminology in forensic entomology study and practice. To avoid the possibly over- or underestimate of PMI, certain assumptions should be given before any particular use of terminology in forensic investigation or studies of the forensic entomologist, like minPMI, TOC, or PCI etc. And we will pay our attention to the new progress on this topic in the future. Here, in this revised version of our manuscript, we modified the statement related to minPMI and referenced the article (Tarone and Sanford 2017). (Line 61,64)

Reviewer #2: It would help to provide information regarding how the species was identified. Where/when/how was it collected? How was it identified? Is there a voucher associated with the specimen?
RESPONSE: Thank you very much for pointing out this insufficient description. The information was provided as the reviewer suggested. The first generation of A. grahami was collected by baits of beef liver, from City Changsha, Hunan province of China in March of 2017. They were brought back to the lab and breed up until eggs were laid down on supplied food resource. Then the eggs were collected and hatched, which were used to establish the laboratory population for further research. The species identification was performed through both the morphological and molecular method. We followed the morphological description on literature of Fan (1992) to distinguish the species. Then cytochrome oxidase gene I (COI) was used as molecular marker and was amplified (Primer F: 5-TACAATTTATCGCCTAAACTTCAGCC-3; R: 5-CATTTCAAGCTGTGTAAGCATC-3) from the DNA extraction of A. grahami. The product of amplification was sequenced and did the BLAST search on NCBI website. And it was recognized as A. grahami finally. The specimens used in the following genome sequencing test were all belong to the descendant of the original established laboratory population. All voucher specimens were assigned with a unique code and deposited in forensic insect herbarium of department of forensic science, Central South University, Changsha. We have added brief descriptions and results into the new version of the manuscript. (Line 106-115)
Reference
Fan, Z. D. 1992. Key to the common flies of China, Science publishing house, Beijing, China.

Reviewer #2: Similarly, what was specifically sequenced? Males? Females? Both?
RESPONSE: The samples used for genome survey, genome sequencing and Hi-C in present work were new emerged and unmated females. And samples used for transcriptom sequencing were new emerged individual of both sexes. We have added this information into relevant parts of the new version of manuscript. (Line 126-132)

Reviewer #2: With respect to genome size calculated in the document: There are different ways of assessing genome size. Not surprisingly, people that use specific techniques tend to prefer their method for estimating genome size. Many researchers tend to use PCR or sequence to estimate genome size, while others use flow cytometry (and some have used Feulgen densitometry). These methods do not always agree. One appealing aspect of non-PCR / sequencing methods is that they may not be as sensitive to issues associated with repeat sequences. If one evaluates Lucilia cuprina genome size by sequencing versus cytometry, there are very different measures. One can interpret this as cytometry overestimating genome size or as sequencing underestimating it. One feels most comfortable with an assembled genome size when the measures converge. It may be worth doing some cytometry on the strain. If not possible, it is at least worth mentioning that it was not done and that sometimes these measures do not agree.
As a good example of what I am discussing above, see: Comparisons with Caenorhabditis (~100 Mb) and Drosophila (~175 Mb) Using Flow Cytometry Show Genome Size in Arabidopsis to be ~157 Mb and

thus ~25 % Larger than the Arabidopsis Genome Initiative Estimate of ~125 Mb.
MICHAEL D. BENNETT ILIA J. LEITCH H. JAMES PRICE J. SPENCER JOHNSTON
Annals of Botany, Volume 91, Issue 5, April 2003, Pages 547-557
RESPONSE: Thank you so much for your constructive suggestion and reminding on the method of estimation on genome size. As you suggested, we performed the flow cytometry on the both sexes of A. grahami and compared the results with the previous one based on K-mer analysis. We used fruit fly as the internal stander. And single head of male or female A. grahami was sampled following the procedure of literature (PICARD, JOHNSTON & TARONE, 2012) and performed flow cytometry. The genome sizes of male (667.5 ± 6.013Mb, N=6) and female (682.5 ± 13.64Mb, N=6) has no significant difference (P-value 0.3388). But it is about 15.9% larger than the K-mer based genome size (582.63Mb) that exhibits a similar situation described in the paper you suggested. And it is also 12.5% larger than the assembly genome size (600MB). We add these results to the revised version, and describe the analyzing procedure in an additional file Figure S2. Thanks again for your suggestion on the reference literature and example of study. (Line 208-217)
Reference
Picard, C. J., J. S. Johnston, and A. M. Tarone. Genome Sizes of Forensically Relevant Diptera. Journal of Medical Entomology 49.1(2012):192-197.

Reviewer #2: Since the authors have produced predicted chromosomes, there are some obvious missing analyses. For instance, what Muller elements correspond to what chromosomes? In addition, are there repeats, or gene families, gene gain/loss, or genes with high rates of evolution that are biased on certain chromosomes or regions of certain chromosomes? Is there a predicted sex chromosome?
RESPONSE: We are very grateful to your suggestion. To check the similarity between A. grahami genome and the published fruit fly (D. melanogaster) genome. We identified the Muller elements on the assembled genome and summarized the results in Figure 6A and Supplementary Table 11 and added the outcomes and the explanations at lines 338-391. The result shows that, the major part of pseudochromosome of A. grahami is collinear with one Muller element of D. melanogaster (AgChr01-Muller B, AgChr02-Muller D, AgChr03-Muller A, AgChr04-Muller C, AgChr06-Muller E), except Chr05. Chr05 had one corresponding region on Muller D and E respectively. And Muller E largely corresponded to AgChr05 and AgChr06. No A. grahami chromosome was collinear with Muller F. For the sex chromosome, since Muller A (chromosome X of fruit fly) was uniquely located on Chr03 of A. grahami, thus we expect it should represent the sex chromosome of A. grahami.
In addition, we investigated the distributions of long terminal repeat (LTR), gene family expansion or contraction, and genes under positive selection, et al. on the genome using a window size of 1 Mb across each chromosome and plotted the distributions in Figure 6B and add the corresponding explanations in the figure note. We haven't found gene number enrichment for any particular chromosomes - all the chromosomes contain a gene density of around 20 genes/per Mb. However, the results showed that longer chromosomes tend to contain higher number of LRT, except for the case of Chr05. In addition, we noticed that the LTR enriched on specific regions of each chromosome where could present the centromere locations. We also summarized the genome regions that contain a significant greater number of positive genes or gene families (expansion or contraction) in Table S11. A brief description has been added at Lines 392-400. Thanks again.

Reviewer #2: The presence and absence of Phormia regina in different analyses is conspicuous. Its absence in genome evolution analyses is especially problematic, as it would help resolve issues related to tribe differences in blow flies. It should be included in analyses. If not, please explain why it was not. It may have the lowest BUSCO score in the set analyzed, but it is also highly related to this fly compared to the other flies analyzed. A blow fly targeted analysis is justified.
RESPONSE: Thank you for your suggestion. But the uploaded GFF of Phormia regina following the link on NCBI website (https://www.ncbi.nlm.nih.gov/genome/36631) is not available. So we did not use it in our analysis. The relevant data of Phormia regina used in this manuscript is referenced from previous report. And we are also looking forward to add genome resource of this highly related species in our further study.

Reviewer #2: Are there citations for all genomes used in this analysis? Please confirm that all are published.
RESPONSE: Thank you for your advice. We add those genome resource used in present paper as references in the revised version. These genome resources also can be downloaded following the links listed in the additional file.

Reviewer #2: I would suggest checking citation orders. Some do not appear to line up with their context in the manuscript.

RESPONSE: Many thanks for your suggestion. We were also aware of the mismatches references in previous version. It was caused by an auto-upload of references list by software at different computer. We carefully check the reference list in the new version of our manuscript.

Close
