# Supplementary material for: Chromosome-level genome assembly of Aldrichina grahami, a forensically important blowfly
Source: Gigascience. 2020 Mar 19;9(3):giaa020. doi: 10.1093/gigascience/giaa020 (PMC7081965; doi:10.1093/gigascience/giaa020)
Supplement: giaa020_Reviewer_1_Report_Original_Submission — Clare Anstead -- 4/18/2019 Reviewed [file giaa020_reviewer_1_report_original_submission.pdf]

Reviewer Report

Title: Chromosomal-level genome assembly of Aldrichina grahami, a forensically important blow fly

Version: Original Submission **Date:** 4/18/2019

Reviewer name: Clare Anstead

Reviewer Comments to Author:

This manuscript describes the sequencing, assembly and analysis of the genome of Aldrichina grahami, a forensically important blow fly. The authors used long-read PacBio sequencing and Hi-C scaffolding technology to produce chromosome-level scaffolds. Considering its forensic importance, the draft genome of A. grahami will be a valuable resource in comparative genomics analyses of other necrophagous dipterans, and arthropods more broadly.
Although the genome assembly appears to be of a high-quality, the manuscript suffers from lack of clarity in several sections and grammatical/language issues, which likely impact on the interpretation of findings.
Herewith is a list of some of the issues that need to be addressed.
Abstract:
Line 21: Clarify/rephrase introductory sentence
Line 25: Change "provides" to "provide" - verb tense error
Line 26: Please elaborate on "forensic issues"
Line 28 & 94: "Pacific Biosciences" is mis-spelled
Line 29: Change "Totally" to "A total of"
Line 30: Elaborate. FALCON and Wtdbg are genome sequence assemblers for PacBio long reads
Line 30: Change "was" to "were" - verb tense error
Line 34 & 190: Clarify "Nearly 96.4% of these scaffolds was anchored to A. grahami genome"
Line 36: Clarify what "de novo and transcriptome" data was used for genome annotation. Are these data deposited and available online?
Line 40: Change "add" to "adds", and "facilitate" to "facilitates" - verb tense errors
Line 41: "Necrophagous" is mis-spelled
Data Description:
Line 47: Clarify/rephrase sentence
Line 52: Is "it" A. grahami or the minPMI - please clarify
Line 57: Clarify/rephrase sentence
Line 57: When referring to "life history" do you mean life cycle?
Figure 1: Remove "remain" from figure legend
Line 72: Combine both sentences, or rephrase
Line 72: When referring to "whole life history" do you mean whole life cycle?
Line 74: When referring to "season of death" do you mean reason of death?
Line 76: Clarify and elaborate why these studies indicate great potential application value
Line 80 & 84 & 91 & 109 & 236 & 305: Suggest to not begin a sentence with

'And'
Line 95: As it appears here for the first time, please introduce "Hi-C" with its full name "Chromosome conformation capture"
Genome Sequencing and Assembly:
Line 135: Refer to "SMRT cell" as Sequel SMRT cells, or PacBio SMRT cells
Line 141: How were the Hi-C libraries constructed?
Line 145: Estimated genome size was calculated using kmer-based calculation. Was there a reason why the kmer length of 17-mers was used (as opposed to >20-mers, or 25-mers which would ensure a more robust estimate). The authors should make this calculation using other kmer-lengths to ensure their calculations are correct.
Table 1: "Pac Bio" is mis-spelled
Line 175: Suggest replacing "proved" with "supported"
Line 198: Which D. melanogaster genome was used? Please reference
Line 200: Please elaborate on MCScanX methodology
Functional Prediction and Genome Annotation:
Line 209: Change "was" to "were" - verb tense error
Line 211: Microsatellite is used here, instead of SSR - suggest consistent use of one term
Table 2: Suggest clarifying the description of "Other*"
Line 237: Which D. melanogaster gene model?
Line 247: Spelling error "homologys"
Line 248: Spelling error "finial"
Table 3: Suggest replacing "Swissprot" with "Swiss-Prot", "Trembl" with "TrEMBL", "Kegg" with "KEGG", "InterProscan" with "InterProScan". What does "nr" stand for? Non-redundant? Please clarify.
Evolutionary analyses:
Line 277: Change "exploring" to "explore" - verb tense error
Line 280: Please reference these genomic resources
Line 282: Why was BLASTP done independently prior to OrthoMCL?
Line 287: Clarify what "statistics results" are referred to here
Line 293: These phylogenetic analyses are not clear. Please add detail.
Line 306: How were these analyses performed?
Figure 6: Spelling error "notes"
Line 334: Change "was" to "were" - verb tense error
Line 337: Is "extinct" the correct term?
Line 342: Change "was" to "were" - verb tense error
Line 344: Suggest replacing "Blastp" with "BLASTP" to be consistent with line 282
Conclusion:
Line 353: Change "forensically" to "forensic" - verb tense error
Line 354: Spelling error "recourse"
Line 362: Provide references for this statement, as this is the first mention of repellent development
References
A vast number of references cited in the text did not match the reference list. Will the RRID citations be replaced with URLs?

Supplementary Material:
Table S1: What does "inter size" refer to?
Fig S2: Spelling error "Brach"
Table S8: Please confirm gene count numbers. The number for L. cuprina is incorrect.

Level of Interest

Please indicate how interesting you found the manuscript: Choose an item.

Quality of Written English

Please indicate the quality of language in the manuscript: Choose an item.

Declaration of Competing Interests

Please complete a declaration of competing interests, considering the following questions:

- Have you in the past five years received reimbursements, fees, funding, or salary from an organisation that may in any way gain or lose financially from the publication of this manuscript, either now or in the future?
- Do you hold any stocks or shares in an organisation that may in any way gain or lose financially from the publication of this manuscript, either now or in the future?
- Do you hold or are you currently applying for any patents relating to the content of the manuscript?
- Have you received reimbursements, fees, funding, or salary from an organization that holds or has applied for patents relating to the content of the manuscript?
- Do you have any other financial competing interests?
- Do you have any non-financial competing interests in relation to this paper?

If you can answer no to all of the above, write 'I declare that I have no competing interests' below. If your reply is yes to any, please give details below.

I declare that I have no competing interests.

I agree to the open peer review policy of the journal. I understand that my name will be included on my report to the authors and, if the manuscript is accepted for publication, my named report including any attachments I upload will be posted on the website along with the authors' responses. I agree for my report to be made available under an Open Access Creative Commons CC-BY license (http://creativecommons.org/licenses/by/4.0/). I understand that any comments which I do not wish to be included in my named report can be included as confidential comments to the editors, which will not be published.

Choose an item.

To further support our reviewers, we have joined with Publons, where you can gain additional credit to further highlight your hard work (see: https://publons.com/journal/530/gigascience). On publication of this paper, your review will be automatically added to Publons, you can then choose whether or not to claim your Publons credit. I understand this statement.

Yes Choose an item.
