# Supplementary material for: Chromosome-level genome assembly of Aldrichina grahami, a forensically important blowfly
Source: Gigascience. 2020 Mar 19;9(3):giaa020. doi: 10.1093/gigascience/giaa020 (PMC7081965; doi:10.1093/gigascience/giaa020)
Supplement: giaa020_Reviewer_1_Report_Revision_1 — Clare Anstead -- 8/20/2019 Reviewed [file giaa020_reviewer_1_report_revision_1.pdf]

Reviewer Report

Title: Chromosomal-level genome assembly of Aldrichina grahami, a forensically important blow fly

Version: Revision 1 **Date:** 8/20/2019

Reviewer name: Clare Anstead

Reviewer Comments to Author:

This R1 manuscript describes the sequencing, assembly and analysis of the genome of Aldrichina grahami, a forensically important blow fly. The authors used long-read PacBio sequencing and Hi-C scaffolding technology to produce chromosome-level scaffolds. Considering its forensic importance, the draft genome of A. grahami will be a valuable resource in comparative genomics analyses of other necrophagous dipterans, and arthropods more broadly.
Thank you to the authors for addressing each point in the reviewer's reports, and taking the time to perform the suggested changes/analyses. I believe the resulting R1 manuscript is of a much higher quality.
However, herewith is a list of some of the issues that still need to be addressed.
Abstract:
Line 25/26: Clarify/rephrase introductory sentence as it is still not clear
Line 34: Change "program" to "programs"
Line 36, 76, 82, 114: Suggest to not begin a sentence with 'And'
Line 43: Clarify "Nearly 96.4% of these scaffolds was anchored to A. grahami genome" as it is still not clear. Is there a different way to describe this?
Data Description:
Line 52: Clarify "medical legal investigation" and how arthropods are used
Line 54: Change "corpse" to "corpses"
Functional Prediction and Genome Annotation:
Table 2: Suggest clarifying the description of "and etc"
Line 269: Clarify "of InterPro with InterProScan"
Evolutionary analyses:
Line 289: Why are these in italics? (necrophagias, parasitism or hematophagia)
Line 292: 'were' used - tense error
Line 299: 'contents' - is this 'contains'?
Line 368: Replace "bowtie2" with "Bowtie 2"
Line 371: 'were valid interaction pairs validated' - remove validated?
Line 385: Replace "blast" with "BLAST"
Line 393: Why 'et al.'?
Conclusion:
Line 410: Change "calyptratae" to "the Calyptratae"
Line 416-418: Please clarify closing sentence.

Level of Interest

Please indicate how interesting you found the manuscript: Choose an item.

Quality of Written English

Please indicate the quality of language in the manuscript: Choose an item.

Declaration of Competing Interests

Please complete a declaration of competing interests, considering the following questions:

- Have you in the past five years received reimbursements, fees, funding, or salary from an organisation that may in any way gain or lose financially from the publication of this manuscript, either now or in the future?
- Do you hold any stocks or shares in an organisation that may in any way gain or lose financially from the publication of this manuscript, either now or in the future?
- Do you hold or are you currently applying for any patents relating to the content of the manuscript?
- Have you received reimbursements, fees, funding, or salary from an organization that holds or has applied for patents relating to the content of the manuscript?
- Do you have any other financial competing interests?
- Do you have any non-financial competing interests in relation to this paper?

If you can answer no to all of the above, write 'I declare that I have no competing interests' below. If your reply is yes to any, please give details below.

I declare that I have no competing interests.

I agree to the open peer review policy of the journal. I understand that my name will be included on my report to the authors and, if the manuscript is accepted for publication, my named report including any attachments I upload will be posted on the website along with the authors' responses. I agree for my report to be made available under an Open Access Creative Commons CC-BY license (http://creativecommons.org/licenses/by/4.0/). I understand that any comments which I do not wish to be included in my named report can be included as confidential comments to the editors, which will not be published.

Choose an item.

To further support our reviewers, we have joined with Publons, where you can gain additional credit to further highlight your hard work (see: https://publons.com/journal/530/gigascience). On publication of this paper, your review will be automatically added to Publons, you can then choose whether or not to claim your Publons credit. I understand this statement.

Yes Choose an item.
