# Supplementary material for: Chromosome-level genome assembly of Aldrichina grahami, a forensically important blowfly
Source: Gigascience. 2020 Mar 19;9(3):giaa020. doi: 10.1093/gigascience/giaa020 (PMC7081965; doi:10.1093/gigascience/giaa020)
Supplement: giaa020_Reviewer_2_Report_Original_Submission — Aaron Tarone -- 4/19/2019 Reviewed [file giaa020_reviewer_2_report_original_submission.pdf]

Reviewer Report

Title: Chromosomal-level genome assembly of Aldrichina grahami, a forensically important blow fly

Version: Original Submission **Date:** 4/19/2019

Reviewer name: Aaron Tarone

Reviewer Comments to Author:

The authors do an admirable job of assembling and annotating a blow fly genome. This is an interesting species to study and if published this genome will be a valuable resource for further blow fly research. There are some interpretations and analyses that should be improved before publication.

There are some minor language issues throughout the document that could be helped by another round of editing, especially when deciding to use singular versus plural forms of a word. There are also some misuses of words (ex. Recourse vs. Resource). Similarly, it would help to standardize language in the paper. For instance, PacBio or Pacbio?

The authors do a good job of justifying the study of this species. In forensic entomology, it is worth noting that there are some assumptions associated with interpreting an minimum PMI, one of which the authors note later in the manuscript. It may help to read Tarone and Sanford 2017 to help clarify the conditional nature of PMImin interpretations in forensic entomology; especially if myiasis is associated with this fly.

It would help to provide information regarding how the species was identified. Where/when/how was it collected? How was it identified? Is there a voucher associated with the specimen?

Similarly, what was specifically sequenced? Males? Females? Both?

With respect to genome size calculated in the document: There are different ways of assessing genome size. Not surprisingly, people that use specific techniques tend to prefer their method for estimating genome size. Many researchers tend to use PCR or sequence to estimate genome size, while others use flow cytometry (and some have used Feulgen densitometry). These methods do not always agree. One appealing aspect of non-PCR / sequencing methods is that they may not be as sensitive to issues associated with repeat sequences. If one evaluates Lucilia cuprina genome size by sequencing versus cytometry, there are very different measures. One can interpret this as cytometry overestimating genome size or as sequencing underestimating it. One feels most comfortable with an assembled genome size when the measures converge. It may be worth doing some cytometry on the strain. If not possible, it is at least worth mentioning that it was not done and that sometimes these measures do not agree.

As a good example of what I am discussing above, see:

Comparisons with Caenorhabditis (âˆ¼100 Mb) and Drosophila (âˆ¼175 Mb) Using Flow Cytometry Show Genome Size in Arabidopsis to be âˆ¼157 Mb and thus âˆ¼25 % Larger than the Arabidopsis Genome Initiative Estimate of âˆ¼125 Mb

MICHAEL D. BENNETT ILIA J. LEITCH H. JAMES PRICE J. SPENCER JOHNSTON

Annals of Botany, Volume 91, Issue 5, April 2003, Pages 547-557

Since the authors have produced predicted chromosomes, there are some obvious missing analyses. For

instance, what Muller elements correspond to what chromosomes? In addition, are there repeats, or gene families, gene gain/loss, or genes with high rates of evolution that are biased on certain chromosomes or regions of certain chromosomes? Is there a predicted sex chromosome?
The presence and absence of Phormia regina in different analyses is conspicuous. Its absence in genome evolution analyses is especially problematic, as it would help resolve issues related to tribe differences in blow flies. It should be included in analyses. If not, please explain why it was not. It may have the lowest BUSCO score in the set analyzed, but it is also highly related to this fly compared to the other flies analyzed. A blow fly targeted analysis is justified.
Are there citations for all genomes used in this analysis? Please confirm that all are published.
I would suggest checking citation orders. Some do not appear to line up with their context in the manuscript.

Level of Interest

Please indicate how interesting you found the manuscript: Choose an item.

Quality of Written English

Please indicate the quality of language in the manuscript: Choose an item.

Declaration of Competing Interests

Please complete a declaration of competing interests, considering the following questions:

- Have you in the past five years received reimbursements, fees, funding, or salary from an organisation that may in any way gain or lose financially from the publication of this manuscript, either now or in the future?
- Do you hold any stocks or shares in an organisation that may in any way gain or lose financially from the publication of this manuscript, either now or in the future?
- Do you hold or are you currently applying for any patents relating to the content of the manuscript?
- Have you received reimbursements, fees, funding, or salary from an organization that holds or has applied for patents relating to the content of the manuscript?
- Do you have any other financial competing interests?
- Do you have any non-financial competing interests in relation to this paper?

If you can answer no to all of the above, write 'I declare that I have no competing interests' below. If your reply is yes to any, please give details below.

I do fly genomics and forensic entomology research, if you consider that to be in competition with this paper.

I agree to the open peer review policy of the journal. I understand that my name will be included on my report to the authors and, if the manuscript is accepted for publication, my named report including any attachments I upload will be posted on the website along with the authors' responses. I agree for my report to be made available under an Open Access Creative Commons CC-BY license (http://creativecommons.org/licenses/by/4.0/). I understand that any comments which I do not wish to be included in my named report can be included as confidential comments to the editors, which will not be published.

Choose an item.

To further support our reviewers, we have joined with Publons, where you can gain additional credit to further highlight your hard work (see: https://publons.com/journal/530/gigascience). On publication of this paper, your review will be automatically added to Publons, you can then choose whether or not to claim your Publons credit. I understand this statement.

Yes Choose an item.
