# Supplementary material for: Chromosome-level genome assembly of Aldrichina grahami, a forensically important blowfly
Source: Gigascience. 2020 Mar 19;9(3):giaa020. doi: 10.1093/gigascience/giaa020 (PMC7081965; doi:10.1093/gigascience/giaa020)
Supplement: giaa020_Reviewer_2_Report_Revision_1 — Aaron Tarone -- 8/20/2019 Reviewed [file giaa020_reviewer_2_report_revision_1.pdf]

Reviewer Report

Title: Chromosomal-level genome assembly of Aldrichina grahami, a forensically important blow fly

Version: Revision 1 **Date:** 8/20/2019

Reviewer name: Aaron Tarone

Reviewer Comments to Author:

The authors did a good job of beginning to address the issues raised in the previous reviews. However, there are some areas of concern to address with the new round.

1. The authors should make more of an attempt to either standardize or justify specific comparative genomic comparisons they are making. The excuse for not including Phormia regina in all analyses seems wrong. If NCBI has a faulty link, authors should work with NCBI to fix it and gain access to the genome. Also, did the manuscript authors try contacting the authors of the Phormia regina genome paper to see if they can provide?
2. The authors are to be commended for their reevaluation of genome size based on flow cytometry. However, a few items are concerning in their description and implementation of the work. First, in supplemental data the authors clarify they used Drosophila melanogaster as the reference genome, but they do not report the genus or species name in the main text. Since D. virilis is a common genome size reference, the authors should clarify which species (and strain) they analyzed to identify genome size. It would also help to see the genome size the authors are assuming (175 Mb for D. melanogaster?). The CV values for the histograms appear to be unacceptably large (I believe <3 is considered good practice). Finally, when one looks at the genome size histograms there appears to be a linearity issues with the analysis. The peaks for 2C/4C do not appear with 4C as 2x of 2C. This can be a problem with some cytometers, which do not all report fluorescence in a linear manner. This could indicate a biased genome size estimate.
3. Muller element analyses are good, but authors need to delve into the literature on this more. Muller F is sometimes a sex chromosome. Others can be associated with sex in other species too. See work by Doris Bachtrog, Rich Meisel, and Max Scott for higher flies (including blow flies and house/stable flies). In addition, coverage analysis might help authors determine elements that differ in coverage between the sexes. It is not sufficient to imply a sex chromosome because it is the sex chromosome in Drosophila melanogaster.

Minor

Uningnorable is a difficult word to read through. Maybe replace with "a detectable and potentially important"

Report COI sequence to a database (Nucleotide database on NCBI?)

Provide the specific voucher number for the specimens submitted as vouchers to the museum

Why were wings/gut used to provide DNA? Or was DNA isolated without wings and gut?

Why do authors sometimes italicize necrophagia/parasitism/hematophagia?

More justification for parameter settings on divergence time analyses would help.

On line 146 - which fragments were proper size?

Level of Interest

Please indicate how interesting you found the manuscript: Choose an item.

Quality of Written English

Please indicate the quality of language in the manuscript: Choose an item.

Declaration of Competing Interests

Please complete a declaration of competing interests, considering the following questions:

- Have you in the past five years received reimbursements, fees, funding, or salary from an organisation that may in any way gain or lose financially from the publication of this manuscript, either now or in the future?
- Do you hold any stocks or shares in an organisation that may in any way gain or lose financially from the publication of this manuscript, either now or in the future?
- Do you hold or are you currently applying for any patents relating to the content of the manuscript?
- Have you received reimbursements, fees, funding, or salary from an organization that holds or has applied for patents relating to the content of the manuscript?
- Do you have any other financial competing interests?
- Do you have any non-financial competing interests in relation to this paper?

If you can answer no to all of the above, write 'I declare that I have no competing interests' below. If your reply is yes to any, please give details below.

I work in blow fly genomics, but not on their species.

I agree to the open peer review policy of the journal. I understand that my name will be included on my report to the authors and, if the manuscript is accepted for publication, my named report including any attachments I upload will be posted on the website along with the authors' responses. I agree for my report to be made available under an Open Access Creative Commons CC-BY license (http://creativecommons.org/licenses/by/4.0/). I understand that any comments which I do not wish to be included in my named report can be included as confidential comments to the editors, which will not be published.

Choose an item.

To further support our reviewers, we have joined with Publons, where you can gain additional credit to further highlight your hard work (see: https://publons.com/journal/530/gigascience). On publication of

this paper, your review will be automatically added to Publons, you can then choose whether or not to claim your Publons credit. I understand this statement.

Yes Choose an item.
