# Supplementary material for: Chromosome-level genome assembly of Aldrichina grahami, a forensically important blowfly
Source: Gigascience. 2020 Mar 19;9(3):giaa020. doi: 10.1093/gigascience/giaa020 (PMC7081965; doi:10.1093/gigascience/giaa020)
Supplement: giaa020_Reviewer_2_Report_Revision_2 — Aaron Tarone -- 11/11/2019 Reviewed [file giaa020_reviewer_2_report_revision_2.pdf]

Reviewer Report

Title: Chromosomal-level genome assembly of Aldrichina grahami, a forensically important blow fly

Version: Revision 2 **Date:** 11/11/2019

Reviewer name: Aaron Tarone

Reviewer Comments to Author:

The authors have done an admirable job of addressing reviews. They have justified their cytometric estimates of genome size in much more detail and have addressed my questions about their claims regarding sex chromosomes.

Small comments for consideration: 1. I believe the genome size of D. melanogaster of 175 Mb is linked to the originally sequenced strain. There may be subtle differences between it and W1118. For example, see Ellis et al. 2014 in PLoS Genetics for variation in the DGRP strains of Drosophila melanogaster. Surely the authors are close enough to the true value for the purposes of this paper, but it is worth remembering. 2. For future reference, given the size of this genome, D. virilis may have been a better choice as it is closer to the size of what is of interest to the authors. Not necessary for this paper, but something to consider for future projects if authors continue to do this sort of work. 3. The fonts in some of the figures are low resolution and hard to read.

Level of Interest

Please indicate how interesting you found the manuscript: Choose an item.

Quality of Written English

Please indicate the quality of language in the manuscript: Choose an item.

Declaration of Competing Interests

Please complete a declaration of competing interests, considering the following questions:

- Have you in the past five years received reimbursements, fees, funding, or salary from an organisation that may in any way gain or lose financially from the publication of this manuscript, either now or in the future?
- Do you hold any stocks or shares in an organisation that may in any way gain or lose financially from the publication of this manuscript, either now or in the future?
- Do you hold or are you currently applying for any patents relating to the content of the manuscript?
- Have you received reimbursements, fees, funding, or salary from an organization that holds or has applied for patents relating to the content of the manuscript?
- Do you have any other financial competing interests?

- Do you have any non-financial competing interests in relation to this paper?

If you can answer no to all of the above, write 'I declare that I have no competing interests' below. If your reply is yes to any, please give details below.

I do research in this area. Technically, funding of my research is tied to results like this.

I agree to the open peer review policy of the journal. I understand that my name will be included on my report to the authors and, if the manuscript is accepted for publication, my named report including any attachments I upload will be posted on the website along with the authors' responses. I agree for my report to be made available under an Open Access Creative Commons CC-BY license (http://creativecommons.org/licenses/by/4.0/). I understand that any comments which I do not wish to be included in my named report can be included as confidential comments to the editors, which will not be published.

Choose an item.

To further support our reviewers, we have joined with Publons, where you can gain additional credit to further highlight your hard work (see: https://publons.com/journal/530/gigascience). On publication of this paper, your review will be automatically added to Publons, you can then choose whether or not to claim your Publons credit. I understand this statement.

Yes Choose an item.
